# Metabolic Adaptations of Benthic Forams: Foraminiferal Species Adaptations to Intertidal Mudflat Assessed by a Metabolic Approach

**DOI:** 10.1111/jeu.70051

**Published:** 2025-11-11

**Authors:** Julia Courtial, Jeremy Lothier, Caroline Cukier, Anis M. Limami, Emmanuelle Geslin

**Affiliations:** ^1^ Univ Angers, Nantes Université, Le Mans Univ, CNRS Laboratoire de Planétologie et Géosciences, LPG UMR 6112 Angers France; ^2^ Univ Angers, Institut Agro, INRAE, IRHS SFR QuaSaV Angers France

**Keywords:** *Ammonia confertitesta*, *Elphidium oceanense*, *Haynesina germanica*, kleptoplasty, metabolic adaptation

## Abstract

Benthic foraminifera are characterized by their rapid response and high resistance to variable and extreme conditions such as those typically found in intertidal environments. However, knowledge of cellular and metabolic adaptations by foraminifera remains incomplete. Here we explore the metabolic profile of three species from an intertidal mudflat: *Haynesina germanica* (kleptoplast, mixotrophic), *Elphidium oceanense*, and *Ammonia confertitesta* (heterotrophic). Given the challenges associated with culturing foraminifera, specimens were collected directly from the field. To analyze their metabolic profiles, a non‐targeted gas chromatography–mass spectrometry methodology was optimized with the aim of reducing sample size. We constructed a foraminifera‐specific library containing 382 features. Within the 30 metabolites identified, those present in all three species include osmolytes, oxidant‐ and thermo‐protective molecules, which are consistent with their high tolerance to variations in environmental conditions. Species‐specific features were also observed. *A. confertitesta* accumulates myoinositol, aspartate, and asparagine. *H. germanica* accumulated malate, glycerate, and glycolate/glyoxylate, indicating concurrent activity of a C4‐like carbon concentrating mechanism and photorespiratory metabolism. Our approach enabled us to discriminate between the species based on their metabolites and highlights two probable metabolic pathways not previously described in kleptoplast foraminifera. These metabolic adaptations likely contribute to the ecological success of intertidal foraminiferal species.

## 1 Introduction

Foraminifera are unicellular protists belonging to the clade Rhizaria (Adl et al. 2012), some of which produce hard shells. Shell‐bearing foraminifera live in various marine environments, from foreshore to deep basins, adopting either a planktonic or benthic lifestyle. Various marine benthic foraminiferal species live in specific habitats where they have particular ecological requirements (Murray 2006) and are able to develop specific life strategies and physiological adaptations (Tavera Martínez et al. 2023). In this context, we use “adaptation” to refer to functional traits or biochemical responses that improve an organism's survival and development under environmental constraints rather than implying experimentally proven evolutionary mechanisms. Consequently, some species respond rapidly to stress and exhibit varying degrees of tolerance to variations in environmental factors (Debenay et al. 2000). Overall, foraminifera exhibit a wide range of trophic strategies: they can be either heterotrophic or mixotrophic, omnivorous or herbivorous with varying degrees of food selectivity. Foraminifera can feed on diatoms, bacteria, detritus, metazoans, but can also take up inorganic nitrogen and carbon (Bird et al. 2020; Cesbron et al. 2017; Chronopoulou et al. 2019; Dupuy et al. 2010; Goldstein and Corliss 1994; Heinz et al. 2001, 2002; LeKieffre et al. 2018; Lintner et al. 2020; Mojtahid et al. 2011). Besides different feeding strategies, foraminifera can have different metabolic adaptation mechanisms such as denitrification (Risgaard‐Petersen et al. 2006), dormancy (Glock 2023; LeKieffre et al. 2017; Ross and Hallock 2016), symbiosis (Lee 2006), and kleptoplasty (Jauffrais et al. 2018, 2019; Lopez 1979). Kleptoplasty is a phenomenon observed in various species of benthic foraminifera, enabling the long‐term retention of intact chloroplasts from their diatom prey (Lopez 1979; Pinko et al. 2023). As shown for some kleptoplast species living in photic zones (such as *Haynesina germanica* or *Elphidium williamsoni*), kleptoplasts can fulfill different roles, like photosynthetic activity (Cesbron et al. 2017; Jauffrais et al. 2016; Jesus et al. 2022; Lopez 1979). Other kleptoplast foraminiferal species living deeper in aphotic marine environments are not photosynthetically active, but certain evidence suggests a role of kleptoplasts in nitrogen assimilation (Gomaa et al. 2021; Grzymski et al. 2002; Jauffrais et al. 2019; LeKieffre et al. 2018).

Among the different habitats where foraminifera are present, intertidal environments are particularly complex, with strong temporal and spatial variations. Additionally, over very short time scales (hours), tidal processes induce additional large‐scale variations in sediment dynamics (Widdows and Brinsley 2002), food availability (Méléder et al. 2007), and abiotic parameters (e.g., salinity, temperature, oxygen content, granulometry, redox conditions, and organic matter mineralization; Hulot et al. 2023; Méléder et al. 2007; Middelburg et al. 1996; Thibault de Chanvalon et al. 2017). For instance, in temperate French Atlantic mudflats, water temperature at high tide can vary between 5°C and 25°C, and monthly mean water salinity ranges between 28 and 36 (Dutertre et al. 2010; Soletchnik et al. 2007). At low tide, temperatures can rise to more than 34°C, with daily variations exceeding 15°C (Guarini et al. 1997). All in all, benthic foraminiferal species in an intertidal environment need to withstand numerous ecological constraints on both short‐ and long‐term scales. Since the composition and biomass of benthic microphytobenthos show high spatio‐temporal variations in the intertidal zone (Guarini et al. 1997; Jesus et al. 2005; Méléder et al. 2007; Soletchnik et al. 2007; Dutertre et al. 2010; Ribeiro et al. 2021), most intertidal foraminiferal species have specific feeding strategies, with herbivory being the dominant mode (Haynert et al. 2020; Schweizer et al. 2022). The dominant species of foraminifera in intertidal mudflat communities along the French Atlantic coast are species of *Ammonia* belonging to the *tepida* morphogroup (*A. confertitesta*, *A. aberdoveyensis*, *A. veneta* ), *H. germanica* , and members of the genus *Elphidium* (du Chatelet et al. 2009; Fouet et al. 2022; Pavard et al. 2023; Thibault de Chanvalon et al. 2015). Each of these species exhibits different trophic strategies. *Ammonia* spp. are heterotrophic, ubiquitous, opportunistic, and adaptive omnivores. They can use a wide variety of food sources, such as detrital organic matter, bacteria, fungi, microalgae, and nematodes (Bird et al. 2020; Dupuy et al. 2010; Lintner et al. 2020; Mojtahid et al. 2011; Murray 2006; Schweizer et al. 2022). *E. oceanense* is mainly herbivorous and can retain the chloroplasts of its diatom prey. However, these chloroplasts are damaged and are likely not photosynthetically active (Jauffrais et al. 2018). Lastly, *H. germanica* is strictly herbivorous, with a specific diet of large diatoms as demonstrated through direct observation (Austin et al. 2005) and DNA analyses revealing only DNA from pennate diatoms within its cells (Schweizer et al. 2022). This species is mixotrophic since it can perform kleptoplasty, i.e., retain the chloroplasts and photosynthetic activity for 7–21 days depending on light exposure (Jauffrais et al. 2016).

Although foraminifera serve as important biological models and bioindicators, their metabolism and physiology remain incompletely understood. Gaining a better understanding of these processes is essential to better understanding the ecological trends, life strategies, and the phenotypes observed for this particularly adapted organism. Although the amount of published foraminiferal omics data (genome, transcriptome, and proteome) is increasing and progressively enabling the deciphering of the metabolic pathways of these protists, the number of such studies remains unfortunately limited (Glöckner et al. 2014; Gomaa et al. 2021, 2025; Habura et al. 2011; Macher et al. 2021, 2023; Orsi et al. 2020; Pillet and Pawlowski 2013; Powers et al. 2022; Sabbatini et al. 2014; Sierra et al. 2022; Stuhr et al. 2021; Titelboim et al. 2021; Woehle et al. 2018). Furthermore, species with available omics data are taxonomically distant and live in highly contrasting ecological niches (freshwater environments, polar and tropical climates). Consequently, formulating general metabolic models applicable to the group is challenging, particularly for temperate intertidal foraminifera. The small number of omic data on foraminifera may be in part due to the challenges of maintaining and reproducing most species of foraminifera under controlled conditions. Indeed, given the wide diversity of ecological niches occupied by foraminifera and their species‐specific requirements, culture protocols developed for some species are not readily applicable to others. Therefore, the harvesting of individuals from in situ sediment remains necessary, in particular for smaller benthic foraminifera species.

The objective of this study is to increase our knowledge of the metabolism of three dominant species in foraminiferal communities of intertidal temperate mudflats, *H. germanica* , *A. confertitesta*, and *E. oceanense*. To achieve this goal, we developed the first protocol to determine the foraminifera metabolome by a non‐targeted GC–MS approach. Our results reveal metabolic differences among the three investigated species and provide insights into the metabolic exchanges between the kleptoplast and the cell of *H. germanica* . The nature of the metabolites identified in the metabolome can suggest functional adaptation to environmental constraints.

## 2 Materials and Methods

### 2.1 Field Site, Mud Sampling, and Foraminifera Collection

**FIGURE 1:**
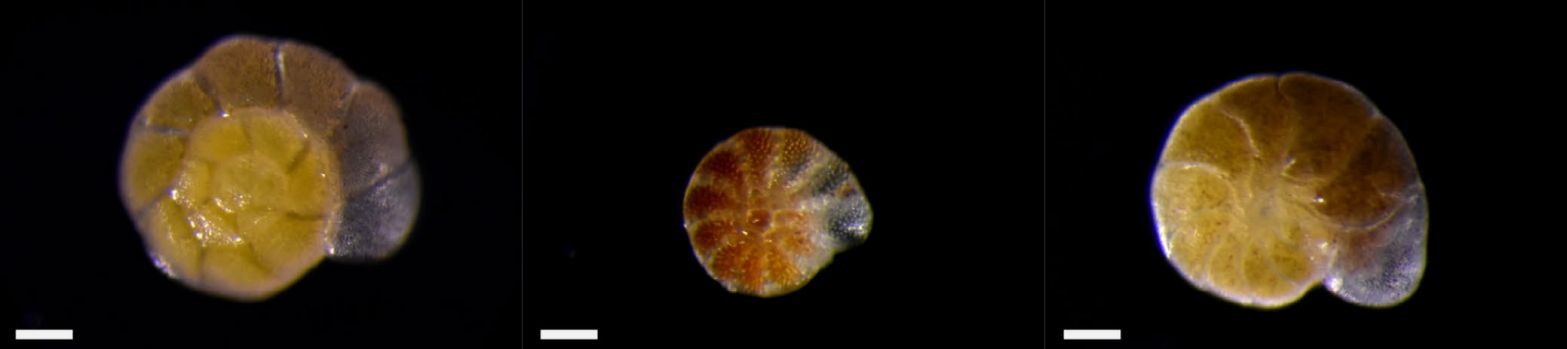
Light microscope images of the three dominant species of foraminifera from Bourgneuf Bay, France. From left to right: *Ammonia confertitesta*, *Elphidium oceanense* and *Haynesina germanica*. Scale bar = 100 μm.

The upper first 0.5 cm of sediment was collected from an intertidal mudflat located in the Bourgneuf bay, located south of the Loire estuary in France (47°0′56.67″ N, 2°1′27.16″ W). Sediment was sieved in the field over 500 and 150 μm using in situ seawater. This fraction contains adult specimens. The sieved fraction (150–500 μm) was transported to the laboratory and left to rest overnight in a flask with oxygenated seawater (maintained through continuous bubbling), in a temperature‐controlled room set to field temperature. The following day, living foraminiferal specimens (with colored cytoplasm) were selected and harvested from the 150 to 500 μm fraction with a brush under a stereomicroscope. Depending on the foraminiferal richness of the samples, it takes two operators between 4 and 12 h to obtain up to 600 foraminifera. The foraminifera population of this mudflat consists mainly of *Haynesina germanica* (Ehrenberg, 1840), *Elphidium oceanense* (d'Orbigny in Fornasini, 1904), and *Ammonia* spp., which are the focus of this study (Figure 1; Choquel 2021). The *Ammonia* spp. in Bourgneuf bay belong to the *tepida* morphogroup (Hayward et al. 2021), which includes three cryptic species found along the Atlantic west coast of France (Fouet et al. 2024; Richirt et al. 2021). These cryptic species are difficult to distinguish using stereomicroscope observations. However, in our specific study area, *Ammonia confertitesta* (*Zheng*, *1978*) dominates the *Ammonia* spp. morphocomplex (Choquel 2021). Another study using SEM images for the identification of *Ammonia* specimens from this location shows ~95% dominance of *A. confertitesta* (Daviray et al. 2025). We will therefore assume that our specimens belong to *A. confertitesta*. To date, no occurrence of in vitro reproduction has been published for *H. germanica* and *E. oceanense*, unlike for *Ammonia* belonging to the *tepida* morphogroup (Stouff et al. 1999; de Nooijer et al. 2014). This necessitates the direct collection of cells from sediment. Different cell pool sizes of 200, 300 (*A. confertitesta* and *H. germanica* ), and 600 (*A. confertitesta*) cells were tested to optimize our protocol for optimal sensitivity and varying availability of foraminifera. Due to fluctuations in the population dynamics of each species, samples were collected at different times in 2020. On February 24th, three pools of *H. germanica* (one of 300 and two of 200 cells) and two pools of *A. confertitesta* (200 and 300 cells) were picked from the sediment, whereas *E. oceanense* could not be sampled due to insufficient numbers. According to Choquel (2021), *E. oceanense* reaches densities of over 200 living individuals per 50 cm^−3^ around August. Consequently, three pools of 200 *E. oceanense* cells were collected from the sediment samples on August 24th, 2020, with no collection of *A. confertitesta* and *H. germanica* due to very low population densities. To obtain a third (winter) sample of *A. confertitesta*, an additional collection of 600 cells from the field was performed on December 16th. Picked living specimens were cleaned in Petri dishes using two successive baths of filtered seawater (< 0.2 μm), and surface particles were removed using a brush. They were then transferred three times to a Petri dish containing milli‐Q water using a micropipette. Each transfer was performed within a few seconds to minimize foraminifera exposure to osmotic stress and ensure effective removal of salts that might occlude the GC–MS column, after which samples were stored at –80°C until further analysis. The total process time from field to freezer never exceeded 48 h.

### 2.2 Foraminiferal Metabolites Extraction and Derivation

Foraminifera samples were suspended in a methanol–water mix (80:20 v/v) at 70°C, using a volume of 500 μL for pools of 200 and 300 cells and 1 mL for the pool of 600 cells. Three rounds of metabolite extractions were performed as described below. At the beginning of the first round, 1 μL of ribitol (2.5 mM) was added as an internal standard. Then, shells and cells were broken using a sterile polypropylene micro‐pestle in a 1.5 mL microtube, followed by 10 min of sonication in an ultrasonic tank (47 KHz; BRANSON B1210E‐MT). This ultrasonic treatment aims to reduce processing time and enhance extraction efficiency. Samples were centrifuged at 14,000 *g* for 15 min to separate the cell and shell debris. The supernatant was collected in a new microtube. Following the three extraction rounds, the pooled supernatant metabolic extracts were vacuum dried and stored at −20°C until analysis. For derivatization, the samples were re‐suspended in 40 μL of methoxyamine HCl in pyridine (40 mg mL^−1^) and incubated for 2 h at 50°C in a dry bath heater. Subsequently, 40 μL of N‐methyl‐N‐(trimethylsilyl)‐trifluoroacetamide (MSTFA) and a standard n‐alkanes mix (C10–C36; 28 mg mL^−1^) were added. The samples were incubated in a dry water bath at 50°C for 30 min. Gas chromatography coupled to mass spectrometry (GC–MS) analyses were carried out on the extracts following Malécange et al. (2022). In short, GC–MS analyses were performed using a 436‐GC system coupled to a SCION SQ MS (Bruker) equipped with an RTX‐5 column (30 m × 0.25 mm iD × 0.25 μm df; Restek). Helium was used as carrier gas (1 mL min^−1^, constant flow), with a temperature gradient from 70°C to 320°C over 18 min, preceded by an initial isothermal phase of 4 min and followed by 5 min at 320°C. Injections (1 μL) were made in splitless mode at 280°C. The injection sequence began with blank samples to define the baseline and check for contaminants with randomized sample injections in between. Mass spectra were acquired at 20 spectra/s over the 70–500 m/z range, and ionization was by EI at 70 eV.

### 2.3 Data Processing and Statistical Analysis

Peak integration (i.e., integrated area under a peak which reflects the relative amount of a compound), and identification were done on each sample individually using AMDIS with both in‐house and NIST reference libraries. Based on the detected peaks, we built a library containing all the features characterized by their retention times and mass spectral profiles (Table S1). When a feature could not be identified, it was assigned a random numerical label, used solely for reference purposes. The peak intensities were normalized by an internal ribitol standard. We also normalized peak intensity to sample size by dividing the intensities by the number of cells extracted. Quantitative analysis of the dataset (nine samples) was performed using MetaboAnalyst 5.0 (www.metaboanalyst.ca; Xia et al. 2009). First, the peak intensities dataset was filtered to lower low repeatability using the Relative Standard Deviation ($\mathrm{RSD}=\frac{\text{Standard deviation}}{\text{Mean}}$) with a threshold of 30% to ensure only consistent metabolites are retained. For comparisons between all three species, median normalization and autoscaling (mean centering and division by the standard deviation for each feature) were performed prior to statistical treatment. For comparisons with only *A. confertitesta* and *H. germanica* , a median normalization, followed by a log_10_ transformation and a mean centering was performed on a subset of 61 features present in both species. These pretreatments were chosen to ensure comparability and reduce variation across datasets. For the three species analysis, sample distribution was studied by analysis of variance (ANOVA)–principal component analysis (PCA) and metabolites were compared between species, highlighting species‐specific differences. Subsequently, hierarchical clustering and heatmap analysis were performed on the normalized data, applying Euclidean distance and Ward's linkage method by default. The differences in metabolome between *A. confertitesta* and *H. germanica* sampled in winter were analyzed through PCA and PLS‐DA. Subsequently, a Welch‐*t*‐test was conducted on features detected in both species to identify significant differences between the species, with multiple testing correction applied using the False Discovery Rate (FDR) method.

## 3 Results

### 3.1 Total Compounds Identified in the Three Foraminiferal Species

To investigate the foraminiferal metabolism, we profiled metabolites in polar extracts from three species of foraminifera using GC–MS. Across the nine samples studied, we detected 382 peaks with specific retention times and m/z combinations (hereafter referred to as features) and 30 identified molecules, which enabled us to construct a spectral library (Table S1). With our analysis, we identified 30 molecules within this library, including 15 amino acids and related compounds, nine sugars, five alpha hydroxycarboxylic acids, and one diamine (Table 1).

**TABLE 1:** List of identified compounds in *Ammonia confertitesta*, *Elphidium oceanense* and *Haynesina germanica* studied by GC–MS.

**Amino acids and derivatives**	**Sugars**
Alanine Arginine/citrulline/ornithine^a^ Asparagine Aspartate Glutamate Glutamine Glycine Histidine Ornithine/citrulline^a^	Cellobiose Fructose Galactose Glucose Glucose 6P Maltose Myo‐inositol Ribose Saccharose Trehalose
Phenylalanine	**Alpha hydroxycarboxylic acid**
Serine	Glycerate
Threonine	Lactate
Tryptophane	Malate
Tyrosine	Glycolate/glyoxylate^a^
Valine	**Diamine**
	Putrescine

^a^ Discrimination of these molecules is not possible in our protocol.

**FIGURE 2:**
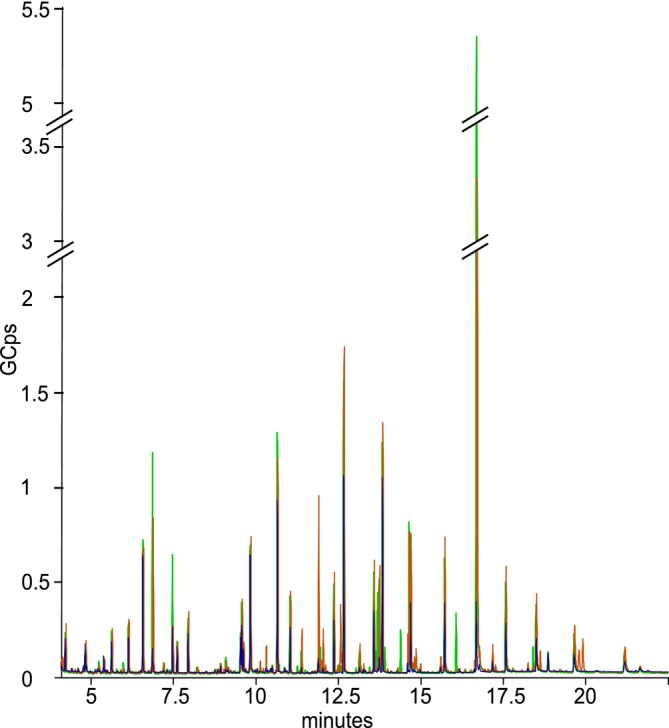
The overlay total ion chromatograms of the metabolites extracted from three foraminifera species by GC–MS analysis. The run time was 27 min, only data up to 22.5 are shown as no metabolites were detected after this point. The orange, blue, and green peaks respectively indicated a representative sample extracted from 200 specimens of *Ammonia confertitesta*, *Elphidium oceanense*, and *Haynesina germanica*.

For *A. confertitesta*, metabolite extracts from three different cell amounts (600, 300, and 200) were analyzed to investigate the effect of sample size on metabolite detection in foraminifera samples. The extract from 600 cells provided the greatest overall metabolome coverage, with 320 detected features (Table 2). For *H. germanica* , two sample sizes (200 and 300 cells) were analyzed, with a slightly higher number of features detected in the larger sample (99 vs. 82.5). The number of detected features did not appear to scale linearly with the cell number used for the metabolite extraction. For *A. confertitesta* and *H. germanica* , the reduction from 300 to 200 cells led to a decrease of less than one‐third in detected features (respectively 120 vs. 91 and 99 vs. 82.5). Taking into consideration the effort required to pick 100 cells more, especially when foraminiferal densities are low, the gain in detection does not justify the additional effort. Interspecific comparisons of chromatograms revealed differences in the presence and intensity of peaks, suggesting qualitative and quantitative variability (Figure 2). In this study, *A. confertitesta* and *H. germanica* were sampled during the same season, whereas *E. oceanense* was sampled in summer. The comparison between *A. confertitesta* and *H. germanica* may reflect an interspecific variation in metabolite composition and concentration. In contrast, the distinct chromatographic profile of *E. oceanense* may result from species‐specific metabolome and/or from seasonal biotic and abiotic environmental factors. Overall, extracts from *A. confertitesta* contained more features than those from *H. germanica* , and even more than those from *E. oceanense* (e.g., 91, 82.5, and 54.6 features respectively). In the chromatograms of *A. confertitesta* and *H. germanica* , the peak attributed to trehalose (Retention Time = 16.698 min) clearly dominated the chromatogram, while the intensities of other peaks were markedly lower (Figure 2). The major peak with the highest intensity corresponded to trehalose. In contrast, the chromatogram of *E. oceanense* shows a more uniform distribution, with no single dominant peak (Figure 2).

**TABLE 2:** Mean number of features detected in foraminifera polar extracts by GC–MS.

Species	Sampling date	Number of cell extracts	Number of pools analyzed	Number of features	Number of identified compounds
*Ammonia confertitesta*	December 16th 2020	600	1	320	30
	February 24th 2020	300	1	120	21
		200	1	91	17
*Haynesina germanica*	February 24th 2020	300	1	99	15
		200	2	82.5	15
*Elphidium oceanense*	August 24th 2020	200	3	54.6	9

### 3.2 Metabolites Profiling Comparison Between Species

**FIGURE 3:**
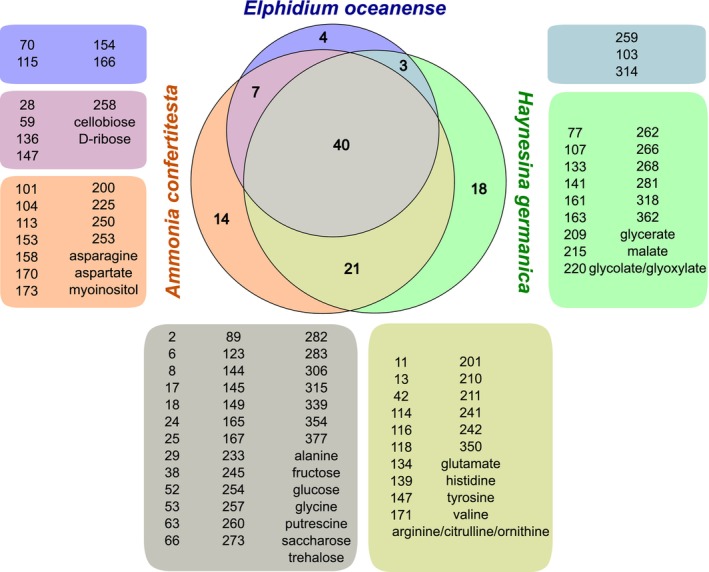
Euler diagram showing the number of metabolites shared among *Ammonia confertitesta*, *Elphidium oceanense*, and *Haynesina germanica*. The size of each circle and each intersection is proportional to the number of metabolites in each species. The list of features corresponding to each section is provided with a matching color. Feature numbering is random, and the GC–MS library (Table S1) associates these numbers with peak characteristics. Molecules whose names are separated by slashes are not discriminated by our GC–MS analysis protocol.

A total of 107 features were present in at least three replicates of one species and were selected for further analysis (Table S2). As it was not possible to collect all species in the same season, seasonal effects could not be investigated here; we focus on species comparisons. Given this limitation, statistical analysis between all three species should be considered exploratory. Forty features are common to all three species. Focusing on quality, some features were specific to one foraminifera species: 18 were detected only in *H. germanica* , 14 only in *A. confertitesta*, and four only in *E. oceanense* (Figure 3). With regard to the identified metabolites, asparagine, aspartate, and myoinositol were detected only in *A. confertitesta*, while malate, glycerate, and glycolate/glyoxylate were detected only in *H. germanica* . When focusing on foraminifera sampled in winter, *A. confertitesta* and *H. germanica* , a total of 61 features were shared. However, 21 features were unique to *A. confertitesta*, and another 21 features were unique to *H. germanica* .

**FIGURE 4:**
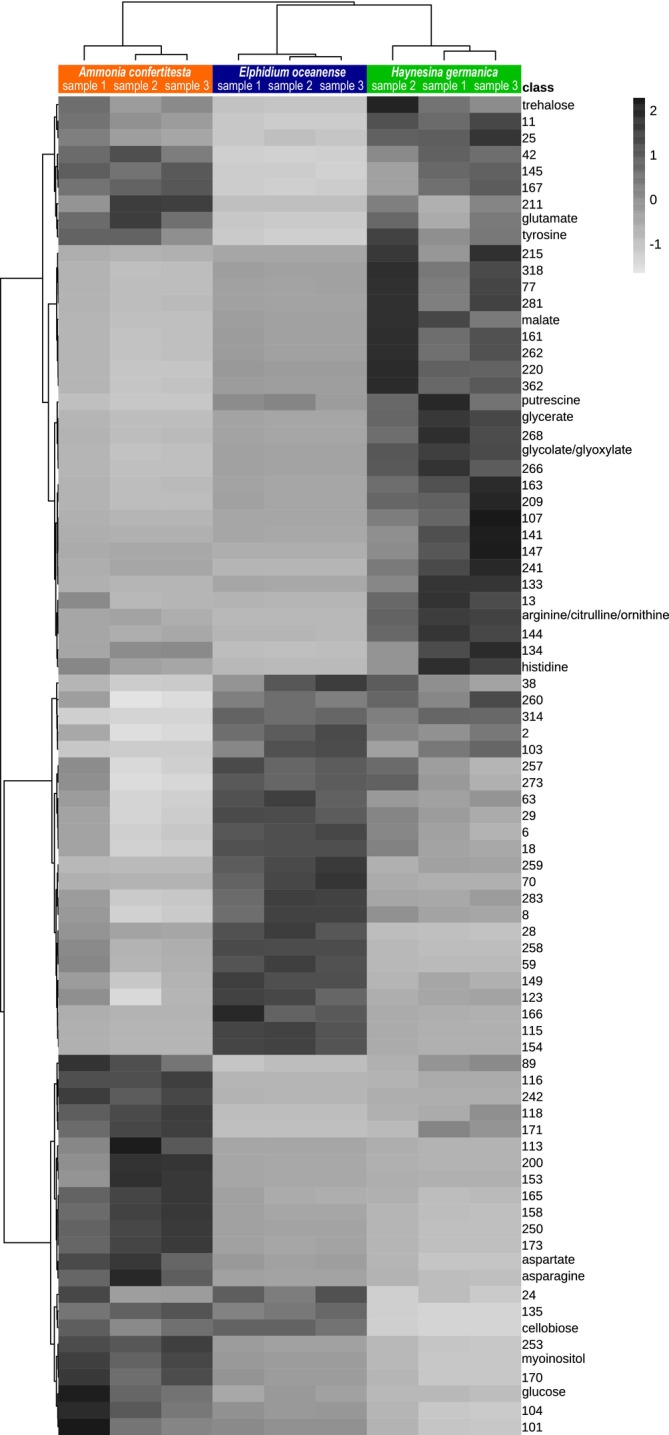
Heatmap matrix of the correlation between features and three foraminifera species. Only the 81 features showing significant differences in terms of peak intensity (i.e., area under the peak) by one‐way ANOVA test (*p* < 0.05) are shown (Table 3). The relative abundance of each feature, normalized across all samples, is represented by the gray intensity, with the scale bar indicating the range of values. The heatmap reveals specific features that are accumulated or not in certain species, illustrating the specificity of each species metabolic profile. The top dendrogram represents hierarchical clustering of the samples, clustering reflects similarity in overall metabolite composition. The left dendrogram represents hierarchical clustering of the features, with clusters indicating similar abundance patterns across samples. Feature numbering is random, and the GC–MS library (Table S1) associates these numbers with peak characteristics. Features whose names are separated by slashes are not discriminated by our GC–MS analysis protocol.

**FIGURE 5:**
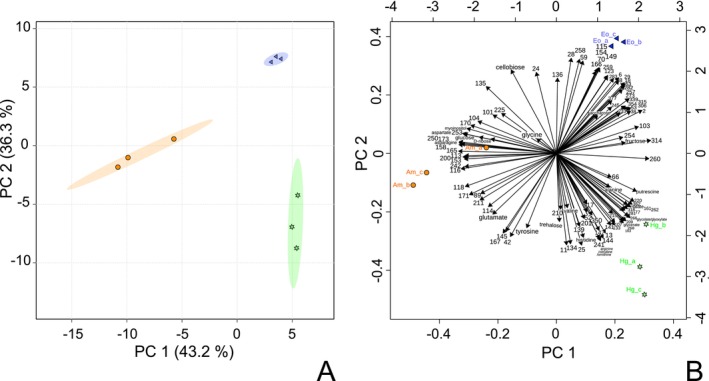
(A) Score plot showing principal components 1 and 2 of a Principal component analysis (PCA) of the foraminifera species based on GC–MS peak intensity (normalized by ribitol internal standard and number of cells). The circles represent *Ammonia confertitesta*, the triangles represent *Elphidium oceanense*, and the stars represent the *Haynesina germanica* samples. The 95% confidence ellipses around each group are shown, illustrating the spread of the samples within each species. Clustering of the samples represents similarity in metabolic profiles, while separation between samples suggests distinct metabolic profiles. (B) Principal component loading plot of compounds identified for the three foraminifera species that contributed most to the variance. The length and direction of the metabolite loading vector represent the contribution of each metabolite to species segregation. Feature numbering is random, and the GC–MS library (Table S1) associates these numbers with peak characteristics. Am_a refers to the extract derived from a sample of 200 *Ammonia confertitesta* cells collected on February 24th, 2020. Am_b refers to the extract derived from a sample of 300 *Ammonia confertitesta* cells collected on February 24th, 2020. Am_c refers to the extract derived from a sample of 600 *Ammonia confertitesta* cells collected on December 16th, 2020. Hg_a, Hg_b, and Hg_c refer to the extracts derived from a sample of respectively 200, 200, and 300 *Haynesina germanica* cells collected on February 24th, 2020. Eo_a, Eo_b, and Eo_c refer to the extracts derived from a sample of 200 *Elphidium oceanense* cells collected on August 24th, 2020.

To investigate differences in metabolite compositions among the three pools of foraminifera samples, *we performed* one‐way ANOVA using species as the only factor. Eighty‐one features studied show a significant difference between species with a *p*‐value threshold of 0.05 (Table 3). To visualize the changes in the metabolite patterns responsible for the discrimination among the three pools of samples, a heatmap and a hierarchical clustering were created by using the 81 features that were identified by the one‐way ANOVA test (Figure 4). Hierarchical clustering analysis revealed three distinct clusters, each corresponding to one of the three foraminifera species. Remarkably, the three *A. confertitesta* samples are clustered together, while samples collected in February do not cluster together regardless of species. This indicates that the sample‐specific metabolic signature of *A. confertitesta* may remain consistent between December and February. We point out the fact that the compounds from the second cluster from the top (Glycolate/glyoxylate, glycerate, malate, arginine/citrulline/ornithine, histidine, and putrescine for identified metabolites) are produced in higher quantities by the only kleptoplast species investigated ( *H. germanica* ) compared to the two other species. Aspartate, asparagine, myoinositol, and glucose have a higher accumulation in *A. confertitesta* than in the other two species. To classify samples and explore the metabolic variations, we performed a *Principal Component Analysis* (*PCA*). The PCA score plot clearly separates the sample groups *from each species*, *with no overlapping of their* 95% confidence region, which is supported by an accumulated variance contribution rate for the first two components of 79.5% (Figure 5A). The metabolite profiles of each pool of samples are significantly distinct. To study the features' contribution to sample separation, the PCA loading plot is presented in Figure 5B. The plot reveals that many features' vectors are in the same or opposite direction as the samples and have similar lengths, so this representation does not allow us to identify features that differentiate the species.

**TABLE 3:** Results of one‐way ANOVA performed on metabolite features of the three species.

Feature	*f* value	*p*	−log10(*p*)	FDR	Tukey's HSD		
2	22	1.69E‐03	2.77	0.004	Eo‐Am		
6	27	1.03E‐03	2.99	0.003	Eo‐Am	Hg‐Am	
8	16	3.78E‐03	2.42	0.007	Eo‐Am		Hg‐Eo
11	37	4.23E‐04	3.37	0.002	Eo‐Am		Hg‐Eo
13	21	2.06E‐03	2.69	0.005		Hg‐Am	Hg‐Eo
18	26	1.09E‐03	2.96	0.003	Eo‐Am	Hg‐Am	Hg‐Eo
24	8	2.15E‐02	1.67	0.033			Hg‐Eo
25	32	6.42E‐04	3.19	0.002	Eo‐Am		Hg‐Eo
28	109	1.90E‐05	4.72	0.000	Eo‐Am	Hg‐Am	Hg‐Eo
29	26	1.13E‐03	2.95	0.003	Eo‐Am	Hg‐Am	Hg‐Eo
38	6	3.56E‐02	1.45	0.048	Eo‐Am		Hg‐Eo
42	29	8.36E‐04	3.08	0.003	Eo‐Am		
59	37	4.36E‐04	3.36	0.002	Eo‐Am		Hg‐Eo
63	23	1.45E‐03	2.84	0.004	Eo‐Am		Hg‐Eo
70	54	1.50E‐04	3.82	0.001	Eo‐Am		Hg‐Eo
77	16	3.72E‐03	2.43	0.007			Hg‐Eo
89	19	2.72E‐03	2.57	0.006	Eo‐Am	Hg‐Am	Hg‐Eo
101	6	3.27E‐02	1.49	0.044		Hg‐Am	
103	14	5.89E‐03	2.23	0.010	Eo‐Am	Hg‐Am	
104	16	3.91E‐03	2.41	0.008	Eo‐Am	Hg‐Am	
107	8	1.84E‐02	1.73	0.029		Hg‐Am	
113	8	2.15E‐02	1.67	0.033	Eo‐Am	Hg‐Am	Hg‐Eo
115	282	1.16E‐06	5.93	0.000	Eo‐Am	Hg‐Am	
116	322	7.83E‐07	6.11	0.000	Eo‐Am		Hg‐Eo
118	52	1.62E‐04	3.79	0.001	Eo‐Am	Hg‐Am	
123	15	4.64E‐03	2.33	0.009	Eo‐Am	Hg‐Am	
133	12	8.28E‐03	2.08	0.014			Hg‐Eo
134	8	2.30E‐02	1.64	0.034		Hg‐Am	Hg‐Eo
135	77	5.33E‐05	4.27	0.001			Hg‐Eo
141	8	2.28E‐02	1.64	0.034		Hg‐Am	Hg‐Eo
144	43	2.83E‐04	3.55	0.001		Hg‐Am	Hg‐Eo
145	20	2.25E‐03	2.65	0.005	Eo‐Am	Hg‐Am	Hg‐Eo
147	7	2.48E‐02	1.61	0.036			Hg‐Eo
149	55	1.41E‐04	3.85	0.001	Eo‐Am	Hg‐Am	Hg‐Eo
153	8	2.27E‐02	1.64	0.034	Eo‐Am		Hg‐Eo
154	273	1.29E‐06	5.89	0.000	Eo‐Am	Hg‐Am	
158	39	3.55E‐04	3.45	0.002	Eo‐Am		Hg‐Eo
161	25	1.27E‐03	2.90	0.003		Hg‐Am	
163	26	1.13E‐03	2.95	0.003		Hg‐Am	Hg‐Eo
165	56	1.31E‐04	3.88	0.001	Eo‐Am	Hg‐Am	Hg‐Eo
166	29	8.19E‐04	3.09	0.003	Eo‐Am	Hg‐Am	
167	19	2.71E‐03	2.57	0.006	Eo‐Am		Hg‐Eo
170	36	4.46E‐04	3.35	0.002	Eo‐Am		Hg‐Eo
171	22	1.67E‐03	2.78	0.004	Eo‐Am	Hg‐Am	
173	53	1.50E‐04	3.82	0.001	Eo‐Am	Hg‐Am	
200	9	1.46E‐02	1.84	0.024	Eo‐Am	Hg‐Am	
209	22	1.72E‐03	2.77	0.004		Hg‐Am	
211	7	2.58E‐02	1.59	0.037	Eo‐Am	Hg‐Am	Hg‐Eo
215	7	2.68E‐02	1.57	0.038			
220	28	8.70E‐04	3.06	0.003		Hg‐Am	
241	16	3.98E‐03	2.40	0.008		Hg‐Am	Hg‐Eo
242	124	1.32E‐05	4.88	0.000	Eo‐Am	Hg‐Am	Hg‐Eo
250	63	9.25E‐05	4.03	0.001	Eo‐Am	Hg‐Am	
253	152	7.29E‐06	5.14	0.000	Eo‐Am	Hg‐Am	
257	7	3.09E‐02	1.51	0.043	Eo‐Am	Hg‐Am	Hg‐Eo
258	62	9.93E‐05	4.00	0.001	Eo‐Am		
259	89	3.49E‐05	4.46	0.000	Eo‐Am		Hg‐Eo
260	10	1.22E‐02	1.91	0.020	Eo‐Am		Hg‐Eo
262	24	1.37E‐03	2.86	0.004		Hg‐Am	
266	56	1.31E‐04	3.88	0.001		Hg‐Am	Hg‐Eo
268	29	7.98E‐04	3.10	0.003		Hg‐Am	Hg‐Eo
273	6	3.70E‐02	1.43	0.049	Eo‐Am	Hg‐Am	Hg‐Eo
281	15	4.77E‐03	2.32	0.009			
283	21	1.86E‐03	2.73	0.004	Eo‐Am	Hg‐Am	Hg‐Eo
314	123	1.36E‐05	4.87	0.000	Eo‐Am		Hg‐Eo
318	20	2.24E‐03	2.65	0.005		Hg‐Am	
362	28	9.28E‐04	3.03	0.003		Hg‐Am	Hg‐Eo
Arginine/citrulline/ornithine	70	6.88E‐05	4.16	0.001		Hg‐Am	Hg‐Eo
Asparagine	23	1.49E‐03	2.83	0.004	Eo‐Am	Hg‐Am	Hg‐Eo
Aspartate	48	2.00E‐04	3.70	0.001	Eo‐Am	Hg‐Am	
Cellobiose (2)	56	1.30E‐04	3.89	0.001		Hg‐Am	
Glucose	12	8.33E‐03	2.08	0.014	Eo‐Am	Hg‐Am	Hg‐Eo
Glutamate	14	5.46E‐03	2.26	0.010	Eo‐Am	Hg‐Am	
Glycerate	52	1.61E‐04	3.79	0.001			Hg‐Eo
Glycolate/glyoxylate	209	2.83E‐06	5.55	0.000	Eo‐Am	Hg‐Am	Hg‐Eo
Histidine	7	2.49E‐02	1.60	0.036		Hg‐Am	Hg‐Eo
Malate	19	2.49E‐03	2.60	0.005			Hg‐Eo
Myoinositol	67	7.91E‐05	4.10	0.001	Eo‐Am	Hg‐Am	Hg‐Eo
Putrescine	14	5.35E‐03	2.27	0.010		Hg‐Am	Hg‐Eo
Trehalose	6	3.21E‐02	1.49	0.044		Hg‐Am	
Tyrosine	13	7.19E‐03	2.14	0.012	Eo‐Am		Hg‐Eo

*Note:* For each feature, the *F*‐value, raw *p*‐value, its –log10 transformation, and the FDR‐adjusted *p*‐value are shown. Post hoc pairwise comparisons were conducted using Tukey's HSD test, and significant group differences are indicated in the last column.

Abbreviations: Am, *Ammonia confertitesta*; Eo, *Elphidium oceanense*; Hg, *Haynesina germanica*.

**FIGURE 6:**
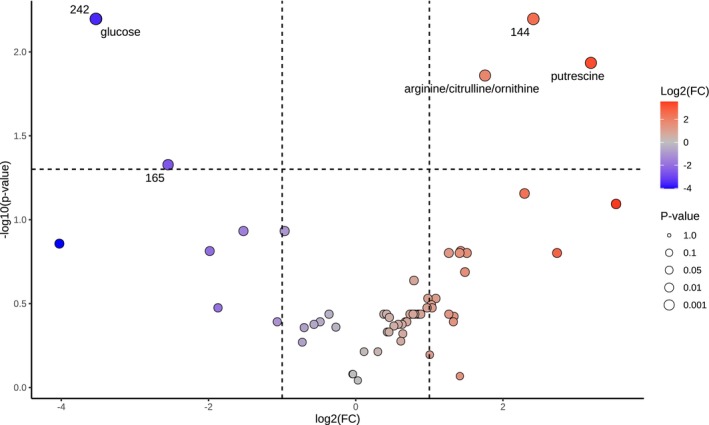
Volcano plot displaying the results of the Welch *t*‐test for metabolite abundance differences between *Haynesina germanica* and *Ammonia confertitesta*. The *x*‐axis represents the log2 fold change (logFC) between species, and the *y*‐axis shows the −log10 *p*‐value. Each point represents a metabolite. The size of the point represents the number of the *p*‐value. The threshold for significance is marked by the horizontal dashed line at −log10(*p*) = 1.3 (corresponding to *p* = 0.05) and the vertical dashed lines at logFC = ±1, indicating a twofold change in abundance. Those significantly different between species are labeled. Red points indicate metabolites more accumulated in *H. germanica* and blue points indicate metabolites more accumulated in *A. confertitesta*.

To exclude the seasonal parameter, we performed a second analysis focusing on *A. confertitesta* and *H. germanica* . A presence/absence comparison reveals 21 unique features in each species. To explore these differences further, a PCA was performed. Although a visual separation of species was observed, PERMANOVA does not support a statistically significant difference (*F* = 8.3315; *R* ^2^ = 0.67563; *p* = 0.1). To investigate differences in metabolite abundance, Welch's *t*‐test was conducted on the 61 metabolites shared between *H. germanica* and *A*. *confertitesta*. Six features were significantly different between *A*. *confertitesta* and *H. germanica* (FDR < 0.05, Welch's *t*‐test, Table 4, Figure 6). Among the significant features, three are identified: glucose, putrescine, and arginine/ornithine/citrulline. The presence/absence analysis and the Welch's test highlight a large portion of the features identified in the previous analysis. Figure 6 shows that putrescine and arginine/ornithine/citrulline are more accumulated while glucose is less accumulated in *H. germanica* compared to *A. confertitesta*. This limited number of significant features is consistent with the small sample size and the conservative correction applied, yet it still suggests the presence of robust species‐specific abundance differences.

**TABLE 4:** Results of the Welch *t*‐test for the comparison of features abundance between *H. germanica* and *A. confertitesta*.

Features	*t*.stat	*p*	‐log10(*p*)	FDR
144	−15.914	0.00018515	3.7325	0.0063564
Glucose	12.514	0.00025287	3.5971	0.0063564
242	19.365	0.00031261	3.505	0.0063564
Putrescine	−13.151	0.00076213	3.118	0.011622
Arginine/citrulline/ornithine	−8.8892	0.001133	2.9458	0.013823
165	6.0998	0.0046247	2.3349	0.047018

*Note:* For each feature, the *t*‐statistic (*t*.stat), *p*‐value, −log10(*p*) transformation, and FDR‐adjusted *p*‐value are shown.

## 4 Discussion

The metabolism of foraminiferal species from intertidal mudflats remains only partially explored, although notable studies have investigated the metabolic mechanisms underlying anoxia resistance in these species (Gomaa et al. 2021; Koho et al. 2018; LeKieffre et al. 2017; Orsi et al. 2020). Our study presents the first analysis of the foraminiferal metabolome acquired using an untargeted GC–MS approach on polar (methanol–water) extracts from three dominant species of intertidal mudflats. The results contribute to a more comprehensive understanding of the metabolism of intertidal foraminifera, suggesting a metabolism oriented towards adaptation to environmental constraints. Furthermore, the results revealed a link between species‐specific metabolic features and trophic strategies.

### 4.1 First Integrative Metabolomic Analysis of Foraminifera

Genomic, transcriptomic, proteomic, and metabolomic approaches to foraminifera remain underdeveloped, mainly due to technical and analytical challenges. These challenges include the complexity of the genome, the lack of reference data for assemblages and for gene identification, and the low biomass of smaller foraminiferal species. The study of an organism's global metabolome requires enough biomass, the amount of which depends on both the sensitivity of the analytical method used and the quantity and nature of metabolites produced by the organism in question. To our knowledge, before this study, no integrative analysis of the polar metabolome of foraminifera had been conducted. Previous studies have demonstrated the feasibility of GC–MS lipid analyses on smaller foraminiferal species, using between 100 and 300 cells per extraction (Nomaki et al. 2009; LeKieffre et al. 2017). At present, obtaining such numbers of foraminifera involves either manual collection, where individuals are picked individually using a brush under a stereomicroscope from sieved sediment samples, or reproduction under controlled laboratory conditions. Few species are successfully maintained in laboratory conditions (e.g., Kitazato and Bernhard 2014; Mojtahid et al. 2023; Dueñas‐Bohórquez et al. 2011; Nigam et al. 2008), and in vitro reproduction remains challenging to achieve, with few species‐specific protocols available (e.g., Barras et al. 2009; Lee et al. 1961, 1963; de Nooijer et al. 2014; Meilland et al. 2024). This is likely due to the variability in the ecological niches and species‐specific food requirements. Even though the three species studied here live in the same intertidal mudflat environment, seasonal population monitoring has shown that their reproductive events do not overlap (Choquel 2021), suggesting that environmental factors triggering reproduction likely differ between species. To the best of our knowledge, there is currently no protocol for the reproduction of *H. germanica* and *E. oceanense*. To study and compare these species, we therefore need to use manual harvesting, even though this is a time‐consuming method. Unfortunately, due to seasonal variations in the abundance of the three most dominant species in our study, it was not possible to collect all specimens of all species in the same season in 2020. The sampling constraint may introduce a potential seasonal bias that must be considered when interpreting interspecific metabolic differences. Each species was sampled during periods of high abundance, presumably under favorable conditions when the specific species fully occupied its ecological niche. Even though the samples were not collected at the same time, we can assume that the environmental conditions and constraints at the time of sampling were optimal for the development of the species sampled.

To circumvent the above‐described difficulties, we have developed an efficient protocol to extract metabolites from a reasonable size of in situ samples, revealing metabolic differences between three foraminiferal species of Bourgneuf Bay (Figure 4). This protocol will facilitate metabolomic studies for a wider range of foraminiferal species, avoiding culturing in the laboratory. We selected an ultrasound extraction using methanol–water (80:20; v/v) solvent baths from samples of different sizes (200, 300, and 600 cells). The challenge is to minimize the sample size to save time on picking samples and limit metabolic drift to obtain a snapshot of the metabolism of foraminifera in their living conditions. For *A. confertitesta*, 600 foraminifera allow for the detection of 320 features compared with 91 for 200 foraminifera (Table 2). A large sample enables an in‐depth study by lowering the detection threshold to better capture the diversity of molecules produced. Interestingly, for both *A. confertitesta* and *H. germanica* , the decrease in the number of features detected between the 300 and 200 cell samples is not proportional to the decrease in the size of the sample, remaining of the same order of scale. As mentioned in the materials and methods section, it takes two operators up to 12 h to collect 600 clean foraminifera from the sediment. Since reducing handling time minimizes metabolomic drift, using samples of 200 cells appears to be a reliable approach. Furthermore, PCA analysis, explaining 79.5% of the overall variance (Figure 5), and the Welch's *t*‐test on *H. germanica* and *A*. *confertitesta* (Figure 6) has shown that the detection threshold of a sample size of 200 cells is sufficient to observe interspecific differences on *A. confertitesta*, *H. germanica* , and *E. oceanense*.

**FIGURE 7:**
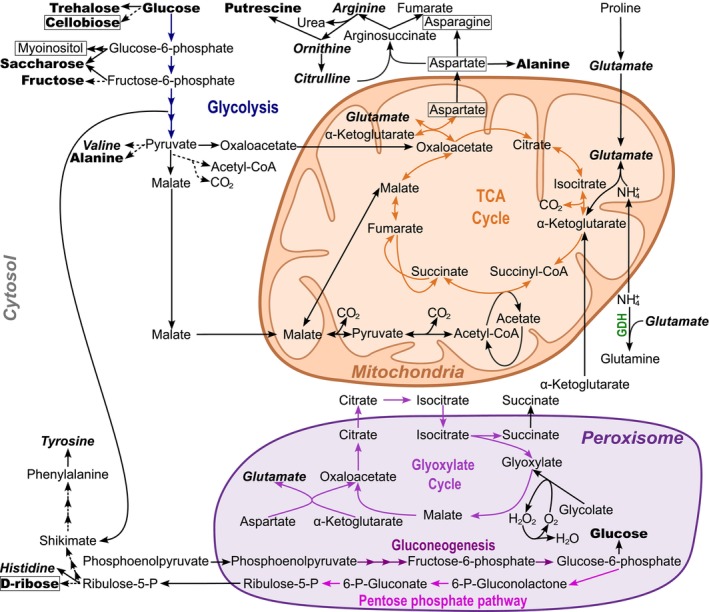
Schematic of the hypothetical metabolism of foraminifera. The metabolic pathway enzymes indicated by full arrows were found either in the genome or transcriptome of previously studied foraminifera (Glöckner et al. 2014; Gomaa et al. 2021; Orsi et al. 2020; Powers et al. 2022). The dotted arrows indicate potential metabolic pathways whose enzymes have not yet been found in previously studied foraminifera. Metabolites detected by GC–MS in *Haynesina germanica*, *Ammonia confertitesta*, and *Elphidium oceanense* are indicated in bold font. Metabolites detected by GC–MS only in *Ammonia confertitesta* and *Elphidium oceanense* are indicated in bold and framed. Metabolites detected by GC–MS common to *Ammonia confertitesta* and *Haynesina germanica* are indicated in bold italic font. Metabolites detected only in *Ammonia confertitesta* are framed. TCA cycle, tricarboxylic acid cycle.

**FIGURE 8:**
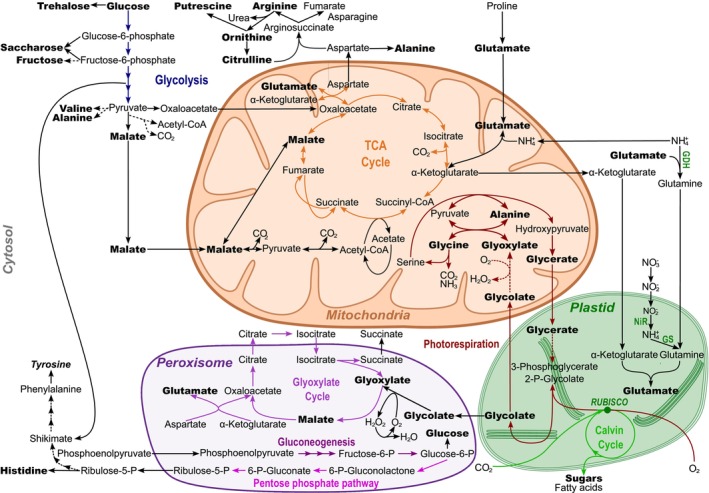
Schematic of the hypothetical metabolism of a kleptoplast foraminifera. The metabolic pathway enzymes indicated by full arrows were observed either in the genome or transcriptome of foraminifera in previous studies (Glöckner et al. 2014; Gomaa et al. 2021; Orsi et al. 2020; Powers et al. 2022). The metabolic pathways indicated in the kleptoplast correspond to those found in diatoms (Davis et al. 2017). Dotted arrows indicate potential metabolic pathways whose enzymes have not yet been identified in foraminifera and diatoms. Solid red arrows indicate reactions linked to photorespiration. Metabolites detected by our GC–MS analysis in *Haynesina germanica* are indicated in heavy font. TCA cycle, tricarboxylic acid cycle.

The protocol developed in this study revealed qualitative (Figure 3) and quantitative (Figures 4 and 6) differences in the metabolome of three intertidal foraminiferal species, demonstrating the potential of metabolomics for chemotaxonomical applications, even with a limited number of samples. We have established a library of 382 features and 30 identified molecules produced by *A. confertitesta*, *H. germanica* , and *E. oceanense* (Table S1). Our results complement the few previous studies on the chemical composition of foraminifera that focused mainly on test‐bound organic lining (Gooday et al. 2002; Grabenstatter et al. 2013; Ní Fhlaithearta et al. 2013; Paoloni et al. 2023; Suhr et al. 2008; Weiner and Erez 1984), on lipid composition and lipid consumption by foraminifera (Kharlamenko et al. 2017; Nomaki et al. 2009), or on shell amino acid ratios (Haugen et al. 1989; King and Hare 1972). Briefly, the panel of 30 identified metabolites in the present study proved to belong to four pathways: (1) amino acid metabolism; (2) carbohydrate metabolism (sugars); (3) photorespiratory glycolate metabolism (alpha hydroxycarboxylic acids); (4) polyamine metabolism (Table 1). In the published genome of *Reticulomyxa filosa* (Glöckner et al. 2014), we found all genes encoding enzymes necessary for core eukaryotic primary metabolic pathways such as glycolysis, gluconeogenesis, the tricarboxylic acid cycle, the pentose phosphate pathway, the polyamines, and the amino acid pathways. Additionally, genes coding for more specialized pathways, including the glyoxylate cycle, were present. Transcriptomic and metatranscriptomic analyses of benthic foraminifera (*Nonionella* sp., *Bolivina* sp., *Stainforthia* sp., and *Ammonia* sp.) have shown that most of the enzymes of the classical metabolic pathways are constitutively expressed (Gomaa et al. 2021; Orsi et al. 2020; Powers et al. 2022). Those data also revealed distinct transcription of metabolic pathways between species and oxygenation conditions (Gomaa et al. 2021; Powers et al. 2022). For example, *Bolivina argentea* expresses all the enzymes of the pentose phosphate pathway, while in *Nonionella stella*, the expression of the gene encoding 6‐phosphogluconolactonase (the enzyme catalyzing the conversion of 6‐P‐Gluconolactone to 6‐P‐Gluconate) has not been detected, illustrating species‐specific metabolite adaptations (Powers et al. 2022). Given the variability in gene expression both between species and across environmental conditions and considering that no transcriptomes are currently available for the three species studied here, we propose a hypothetical metabolism that incorporates all metabolic pathways previously reported in different foraminiferal species (Figure 7). This schematic represents the full published genetically encoded metabolic potential and highlights the metabolites accumulated by non‐kleptoplast foraminiferal species studied here (*A. confertitesta* and *E. oceanense*). For kleptoplast foraminifera, a recent metatranscriptomic study by Gomaa et al. (2025) on *N. stella* inhabiting euxinic, aphotic bathyal sediments revealed a kleptoplast‐related metabolism in this environment. However, up until now, no transcriptomic or metatranscriptomic investigations have been conducted on kleptoplast foraminifera in photic environments, even if studies have shown the photosynthetic activity and the formation of lipid droplets (Cesbron et al. 2017; Jauffrais et al. 2016; Jesus et al. 2022; LeKieffre et al. 2018; Lopez 1979). We propose a hypothetical metabolism of kleptoplastic foraminiferal species (Figure 8) based on all pathways observed in the genomic and transcriptomic studies of foraminifera and metabolic pathways of diatoms (Davis et al. 2017).

### 4.2 Intertidal Foraminifera Metabolome Reveals Potential Adaptation Mechanisms to Abiotic Constraints

**FIGURE 9:**
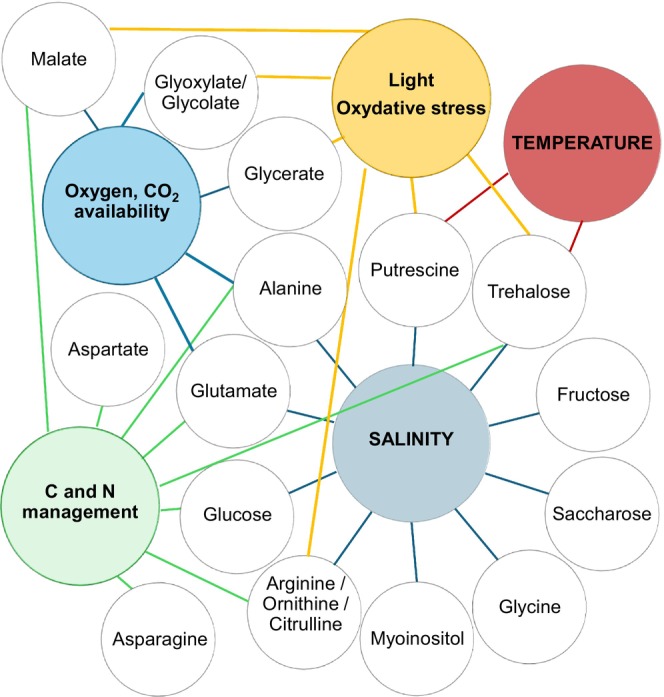
Schematic links between accumulated metabolites and abiotic constraints in intertidal foraminifera. Metabolites detected by GC–MS in *Ammonia confertitesta*, *Haynesina germanica* and *Elphidium oceanense* are represented as white circles. Colored circles correspond to main abiotic constraints occurring in intertidal environments. Links between metabolites and environmental factors are inferred from literature‐based evidence and are hypothetical in this study.

Many of the identified accumulated molecules are known to play a role in abiotic stress response and/or adaptation mechanisms to environmental constraints (Figure 9). In this study, foraminifera were sampled under conditions presumed to be optimal, as indicated by their high density in the sediment. In this context, the accumulation of such compounds could be interpreted as a long‐term adaptive strategy that enables these organisms to deal with the naturally fluctuating and constraining conditions of intertidal environments. Identifying stressful conditions for intertidal foraminifera is complex, as they are constantly exposed to strong abiotic fluctuations. Moreover, species‐specific physiological adaptations to these variations have not been sufficiently explored for all species. Most of the 30 molecules identified are known as osmolytes, and as such, they are osmotically active (Harding et al. 2016; Nikitashina et al. 2022). The increase in osmotic pressure is one of two strategies well‐known in protists to prevent salt from penetrating the cell, called the “salt‐out” strategy (Czech and Bremer 2018; Harding et al. 2016). This accumulation of osmolytes could help foraminifera to deal with long‐term exposure to marine salinity (ca. 33 psu in Bourgneuf Bay; Dutertre et al. 2010) and increased or decreased salinity in the first centimeters of the sediment during low tide caused by evaporation or rain.

Comparison of the presence/absence of features across the three species revealed 40 (36.7%) common features, including seven identified metabolites (i.e., alanine, glycine, fructose, glucose, saccharose, trehalose, and putrescine). Among those seven metabolites, two are particularly noteworthy. The biosynthesis of putrescine is known to be responsive to temperature, osmotic, and oxidative stress in many organisms, such as plants or fungi. Intertidal species are particularly exposed to oxidant stress, particularly due to UV exposure at low tide (de Mora et al. 2005), as well as variation in oxygen concentration, salinity (Choquel 2021), and/or pH. As a protectant molecule, putrescine has been shown to contribute to cellular pH regulation, stabilization of membrane lipids and proteins, and accumulation as an osmo‐protectant and antioxidant (Liu et al. 2007; Valdés‐Santiago and Ruiz‐Herrera 2014). Interestingly, Yasumoto et al. (2014) demonstrated that putrescine may also have a role in regulating pH during calcification by bacteria. These findings suggest that putrescine likely plays a key role in both stress response and the biomineralization process in various foraminifera species. Trehalose appeared as the dominant compound of the *A. confertitesta* and *H. germanica* metabolome (Figures 2 and 4), accumulating probably as a carbon storage molecule. This molecule is also well known for its osmo‐ and thermo‐protective properties similar to putrescine, by protecting proteins and membranes from oxidation (Elbein et al. 2003). The high content of trehalose in these two species may contribute to the widespread presence of foraminifera and their tolerance of abiotic challenging conditions, as can be found in intertidal mudflats (Dutertre et al. 2010; Guarini et al. 1997; Soletchnik et al. 2007). *A. confertitesta* and *H. germanica* shared five more identified molecules, i.e., glutamate, histidine, tyrosine, valine, and arginine/citrulline/ornithine (Figure 3). Among these, glutamate and arginine/citrulline/ornithine are precursors of polyamines such as putrescine (Figure 8). This suggests that foraminifera could maintain protective polyamine levels. Glutamate could also play a role in nitrogen transfer and storage. In *H. germanica* , some evidence of GS‐GOGAT activity has been observed (LeKieffre et al. 2018). A high concentration of ${\mathrm{NH}}_{4}^{+}$ in the environment could lead to the transient glutamate accumulation. Glutamate, along with alanine, may accumulate as part of hypoxia‐adapted metabolism, as observed in plants where it plays a central role in nitrogen and carbon mobilization under low oxygen conditions (Diab and Limami 2016). Glutamate donates amino groups for alanine synthesis by Alanine Aminotransferase (AlaAT) during hypoxia and is regenerated via GOGAT. Upon reoxygenation, glutamate is recovered from alanine and deaminated by GDH to supply 2‐oxoglutarate for the TCA cycle and regenerate NADH. Although genes encoding for some AlaAT, GDH, and GOGAT were found in the foraminiferal genome and GDH expression has been observed under anoxic conditions (Bird et al. 2020; Orsi et al. 2020). However, direct evidence for the operation of this glutamate‐alanine cycle in foraminifera remains to be demonstrated. Furthermore, myoinositol, asparagine, and aspartate are only detected in *A. confertitesta*. Myoinositol is strongly accumulated, and this molecule is known to be a salt protectant in diatom and protist cells (Harding et al. 2016; Nikitashina et al. 2022). This accumulation further reinforces a metabolism focused on resistance to abiotic constraints, contributing to the species' widespread distribution. The accumulation of asparagine and its precursor, aspartate, in *A. confertitesta* may indicate a potential strategy for nitrogen and carbon management, as has been observed in plants where asparagine aids in transporting and storing these elements (Lea et al. 2007). Further investigation into nitrogen assimilation in *A. confertitesta* could shed light on the potential role of asparagine.

In general, *H. germanica* and *A. confertitesta* exhibited a higher metabolite diversity and a greater number of metabolites potentially involved in adaptation to environmental constraints compared to *E. oceanense* (Figure 3). It is important to note that *E. oceanense* was sampled in a different season. As a result, it is not possible to distinguish species‐specific from seasonal effects, and caution is needed when interpreting interspecific differences. The reduced diversity of stress‐related metabolites observed in *E. oceanense*, including lower levels of trehalose, may partly explain its generally lower abundance in mudflats compared to *H. germanica* and *A. confertitesta* (Debenay et al. 2000, 2006; Pavard et al. 2023). *E. oceanense* is more likely to be found in estuarine areas under marine influence (Debenay et al. 2000, 2006), which likely results in less complex and variable environmental conditions. This ecological niche may reduce the selective pressure for extensive metabolic adaptations to fluctuating stressors compared to the other two species. A Welch *t*‐test on *A. confertitesta* and *H. germanica* sampled in the same season confirmed some significant differences in both abundance and presence or absence of metabolites. Six metabolites showed significant differences in the level of accumulation. Among the three features more accumulated in *A. confertitesta* than in *H. germanica* , only glucose is identified. This accumulation could indicate that *A. confertitesta* prioritizes glucose accumulation for energy storage, while *H. germanica* prioritizes other pathways. To confirm whether the observed differences are driven by species and/or seasonal variation, a comparative analysis with all species sampled during the same season, or after incubation under controlled conditions, would be necessary. Furthermore, a comparison of metabolome osmolyte compositions between intertidal and fully marine foraminiferal species could provide further insights into how osmolytes adapt to different environmental constraints, particularly salinity fluctuations.

### 4.3 Metabolism of *Haynesina germanica* Linked to Kleptoplasty

For *H. germanica* , previous studies have demonstrated photosynthetic activity and inorganic carbon assimilation in light conditions (Cesbron et al. 2017; Jauffrais et al. 2016; Jesus et al. 2022; LeKieffre et al. 2018; Lopez 1979). Interestingly, no starch accumulation was observed in the kleptoplast of *H. germanica* (Jauffrais et al. 2018), nor was any accumulation of photosynthates detected in the kleptoplasts themselves (LeKieffre et al. 2018). LeKieffre et al. (2018) have demonstrated the active role of chloroplasts in carbon assimilation, with photo‐assimilates accumulating mainly in lipid droplets as well as in electron‐opaque bodies and fibrillar vesicles. Furthermore, they also hypothesized multiple nitrogen assimilation pathways linked to kleptoplasty. However, for now, the transfer of photosynthates to the host cell and their implication in *H. germanica* metabolism remains poorly understood.

Here, we highlight other types of interaction between kleptoplast activity and the metabolism of the foraminifera. A total of 18 features were detected uniquely in *H. germanica* , three of which were identified as glycerate, malate, and glycolate/glyoxylate (Figure 3). Accumulation of these molecules has been described in diatoms and linked to photosynthesis, glycolate cycle, and photorespiration pathways (Davis et al. 2017; Florian et al. 2013; Kroth et al. 2008). The accumulation of glycerate could result from the first step of carbon fixation (Smith et al. 2016) and is consistent with the photosynthetic activity demonstrated in *H. germanica* (Cesbron et al. 2017; LeKieffre et al. 2018; Lopez 1979). Malate accumulation could be linked to a C4‐like pathway as observed in diatoms, which concentrates CO_2_ near the Rubisco to facilitate the photosynthetic reaction (Obata et al. 2013). Malate is also known to be a mobile form of inorganic carbon storage in plants (Zell et al. 2010), and this may also be its role in *H. germanica* . Lastly, both glyoxylate/glycolate and glycerate accumulation can be linked to peroxisomal reactions through the glyoxylate cycle, suggesting the occurrence of photorespiration activity (Figure 8; Davis et al. 2017). If photorespiration is known to be commonly activated in diatoms in relation to photosystem protection, this is the first time its occurrence has been proposed in foraminifera. Photorespiration is also involved in abiotic constraints. In high light or high O_2_ conditions, the oxygenase activity of Rubisco is enhanced, leading to increased production of glycine. Subsequently, glycine is converted to serine in the mitochondria with the release of nitrogen (NH_3_), NADH, and CO_2_ for photosynthesis (Figure 8; Voss et al. 2013). Reactions associated with photorespiration contributed to redox homeostasis by consuming ATP, NAD(P)H, and reduced ferredoxin: NH_3_ is reassimilated into glutamine using ATP, hydroxypyruvate conversion to glycerate consumes NAD(P)H, and glutamine conversion to glutamate uses reduced ferredoxin (Voss et al. 2013). Therefore, photorespiration played an important role in the dissipation of excess light energy and in pH homeostasis. In *H. germanica* , a functional xanthophyll cycle has been observed, which shares similar properties (Jauffrais et al. 2017). Together, these two mechanisms could play a key role in the maintenance of kleptoplasts in an environment where they are exposed to high light intensity.

A comparison of the abundance of common metabolites between *A. confertitesta* and *H. germanica* in winter revealed three molecules that were significantly more accumulated in *H. germanica*. Among them, arginine/ornithine/citrulline and putrescine were identified (Table 4; Figure 6). Arginine/ornithine/citrulline are key precursors for polyamines, such as putrescine, so their co‐accumulation with putrescine could reflect a strong activation of the polyamine biosynthetic pathway. As discussed previously, putrescine plays a crucial role in protecting cells against various abiotic stresses, notably oxidative stress. Arginine/ornithine/citrulline, which are part of the urea cycle, may also play a role in nitrogen recycling. In diatoms under high light and nitrogen‐rich conditions, a concomitant activation of the urea cycle and photorespiration has been reported (Bender et al. 2012). Although direct evidence for photorespiration and the urea cycle in *H. germanica* is still lacking, the metabolic signature we observed (i.e., the accumulation of malate, glycolate/glyoxylate, and glycerate) could indicate integration of nitrogen recycling and carbon metabolism, comparable to diatoms. While kleptoplasty through photosynthesis provides an additional carbon source, it also creates oxidative stress; the metabolite signature of *H. germanica* may reflect a physiological adjustment to kleptoplastic activity and protection.

The molecules accumulated in *H. germanica* could be linked to two newly described phenomena in kleptoplastic foraminiferal cells, i.e., photorespiration and C4‐like pathways. Those pathways need to be studied further to validate their activity in kleptoplastic cells. We could potentially investigate the presence and the light‐dependent expression of the gene coding for glycine decarboxylase in order to confirm the activation of photorespiration, as has been done for diatoms in the past (Schnitzler Parker et al. 2004). To prove that accumulation of malate can be used in a C4‐type mechanism in kleptoplasts, we could study one of the key enzymes, phosphoenolpyruvate carboxylase, whose inhibition significantly reduces photosynthetic efficiency (Reinfelder et al. 2004). The mechanisms underlying chloroplast sequestration in foraminiferal cells and their role need to be further explored to confirm the importance of kleptoplasty‐related metabolism and the nature of the reactions in *H. germanica* .

## 5 Conclusion

This study reveals a significant accumulation of numerous osmolytes and stress‐response‐related molecules in the global metabolome of intertidal foraminifera. Noticeably, the massive storage of sugar in the form of trehalose in the cells of *A. confertitesta* and *H. germanica* could be one of the keys to the widespread occurrence of these species, by providing them with osmoprotection and thermotolerance well suited to the environmental conditions in the intertidal zone. For *H. germanica* , the accumulation of glycerate, malate, and glycolate/glyoxylate suggests a potential C4‐like photosynthesis activity coupled with photorespiration. While photorespiration has not yet been observed in foraminifera, it has been documented in diatoms. Such a mechanism could help protect the kleptoplast from stress‐induced damage while allowing it to retain function within the cell under fluctuation in environmental conditions such as temperature, light, and CO_2_ availability. Furthermore, glycerate, malate, and glycolate/glyoxylate may become potential biomarkers to detect if kleptoplasts in *H. germanica* are active when exposed to specific environmental constraints.

However, to confirm that molecules accumulated in foraminifera contribute to the species' tolerance to extreme or stressful conditions, further experiments are needed. Specifically, comparative metabolome analyses under different abiotic controlled conditions (e.g., temperature, salinity, oxygen availability) would help to first determine the threshold at which abiotic factors become stressors and secondly to validate the molecular mechanism of adaptation. A comparison of metabolome osmolyte compositions between intertidal and fully marine foraminiferal species could provide further insights into how osmolytes adapt to different environmental constraints, particularly salinity fluctuations. Future investigations could assess whether similar metabolites' accumulation is observed in other kleptoplastic foraminifera from the photic zone, as such metabolome patterns might serve as potential biomarkers of active kleptoplasty. Further investigation, particularly into genes encoding enzymes related to photorespiration required for validation of this mechanism and a more comprehensive understanding. Additionally, this untargeted approach on in situ samples could serve as a powerful tool to investigate spatial and seasonal variability in foraminiferal metabolomes. This could improve our understanding of foraminiferal trophic strategies, fitness, and reproductive and population dynamics.

## Supporting information


**Table S1:** GC–MS Spectral library for metabolites from intertidal benthic foraminifera.

**Table S2:** Normalized features intensities for three foraminiferal species: *Elphidium oceanense*, *Haynesina germanica*, and *Ammonia confertitesta*. Each sample (a, b, c) corresponds to a pool of specimens, whose sizes are detailed in Table 2. The values represent the area under the curve for each of the 107 features detected in at least three replicates of a given species. The areas under the curve were divided by the number of individuals in each pool and normalized to the internal standard (ribitol).

## Data Availability

The data that support the findings of this study are available from the corresponding author upon reasonable request.
