# Supplementary material for: Metabolic Adaptations of Benthic Forams: Foraminiferal Species Adaptations to Intertidal Mudflat Assessed by a Metabolic Approach
Source: J Eukaryot Microbiol. 2025 Nov 11;72(6):e70051. doi: 10.1111/jeu.70051 (PMC12605782; doi:10.1111/jeu.70051)
Supplement: Supplementary file 1 — Table S1: GC–MS Spectral library for metabolites from intertidal benthic foraminifera. [file JEU-72-e70051-s001.docx]

Table S1: GC-MS Spectral library for metabolites from intertidal benthic foraminifera.

This table presents the GC-MS spectral library created for the identification of metabolites. It includes the name of each metabolite (NAME), retention index (RI), retention time (RT), the number of detected peaks (NUM.PEAKS), and the corresponding m/z values with peak intensities (PEAKS). This library was developed based on metabolites detected in *Ammonia confertitesta, Haynesina germanica* and *Elphidium oceanense*.

| **NAME** | **RI** | **RT** | **NUM.PEAKS** | **PEAKS** |
| --- | --- | --- | --- | --- |
| 1 | 1001,4 | 4,3567 | 86 | (50 42) (51 30) (52 43) (53 25) (54 11) (55 108) (56 103) (57 508) (60 89) (61 38) (62 16) (63 53) (64 12) (69 355) (70 66) (71 175) (72 43) (73 737) (74 67) (75 215) (76 51) (77 999) (78 146) (79 70) (80 7) (81 14) (83 10) (84 54) (85 84) (86 10) (87 6) (88 42) (89 33) (90 63) (91 41) (92 8) (93 10) (94 6) (95 7) (96 6) (97 24) (98 18) (99 25) (100 53) (102 11) (103 163) (104 27) (105 11) (107 35) (108 7) (109 6) (110 45) (111 8) (112 26) (113 21) (114 6) (115 9) (116 16) (118 40) (120 85) (121 11) (122 4) (130 23) (132 49) (134 629) (135 62) (136 27) (137 4) (138 3) (140 101) (141 7) (143 77) (144 9) (145 7) (156 2) (184 540) (185 59) (186 22) (198 7) (199 2) (214 248) (215 29) (216 11) (229 79) (230 10) (231 3) |
| 2 | 1043,2 | 4,7443 | 50 | (54 6) (66 21) (67 13) (68 9) (69 17) (78 118) (80 8) (82 7) (83 21) (84 12) (85 5) (86 11) (87 4) (92 9) (96 5) (97 21) (98 11) (100 7) (122 82) (133 5) (136 34) (137 16) (138 9) (143 3) (146 6) (152 999) (153 129) (154 34) (155 5) (157 3) (159 4) (166 136) (167 86) (168 14) (169 3) (173 2) (177 2) (189 4) (191 12) (192 3) (193 3) (207 92) (208 18) (209 11) (210 1) (247 2) (279 2) (295 14) (296 5) (297 3) |
| 3 | 1050,1 | 4,8084 | 5 | (72 999) (102 113) (147 32) (174 25) (218 8) |
| 4 | 1060,9 | 4,9091 | 44 | (55 46) (56 32) (57 174) (59 122) (60 12) (61 28) (66 143) (67 21) (70 50) (71 133) (72 20) (73 379) (74 31) (75 38) (81 47) (84 30) (85 119) (87 25) (99 23) (101 47) (102 8) (103 55) (105 14) (115 320) (116 37) (127 17) (130 270) (131 78) (132 23) (133 88) (134 13) (146 45) (147 999) (148 158) (149 84) (150 7) (163 9) (177 75) (178 13) (205 29) (206 8) (234 78) (235 18) (236 10) |
| 5 | 1119,5 | 5,4516 | 28 | (59 175) (66 112) (72 50) (73 423) (74 60) (75 72) (86 81) (101 20) (102 22) (113 30) (114 20) (119 120) (120 32) (130 83) (131 69) (133 274) (135 28) (146 140) (147 999) (148 136) (149 71) (160 19) (181 14) (204 171) (205 29) (206 13) (219 9) (249 65) |
| 6 | 1122,4 | 5,4794 | 59 | (50 82) (51 56) (52 68) (54 19) (55 80) (56 26) (57 20) (58 56) (59 204) (60 20) (61 16) (66 16) (69 34) (70 24) (71 24) (72 95) (73 999) (74 82) (75 62) (78 34) (83 38) (85 18) (86 116) (87 45) (88 25) (99 10) (100 99) (101 14) (109 7) (113 26) (114 33) (115 26) (116 41) (119 312) (120 42) (124 4) (130 144) (131 41) (132 28) (133 476) (134 78) (135 36) (145 6) (146 257) (147 180) (148 28) (158 17) (159 5) (160 11) (173 14) (174 9) (177 5) (188 9) (190 7) (206 14) (249 126) (250 21) (251 13) (333 3) |
| 7 | 1131 | 5,5594 | 27 | (57 15) (59 65) (61 15) (66 49) (72 27) (73 599) (74 54) (75 68) (86 20) (87 19) (94 9) (101 10) (102 999) (103 122) (104 43) (130 8) (131 20) (133 16) (147 330) (148 50) (149 26) (176 54) (177 10) (178 14) (204 65) (205 10) (206 5) |
| 8 | 1153,4 | 5,7665 | 92 | (52 4) (53 3) (54 2) (55 11) (56 10) (57 14) (58 43) (59 187) (60 17) (61 18) (62 1) (66 11) (67 2) (70 50) (71 11) (72 130) (73 999) (74 98) (75 105) (76 6) (77 2) (84 2) (85 4) (86 110) (87 17) (88 27) (89 82) (90 7) (91 4) (99 4) (100 89) (101 11) (102 22) (103 78) (104 9) (105 10) (106 1) (107 1) (113 4) (114 4) (115 16) (116 10) (117 24) (118 9) (119 22) (120 2) (121 1) (128 1) (129 2) (130 12) (131 73) (132 28) (133 327) (134 47) (135 25) (136 2) (144 4) (145 2) (146 89) (147 833) (148 134) (149 66) (150 6) (151 1) (158 4) (159 1) (160 61) (161 9) (162 19) (163 13) (164 2) (165 1) (174 25) (175 5) (176 3) (177 3) (188 2) (189 1) (190 29) (191 5) (192 2) (205 28) (206 5) (207 2) (220 100) (221 18) (222 8) (223 1) (235 79) (236 15) (237 7) (238 1) |
| 9 | 1164,1 | 5,8677 | 40 | (51 96) (57 72) (59 508) (60 38) (70 34) (71 118) (72 56) (73 999) (85 31) (89 190) (90 23) (93 85) (99 21) (100 541) (101 102) (102 46) (103 48) (105 21) (116 110) (120 24) (129 19) (130 46) (131 49) (132 21) (133 82) (134 20) (142 75) (143 18) (147 340) (148 48) (149 37) (157 14) (159 10) (160 16) (174 517) (175 87) (176 50) (205 64) (206 13) (235 11) |
| 10 | 1167,2 | 5,8947 | 31 | (55 19) (56 25) (57 17) (61 33) (69 28) (70 33) (74 50) (75 258) (76 23) (77 14) (86 999) (87 68) (102 9) (103 39) (104 10) (128 8) (129 16) (142 7) (143 36) (144 8) (146 41) (170 30) (171 18) (185 7) (188 44) (189 7) (190 4) (203 9) (267 9) (355 7) (356 2) |
| 11 | 1174,9 | 5,9661 | 103 | (55 15) (57 11) (59 51) (60 7) (61 15) (69 11) (71 6) (72 17) (73 999) (74 87) (75 170) (76 11) (77 8) (85 10) (87 6) (95 9) (99 8) (101 6) (103 6) (115 5) (116 6) (117 77) (118 7) (119 12) (128 9) (129 21) (131 38) (132 15) (133 38) (134 7) (135 4) (136 2) (143 6) (145 10) (147 673) (148 102) (149 56) (150 6) (152 45) (153 7) (159 5) (163 8) (165 7) (167 10) (175 4) (177 15) (178 4) (179 12) (187 57) (188 7) (189 16) (190 3) (191 54) (192 10) (193 29) (194 5) (195 3) (203 9) (204 2) (205 24) (206 6) (207 90) (208 18) (209 11) (219 2) (221 6) (235 9) (236 2) (237 2) (247 5) (248 2) (249 95) (250 24) (251 28) (252 6) (253 3) (255 4) (263 2) (265 99) (266 27) (267 21) (268 5) (269 2) (279 4) (280 2) (281 672) (282 188) (283 119) (284 24) (285 8) (286 1) (309 1) (321 1) (324 2) (337 1) (353 4) (354 2) (369 145) (370 55) (371 34) (372 8) (373 2) (374 1) |
| 12 | 1179,9 | 6,0122 | 16 | (54 9) (55 27) (56 5) (57 5) (67 17) (68 8) (69 8) (80 5) (82 78) (83 13) (84 999) (85 60) (186 17) (187 8) (188 2) (201 2) |
| 13 | 1188,8 | 6,0955 | 49 | (55 372) (56 62) (57 41) (58 51) (59 100) (60 37) (61 377) (62 13) (63 19) (69 59) (70 14) (71 10) (72 31) (73 793) (74 999) (75 783) (76 95) (77 42) (85 25) (86 501) (87 55) (88 16) (90 6) (91 19) (101 19) (102 40) (103 32) (104 5) (105 62) (106 8) (107 10) (113 11) (115 5) (116 12) (129 483) (130 79) (131 55) (145 5) (146 31) (170 14) (171 6) (177 134) (178 17) (179 12) (188 17) (192 233) (193 32) (194 21) (267 6) |
| 14 | 1218,3 | 6,3606 | 36 | (56 25) (58 90) (59 21) (60 24) (63 25) (69 268) (72 43) (73 999) (74 87) (75 181) (76 27) (77 259) (78 36) (88 8) (91 24) (108 15) (110 286) (116 25) (118 17) (127 26) (130 24) (134 293) (135 28) (136 59) (143 9) (184 251) (185 25) (195 7) (198 6) (228 483) (229 58) (230 20) (261 4) (277 9) (285 25) (286 5) |
| 15 | 1232,9 | 6,4888 | 30 | (54 9) (55 36) (56 10) (58 28) (59 120) (60 22) (61 31) (72 18) (74 32) (75 139) (76 13) (86 31) (87 12) (88 4) (89 218) (90 15) (91 10) (100 8) (101 219) (102 20) (103 15) (116 45) (117 999) (118 91) (119 39) (128 5) (140 5) (159 31) (160 4) (171 9) |
| 16 | 1233,4 | 6,4933 | 38 | (54 25) (55 94) (59 207) (60 46) (61 59) (67 19) (68 12) (69 38) (72 32) (73 999) (74 63) (75 341) (76 29) (81 11) (82 26) (86 76) (87 20) (89 362) (90 23) (91 16) (96 10) (99 17) (101 356) (102 33) (114 8) (118 140) (119 61) (128 11) (129 7) (140 8) (142 7) (143 16) (159 49) (163 6) (170 85) (171 22) (217 34) (218 6) |
| 17 | 1243,3 | 6,5811 | 44 | (59 64) (60 21) (61 16) (64 11) (66 90) (72 38) (73 632) (74 125) (75 92) (87 55) (88 9) (99 119) (100 46) (101 21) (102 13) (114 13) (115 17) (116 12) (129 39) (130 61) (131 77) (132 44) (133 37) (146 68) (147 999) (148 159) (149 79) (150 8) (157 16) (171 200) (172 34) (173 60) (186 6) (187 5) (189 451) (190 76) (191 35) (202 10) (204 9) (205 9) (221 12) (231 5) (246 7) (247 4) |
| 18 | 1250,2 | 6,642 | 43 | (54 3) (55 7) (58 22) (59 75) (60 8) (61 23) (62 2) (66 86) (67 12) (72 25) (73 999) (74 87) (75 119) (76 6) (77 7) (85 14) (87 20) (88 31) (101 88) (102 11) (103 108) (104 9) (105 5) (115 9) (116 201) (117 403) (118 49) (119 24) (120 3) (125 1) (131 6) (133 33) (134 3) (135 2) (145 15) (147 107) (148 15) (149 8) (191 21) (192 4) (193 2) (207 3) (235 4) |
| 19 | 1256,7 | 6,6987 | 17 | (59 56) (60 24) (61 61) (68 24) (69 514) (73 559) (74 87) (75 389) (86 56) (87 11) (103 14) (130 999) (131 93) (132 40) (141 11) (154 8) (242 6) |
| 20 | 1260,6 | 6,7332 | 31 | (51 219) (55 32) (57 117) (70 40) (71 74) (74 31) (76 44) (77 784) (78 54) (84 36) (85 74) (90 17) (99 20) (104 12) (105 963) (106 78) (121 12) (133 18) (135 733) (136 103) (137 30) (140 8) (161 13) (163 14) (173 10) (179 999) (180 146) (181 45) (193 25) (194 67) (195 13) |
| 21 | 1267,3 | 6,7923 | 72 | (50 6) (57 154) (58 26) (59 76) (60 19) (61 48) (62 3) (65 7) (66 31) (70 10) (72 29) (73 999) (74 127) (75 346) (76 32) (77 20) (80 73) (86 17) (87 25) (88 44) (89 22) (90 6) (91 19) (92 2) (95 5) (100 62) (101 21) (102 37) (103 128) (104 14) (105 11) (115 17) (116 882) (118 43) (119 10) (128 4) (130 62) (131 38) (132 677) (133 107) (134 31) (135 6) (142 1) (144 109) (145 15) (146 69) (147 132) (148 24) (149 17) (150 3) (158 1) (159 47) (160 7) (161 3) (174 2) (175 53) (176 8) (188 17) (189 6) (190 11) (204 3) (206 6) (216 5) (217 4) (218 3) (219 37) (220 7) (221 3) (228 1) (234 26) (235 5) (236 2) |
| 22 | 1268 | 6,7984 | 47 | (55 16) (58 33) (59 72) (60 20) (61 50) (62 3) (65 6) (66 29) (72 31) (73 999) (74 125) (75 416) (76 35) (77 22) (86 17) (87 23) (88 40) (89 21) (90 5) (95 5) (101 21) (102 33) (104 12) (105 12) (115 17) (117 163) (118 43) (119 12) (128 4) (130 59) (131 49) (135 5) (143 5) (145 19) (158 2) (173 3) (174 3) (189 6) (202 9) (203 4) (204 2) (206 5) (216 5) (217 3) (220 6) (221 3) (236 2) |
| 23 | 1272,5 | 6,8383 | 18 | (54 5) (55 13) (67 3) (68 19) (70 116) (81 3) (82 8) (94 5) (96 31) (97 7) (98 999) (99 57) (124 1) (200 13) (231 3) (319 4) (320 1) (321 1) |
| 24 | 1274,9 | 6,8599 | 73 | (56 1) (58 14) (59 124) (60 10) (61 6) (66 2) (72 15) (73 637) (74 52) (75 29) (76 1) (85 3) (86 173) (87 20) (88 12) (89 7) (90 1) (91 1) (100 179) (101 24) (102 13) (103 12) (104 2) (105 5) (106 1) (113 7) (114 25) (115 11) (116 12) (117 22) (118 4) (119 9) (120 1) (121 1) (128 1) (129 3) (130 45) (131 39) (132 10) (133 84) (134 12) (135 6) (136 1) (142 1) (143 1) (144 7) (145 2) (146 5) (147 178) (148 27) (149 14) (150 1) (158 9) (159 2) (160 2) (171 1) (172 14) (173 4) (174 999) (175 181) (176 79) (177 9) (178 2) (183 5) (184 1) (187 1) (188 10) (189 2) (190 1) (204 2) (262 17) (263 4) (264 2) |
| 25 | 1282,1 | 6,9233 | 79 | (52 1) (53 2) (54 1) (55 12) (56 5) (58 21) (59 92) (60 8) (61 14) (62 1) (66 7) (67 1) (70 6) (73 999) (74 86) (75 93) (76 5) (77 2) (86 1) (87 9) (88 7) (89 41) (90 3) (91 2) (99 5) (101 57) (102 9) (103 209) (104 20) (105 15) (106 1) (111 1) (113 7) (115 19) (116 20) (117 317) (118 31) (119 31) (120 2) (129 58) (131 56) (133 227) (134 31) (135 17) (136 2) (143 1) (145 2) (146 1) (147 757) (148 116) (149 83) (150 9) (159 6) (161 2) (163 13) (164 2) (173 1) (175 44) (176 8) (177 23) (178 4) (189 9) (191 41) (192 7) (203 38) (204 52) (205 305) (206 58) (207 22) (217 19) (218 95) (219 20) (220 8) (221 4) (222 1) (263 2) (293 8) (294 2) (295 1) |
| 26 | 1282,3 | 6,9247 | 53 | (53 2) (54 1) (55 15) (56 7) (58 22) (59 93) (60 8) (61 14) (62 1) (66 7) (67 2) (70 8) (73 999) (74 84) (75 94) (76 5) (83 2) (85 17) (86 2) (87 9) (88 7) (89 42) (90 3) (97 1) (99 6) (101 58) (102 9) (103 214) (104 20) (111 1) (113 8) (116 20) (117 321) (118 31) (129 58) (131 54) (143 1) (145 2) (147 754) (148 114) (149 81) (150 8) (163 13) (164 2) (173 1) (175 44) (176 8) (177 22) (203 37) (204 51) (217 18) (218 93) (263 2) |
| 27 | 2495,6 | 16,7363 | 13 | (61 37) (117 146) (131 40) (133 132) (192 65) (193 55) (216 999) (219 18) (232 15) (244 18) (245 28) (246 7) (257 11) |
| 28 | 1284,7 | 6,946 | 70 | (78 1) (93 1) (107 7) (108 1) (109 3) (121 15) (122 2) (123 7) (125 1) (137 24) (138 2) (139 3) (151 18) (152 2) (153 3) (165 11) (166 2) (167 11) (179 8) (180 2) (181 28) (182 4) (183 9) (184 1) (193 62) (194 12) (195 15) (196 2) (197 4) (200 1) (208 11) (209 11) (210 2) (211 100) (212 14) (213 9) (214 1) (223 2) (225 44) (226 8) (227 12) (228 2) (229 1) (239 1) (253 4) (254 1) (255 3) (256 1) (267 5) (268 1) (269 8) (270 2) (271 1) (283 70) (284 16) (285 10) (286 2) (299 999) (300 239) (301 131) (302 22) (303 6) (304 1) (313 2) (314 138) (315 35) (316 19) (317 3) (318 1) (373 1) |
| 29 | 1291,3 | 7,0046 | 18 | (55 224) (56 174) (57 999) (68 31) (69 126) (70 172) (71 846) (72 44) (84 142) (85 521) (86 34) (99 90) (113 46) (126 26) (127 61) (130 36) (146 27) (155 19) |
| 30 | 1304,1 | 7,1175 | 23 | (64 24) (72 129) (76 85) (77 61) (88 999) (89 50) (90 52) (91 111) (100 31) (120 11) (128 10) (135 586) (142 6) (158 537) (159 77) (160 24) (161 3) (170 5) (218 79) (232 19) (233 6) (234 2) (260 4) |
| 31 | 1305,6 | 7,1306 | 54 | (55 29) (56 91) (57 293) (60 14) (66 13) (70 29) (71 112) (73 999) (75 250) (80 34) (83 8) (84 18) (85 70) (86 17) (87 52) (99 17) (101 30) (102 26) (103 17) (104 6) (112 6) (113 11) (114 32) (115 32) (116 19) (117 442) (118 46) (119 19) (127 10) (130 299) (131 103) (132 94) (133 35) (134 7) (140 3) (146 120) (147 141) (148 26) (149 11) (155 6) (176 4) (177 2) (183 12) (198 2) (202 3) (203 4) (204 12) (205 4) (219 130) (220 32) (221 12) (222 2) (248 12) (249 2) |
| 32 | 1307,6 | 7,1484 | 19 | (51 211) (52 31) (78 566) (90 24) (93 8) (94 9) (106 561) (107 39) (120 13) (122 10) (136 755) (137 93) (138 30) (180 999) (181 137) (182 45) (194 8) (195 28) (196 4) |
| 33 | 1310,9 | 7,1768 | 35 | (53 78) (54 32) (55 694) (56 270) (57 847) (65 28) (67 115) (68 57) (69 999) (70 420) (71 614) (81 33) (82 69) (83 474) (84 423) (85 607) (86 44) (95 30) (96 20) (97 137) (98 29) (99 42) (110 30) (111 298) (112 83) (125 86) (126 46) (140 12) (141 31) (142 199) (143 15) (153 21) (154 31) (168 16) (216 6) |
| 34 | 1319,5 | 7,2526 | 108 | (53 13) (54 4) (55 97) (56 40) (57 88) (58 26) (59 149) (60 14) (61 16) (65 2) (66 4) (67 14) (68 8) (69 113) (70 52) (71 60) (72 29) (73 999) (74 86) (75 127) (76 7) (77 7) (81 5) (82 7) (83 55) (84 49) (85 64) (86 227) (87 26) (88 10) (95 5) (97 16) (98 4) (99 8) (100 123) (101 28) (102 14) (103 12) (104 2) (105 5) (109 3) (110 6) (111 39) (112 10) (113 8) (115 11) (116 18) (117 43) (118 7) (119 13) (120 2) (121 1) (125 9) (126 5) (129 18) (130 40) (131 46) (132 12) (133 131) (134 17) (135 10) (144 11) (145 5) (146 3) (147 518) (148 78) (149 43) (150 4) (153 3) (154 4) (158 20) (159 5) (160 5) (161 1) (172 19) (173 9) (175 189) (176 83) (177 14) (178 2) (188 9) (189 3) (190 3) (202 5) (203 2) (204 4) (205 2) (218 2) (246 11) (247 19) (249 38) (250 18) (251 3) (252 1) (262 1) (276 40) (277 9) (279 1) (325 2) (326 1) (327 1) (328 1) (341 8) (342 2) (343 2) (429 3) (430 1) (431 1) |
| 35 | 1328,4 | 7,3316 | 41 | (53 102) (54 35) (55 716) (56 331) (65 21) (67 105) (68 75) (69 755) (70 494) (72 80) (81 39) (82 60) (83 344) (84 425) (85 999) (86 74) (95 19) (96 12) (97 92) (98 49) (103 16) (109 12) (110 31) (111 250) (112 100) (113 117) (125 50) (126 70) (127 102) (128 14) (140 8) (141 35) (152 12) (153 15) (154 33) (155 58) (166 9) (227 5) (240 73) (241 15) (242 7) |
| 36 | 1328,9 | 7,3359 | 30 | (53 46) (54 15) (55 372) (56 188) (57 999) (67 47) (68 34) (69 347) (70 269) (71 928) (72 36) (82 29) (84 212) (85 595) (86 34) (98 25) (99 90) (112 51) (113 79) (126 35) (127 63) (128 6) (140 5) (141 16) (155 37) (156 3) (169 20) (240 37) (241 7) (242 3) |
| 37 | 1332,4 | 7,3664 | 45 | (52 90) (53 37) (54 17) (58 71) (59 55) (60 21) (61 28) (65 25) (66 135) (72 84) (73 999) (74 91) (75 199) (76 19) (83 111) (92 10) (93 246) (94 22) (96 13) (108 67) (110 43) (124 44) (125 53) (137 10) (147 116) (149 14) (151 389) (152 46) (153 17) (164 18) (165 38) (166 80) (167 238) (168 31) (181 9) (182 45) (195 981) (196 142) (197 43) (209 14) (210 11) (217 8) (239 9) (254 16) (261 5) |
| 38 | 1349,7 | 7,5191 | 93 | (55 15) (56 28) (58 71) (59 345) (60 69) (61 20) (62 5) (63 39) (64 8) (69 156) (70 23) (72 47) (73 999) (74 89) (75 70) (76 21) (77 499) (78 64) (81 11) (83 11) (86 326) (87 32) (88 50) (89 10) (90 24) (91 37) (92 5) (97 13) (100 215) (101 38) (102 27) (104 15) (108 7) (110 89) (111 6) (112 6) (113 16) (114 17) (115 18) (116 23) (117 27) (118 65) (119 16) (120 8) (121 4) (129 29) (130 89) (131 20) (133 20) (134 780) (135 96) (136 34) (138 6) (140 46) (143 70) (144 12) (145 4) (146 8) (148 11) (155 4) (158 6) (160 17) (161 3) (165 10) (170 3) (172 69) (173 14) (174 276) (175 50) (176 23) (177 3) (178 38) (179 6) (180 3) (184 924) (185 97) (186 58) (187 11) (188 4) (198 3) (200 15) (201 5) (203 3) (227 70) (228 9) (246 12) (247 6) (285 284) (286 56) (287 26) (288 3) (300 8) (301 2) |
| 39 | 1363,8 | 7,6435 | 73 | (53 14) (54 8) (55 162) (56 27) (60 16) (61 77) (62 4) (63 4) (65 3) (67 39) (68 9) (69 50) (70 11) (71 12) (72 53) (73 999) (74 109) (75 982) (76 70) (77 44) (81 67) (83 21) (85 18) (86 15) (87 8) (88 7) (89 13) (90 2) (91 3) (93 5) (95 8) (98 18) (99 18) (101 17) (105 22) (106 2) (111 12) (113 3) (116 40) (117 712) (118 66) (119 28) (121 2) (123 3) (128 4) (129 282) (130 36) (131 157) (132 154) (133 25) (134 7) (139 2) (140 2) (141 3) (143 20) (144 3) (145 91) (146 8) (157 6) (159 14) (171 28) (172 3) (173 5) (174 2) (185 9) (187 17) (201 14) (213 1) (215 456) (216 77) (217 24) (218 2) (230 6) |
| 40 | 1369,4 | 7,6928 | 24 | (73 752) (100 239) (114 20) (116 74) (144 10) (174 38) (203 10) (204 999) (205 159) (206 78) (216 32) (217 13) (218 537) (219 107) (220 48) (267 19) (268 7) (281 16) (306 24) (307 6) (355 11) (356 5) (444 2) (445 2) |
| 41 | 1424,4 | 8,1614 | 66 | (54 23) (55 93) (56 434) (59 81) (60 19) (61 415) (62 14) (63 21) (72 34) (73 999) (74 114) (75 403) (76 39) (77 30) (81 38) (83 33) (84 32) (87 26) (88 29) (103 47) (104 389) (105 34) (106 18) (108 27) (109 14) (113 25) (114 13) (116 45) (117 27) (129 236) (130 114) (131 113) (132 19) (133 70) (138 31) (139 13) (141 20) (143 40) (144 17) (147 250) (148 61) (149 44) (155 45) (157 17) (159 11) (177 18) (178 43) (179 10) (183 28) (191 12) (198 11) (204 26) (215 70) (216 16) (217 433) (218 91) (219 36) (220 7) (221 46) (229 10) (243 101) (244 44) (245 13) (254 8) (291 18) (292 6) |
| 42 | 1433 | 8,2307 | 110 | (53 3) (54 2) (55 14) (56 3) (57 10) (58 18) (59 43) (60 16) (61 42) (62 3) (63 1) (66 1) (67 1) (68 2) (69 2) (70 48) (71 7) (72 32) (73 999) (74 122) (75 340) (76 30) (77 15) (82 1) (83 2) (84 4) (85 4) (86 11) (87 9) (88 5) (89 6) (90 1) (91 4) (98 5) (99 8) (100 65) (101 13) (102 25) (103 12) (104 2) (105 2) (112 1) (114 2) (115 7) (116 223) (117 206) (118 30) (119 10) (120 1) (123 1) (126 1) (128 18) (129 10) (130 310) (131 47) (132 19) (133 26) (134 5) (135 3) (142 2) (143 3) (144 44) (145 6) (146 27) (147 158) (148 26) (149 20) (150 2) (151 1) (154 1) (155 1) (157 1) (158 1) (159 20) (160 564) (161 68) (162 24) (163 2) (171 1) (172 7) (173 2) (174 7) (175 2) (176 2) (187 4) (188 1) (189 1) (190 3) (191 1) (202 19) (203 3) (204 2) (216 2) (217 2) (218 1) (220 16) (221 3) (222 1) (232 2) (234 36) (235 7) (236 3) (244 7) (245 54) (246 11) (247 5) (262 11) (263 2) (264 1) (277 1) |
| 43 | 1440,8 | 8,2943 | 79 | (53 201) (54 34) (55 405) (56 112) (57 280) (63 34) (64 22) (65 213) (66 69) (67 362) (68 43) (69 189) (70 85) (71 87) (73 397) (77 466) (78 128) (79 681) (80 106) (81 267) (82 118) (83 56) (85 43) (89 46) (91 999) (92 251) (93 625) (94 576) (95 416) (96 51) (98 94) (101 35) (103 102) (104 39) (105 737) (106 239) (107 535) (108 288) (109 318) (110 50) (111 24) (114 11) (115 146) (117 128) (118 25) (119 491) (120 216) (121 247) (122 124) (123 46) (128 42) (129 61) (131 55) (133 457) (134 187) (135 325) (136 81) (137 66) (141 20) (145 56) (147 342) (148 106) (149 55) (161 690) (162 107) (163 83) (164 12) (175 97) (176 42) (189 336) (190 48) (202 39) (203 15) (204 200) (205 34) (233 129) (234 23) (235 9) (304 8) |
| 44 | 1461 | 8,4582 | 46 | (55 216) (56 68) (57 155) (59 38) (61 77) (67 29) (69 70) (70 50) (71 79) (72 60) (73 999) (74 111) (75 931) (76 69) (77 53) (81 46) (83 27) (84 30) (85 66) (86 21) (95 48) (97 25) (98 25) (99 48) (101 23) (105 24) (115 21) (116 47) (117 788) (118 73) (119 33) (129 362) (130 84) (131 157) (132 194) (133 24) (143 30) (145 96) (146 15) (174 31) (185 31) (201 28) (202 8) (229 473) (230 85) (231 24) |
| 45 | 1477,1 | 8,5885 | 6 | (75 66) (98 999) (99 50) (172 43) (200 27) (256 4) |
| 46 | 1480,6 | 8,6171 | 23 | (53 47) (54 53) (55 567) (56 172) (57 642) (63 40) (67 57) (69 418) (70 77) (71 181) (83 152) (84 34) (91 999) (92 37) (93 319) (97 95) (104 17) (105 234) (106 19) (107 48) (119 36) (121 14) (233 10) |
| 47 | 1014,1 | 4,677 | 12 | (59 115) (60 25) (73 999) (74 157) (75 261) (89 192) (100 21) (102 131) (103 26) (104 147) (119 84) (148 61) |
| 48 | 1514,7 | 8,8941 | 22 | (54 15) (56 39) (61 17) (68 17) (75 182) (76 16) (84 999) (86 6) (114 9) (124 4) (140 37) (156 33) (157 83) (158 106) (159 6) (165 4) (180 3) (186 61) (187 7) (188 3) (228 3) (331 2) |
| 49 | 1515 | 8,8964 | 53 | (54 18) (55 27) (56 48) (58 33) (59 32) (60 11) (61 26) (68 21) (72 19) (73 348) (74 49) (75 244) (76 21) (77 17) (82 7) (83 25) (84 999) (85 81) (86 9) (87 5) (93 6) (98 9) (99 15) (102 5) (103 22) (112 7) (113 16) (114 11) (124 4) (125 4) (126 5) (140 36) (142 14) (153 3) (154 9) (156 31) (157 77) (158 101) (159 8) (165 6) (168 3) (180 4) (186 54) (187 6) (188 3) (193 9) (228 3) (231 5) (243 30) (244 18) (245 6) (246 2) (331 3) |
| 50 | 1525 | 8,9773 | 31 | (65 79) (66 77) (67 56) (78 94) (92 165) (93 501) (94 284) (95 20) (96 21) (97 162) (106 52) (107 34) (121 19) (122 333) (123 27) (136 29) (138 301) (139 23) (151 41) (152 92) (153 14) (166 12) (167 14) (180 18) (195 22) (196 148) (197 22) (210 999) (211 381) (212 83) (213 14) |
| 51 | 1540,2 | 9,1008 | 138 | (52 5) (53 15) (54 22) (55 110) (56 366) (57 66) (59 161) (60 33) (61 85) (62 6) (63 3) (65 2) (66 46) (67 32) (68 19) (69 40) (70 51) (72 143) (74 265) (75 804) (76 61) (77 36) (80 9) (81 5) (82 13) (83 40) (84 999) (85 81) (86 55) (87 33) (89 12) (90 2) (91 6) (93 17) (95 6) (96 7) (97 13) (98 21) (99 21) (100 43) (101 28) (102 20) (103 35) (104 4) (105 8) (107 11) (110 15) (111 18) (112 69) (113 18) (114 31) (115 29) (116 37) (117 57) (118 10) (119 12) (121 47) (124 4) (125 4) (126 12) (127 7) (128 17) (129 43) (130 43) (131 91) (132 20) (133 132) (134 20) (135 11) (138 1) (139 6) (140 136) (141 31) (142 34) (143 9) (144 18) (145 21) (146 20) (147 746) (148 118) (149 76) (150 7) (151 2) (152 2) (154 32) (155 19) (157 507) (158 385) (159 47) (160 19) (162 1) (168 6) (169 2) (170 7) (171 1) (172 7) (173 27) (174 549) (175 73) (176 24) (177 2) (183 3) (184 2) (185 4) (186 87) (187 12) (188 5) (189 2) (190 5) (191 2) (198 2) (201 16) (202 5) (203 2) (213 1) (214 43) (215 9) (216 5) (227 1) (228 13) (229 3) (230 309) (231 63) (232 27) (233 4) (246 3) (248 19) (249 4) (250 1) (258 260) (259 55) (260 25) (261 3) (273 8) (274 1) (276 47) (277 10) (278 4) |
| 52 | 1540,6 | 9,1037 | 115 | (52 2) (53 4) (54 6) (55 26) (56 84) (58 32) (59 44) (60 8) (61 21) (62 1) (63 1) (66 13) (67 8) (68 5) (70 12) (72 37) (73 689) (74 69) (75 197) (76 14) (77 8) (80 2) (81 1) (82 3) (83 9) (84 226) (85 18) (86 15) (87 8) (89 3) (91 1) (93 4) (95 1) (96 2) (97 3) (98 6) (99 5) (100 12) (101 7) (102 5) (103 9) (104 1) (105 2) (107 3) (110 4) (112 18) (113 5) (114 8) (115 7) (116 9) (117 14) (118 2) (119 3) (121 13) (124 1) (126 3) (127 2) (128 4) (129 10) (130 10) (131 23) (132 5) (133 34) (134 5) (135 3) (139 1) (140 34) (141 8) (142 8) (143 2) (144 4) (145 5) (146 4) (147 193) (148 30) (149 19) (150 2) (154 8) (155 5) (156 999) (157 129) (158 90) (159 10) (160 4) (168 2) (170 2) (172 2) (173 6) (175 15) (176 5) (183 1) (185 1) (186 19) (187 2) (188 1) (190 1) (191 1) (201 3) (202 1) (214 11) (215 2) (216 1) (228 3) (229 1) (230 75) (231 15) (232 6) (233 1) (246 1) (248 4) (258 64) (259 13) (260 6) (261 1) (273 2) |
| 53 | 1545,2 | 9,1413 | 32 | (53 24) (54 11) (55 258) (56 145) (57 999) (65 7) (67 31) (68 22) (69 200) (70 169) (71 932) (72 60) (82 24) (83 65) (84 86) (85 627) (86 26) (98 25) (99 178) (110 10) (111 53) (112 35) (113 111) (125 19) (126 30) (127 76) (141 14) (154 9) (155 53) (168 6) (169 43) (197 8) |
| 54 | 1554,6 | 9,2179 | 24 | (56 125) (57 994) (70 102) (71 999) (84 102) (85 760) (86 37) (98 32) (99 205) (100 40) (106 20) (113 97) (127 62) (140 25) (155 27) (174 123) (197 17) (211 18) (239 23) (254 123) (255 70) (269 15) (284 57) (285 15) |
| 55 | 1556,5 | 9,2332 | 35 | (59 61) (67 23) (68 16) (72 19) (73 999) (74 85) (101 15) (103 58) (129 79) (131 17) (133 59) (147 253) (148 35) (149 21) (153 13) (157 16) (169 33) (171 10) (185 48) (215 10) (217 91) (218 15) (230 9) (243 204) (244 46) (245 476) (246 97) (247 39) (248 6) (257 11) (258 8) (259 5) (348 23) (349 6) (350 3) |
| 56 | 1556,9 | 9,2359 | 42 | (55 150) (59 55) (60 6) (61 13) (65 7) (67 28) (68 18) (69 191) (72 22) (73 999) (74 85) (75 161) (83 93) (89 13) (95 18) (97 45) (101 15) (109 8) (110 8) (111 73) (112 30) (125 23) (129 85) (131 27) (133 50) (147 233) (148 32) (149 24) (153 12) (167 4) (212 7) (217 74) (218 14) (230 6) (243 149) (244 32) (245 292) (246 63) (247 27) (248 5) (257 7) (258 5) |
| 57 | 1563,9 | 9,2929 | 17 | (55 243) (56 124) (57 955) (70 120) (71 999) (72 46) (82 65) (84 118) (85 618) (97 64) (99 189) (113 146) (127 68) (141 54) (156 72) (169 38) (318 26) |
| 58 | 1565,5 | 9,3063 | 29 | (61 26) (63 15) (65 57) (74 50) (75 170) (76 17) (77 70) (87 20) (89 17) (90 8) (91 227) (92 29) (93 29) (100 19) (103 91) (104 15) (118 42) (119 28) (120 999) (121 95) (130 180) (131 35) (146 368) (160 8) (166 11) (176 11) (177 12) (194 11) (204 36) |
| 59 | 1595,7 | 9,5512 | 123 | (53 5) (54 3) (58 7) (59 42) (60 5) (61 18) (62 1) (65 2) (66 2) (67 2) (68 1) (72 17) (73 873) (74 75) (75 154) (76 10) (77 11) (81 18) (82 1) (87 5) (88 2) (89 15) (90 1) (91 1) (97 1) (98 2) (99 7) (101 17) (102 4) (103 113) (104 10) (105 8) (109 1) (111 13) (112 3) (113 7) (114 2) (115 8) (116 4) (117 31) (118 3) (119 6) (125 3) (127 9) (128 2) (129 101) (130 11) (131 23) (132 3) (133 54) (134 7) (135 6) (136 1) (137 1) (139 1) (141 2) (142 7) (143 57) (144 7) (145 5) (147 113) (148 17) (149 18) (150 2) (151 1) (152 1) (153 4) (154 1) (155 20) (156 3) (157 3) (158 2) (159 5) (160 1) (169 3) (170 2) (171 3) (172 1) (173 1) (174 8) (175 2) (176 1) (177 10) (178 1) (179 1) (187 1) (189 8) (190 2) (191 5) (192 1) (193 1) (199 1) (201 3) (202 1) (214 1) (215 8) (216 3) (217 999) (218 197) (219 87) (220 11) (221 4) (222 1) (230 6) (231 1) (232 1) (241 1) (242 5) (243 26) (244 7) (245 6) (247 4) (248 1) (265 1) (273 2) (303 1) (317 5) (318 1) (319 1) (331 4) (332 5) (333 2) (334 1) |
| 60 | 1609,2 | 9,6534 | 79 | (55 53) (56 34) (57 40) (59 72) (60 45) (61 103) (62 8) (67 8) (68 7) (69 57) (70 85) (71 50) (72 112) (74 257) (75 999) (76 92) (77 40) (83 9) (84 24) (85 20) (86 34) (87 27) (88 13) (90 26) (91 9) (92 3) (98 12) (99 13) (100 185) (102 35) (112 6) (114 43) (115 39) (116 461) (118 76) (123 7) (124 3) (126 6) (128 102) (130 192) (131 128) (132 67) (141 30) (142 22) (144 50) (145 33) (146 25) (150 3) (154 7) (157 15) (158 19) (159 795) (160 110) (161 54) (162 5) (169 6) (171 10) (174 10) (186 31) (187 8) (188 5) (190 14) (202 24) (215 22) (216 10) (232 3) (233 17) (234 3) (241 4) (244 40) (245 7) (246 3) (258 7) (259 4) (261 18) (262 3) (276 14) (277 3) (278 1) |
| 61 | 1614,3 | 9,6909 | 49 | (59 50) (74 79) (77 24) (91 16) (92 5) (93 18) (101 45) (103 42) (106 13) (107 20) (111 11) (117 25) (119 17) (121 18) (129 174) (131 27) (133 112) (137 27) (143 38) (147 131) (148 20) (149 44) (177 6) (181 17) (183 15) (193 10) (195 7) (203 12) (204 6) (207 5) (211 303) (212 47) (213 28) (217 999) (218 201) (219 85) (220 11) (225 20) (227 47) (230 6) (237 10) (243 13) (253 15) (255 7) (283 68) (291 4) (317 5) (344 23) (345 5) |
| 62 | 1614,9 | 9,6948 | 78 | (59 53) (61 9) (65 5) (72 16) (73 999) (74 82) (75 79) (77 24) (85 12) (91 12) (101 32) (103 30) (107 12) (109 10) (111 8) (113 33) (121 11) (123 9) (129 123) (130 8) (131 25) (133 104) (134 15) (135 32) (137 33) (143 21) (147 88) (148 13) (149 29) (153 5) (155 11) (165 4) (167 5) (177 4) (179 14) (180 3) (181 19) (183 17) (184 3) (193 9) (195 8) (197 5) (207 3) (209 4) (211 320) (212 48) (213 28) (223 2) (225 22) (226 5) (227 47) (228 4) (230 4) (237 9) (238 2) (239 114) (240 24) (243 18) (253 15) (254 16) (255 4) (267 2) (268 3) (269 3) (283 65) (284 11) (285 5) (304 1) (317 2) (327 9) (328 3) (329 1) (332 5) (333 2) (342 108) (343 32) (344 16) (345 3) |
| 63 | 1635,1 | 9,8434 | 148 | (53 5) (54 3) (55 7) (56 1) (57 5) (58 7) (59 39) (60 5) (61 17) (62 1) (63 1) (65 2) (66 2) (67 2) (68 1) (69 4) (70 3) (71 4) (72 15) (73 755) (74 66) (75 132) (76 8) (77 10) (78 1) (80 1) (81 15) (82 1) (83 2) (84 1) (85 5) (86 1) (87 4) (88 2) (89 13) (90 1) (91 1) (95 1) (96 1) (97 2) (98 3) (99 8) (100 2) (101 15) (102 4) (103 100) (104 9) (105 7) (109 1) (111 12) (112 2) (113 6) (114 1) (115 7) (116 4) (117 27) (118 3) (119 6) (120 1) (121 1) (125 3) (126 1) (127 11) (129 100) (130 12) (131 22) (132 3) (133 53) (134 7) (135 6) (136 1) (137 1) (139 1) (141 2) (142 7) (143 59) (144 7) (145 5) (146 1) (147 114) (148 18) (149 19) (150 3) (151 1) (152 1) (153 4) (154 2) (155 17) (156 3) (157 3) (158 2) (159 6) (160 1) (161 1) (168 1) (169 3) (170 2) (171 3) (172 1) (173 2) (175 1) (177 12) (178 2) (179 1) (185 1) (187 1) (189 8) (190 2) (191 6) (192 2) (193 1) (194 1) (199 1) (201 4) (202 1) (203 1) (207 1) (215 9) (216 3) (217 999) (218 197) (219 87) (220 11) (221 4) (227 1) (229 1) (230 2) (231 1) (241 1) (242 7) (243 27) (244 7) (245 7) (246 1) (247 4) (248 1) (249 1) (265 1) (273 2) (291 1) (303 1) (317 5) (318 1) (319 1) (331 5) (332 6) (333 2) (334 1) |
| 64 | 1642,4 | 9,8962 | 39 | (59 71) (67 115) (68 24) (88 40) (96 42) (109 60) (119 48) (122 12) (123 31) (133 86) (163 9) (175 11) (177 31) (179 45) (186 15) (189 36) (191 71) (193 72) (194 18) (195 14) (205 43) (206 10) (207 999) (208 213) (209 117) (210 18) (247 11) (254 10) (257 12) (261 42) (262 10) (275 492) (276 121) (277 61) (278 11) (328 60) (329 57) (330 18) (343 7) |
| 65 | 1652,7 | 9,9721 | 73 | (58 20) (59 78) (70 11) (72 85) (73 870) (74 74) (86 32) (87 7) (99 22) (100 76) (101 13) (102 5) (113 49) (114 12) (115 11) (130 9) (131 16) (133 51) (134 6) (135 6) (141 35) (142 7) (144 5) (147 118) (148 19) (149 17) (155 24) (156 10) (157 48) (158 21) (160 9) (161 6) (172 7) (174 9) (185 7) (186 5) (195 3) (209 3) (213 195) (214 38) (215 37) (216 12) (227 5) (228 10) (229 999) (230 206) (231 86) (232 12) (233 3) (243 19) (244 10) (245 4) (253 9) (268 11) (269 3) (279 4) (287 30) (288 10) (289 4) (303 54) (304 14) (305 8) (311 2) (327 64) (328 19) (329 10) (331 38) (332 11) (333 5) (341 10) (342 66) (343 23) (344 10) |
| 66 | 1655,4 | 9,9919 | 46 | (53 21) (55 236) (56 45) (57 118) (61 70) (67 43) (68 12) (69 94) (75 897) (76 65) (77 50) (81 49) (83 47) (88 10) (89 20) (95 49) (97 22) (98 20) (103 20) (105 18) (111 18) (116 54) (117 999) (118 93) (119 39) (129 411) (130 40) (131 111) (132 249) (134 12) (143 31) (145 136) (146 14) (153 9) (159 13) (171 17) (185 13) (187 13) (201 28) (255 9) (257 537) (258 111) (259 34) (272 10) (330 7) (345 13) |
| 67 | 1501,2 | 8,7845 | 30 | (54 8) (55 208) (56 136) (57 999) (65 8) (67 33) (68 29) (69 201) (70 163) (71 967) (82 19) (83 84) (84 127) (85 668) (86 44) (99 182) (100 15) (110 11) (111 48) (112 34) (113 128) (125 18) (126 26) (127 68) (128 6) (141 14) (154 6) (168 4) (197 7) (271 4) |
| 68 | 1662,4 | 10,0428 | 78 | (59 107) (60 15) (61 44) (77 87) (79 37) (89 24) (91 34) (92 7) (98 11) (101 134) (106 24) (107 65) (108 9) (109 50) (113 193) (114 21) (115 49) (116 74) (119 29) (120 9) (121 58) (123 64) (129 118) (130 31) (131 81) (133 157) (134 22) (135 107) (136 14) (137 153) (138 15) (139 31) (144 28) (145 9) (155 30) (163 21) (164 7) (165 12) (169 64) (171 9) (179 14) (181 69) (183 33) (193 37) (195 38) (197 14) (208 9) (209 18) (211 999) (212 150) (213 95) (214 9) (225 85) (227 127) (228 23) (229 10) (240 41) (241 69) (242 15) (243 103) (244 17) (245 16) (253 10) (257 66) (258 11) (259 6) (267 10) (269 4) (270 80) (271 16) (272 6) (283 376) (284 69) (285 34) (286 5) (290 3) (298 5) (323 2) |
| 69 | 1664,8 | 10,0611 | 80 | (54 9) (58 14) (59 85) (60 9) (65 6) (72 26) (73 999) (74 87) (75 101) (76 6) (84 5) (87 7) (95 6) (98 8) (99 26) (100 10) (101 99) (102 12) (103 105) (104 8) (111 9) (112 6) (114 11) (119 16) (125 4) (127 10) (129 112) (130 23) (131 76) (132 9) (133 150) (134 21) (141 6) (143 10) (145 5) (148 45) (149 39) (150 5) (151 28) (152 5) (157 3) (158 3) (161 5) (166 11) (167 52) (168 8) (172 12) (177 4) (185 4) (192 11) (196 5) (197 9) (198 2) (200 17) (203 35) (204 46) (215 7) (216 4) (218 190) (219 90) (220 13) (221 7) (223 4) (229 5) (230 32) (231 8) (239 2) (242 8) (255 8) (256 43) (258 8) (315 13) (316 3) (317 2) (330 1) (331 38) (332 11) (333 5) (346 10) (347 3) |
| 70 | 1665 | 10,062 | 77 | (53 5) (54 7) (55 34) (56 26) (57 121) (69 27) (70 15) (71 78) (72 21) (73 940) (74 79) (84 5) (85 54) (87 5) (95 7) (99 25) (100 7) (103 108) (104 8) (111 7) (112 6) (125 3) (127 10) (132 7) (141 7) (143 11) (147 331) (148 50) (149 40) (150 4) (151 28) (152 4) (159 3) (160 7) (161 5) (166 11) (167 52) (168 8) (172 12) (173 3) (177 4) (185 3) (186 3) (189 7) (191 56) (192 10) (196 4) (198 2) (200 15) (201 9) (202 3) (203 37) (204 52) (205 9) (206 4) (215 8) (216 4) (217 999) (218 201) (219 95) (220 14) (221 7) (223 4) (230 34) (231 8) (232 4) (255 7) (256 44) (304 1) (315 11) (316 3) (317 2) (331 40) (332 12) (333 5) (346 11) (347 3) |
| 71 | 1702,3 | 10,3352 | 21 | (56 137) (57 999) (65 10) (70 75) (71 595) (84 38) (85 381) (97 39) (98 32) (99 87) (110 24) (140 10) (141 16) (154 8) (223 16) (237 21) (238 4) (240 8) (251 10) (281 8) (296 6) |
| 72 | 1715,7 | 10,4339 | 35 | (53 20) (55 237) (56 137) (57 999) (67 24) (68 30) (69 263) (70 152) (71 985) (72 45) (82 22) (83 100) (84 137) (85 705) (86 44) (90 8) (97 28) (98 10) (99 237) (100 22) (110 15) (111 67) (112 40) (113 157) (114 9) (125 30) (126 35) (127 105) (128 10) (141 43) (155 63) (156 30) (169 17) (173 33) (282 8) |
| 73 | 1719,1 | 10,4584 | 106 | (59 151) (60 15) (61 29) (73 999) (74 90) (75 227) (77 25) (87 14) (89 36) (91 24) (92 6) (101 212) (102 31) (103 175) (104 16) (105 90) (106 15) (107 11) (115 40) (116 56) (118 7) (119 16) (121 22) (123 17) (129 128) (130 20) (131 111) (132 9) (133 149) (134 22) (135 41) (136 15) (137 24) (138 8) (139 11) (147 181) (148 36) (149 41) (151 7) (153 20) (154 29) (155 33) (163 25) (166 8) (167 10) (169 9) (179 10) (181 30) (183 16) (185 7) (191 11) (195 28) (197 17) (198 7) (203 19) (204 16) (210 11) (211 197) (212 28) (213 32) (217 19) (218 22) (219 13) (225 38) (226 5) (227 25) (237 41) (238 10) (240 4) (241 99) (242 14) (243 37) (253 184) (254 29) (255 24) (257 21) (267 16) (269 36) (270 17) (283 10) (285 11) (287 5) (298 3) (299 98) (300 22) (301 12) (303 10) (311 275) (312 63) (313 25) (314 5) (327 11) (329 15) (331 5) (341 51) (342 12) (343 18) (344 4) (371 25) (372 7) (373 3) (387 5) (388 2) (399 21) (400 6) (401 3) |
| 74 | 1723,1 | 10,4875 | 69 | (53 43) (55 130) (56 64) (57 256) (58 52) (65 24) (67 48) (69 105) (70 83) (71 270) (72 25) (73 999) (74 58) (79 70) (81 59) (83 60) (84 46) (85 212) (88 51) (91 26) (93 30) (94 15) (95 34) (96 117) (97 33) (99 74) (103 76) (107 29) (108 13) (109 34) (111 30) (113 49) (121 28) (123 24) (127 27) (133 106) (134 15) (135 20) (141 23) (149 28) (151 14) (154 15) (155 33) (167 15) (168 16) (169 40) (179 11) (183 18) (211 47) (212 10) (229 10) (239 42) (240 13) (241 20) (242 13) (255 194) (256 51) (257 77) (258 26) (259 10) (270 7) (330 28) (331 7) (345 178) (346 51) (347 26) (360 38) (361 11) (362 6) |
| 75 | 1728,5 | 10,527 | 49 | (55 61) (56 41) (57 250) (59 46) (69 42) (71 226) (73 999) (74 51) (75 113) (84 34) (85 151) (99 57) (101 50) (103 92) (111 36) (112 15) (113 90) (116 22) (129 100) (133 92) (134 12) (143 25) (147 173) (148 41) (149 20) (155 32) (164 19) (177 23) (180 49) (189 98) (190 18) (191 70) (192 13) (204 349) (205 71) (206 36) (207 517) (208 77) (209 28) (217 312) (218 54) (219 22) (221 12) (222 96) (223 18) (317 7) (333 57) (334 17) (335 9) |
| 76 | 1731,4 | 10,5488 | 79 | (54 39) (55 113) (56 43) (58 85) (59 198) (60 26) (67 40) (70 626) (71 186) (72 197) (73 999) (74 100) (75 210) (77 57) (81 36) (83 48) (84 66) (85 160) (88 39) (93 49) (94 32) (96 37) (97 62) (98 30) (99 69) (100 105) (101 56) (102 30) (112 51) (113 44) (114 109) (115 71) (121 22) (123 39) (124 19) (126 36) (128 39) (129 129) (130 133) (131 81) (132 48) (139 18) (140 14) (146 73) (153 73) (154 29) (155 41) (157 127) (158 89) (170 21) (171 35) (172 90) (173 33) (174 27) (186 21) (187 39) (188 15) (197 16) (200 69) (201 16) (204 141) (227 175) (228 38) (229 24) (237 49) (238 12) (239 10) (240 22) (242 349) (243 496) (244 114) (245 53) (256 82) (257 27) (258 19) (269 9) (295 36) (296 10) (330 22) |
| 77 | 1742 | 10,6263 | 60 | (59 28) (73 999) (74 81) (75 63) (89 34) (91 18) (101 13) (103 60) (104 7) (117 210) (118 19) (119 18) (131 16) (133 76) (134 8) (143 10) (147 391) (148 58) (149 44) (150 6) (156 17) (157 16) (161 4) (163 6) (169 14) (175 9) (187 5) (189 15) (201 6) (204 10) (205 108) (206 16) (215 22) (216 4) (217 54) (221 8) (228 4) (229 5) (230 67) (231 16) (246 255) (247 52) (248 22) (255 4) (257 7) (277 5) (291 3) (303 3) (307 8) (318 204) (319 70) (320 30) (321 7) (327 23) (328 7) (345 10) (346 3) (347 3) (405 3) (406 2) |
| 78 | 1745,3 | 10,65 | 69 | (52 69) (79 107) (84 68) (96 16) (123 15) (167 16) (181 49) (182 10) (183 42) (184 10) (195 36) (196 11) (197 22) (200 18) (211 851) (212 125) (213 83) (214 11) (225 46) (226 11) (227 108) (228 19) (253 16) (255 18) (269 34) (270 8) (271 10) (283 31) (284 9) (285 110) (286 24) (287 19) (289 55) (299 999) (300 236) (301 141) (302 25) (313 15) (314 11) (315 222) (316 61) (327 16) (341 30) (342 18) (343 9) (355 8) (356 4) (357 55) (358 15) (359 9) (369 5) (370 3) (373 116) (374 33) (375 22) (376 4) (387 30) (388 9) (389 161) (390 54) (391 25) (392 6) (415 25) (416 5) (445 104) (446 35) (447 20) (448 5) (484 2) |
| 79 | 1758,1 | 10,7439 | 25 | (53 16) (55 235) (56 114) (57 999) (58 28) (67 23) (69 157) (71 965) (72 28) (82 12) (84 92) (85 677) (99 195) (111 34) (112 16) (113 163) (125 22) (127 96) (141 39) (155 43) (169 44) (183 9) (197 28) (211 11) (212 7) |
| 80 | 1771,8 | 10,8447 | 11 | (66 52) (88 70) (89 39) (101 214) (103 318) (116 550) (117 999) (118 114) (119 53) (161 135) (235 14) |
| 81 | 1780,3 | 10,9068 | 200 | (53 1) (54 2) (55 7) (56 2) (57 8) (58 12) (59 80) (60 7) (61 14) (62 1) (63 1) (70 5) (71 8) (73 999) (74 85) (75 118) (76 7) (77 9) (85 8) (86 1) (87 9) (88 4) (89 11) (90 1) (91 2) (98 1) (99 7) (101 141) (102 15) (103 127) (104 12) (105 11) (106 1) (107 2) (109 1) (111 3) (113 34) (114 4) (115 32) (116 33) (117 30) (118 4) (119 14) (120 2) (121 7) (123 2) (127 2) (128 1) (129 102) (130 19) (131 87) (133 147) (134 20) (135 40) (136 5) (137 11) (138 1) (139 1) (143 1) (145 4) (146 1) (147 250) (148 38) (149 33) (150 4) (151 12) (152 1) (153 3) (155 1) (159 2) (161 2) (162 1) (163 12) (164 2) (165 5) (166 1) (167 4) (175 3) (176 1) (177 4) (178 1) (179 6) (180 1) (181 19) (182 2) (183 9) (184 1) (185 1) (189 7) (190 1) (191 25) (192 5) (193 29) (194 5) (195 26) (196 4) (197 7) (198 1) (199 1) (203 18) (204 5) (205 12) (206 2) (207 54) (208 11) (209 9) (210 3) (211 149) (212 21) (213 14) (214 1) (217 5) (218 39) (219 18) (220 5) (221 3) (222 1) (223 1) (225 39) (226 11) (227 30) (228 5) (229 2) (237 1) (239 1) (241 42) (242 7) (243 21) (244 3) (245 1) (249 1) (251 1) (253 6) (254 1) (255 7) (256 44) (257 7) (258 4) (267 2) (269 11) (270 3) (271 2) (281 1) (283 15) (285 43) (286 9) (287 6) (288 1) (297 1) (299 533) (300 128) (301 72) (302 12) (303 4) (311 1) (313 11) (314 28) (315 143) (316 37) (317 19) (318 4) (319 1) (327 8) (328 33) (329 10) (330 5) (341 49) (342 17) (343 8) (344 3) (345 1) (355 2) (357 415) (358 110) (359 59) (360 11) (361 3) (370 60) (371 18) (372 9) (373 35) (374 11) (375 6) (376 1) (387 54) (388 19) (389 25) (390 7) (391 3) (392 1) (415 7) (416 2) (417 3) (418 1) (429 1) (445 75) (446 26) (447 15) (448 4) (449 1) |
| 82 | 1780,6 | 10,9091 | 90 | (53 1) (54 2) (55 10) (56 3) (57 12) (58 12) (59 82) (60 7) (61 14) (62 1) (63 1) (70 6) (71 12) (73 999) (74 82) (75 118) (76 6) (77 9) (83 3) (84 2) (85 10) (86 1) (87 9) (88 4) (89 11) (90 1) (91 2) (97 2) (98 1) (99 7) (101 137) (102 14) (103 123) (104 11) (105 10) (106 1) (107 2) (109 1) (111 4) (112 1) (113 32) (114 3) (115 30) (116 32) (117 29) (118 4) (119 14) (120 2) (121 6) (123 2) (125 1) (127 2) (128 1) (129 95) (130 17) (131 80) (133 136) (134 18) (135 37) (136 4) (137 10) (138 1) (139 1) (145 4) (146 1) (150 4) (151 11) (152 1) (153 3) (159 2) (161 2) (163 11) (164 2) (165 4) (166 1) (167 4) (177 4) (178 1) (179 5) (181 16) (182 2) (183 8) (189 6) (190 1) (194 4) (197 6) (223 1) (228 4) (237 1) (267 1) |
| 83 | 1511,4 | 8,8668 | 34 | (55 46) (59 83) (60 10) (61 28) (66 55) (67 12) (69 19) (72 31) (73 999) (74 89) (75 141) (76 13) (77 17) (87 32) (88 51) (101 121) (103 175) (104 15) (112 6) (116 286) (117 509) (118 62) (119 34) (145 25) (147 91) (148 11) (149 15) (161 84) (162 11) (191 7) (235 3) (245 8) (275 13) (318 2) |
| 84 | 1785,5 | 10,9445 | 58 | (59 23) (60 5) (72 18) (73 999) (74 88) (81 9) (89 7) (98 7) (113 11) (127 8) (131 27) (133 50) (134 5) (135 5) (140 6) (141 4) (142 8) (143 71) (144 12) (147 209) (148 28) (149 29) (150 3) (151 4) (159 5) (163 8) (169 14) (170 3) (173 5) (177 4) (191 28) (192 3) (201 2) (203 18) (215 4) (218 112) (219 47) (220 7) (222 2) (231 6) (232 21) (233 6) (243 7) (255 5) (257 15) (258 4) (270 6) (271 3) (291 3) (306 5) (317 3) (331 4) (332 2) (344 2) (345 4) (348 3) (349 1) (362 2) |
| 85 | 1801,7 | 11,0624 | 52 | (59 101) (60 11) (61 34) (73 999) (74 97) (100 83) (101 20) (106 8) (107 20) (115 35) (116 36) (118 10) (130 34) (131 27) (133 69) (137 9) (147 88) (148 13) (151 10) (172 126) (173 27) (174 130) (175 22) (176 9) (181 6) (187 13) (188 100) (189 32) (190 9) (191 14) (195 8) (204 41) (205 11) (209 6) (227 4) (274 6) (283 11) (298 9) (299 223) (300 57) (301 30) (302 4) (314 19) (315 39) (316 11) (326 6) (328 29) (329 7) (330 4) (387 3) (414 13) (415 4) |
| 86 | 1804 | 11,0779 | 29 | (76 178) (89 58) (90 23) (91 39) (103 448) (104 246) (105 34) (121 25) (134 47) (135 106) (136 20) (140 94) (177 31) (178 61) (179 15) (192 25) (193 62) (221 301) (222 54) (223 26) (251 239) (252 58) (253 23) (295 999) (296 235) (297 95) (309 11) (310 57) (311 13) |
| 87 | 1828,7 | 11,2423 | 94 | (54 18) (56 15) (57 36) (58 68) (59 39) (60 13) (66 11) (70 53) (71 40) (72 48) (73 999) (74 100) (84 133) (85 58) (86 22) (87 9) (92 6) (98 12) (99 28) (100 50) (101 16) (102 6) (110 19) (111 43) (112 18) (113 14) (114 8) (115 12) (123 14) (125 70) (131 28) (133 12) (136 6) (138 8) (139 11) (140 6) (147 66) (148 15) (150 12) (151 6) (152 16) (153 11) (158 38) (159 15) (164 6) (165 15) (166 50) (167 8) (168 7) (172 47) (173 25) (177 14) (180 19) (181 51) (182 8) (192 18) (193 64) (194 10) (195 4) (197 7) (206 94) (207 60) (208 21) (211 18) (212 5) (217 17) (222 12) (223 9) (224 7) (225 3) (227 6) (228 4) (235 9) (236 4) (237 6) (238 36) (239 15) (240 6) (249 22) (250 6) (251 5) (263 3) (264 2) (265 850) (266 188) (267 79) (268 10) (269 3) (279 32) (280 273) (281 63) (282 27) (299 8) (357 7) |
| 88 | 1837,9 | 11,3032 | 142 | (55 10) (56 10) (58 22) (59 49) (60 7) (61 5) (66 4) (67 6) (68 56) (69 17) (70 107) (71 18) (72 27) (73 999) (74 101) (75 92) (76 6) (82 4) (83 12) (84 35) (85 21) (86 7) (87 7) (88 2) (90 11) (91 4) (97 4) (98 18) (99 52) (100 73) (101 12) (102 4) (103 11) (111 3) (112 8) (113 10) (114 17) (115 45) (116 7) (117 13) (120 6) (124 3) (125 7) (126 5) (127 30) (128 28) (129 17) (130 10) (131 20) (132 15) (133 34) (134 6) (135 3) (138 1) (139 10) (140 37) (141 99) (142 152) (143 28) (144 11) (147 125) (148 24) (149 10) (154 11) (155 25) (156 21) (157 475) (158 69) (159 24) (163 3) (167 8) (168 10) (169 4) (170 7) (171 25) (172 25) (173 10) (182 2) (183 10) (184 6) (185 4) (186 3) (187 16) (188 7) (189 3) (190 3) (202 3) (203 2) (211 8) (212 2) (213 3) (215 10) (216 12) (217 5) (218 20) (219 5) (221 5) (228 1) (229 2) (230 3) (231 8) (232 2) (240 10) (241 3) (242 3) (243 2) (244 19) (245 8) (246 3) (254 2) (255 2) (256 225) (257 56) (258 22) (259 10) (260 2) (268 3) (269 2) (273 40) (274 10) (275 4) (283 3) (284 2) (285 2) (305 2) (319 1) (330 3) (347 9) (349 2) (358 6) (359 1) (363 6) (364 2) (365 1) (373 9) (374 4) (375 8) (376 2) (377 1) (465 3) (466 1) (467 1) |
| 89 | 1851,6 | 11,3944 | 154 | (53 16) (54 15) (55 222) (56 42) (57 87) (58 23) (59 29) (60 11) (61 59) (62 4) (63 2) (65 3) (66 2) (67 40) (68 10) (69 88) (70 19) (71 20) (72 43) (73 879) (74 94) (75 728) (76 50) (77 35) (78 2) (79 12) (80 2) (81 37) (82 6) (83 39) (84 21) (85 23) (86 11) (87 8) (88 6) (89 20) (90 2) (91 6) (92 1) (93 12) (94 2) (95 38) (96 5) (97 32) (98 30) (99 21) (100 3) (101 13) (102 2) (103 4) (105 18) (106 1) (107 7) (108 1) (109 13) (110 3) (111 17) (112 10) (113 4) (114 1) (115 8) (116 57) (117 999) (118 94) (119 42) (120 2) (121 6) (122 1) (123 3) (124 1) (125 5) (126 2) (127 4) (128 3) (129 454) (130 56) (131 133) (132 274) (133 54) (134 13) (135 5) (137 1) (139 2) (140 2) (141 3) (142 1) (143 33) (144 4) (145 171) (146 22) (147 9) (148 1) (149 2) (151 1) (153 2) (154 5) (155 3) (156 2) (157 10) (158 1) (159 20) (160 3) (161 1) (163 1) (167 6) (168 2) (169 1) (171 20) (172 3) (173 6) (181 3) (182 1) (185 27) (186 4) (187 15) (188 4) (189 1) (191 1) (199 8) (201 41) (202 8) (203 2) (210 1) (211 1) (213 4) (214 1) (215 9) (216 2) (227 4) (228 1) (229 4) (230 1) (241 27) (242 6) (243 10) (244 2) (255 4) (256 1) (257 23) (258 5) (259 1) (269 1) (271 4) (272 1) (283 1) (284 1) (285 444) (286 97) (287 26) (288 4) (299 2) (300 17) (301 4) (302 1) |
| 90 | 1860,7 | 11,4568 | 74 | (61 39) (73 999) (74 112) (75 427) (76 29) (82 23) (89 43) (91 68) (96 29) (101 21) (117 213) (118 35) (129 96) (131 64) (132 24) (133 146) (143 28) (145 19) (147 267) (148 38) (149 32) (154 25) (155 38) (157 25) (159 80) (160 39) (161 16) (163 27) (169 41) (177 14) (189 20) (191 102) (192 16) (193 15) (200 20) (204 49) (205 19) (207 36) (208 17) (210 49) (211 20) (215 10) (217 187) (218 38) (230 13) (231 25) (233 34) (236 11) (247 59) (248 13) (254 119) (255 27) (260 134) (261 31) (262 14) (265 24) (305 54) (306 17) (307 10) (318 87) (319 33) (320 15) (343 28) (344 10) (345 9) (367 6) (374 17) (375 10) (432 12) (433 18) (434 8) (449 13) (450 5) (451 3) |
| 91 | 1864,5 | 11,4805 | 31 | (53 19) (54 18) (55 271) (56 204) (57 999) (58 32) (67 31) (68 21) (69 144) (70 91) (71 607) (72 20) (83 84) (84 29) (85 402) (97 56) (98 24) (99 146) (111 30) (112 16) (113 77) (125 14) (127 53) (141 48) (155 37) (169 22) (183 13) (224 23) (225 41) (226 7) (253 4) |
| 92 | 1869,1 | 11,511 | 53 | (59 108) (60 16) (61 19) (68 32) (76 13) (81 49) (82 79) (84 140) (86 121) (87 21) (88 37) (94 16) (95 38) (100 80) (102 31) (109 14) (110 15) (112 17) (114 27) (115 33) (123 19) (124 17) (128 81) (130 52) (131 47) (132 20) (142 24) (144 39) (146 24) (156 135) (157 17) (158 16) (168 26) (172 19) (174 999) (175 187) (176 81) (177 10) (184 12) (186 11) (198 11) (200 172) (201 30) (202 8) (216 7) (230 15) (241 13) (242 5) (258 35) (259 7) (362 21) (363 7) (364 4) |
| 93 | 1869,5 | 11,5139 | 69 | (59 115) (60 16) (61 19) (72 21) (73 999) (74 140) (75 219) (76 13) (82 71) (84 139) (86 121) (87 22) (88 36) (90 9) (94 14) (95 29) (100 79) (101 27) (102 32) (109 12) (110 15) (112 17) (114 28) (115 31) (116 76) (124 13) (126 10) (128 80) (130 48) (131 54) (132 20) (134 17) (142 22) (143 11) (144 42) (146 21) (148 20) (151 13) (156 122) (157 18) (158 16) (168 23) (172 20) (174 862) (175 168) (176 71) (177 10) (184 13) (186 11) (198 12) (200 158) (201 28) (202 7) (204 8) (214 5) (216 6) (224 7) (225 10) (226 7) (230 12) (241 13) (242 4) (258 32) (259 6) (291 24) (292 9) (362 17) (363 6) (364 3) |
| 94 | 1870,7 | 11,5217 | 33 | (59 67) (72 21) (73 999) (74 103) (87 12) (89 35) (90 7) (101 29) (103 61) (104 6) (117 59) (118 10) (119 10) (126 8) (131 32) (133 79) (134 12) (143 7) (147 114) (148 20) (149 12) (163 6) (186 6) (204 7) (226 6) (256 5) (261 3) (290 80) (291 22) (292 9) (316 29) (317 15) (318 6) |
| 95 | 1893,3 | 11,6726 | 53 | (59 39) (72 24) (73 999) (74 90) (84 34) (93 17) (98 27) (101 28) (103 106) (107 22) (112 35) (129 169) (130 32) (133 46) (135 80) (143 16) (147 172) (148 30) (163 12) (165 20) (169 19) (189 21) (191 38) (193 11) (201 9) (203 13) (204 56) (205 13) (206 13) (209 26) (217 87) (218 49) (219 16) (221 12) (231 10) (237 10) (243 8) (244 7) (248 6) (264 265) (265 61) (266 24) (279 53) (280 12) (281 9) (291 14) (319 11) (361 5) (435 5) (437 3) (438 2) (471 3) (472 2) |
| 96 | 1901,3 | 11,7259 | 38 | (53 21) (54 26) (55 255) (56 152) (57 999) (58 50) (67 32) (68 25) (69 134) (70 123) (71 683) (82 28) (83 80) (84 77) (85 438) (86 27) (96 15) (97 57) (98 35) (99 112) (110 6) (111 29) (112 27) (113 64) (125 10) (126 17) (127 38) (140 13) (141 25) (155 15) (168 7) (169 10) (182 4) (183 6) (196 4) (197 7) (268 6) (299 4) |
| 97 | 1906,8 | 11,7626 | 12 | (63 9) (91 37) (146 55) (165 25) (179 999) (180 197) (181 49) (208 69) (209 13) (257 14) (310 19) (311 7) |
| 98 | 1911,9 | 11,796 | 66 | (53 89) (54 259) (55 874) (56 306) (57 999) (65 22) (67 177) (68 99) (69 502) (70 455) (71 449) (76 32) (77 43) (79 86) (80 38) (81 133) (82 524) (83 503) (84 263) (85 248) (93 95) (94 46) (95 68) (96 694) (97 869) (98 170) (99 95) (107 45) (108 33) (109 39) (110 667) (111 336) (112 87) (116 31) (121 24) (122 31) (124 407) (125 108) (126 59) (129 367) (130 56) (136 18) (138 197) (139 44) (140 42) (152 124) (153 24) (154 29) (166 96) (167 13) (168 15) (171 14) (180 98) (181 21) (182 13) (194 147) (195 27) (196 21) (197 11) (208 88) (209 17) (222 28) (243 40) (257 14) (271 25) (347 6) |
| 99 | 1922,2 | 11,8648 | 114 | (53 14) (54 12) (55 161) (56 43) (57 161) (58 19) (59 29) (60 6) (61 31) (65 5) (67 31) (68 10) (69 79) (70 47) (71 70) (72 35) (73 999) (74 101) (75 441) (76 31) (77 28) (79 42) (80 12) (81 26) (82 14) (83 43) (85 41) (86 12) (87 10) (88 5) (89 34) (91 13) (93 29) (94 6) (95 27) (97 33) (98 30) (99 24) (100 20) (101 13) (102 14) (103 65) (104 7) (105 22) (111 18) (112 10) (113 9) (114 8) (116 14) (117 470) (118 47) (119 22) (121 7) (129 222) (130 34) (131 56) (132 118) (134 7) (135 9) (143 20) (145 94) (146 7) (147 142) (148 19) (149 13) (157 37) (158 8) (159 11) (160 82) (161 14) (162 5) (163 6) (170 6) (171 11) (172 5) (173 9) (185 16) (187 5) (200 5) (201 26) (202 4) (205 108) (206 16) (207 12) (214 2) (216 8) (227 3) (229 20) (239 2) (241 5) (255 11) (256 3) (257 19) (258 6) (262 3) (269 5) (271 7) (274 3) (277 5) (278 3) (285 4) (291 5) (299 172) (300 45) (301 12) (302 2) (307 7) (314 7) (315 2) (319 137) (320 41) (321 19) (322 4) (374 2) |
| 100 | 1929 | 11,91 | 187 | (53 2) (54 3) (55 10) (56 2) (58 7) (59 40) (60 4) (61 5) (66 1) (67 4) (68 2) (69 4) (70 5) (71 7) (73 999) (74 84) (75 73) (76 4) (77 2) (80 1) (81 2) (82 5) (83 6) (84 3) (85 6) (86 6) (87 4) (88 4) (89 51) (90 4) (91 3) (95 1) (96 1) (97 2) (98 2) (99 5) (100 13) (101 17) (102 8) (103 101) (104 10) (105 32) (106 3) (107 1) (109 1) (110 1) (111 2) (112 2) (113 5) (114 14) (115 9) (117 100) (118 10) (119 10) (120 1) (121 1) (125 1) (126 2) (127 5) (128 5) (129 120) (130 23) (131 32) (132 5) (133 74) (134 10) (135 6) (138 1) (139 1) (140 1) (141 2) (142 5) (143 16) (144 3) (145 9) (147 397) (148 61) (149 38) (150 4) (151 2) (152 1) (153 1) (154 1) (155 2) (156 2) (157 90) (158 16) (160 174) (161 29) (162 9) (163 12) (164 2) (165 1) (168 2) (169 3) (170 1) (171 1) (172 3) (173 5) (175 6) (176 1) (177 4) (178 1) (180 1) (185 1) (186 3) (187 1) (189 40) (190 10) (191 18) (192 3) (193 2) (196 1) (198 1) (199 1) (200 2) (201 7) (202 2) (203 4) (204 33) (205 205) (206 39) (207 21) (208 3) (209 1) (210 3) (211 1) (214 1) (215 3) (216 11) (217 109) (218 22) (219 10) (220 1) (221 8) (222 2) (223 1) (229 29) (230 8) (231 12) (232 4) (233 5) (234 3) (235 1) (240 1) (241 1) (242 1) (243 2) (244 4) (245 1) (246 3) (247 2) (256 2) (257 1) (259 1) (260 1) (262 5) (263 1) (265 1) (268 1) (269 4) (270 2) (271 1) (274 7) (275 2) (276 2) (277 9) (278 3) (279 1) (290 1) (291 16) (292 5) (293 3) (300 3) (301 1) (304 1) (305 9) (306 4) (307 7) (308 2) (309 1) (331 1) (332 1) (333 1) (344 1) (358 1) (364 5) |
| 101 | 1948,6 | 12,0407 | 156 | (53 5) (54 4) (58 10) (59 36) (60 4) (66 1) (68 2) (70 6) (71 7) (72 21) (73 999) (74 86) (77 8) (82 2) (85 6) (86 4) (87 4) (88 4) (89 56) (90 5) (93 3) (96 2) (97 8) (98 8) (99 7) (100 12) (101 15) (102 6) (103 147) (104 14) (105 19) (106 2) (107 2) (109 3) (111 5) (112 2) (113 4) (114 4) (115 7) (116 15) (118 25) (119 17) (120 1) (121 2) (125 1) (126 2) (127 4) (129 142) (130 22) (131 46) (133 81) (134 12) (135 7) (139 1) (140 1) (142 2) (143 15) (146 6) (147 283) (148 43) (149 28) (150 3) (157 47) (158 8) (159 9) (160 114) (161 22) (162 6) (163 10) (168 1) (169 3) (170 1) (171 4) (172 3) (173 5) (174 7) (181 1) (182 1) (185 6) (186 4) (187 4) (188 3) (189 31) (190 8) (191 13) (196 1) (199 2) (200 1) (201 13) (202 3) (204 25) (205 174) (206 34) (207 17) (208 2) (213 1) (214 4) (215 4) (216 2) (217 77) (218 15) (219 7) (221 6) (223 1) (227 1) (228 1) (229 22) (230 6) (231 5) (233 10) (234 3) (235 6) (236 1) (237 1) (240 1) (241 1) (242 2) (243 4) (244 2) (245 2) (246 2) (247 1) (256 1) (257 2) (259 1) (268 3) (269 2) (270 1) (271 4) (272 1) (273 2) (274 2) (275 1) (277 7) (278 3) (279 1) (285 1) (291 9) (292 3) (293 1) (300 22) (301 6) (302 1) (304 1) (305 5) (306 2) (307 6) (308 1) (309 1) (314 4) (315 1) (322 4) (331 1) (337 1) (365 1) (376 1) |
| 102 | 1948,8 | 12,042 | 118 | (53 5) (54 5) (56 10) (57 28) (58 10) (59 36) (60 4) (61 14) (62 1) (66 1) (67 9) (68 3) (70 7) (71 7) (72 22) (73 999) (74 86) (76 13) (77 9) (81 7) (82 2) (83 12) (84 5) (85 6) (86 4) (87 4) (88 4) (89 54) (90 5) (93 3) (95 7) (96 2) (97 9) (98 8) (99 7) (100 12) (101 15) (102 6) (103 141) (104 13) (105 18) (106 2) (107 2) (109 3) (111 5) (112 3) (113 4) (114 4) (115 6) (116 17) (117 280) (118 28) (119 17) (120 1) (121 2) (125 2) (126 2) (127 4) (129 155) (130 23) (131 49) (132 66) (133 79) (134 11) (139 1) (140 1) (143 16) (144 1) (145 43) (146 6) (157 46) (158 7) (159 9) (168 1) (169 3) (170 1) (171 5) (172 3) (173 4) (174 8) (181 1) (182 1) (185 6) (186 4) (187 5) (188 3) (199 2) (200 1) (201 13) (202 3) (213 1) (214 4) (215 4) (216 2) (223 1) (227 1) (228 1) (230 6) (241 1) (242 2) (243 3) (244 2) (245 1) (255 5) (256 1) (257 2) (259 1) (269 2) (271 4) (272 1) (273 2) (285 1) (299 95) (300 24) (301 6) (302 1) (314 5) (315 1) |
| 103 | 1949 | 12,0435 | 92 | (53 5) (54 5) (55 62) (56 11) (57 25) (58 11) (59 36) (60 5) (61 17) (62 1) (67 11) (68 3) (69 26) (70 7) (71 8) (72 24) (73 999) (74 87) (75 236) (76 16) (77 9) (81 9) (82 2) (83 14) (84 6) (85 6) (86 5) (87 4) (88 4) (90 5) (93 3) (95 8) (96 2) (97 10) (98 9) (99 8) (101 15) (106 2) (107 2) (109 2) (111 6) (112 3) (113 4) (116 19) (117 326) (118 32) (119 18) (121 1) (125 2) (126 2) (129 172) (130 25) (131 52) (132 80) (140 1) (143 16) (144 1) (145 51) (146 7) (159 9) (167 1) (168 1) (171 6) (172 3) (174 9) (181 1) (185 7) (186 4) (187 5) (188 3) (199 2) (201 14) (202 3) (213 2) (215 4) (227 2) (241 1) (243 3) (255 6) (256 2) (257 3) (258 1) (269 2) (271 5) (272 1) (285 1) (299 112) (300 28) (301 7) (302 1) (314 6) (315 1) |
| 104 | 1949,3 | 12,0453 | 75 | (53 14) (54 16) (55 199) (56 36) (58 31) (60 14) (61 54) (62 3) (67 33) (68 9) (69 83) (70 22) (71 22) (72 67) (75 747) (76 49) (77 28) (81 28) (83 42) (84 19) (85 17) (86 13) (87 12) (88 11) (93 8) (95 26) (97 31) (98 27) (99 23) (107 4) (109 6) (111 16) (112 9) (116 57) (117 999) (118 97) (119 52) (125 5) (129 507) (130 71) (131 149) (132 251) (140 2) (143 45) (145 159) (146 22) (159 25) (167 2) (168 3) (171 17) (174 23) (181 4) (185 22) (187 15) (195 1) (199 6) (201 39) (213 5) (215 12) (227 5) (241 3) (255 19) (256 4) (257 8) (258 1) (271 15) (272 3) (285 3) (299 320) (300 80) (301 21) (302 3) (313 1) (314 16) (315 4) |
| 105 | 1958,8 | 12,1086 | 121 | (53 1) (54 2) (55 16) (58 10) (59 26) (60 4) (61 8) (62 1) (63 1) (65 3) (66 2) (67 3) (68 2) (69 10) (70 16) (72 20) (73 999) (74 86) (75 110) (76 7) (77 8) (78 3) (82 3) (84 7) (86 9) (87 4) (88 1) (89 12) (90 7) (91 17) (92 1) (95 2) (96 1) (98 3) (100 133) (101 20) (102 10) (103 22) (104 5) (105 10) (107 3) (109 1) (112 1) (113 3) (114 1) (115 10) (116 4) (117 15) (118 7) (119 6) (125 2) (128 3) (130 15) (131 17) (132 29) (133 30) (134 6) (135 10) (136 2) (137 1) (140 1) (144 3) (145 5) (146 6) (147 116) (148 21) (149 27) (150 4) (151 5) (158 3) (159 4) (160 7) (161 4) (162 3) (163 22) (164 8) (165 12) (166 2) (167 1) (172 2) (173 1) (175 5) (176 6) (177 7) (178 1) (179 76) (180 17) (181 6) (182 2) (183 1) (188 1) (190 6) (191 3) (192 16) (193 5) (194 1) (202 2) (203 7) (204 3) (205 7) (206 2) (207 10) (208 2) (210 1) (221 9) (223 2) (234 1) (248 2) (250 2) (264 3) (265 9) (266 3) (267 2) (291 1) (299 18) (300 4) (301 1) (307 1) (319 7) (320 2) (321 1) |
| 106 | 1963,5 | 12,1397 | 14 | (55 154) (56 166) (57 999) (71 635) (85 433) (99 145) (113 92) (127 62) (141 53) (155 42) (174 31) (195 19) (197 21) (239 28) |
| 107 | 1967,3 | 12,1654 | 73 | (59 25) (63 5) (72 14) (73 999) (74 81) (75 67) (87 4) (88 5) (89 28) (101 20) (103 208) (104 19) (105 9) (117 98) (118 10) (119 9) (129 54) (130 6) (131 18) (133 61) (134 8) (135 6) (143 11) (147 320) (148 52) (149 33) (157 38) (163 4) (175 10) (189 42) (190 9) (191 32) (192 6) (200 6) (201 3) (203 3) (204 43) (205 196) (206 39) (207 19) (208 3) (217 186) (218 41) (219 18) (221 8) (229 20) (230 6) (231 18) (255 8) (259 5) (277 12) (278 4) (279 2) (291 11) (292 3) (305 14) (306 6) (307 43) (308 11) (309 6) (319 209) (320 61) (321 30) (322 6) (331 15) (332 4) (345 11) (346 4) (347 2) (419 4) (421 5) (422 2) (423 1) |
| 108 | 1967,5 | 12,1668 | 77 | (54 11) (56 45) (58 17) (59 27) (61 6) (63 5) (67 16) (68 19) (72 26) (73 999) (74 82) (75 88) (76 6) (82 21) (83 54) (87 5) (88 5) (89 29) (101 19) (103 188) (104 17) (105 10) (112 20) (117 99) (118 10) (119 9) (129 60) (130 8) (131 19) (133 57) (135 6) (143 15) (145 7) (147 284) (148 46) (149 29) (157 39) (163 4) (172 4) (175 8) (183 4) (189 37) (190 7) (191 27) (192 5) (200 5) (201 3) (204 36) (205 165) (206 32) (207 17) (217 153) (218 33) (219 16) (221 9) (229 17) (230 5) (231 14) (255 7) (259 4) (277 11) (278 4) (291 9) (292 3) (305 11) (307 33) (308 9) (309 5) (320 46) (321 22) (322 5) (331 11) (332 4) (345 9) (419 3) (421 4) (423 1) |
| 109 | 1970,8 | 12,1886 | 32 | (53 21) (55 307) (56 143) (57 999) (58 42) (69 257) (71 973) (84 132) (85 796) (86 53) (97 38) (98 32) (99 297) (110 20) (111 62) (113 197) (125 44) (126 40) (127 137) (128 19) (141 78) (155 70) (159 81) (168 11) (169 49) (197 18) (211 11) (281 16) (341 7) (355 5) (393 5) (429 5) |
| 110 | 1990,7 | 12,3207 | 39 | (59 54) (60 19) (61 72) (72 83) (73 994) (74 138) (75 999) (76 79) (77 50) (91 33) (107 22) (115 128) (116 492) (118 23) (119 23) (124 55) (128 142) (129 167) (130 44) (131 870) (132 136) (133 75) (142 18) (144 136) (145 61) (148 39) (152 58) (158 23) (184 45) (186 11) (200 148) (201 23) (208 12) (216 19) (226 186) (227 29) (316 152) (317 39) (447 10) |
| 111 | 2002,9 | 12,4003 | 39 | (59 37) (73 999) (74 91) (75 134) (89 37) (101 18) (103 87) (118 12) (129 70) (130 26) (133 52) (134 11) (143 17) (147 179) (148 31) (149 24) (157 21) (161 13) (172 11) (173 9) (189 44) (190 13) (200 24) (203 11) (217 279) (218 60) (219 37) (220 9) (231 10) (243 28) (244 16) (271 15) (272 21) (273 7) (286 7) (319 8) (361 40) (362 12) (363 6) |
| 112 | 2009,6 | 12,4409 | 40 | (55 205) (56 55) (57 78) (61 42) (69 91) (70 32) (72 34) (73 864) (74 90) (75 611) (76 39) (83 56) (95 23) (98 30) (116 32) (117 999) (118 71) (129 435) (130 48) (131 80) (132 290) (133 47) (143 36) (145 181) (146 40) (185 27) (187 12) (201 29) (204 17) (215 8) (229 11) (257 9) (269 22) (285 22) (313 347) (314 94) (315 21) (319 13) (328 23) (329 7) |
| 113 | 2014 | 12,4676 | 108 | (53 90) (54 84) (55 219) (58 18) (59 30) (60 14) (61 75) (62 7) (63 9) (65 108) (66 182) (67 425) (68 79) (69 46) (72 48) (73 824) (74 97) (75 999) (76 70) (77 296) (78 129) (79 862) (80 382) (81 257) (82 75) (83 35) (84 26) (89 23) (90 5) (91 516) (92 137) (93 264) (94 151) (95 127) (96 73) (97 35) (98 10) (101 13) (103 29) (104 23) (105 266) (106 100) (107 198) (108 65) (109 45) (110 34) (115 27) (116 59) (118 40) (119 119) (120 109) (121 97) (122 21) (123 22) (124 21) (129 179) (130 33) (131 80) (133 69) (134 36) (135 60) (136 34) (137 15) (138 13) (139 8) (142 10) (144 7) (145 46) (146 15) (147 44) (148 13) (149 41) (150 75) (151 19) (155 24) (156 9) (157 27) (159 29) (160 8) (161 28) (162 16) (163 14) (164 8) (165 5) (169 18) (170 6) (171 30) (173 19) (177 8) (178 5) (179 6) (183 22) (185 14) (186 5) (188 9) (189 14) (192 24) (199 8) (201 11) (202 6) (211 7) (212 5) (213 5) (234 29) (252 5) (291 6) (309 18) (310 5) |
| 114 | 2019,4 | 12,5006 | 174 | (50 5) (51 17) (52 12) (53 81) (54 73) (55 291) (56 18) (57 44) (58 21) (59 30) (60 14) (61 74) (62 7) (63 6) (64 2) (65 97) (66 91) (67 734) (68 65) (69 68) (70 8) (72 51) (73 960) (74 100) (75 999) (76 71) (77 270) (78 138) (79 798) (80 386) (81 260) (82 45) (83 33) (84 34) (86 10) (87 6) (88 8) (89 20) (90 2) (91 408) (92 100) (93 379) (94 169) (95 138) (97 20) (99 10) (101 16) (102 4) (103 21) (104 14) (105 182) (106 81) (107 179) (108 85) (109 116) (110 12) (111 8) (115 22) (116 66) (117 170) (118 34) (119 108) (120 138) (121 126) (122 122) (123 33) (124 4) (125 4) (127 5) (128 7) (129 232) (130 39) (131 88) (132 22) (133 85) (134 44) (135 85) (136 51) (137 10) (139 3) (141 6) (142 9) (143 20) (144 11) (145 72) (146 12) (147 54) (148 26) (149 76) (150 32) (151 7) (153 5) (154 2) (155 19) (156 9) (157 30) (158 7) (159 26) (160 5) (161 43) (162 31) (163 28) (164 8) (165 3) (167 4) (168 2) (169 11) (170 3) (171 24) (172 4) (173 18) (174 5) (175 33) (176 14) (177 7) (178 4) (181 4) (182 2) (183 10) (184 2) (185 24) (186 4) (187 9) (188 5) (189 13) (190 21) (195 3) (196 1) (197 5) (203 10) (204 5) (205 2) (209 5) (210 2) (211 8) (212 3) (213 5) (214 3) (215 15) (216 3) (223 7) (224 3) (225 6) (232 15) (233 3) (235 5) (238 4) (239 3) (241 1) (249 12) (250 3) (251 3) (252 11) (253 3) (254 1) (263 7) (264 2) (265 6) (266 2) (277 5) (278 2) (279 4) (280 1) (289 2) (307 16) (308 4) (309 2) (313 4) (314 1) (322 8) (323 2) (423 3) (424 1) (438 1) |
| 115 | 2023,9 | 12,5279 | 90 | (53 37) (54 98) (55 417) (56 81) (57 124) (58 22) (59 26) (60 10) (61 54) (62 6) (63 31) (68 95) (69 165) (70 49) (71 58) (72 46) (73 843) (74 90) (75 805) (76 61) (81 167) (82 116) (83 139) (84 284) (85 62) (86 16) (87 10) (88 12) (89 33) (95 134) (96 235) (97 140) (98 143) (99 27) (101 22) (109 59) (110 86) (111 55) (112 23) (116 71) (117 999) (118 94) (119 45) (123 73) (124 38) (125 29) (129 486) (130 62) (131 44) (132 64) (137 39) (138 30) (139 8) (141 15) (142 14) (143 21) (145 133) (146 16) (151 19) (152 59) (153 12) (155 71) (156 13) (157 12) (159 13) (166 11) (169 27) (170 7) (171 28) (183 25) (184 6) (185 58) (186 12) (192 9) (194 65) (195 11) (198 9) (199 52) (200 11) (201 8) (208 10) (213 16) (221 21) (227 11) (229 9) (236 33) (283 6) (311 159) (313 8) (326 17) |
| 116 | 2030,2 | 12,5659 | 206 | (51 4) (52 2) (53 52) (54 113) (55 532) (56 99) (57 61) (58 20) (59 29) (60 13) (61 73) (62 5) (63 3) (65 21) (66 15) (67 234) (68 84) (69 216) (70 42) (71 22) (72 51) (73 999) (74 109) (75 984) (76 69) (77 63) (78 9) (79 80) (80 34) (81 200) (82 98) (83 128) (84 156) (85 37) (86 14) (87 8) (88 6) (89 25) (90 3) (91 37) (92 10) (93 53) (94 29) (95 147) (96 201) (97 122) (98 155) (99 35) (100 5) (101 12) (102 2) (103 4) (105 32) (106 7) (107 31) (108 20) (109 82) (110 88) (111 54) (112 31) (113 9) (114 2) (115 8) (116 70) (117 808) (118 78) (119 64) (120 10) (121 33) (122 12) (123 70) (124 38) (125 18) (126 6) (127 7) (129 729) (130 90) (131 127) (132 88) (133 55) (134 25) (135 23) (136 9) (137 48) (138 37) (139 14) (140 4) (141 15) (142 9) (143 48) (144 8) (145 209) (146 28) (147 39) (148 14) (149 11) (151 30) (152 77) (153 16) (154 2) (155 39) (156 10) (157 26) (158 8) (159 21) (160 3) (161 8) (162 4) (163 5) (164 2) (165 19) (166 14) (167 7) (168 5) (169 18) (170 9) (171 36) (172 13) (173 17) (174 4) (175 7) (176 2) (177 2) (178 1) (179 12) (180 6) (181 6) (182 2) (183 26) (184 5) (185 62) (186 15) (187 14) (188 6) (189 5) (190 2) (192 15) (193 19) (194 75) (195 14) (196 2) (197 6) (198 1) (199 67) (200 13) (201 17) (202 3) (203 2) (207 10) (208 11) (209 2) (211 6) (212 1) (213 18) (214 3) (215 5) (218 14) (219 5) (221 3) (222 1) (223 1) (225 4) (226 1) (227 15) (228 4) (229 9) (230 4) (231 2) (235 1) (236 44) (237 8) (238 1) (239 3) (240 1) (241 8) (242 2) (243 7) (244 7) (245 2) (253 3) (254 1) (255 6) (256 1) (257 2) (258 4) (259 1) (267 7) (268 1) (269 3) (270 1) (271 3) (272 1) (281 2) (282 1) (283 7) (284 2) (285 1) (295 2) (297 2) (311 358) (312 86) (313 23) (314 3) (326 18) (327 5) (328 1) |
| 117 | 2035 | 12,5948 | 201 | (51 19) (52 13) (53 99) (59 45) (60 16) (62 6) (63 7) (64 2) (65 98) (66 62) (67 999) (68 248) (74 184) (76 79) (77 199) (78 55) (79 489) (80 213) (81 678) (82 273) (87 10) (88 8) (89 31) (90 3) (91 157) (92 34) (93 223) (94 156) (95 313) (101 25) (102 11) (103 103) (104 19) (105 65) (106 26) (107 135) (108 109) (109 153) (110 82) (113 8) (115 13) (116 38) (119 50) (120 25) (121 157) (122 78) (123 62) (124 38) (125 16) (126 5) (131 111) (133 91) (134 18) (135 148) (136 118) (137 33) (138 27) (139 12) (141 5) (142 5) (143 59) (144 8) (146 9) (147 338) (148 55) (149 118) (150 203) (151 40) (153 8) (157 34) (158 6) (162 2) (163 47) (164 32) (167 5) (169 12) (170 6) (171 23) (172 6) (173 32) (175 7) (177 25) (178 16) (180 3) (181 6) (183 14) (184 3) (187 21) (189 46) (190 21) (191 39) (192 67) (193 12) (195 3) (196 2) (197 8) (198 4) (201 12) (202 2) (203 6) (204 49) (205 98) (206 37) (207 17) (209 3) (211 4) (212 3) (213 6) (215 11) (216 5) (217 220) (218 45) (219 31) (220 8) (221 21) (222 5) (223 4) (225 4) (226 3) (227 5) (229 15) (230 4) (231 10) (232 4) (233 5) (234 131) (235 25) (239 4) (240 2) (241 4) (243 8) (245 8) (246 2) (247 2) (253 2) (257 6) (259 3) (260 1) (265 3) (269 5) (277 21) (278 6) (279 5) (281 2) (285 3) (288 3) (291 14) (292 139) (293 44) (294 22) (295 4) (303 2) (305 80) (306 26) (307 22) (308 5) (309 284) (310 70) (315 3) (317 2) (318 5) (319 63) (320 20) (321 13) (322 3) (324 9) (325 2) (331 12) (332 4) (333 160) (334 47) (335 25) (336 5) (337 1) (343 2) (345 8) (346 3) (347 2) (359 24) (360 8) (361 4) (379 3) (380 1) (389 2) (393 3) (394 1) (395 1) (405 4) (406 2) (407 3) (421 2) (423 17) (424 6) (425 3) (426 1) (433 15) (434 7) (435 16) (436 6) (437 3) (438 1) |
| 118 | 2035,3 | 12,5965 | 216 | (51 8) (52 5) (53 52) (54 124) (55 207) (56 11) (58 12) (59 23) (60 8) (61 44) (62 3) (63 3) (65 47) (66 30) (67 477) (68 120) (69 71) (72 34) (73 999) (74 94) (75 624) (76 41) (77 95) (78 25) (79 228) (80 99) (81 324) (82 133) (83 39) (86 4) (87 4) (88 4) (89 16) (90 2) (91 75) (92 16) (93 105) (94 72) (95 154) (96 85) (97 32) (99 9) (101 9) (102 5) (103 47) (104 8) (105 31) (106 12) (107 63) (108 50) (109 74) (110 43) (111 14) (113 4) (115 6) (116 16) (117 136) (118 16) (119 27) (120 11) (121 72) (122 35) (123 33) (124 19) (125 8) (126 2) (129 195) (130 27) (131 57) (132 12) (133 46) (134 9) (135 67) (136 52) (137 17) (138 14) (139 6) (141 3) (142 2) (143 29) (144 4) (145 24) (146 5) (147 164) (148 27) (149 54) (150 90) (151 18) (153 4) (155 7) (157 17) (158 3) (159 8) (163 21) (164 13) (165 3) (167 3) (169 6) (170 3) (171 12) (172 3) (173 15) (175 3) (177 11) (178 7) (179 1) (180 1) (181 3) (183 7) (184 2) (185 4) (187 10) (189 22) (190 9) (191 17) (192 29) (193 6) (195 2) (197 4) (198 1) (199 4) (200 2) (201 6) (203 3) (204 23) (205 46) (206 17) (207 8) (209 1) (211 2) (212 1) (213 3) (215 5) (216 2) (217 93) (218 20) (219 13) (220 3) (221 10) (222 2) (223 2) (225 2) (226 1) (227 2) (229 7) (230 2) (231 4) (232 1) (233 2) (234 55) (235 10) (239 2) (240 1) (241 1) (243 4) (245 4) (246 1) (253 1) (257 3) (259 1) (263 1) (265 1) (269 2) (277 10) (278 3) (279 2) (288 1) (291 6) (292 63) (293 19) (294 10) (295 2) (303 1) (305 35) (306 11) (307 10) (308 2) (309 113) (310 27) (315 1) (317 1) (318 2) (319 28) (320 9) (321 6) (322 1) (324 4) (325 1) (331 5) (332 2) (333 71) (334 21) (335 11) (336 2) (343 1) (345 4) (346 1) (347 1) (359 10) (360 3) (361 2) (379 1) (389 1) (393 1) (405 2) (406 1) (407 1) (421 1) (423 7) (424 2) (425 1) (433 6) (434 3) (435 7) (436 2) (437 1) |
| 119 | 2035,5 | 12,5979 | 153 | (52 6) (53 49) (54 122) (60 7) (62 3) (63 3) (65 51) (66 32) (67 513) (68 122) (73 999) (74 93) (77 99) (78 29) (79 257) (80 113) (81 346) (82 135) (89 14) (90 2) (91 81) (92 17) (93 114) (94 79) (95 150) (102 6) (103 61) (104 10) (105 32) (106 13) (107 69) (108 55) (109 74) (115 6) (119 25) (120 12) (121 79) (122 39) (133 47) (135 75) (136 59) (142 2) (143 29) (144 4) (147 204) (148 32) (149 63) (150 101) (151 18) (157 17) (158 3) (163 23) (164 16) (169 6) (173 15) (175 3) (177 13) (178 8) (181 3) (187 9) (189 27) (190 11) (191 21) (192 32) (197 4) (198 1) (203 3) (204 28) (205 55) (206 20) (207 9) (209 1) (212 1) (215 5) (217 110) (218 22) (219 15) (220 4) (221 12) (222 3) (223 2) (225 2) (226 1) (229 8) (230 1) (231 4) (232 1) (233 2) (234 61) (235 11) (239 2) (240 1) (243 4) (245 4) (246 1) (257 3) (259 2) (265 2) (269 2) (277 12) (278 3) (279 3) (288 1) (291 8) (292 75) (293 23) (294 12) (295 2) (303 1) (305 42) (306 13) (307 11) (308 3) (309 128) (310 30) (315 2) (317 1) (318 2) (319 33) (320 10) (321 7) (322 1) (324 4) (331 6) (332 2) (333 83) (334 24) (335 13) (336 2) (343 1) (345 4) (346 1) (347 1) (359 12) (360 4) (361 2) (379 1) (380 1) (389 1) (393 2) (394 1) (405 2) (406 1) (407 1) (421 1) (423 8) (424 3) (425 2) (433 7) (434 3) (435 8) (436 3) (437 1) |
| 120 | 2039,2 | 12,6202 | 61 | (56 388) (57 688) (70 188) (71 425) (84 418) (85 367) (97 302) (98 354) (99 144) (111 153) (112 74) (113 56) (118 138) (130 142) (132 221) (140 56) (141 40) (145 396) (146 41) (152 131) (153 31) (154 47) (155 53) (156 16) (159 238) (160 30) (165 40) (166 31) (168 21) (171 64) (179 14) (182 24) (183 36) (184 17) (185 140) (186 41) (194 144) (195 29) (199 121) (200 25) (201 27) (208 27) (213 45) (214 24) (216 24) (227 28) (236 104) (237 19) (241 18) (242 6) (244 16) (255 16) (267 27) (281 13) (283 24) (311 999) (312 258) (313 62) (314 9) (326 43) (327 17) |
| 121 | 2039,5 | 12,6219 | 152 | (53 43) (54 91) (55 546) (56 112) (57 170) (58 25) (59 24) (60 9) (61 55) (62 4) (63 4) (65 14) (66 8) (67 156) (68 74) (69 193) (70 52) (71 106) (72 42) (73 999) (74 98) (75 759) (76 55) (77 42) (79 44) (80 11) (81 122) (82 87) (83 96) (84 110) (85 96) (86 13) (87 10) (88 7) (89 20) (91 14) (92 8) (93 25) (94 13) (95 100) (96 115) (97 91) (98 89) (99 40) (100 6) (101 12) (105 14) (107 12) (108 8) (109 45) (110 46) (111 46) (112 17) (113 14) (115 5) (116 53) (117 431) (118 47) (119 27) (120 4) (121 9) (122 4) (123 35) (124 19) (125 11) (126 19) (127 11) (129 380) (130 47) (131 65) (132 55) (133 24) (134 10) (135 5) (137 19) (138 17) (139 9) (140 13) (141 14) (143 19) (144 3) (145 106) (146 13) (151 10) (152 31) (153 8) (154 9) (155 15) (156 5) (157 10) (158 3) (159 51) (160 5) (161 7) (165 11) (166 8) (167 3) (168 5) (169 9) (170 3) (171 20) (172 5) (173 9) (174 5) (175 4) (176 2) (178 2) (179 3) (180 2) (182 7) (183 11) (184 4) (185 34) (186 11) (187 8) (188 4) (192 5) (193 8) (194 30) (195 8) (196 2) (197 3) (199 28) (200 5) (201 8) (207 5) (208 5) (213 11) (214 5) (215 3) (216 5) (225 3) (227 7) (228 2) (229 4) (230 2) (236 20) (237 4) (241 4) (243 3) (244 4) (245 4) (255 3) (258 2) (267 6) (283 3) (311 169) (312 47) (313 9) (314 2) (326 6) (327 3) |
| 122 | 2049,3 | 12,6813 | 41 | (80 52) (100 11) (111 30) (115 800) (117 999) (118 55) (121 19) (123 14) (126 11) (129 762) (132 265) (133 60) (140 10) (141 14) (144 118) (145 290) (153 10) (154 14) (158 37) (160 13) (161 7) (162 2) (164 5) (168 8) (169 11) (172 13) (173 25) (174 12) (176 3) (180 3) (188 18) (189 8) (190 3) (191 5) (192 2) (273 3) (278 4) (293 2) (297 5) (311 7) (401 2) |
| 123 | 2050,6 | 12,6895 | 152 | (50 1) (51 2) (53 21) (54 22) (55 295) (56 61) (57 150) (58 29) (59 34) (60 12) (61 66) (62 4) (63 3) (65 5) (67 58) (68 15) (69 118) (70 27) (71 34) (73 999) (75 811) (76 59) (77 41) (78 2) (79 19) (81 48) (82 9) (83 60) (84 24) (85 27) (86 12) (87 8) (88 6) (89 21) (90 3) (91 8) (92 1) (93 18) (95 47) (96 7) (97 45) (98 35) (99 24) (100 4) (101 12) (102 2) (103 3) (105 18) (106 2) (107 9) (108 1) (109 17) (111 25) (112 11) (113 5) (114 1) (117 907) (118 89) (119 39) (120 2) (121 9) (122 1) (123 6) (124 1) (125 6) (126 4) (127 5) (129 451) (130 56) (131 148) (132 229) (133 53) (134 12) (135 7) (136 1) (137 2) (139 4) (140 3) (141 3) (143 36) (145 167) (146 21) (147 11) (149 3) (151 1) (153 3) (154 5) (155 3) (157 10) (159 20) (160 3) (161 1) (163 1) (167 3) (168 2) (169 1) (171 21) (172 3) (173 8) (174 3) (175 1) (177 1) (181 2) (182 3) (183 1) (185 30) (186 5) (187 18) (188 5) (189 1) (195 6) (196 1) (199 7) (201 40) (202 6) (203 2) (209 2) (213 7) (214 1) (215 6) (216 2) (219 1) (227 8) (229 10) (230 2) (239 1) (241 5) (243 12) (244 2) (245 1) (255 3) (256 1) (257 9) (258 2) (269 21) (270 5) (271 6) (272 1) (283 4) (285 18) (286 4) (287 1) (297 1) (299 5) (300 1) (313 345) (314 81) (315 21) (316 3) (328 20) (329 5) (330 1) |
| 124 | 2059,6 | 12,7439 | 175 | (52 7) (53 7) (54 10) (58 20) (59 50) (60 6) (61 20) (62 1) (63 1) (65 1) (66 6) (67 7) (68 10) (69 24) (70 27) (72 24) (73 592) (74 58) (75 101) (76 7) (77 4) (78 2) (79 14) (80 5) (81 4) (82 8) (84 41) (86 16) (87 9) (88 4) (89 3) (91 2) (92 2) (93 5) (94 5) (95 8) (98 9) (99 70) (100 31) (101 25) (102 6) (103 11) (104 3) (105 8) (106 23) (107 6) (108 2) (109 3) (110 6) (111 27) (112 5) (113 33) (114 7) (115 17) (116 6) (118 8) (119 6) (120 4) (121 8) (122 6) (123 7) (124 11) (125 11) (126 5) (127 5) (128 2) (130 13) (131 61) (132 32) (133 18) (134 4) (135 3) (136 3) (137 4) (138 3) (143 7) (145 2) (146 5) (147 162) (148 56) (149 18) (150 11) (151 3) (152 22) (153 5) (154 3) (155 3) (156 2) (157 16) (158 9) (159 3) (160 1) (161 1) (162 2) (163 8) (164 3) (165 4) (166 5) (167 3) (168 2) (171 43) (172 9) (173 9) (176 1) (177 2) (178 6) (179 110) (180 54) (181 12) (182 4) (184 1) (185 1) (187 1) (190 2) (192 3) (193 4) (194 3) (195 4) (196 5) (197 2) (198 2) (199 1) (201 1) (202 1) (204 56) (205 9) (206 16) (207 4) (208 6) (209 4) (210 2) (212 1) (217 8) (218 5) (219 8) (220 47) (221 17) (222 14) (223 6) (224 2) (225 1) (234 1) (235 15) (236 16) (237 8) (250 1) (251 4) (262 1) (264 3) (265 4) (266 2) (267 4) (268 1) (269 1) (277 1) (278 59) (279 13) (280 6) (281 1) (292 5) (293 2) (294 999) (295 231) (296 88) (297 14) (298 2) (306 1) (308 11) (309 196) (310 48) (311 18) (312 3) (319 2) (327 1) (333 1) |
| 125 | 2063,9 | 12,7698 | 57 | (50 23) (52 26) (53 26) (54 35) (55 362) (56 231) (57 999) (58 53) (67 47) (68 38) (69 198) (70 126) (71 611) (72 30) (81 14) (82 31) (83 124) (84 40) (85 422) (86 32) (88 4) (89 10) (96 22) (97 90) (98 20) (99 142) (100 11) (111 43) (112 18) (113 78) (114 11) (125 15) (126 8) (127 56) (136 4) (140 6) (141 38) (142 5) (155 41) (156 4) (166 5) (167 6) (168 5) (169 25) (174 26) (183 24) (184 5) (197 16) (211 12) (214 3) (224 4) (225 6) (243 4) (252 18) (253 35) (254 8) (281 6) |
| 126 | 2070 | 12,8068 | 143 | (53 36) (54 15) (55 326) (57 334) (58 44) (59 41) (60 16) (61 56) (65 32) (66 12) (67 54) (68 17) (69 64) (72 52) (73 999) (74 98) (75 815) (76 60) (77 96) (78 28) (79 65) (81 50) (82 27) (83 26) (86 39) (87 12) (89 24) (90 7) (91 152) (92 18) (93 45) (95 32) (96 18) (101 16) (103 26) (104 10) (105 89) (106 11) (107 39) (109 16) (115 91) (116 84) (117 192) (118 23) (119 73) (120 15) (121 45) (122 10) (123 13) (127 16) (128 110) (129 139) (130 38) (131 103) (132 22) (133 65) (134 14) (135 23) (136 8) (138 38) (139 8) (141 57) (142 48) (143 56) (144 26) (145 76) (146 170) (147 718) (148 77) (149 25) (150 6) (152 18) (153 22) (154 17) (155 32) (156 19) (157 37) (158 13) (159 34) (160 108) (161 171) (162 27) (163 17) (164 6) (167 14) (168 8) (169 18) (170 16) (171 16) (172 8) (173 37) (174 15) (175 37) (176 22) (177 17) (179 10) (180 6) (185 44) (186 14) (187 73) (188 17) (189 285) (190 36) (191 11) (198 8) (199 9) (201 37) (202 7) (203 396) (204 57) (205 20) (208 9) (217 158) (218 28) (219 483) (220 77) (221 14) (228 5) (229 11) (230 8) (232 175) (233 40) (234 5) (245 31) (246 7) (249 13) (251 6) (255 6) (257 21) (263 7) (264 22) (265 5) (279 55) (280 11) (282 9) (298 6) (335 464) (336 122) (337 31) (338 5) (350 329) (351 88) (352 23) |
| 127 | 2076,3 | 12,8447 | 47 | (59 33) (73 999) (74 88) (75 247) (103 20) (117 55) (128 15) (129 80) (130 21) (131 52) (133 64) (143 107) (144 20) (145 33) (147 228) (148 33) (149 36) (157 14) (171 24) (172 11) (189 24) (190 11) (191 44) (192 11) (193 19) (204 52) (205 25) (217 604) (218 108) (219 38) (221 12) (231 30) (233 11) (245 8) (257 25) (260 11) (269 12) (285 21) (298 10) (304 4) (305 61) (306 18) (318 11) (331 13) (332 10) (359 11) (360 5) |
| 128 | 2097,5 | 12,9731 | 107 | (51 19) (53 136) (54 330) (55 999) (56 299) (61 13) (65 64) (66 25) (67 467) (68 159) (69 646) (70 293) (73 215) (74 24) (77 26) (78 18) (79 136) (80 121) (81 330) (82 286) (83 418) (84 225) (89 27) (91 38) (93 85) (94 129) (95 141) (96 232) (97 293) (98 108) (103 15) (107 40) (108 111) (109 54) (110 146) (111 74) (112 91) (116 9) (117 51) (118 11) (119 31) (120 43) (121 17) (122 509) (123 137) (124 79) (125 21) (129 20) (133 33) (134 34) (135 16) (136 390) (137 98) (138 66) (140 11) (147 46) (148 31) (149 16) (150 144) (151 36) (152 53) (153 15) (155 12) (161 12) (162 16) (163 9) (164 78) (165 22) (166 29) (178 74) (179 17) (180 30) (191 13) (192 66) (193 10) (194 14) (195 6) (199 4) (205 7) (206 68) (207 16) (208 18) (217 59) (218 13) (219 6) (220 91) (221 24) (222 19) (231 4) (234 56) (235 14) (246 9) (248 12) (257 6) (258 3) (262 10) (263 32) (264 6) (271 3) (305 6) (306 4) (311 6) (318 4) (319 5) (325 13) (326 5) (328 7) |
| 129 | 2101 | 12,9944 | 43 | (53 35) (55 368) (56 192) (57 999) (58 42) (68 38) (69 210) (70 142) (71 644) (72 35) (84 68) (85 411) (86 26) (97 94) (98 45) (99 107) (100 8) (111 42) (112 33) (113 60) (114 4) (125 19) (126 16) (127 37) (128 3) (139 6) (140 13) (141 25) (154 13) (155 18) (168 8) (169 12) (182 6) (183 9) (196 4) (197 7) (211 5) (224 3) (225 5) (239 4) (253 2) (259 1) (296 6) |
| 130 | 2105,6 | 13,0223 | 61 | (53 99) (54 375) (55 999) (56 265) (65 45) (66 28) (67 360) (68 132) (69 759) (70 276) (79 91) (80 98) (81 198) (82 285) (83 378) (84 153) (93 42) (94 120) (95 93) (96 244) (97 275) (98 57) (107 34) (108 104) (109 59) (110 156) (111 74) (112 62) (122 565) (123 137) (124 111) (125 27) (134 22) (136 404) (137 110) (138 78) (139 14) (148 18) (150 163) (151 38) (152 59) (164 109) (165 20) (166 37) (178 74) (179 16) (180 28) (192 61) (193 16) (194 17) (206 72) (207 11) (220 84) (234 70) (235 16) (248 17) (262 10) (263 31) (379 19) (380 7) (394 6) |
| 131 | 2117,9 | 13,0971 | 57 | (53 62) (54 211) (55 606) (56 240) (57 999) (67 77) (68 78) (69 258) (70 445) (71 282) (79 77) (80 36) (81 69) (82 447) (83 355) (84 150) (85 116) (93 70) (94 38) (96 563) (97 769) (98 127) (99 65) (107 46) (108 28) (110 590) (111 269) (112 96) (113 37) (124 349) (125 89) (126 73) (127 26) (138 177) (139 34) (140 61) (152 118) (153 24) (154 42) (166 95) (167 16) (168 27) (180 88) (181 20) (182 27) (194 107) (195 25) (196 16) (208 128) (209 28) (210 13) (222 140) (223 32) (224 20) (236 106) (237 20) (250 16) |
| 132 | 2118,1 | 13,0984 | 56 | (53 56) (54 185) (55 604) (56 240) (57 999) (67 68) (68 68) (69 278) (70 412) (71 274) (72 34) (79 71) (80 28) (81 70) (82 388) (83 322) (84 127) (85 118) (93 69) (95 42) (96 477) (97 680) (98 117) (99 62) (107 39) (110 503) (111 240) (112 86) (113 33) (121 16) (124 293) (125 76) (126 66) (138 150) (139 29) (140 51) (152 98) (153 17) (154 35) (166 75) (167 15) (168 24) (180 74) (181 17) (182 20) (194 88) (195 21) (196 12) (208 102) (209 24) (210 10) (222 111) (223 27) (224 16) (236 87) (237 17) |
| 133 | 2119,8 | 13,1085 | 37 | (61 63) (72 37) (73 864) (74 71) (75 828) (76 47) (116 43) (117 999) (118 96) (119 41) (129 437) (130 46) (131 94) (132 319) (133 49) (135 17) (143 22) (145 241) (146 28) (159 25) (185 16) (187 14) (201 42) (227 11) (241 12) (257 27) (283 26) (285 16) (297 8) (299 24) (313 15) (327 395) (328 102) (329 27) (330 6) (342 33) (343 11) |
| 134 | 2134,1 | 13,1953 | 102 | (53 41) (54 116) (55 516) (56 83) (58 21) (59 28) (60 11) (61 67) (62 6) (65 20) (66 11) (67 232) (68 119) (69 243) (70 41) (72 38) (73 999) (74 53) (75 959) (76 73) (77 56) (79 60) (80 32) (81 203) (82 129) (83 108) (84 111) (89 29) (91 24) (93 45) (94 23) (95 156) (96 171) (97 85) (98 109) (101 16) (107 20) (108 12) (109 68) (110 64) (111 29) (112 17) (116 63) (117 537) (118 54) (119 39) (121 15) (122 8) (123 43) (124 22) (129 501) (130 64) (131 83) (132 78) (133 39) (135 15) (136 7) (137 25) (138 17) (143 23) (145 136) (146 17) (151 16) (152 17) (155 12) (157 12) (159 13) (161 6) (165 12) (166 34) (167 6) (169 9) (171 23) (172 9) (173 10) (174 16) (179 8) (180 7) (183 7) (185 34) (186 9) (187 10) (188 5) (193 6) (199 31) (200 6) (201 9) (206 6) (207 10) (208 33) (209 6) (213 7) (222 8) (227 9) (241 6) (250 27) (251 5) (297 7) (325 245) (326 65) (327 16) (340 12) |
| 135 | 2146,3 | 13,2688 | 151 | (53 19) (54 24) (55 305) (56 65) (57 208) (58 26) (59 28) (60 10) (61 55) (62 3) (63 3) (65 5) (66 3) (67 60) (68 20) (69 124) (70 35) (71 43) (72 40) (73 997) (74 103) (75 766) (76 53) (77 36) (79 16) (80 3) (81 48) (82 15) (83 66) (84 29) (85 25) (86 9) (87 8) (88 5) (89 21) (90 3) (91 7) (92 2) (93 15) (94 3) (95 40) (96 14) (97 50) (98 52) (99 19) (101 7) (105 13) (106 1) (107 9) (108 2) (109 13) (110 5) (111 21) (112 8) (115 9) (116 53) (117 999) (118 95) (119 41) (120 2) (121 8) (122 2) (123 5) (125 6) (126 2) (128 2) (129 450) (130 56) (131 100) (132 293) (133 65) (134 15) (135 7) (136 1) (137 2) (138 2) (139 2) (140 2) (142 2) (143 31) (144 3) (145 197) (146 24) (147 5) (149 3) (153 2) (154 3) (155 3) (157 8) (159 18) (160 2) (161 1) (167 3) (171 19) (172 3) (173 6) (174 2) (185 28) (186 5) (187 16) (188 5) (196 2) (199 8) (200 1) (201 39) (202 7) (203 2) (208 2) (209 4) (210 2) (213 5) (214 1) (215 6) (216 2) (223 1) (227 8) (228 1) (229 4) (230 2) (239 1) (241 8) (242 2) (243 15) (244 4) (250 1) (255 3) (257 13) (258 3) (259 1) (269 3) (271 3) (283 23) (284 5) (285 6) (286 2) (297 4) (298 2) (299 18) (300 5) (301 1) (311 1) (313 5) (314 1) (327 349) (328 90) (329 23) (330 3) (341 2) (342 26) (343 7) (344 2) |
| 136 | 2154,4 | 13,318 | 225 | (53 2) (54 4) (55 8) (56 6) (57 8) (58 20) (59 29) (60 4) (61 9) (66 2) (67 2) (68 4) (69 14) (70 14) (71 11) (72 29) (73 982) (74 89) (75 72) (76 4) (77 1) (80 1) (81 1) (82 1) (83 3) (84 42) (85 13) (86 11) (87 6) (88 1) (89 2) (92 1) (93 1) (94 1) (95 4) (96 3) (97 4) (98 13) (99 72) (100 43) (101 10) (102 5) (103 7) (104 1) (105 3) (106 1) (107 1) (108 1) (109 2) (110 6) (111 15) (112 5) (113 8) (114 6) (115 12) (116 8) (117 14) (118 4) (119 3) (120 1) (121 1) (122 1) (123 3) (124 6) (125 8) (126 5) (127 5) (128 8) (129 5) (130 25) (131 63) (132 28) (133 17) (134 4) (135 2) (136 3) (137 2) (138 6) (139 7) (140 6) (141 17) (142 11) (143 9) (144 7) (145 2) (146 3) (147 140) (148 22) (149 12) (150 3) (151 4) (152 7) (153 5) (154 4) (155 11) (156 9) (157 22) (158 48) (159 10) (160 4) (161 1) (162 1) (163 1) (164 2) (165 4) (166 16) (167 8) (168 11) (169 17) (170 5) (171 52) (172 16) (173 18) (174 3) (175 1) (176 1) (177 1) (178 2) (179 3) (180 28) (181 11) (182 11) (183 6) (184 3) (185 2) (186 1) (187 2) (188 2) (189 2) (190 18) (191 3) (192 2) (193 2) (194 3) (195 3) (196 4) (197 7) (198 12) (199 3) (200 1) (204 2) (205 2) (206 3) (207 2) (208 4) (209 2) (210 4) (211 2) (212 2) (213 1) (214 1) (215 1) (219 1) (220 1) (221 8) (222 9) (223 9) (224 4) (225 2) (226 1) (227 1) (229 1) (234 2) (235 2) (236 2) (237 17) (238 64) (239 19) (240 7) (241 2) (245 1) (248 2) (249 1) (250 2) (251 5) (252 4) (253 5) (254 4) (255 1) (256 1) (262 34) (264 93) (265 21) (266 11) (267 8) (268 4) (269 1) (276 2) (277 6) (278 43) (279 17) (280 20) (281 5) (282 2) (283 1) (292 1) (293 22) (294 26) (295 9) (296 3) (297 1) (309 2) (310 2) (311 1) (322 2) (323 3) (324 1) (325 2) (326 1) (336 26) (337 8) (338 4) (339 1) (350 4) (352 999) (353 296) (354 135) (355 28) (356 6) (357 1) (367 224) (368 71) (369 33) (370 7) (371 1) |
| 137 | 2163 | 13,3699 | 26 | (54 47) (55 412) (56 239) (57 999) (67 80) (68 36) (69 239) (70 79) (71 495) (72 40) (75 131) (82 63) (83 163) (85 389) (95 46) (96 40) (97 110) (99 127) (111 39) (113 72) (116 55) (117 67) (155 51) (169 40) (252 19) (296 36) |
| 138 | 2172,5 | 13,4278 | 81 | (59 19) (61 38) (71 48) (72 19) (73 999) (74 72) (75 51) (81 10) (84 11) (87 9) (89 58) (91 18) (101 29) (102 8) (103 169) (104 17) (105 21) (114 6) (117 84) (118 7) (119 9) (129 56) (130 11) (131 16) (133 49) (134 7) (135 5) (146 6) (147 254) (148 37) (149 93) (150 10) (151 8) (157 44) (158 7) (159 102) (160 21) (161 10) (163 8) (169 28) (170 5) (172 10) (173 25) (174 11) (175 8) (187 5) (188 4) (189 32) (190 8) (191 15) (201 6) (202 6) (203 8) (204 28) (205 147) (206 29) (207 19) (216 7) (217 103) (218 32) (219 31) (220 5) (229 19) (230 6) (231 4) (248 9) (249 14) (250 4) (262 33) (263 7) (277 5) (279 6) (291 7) (305 7) (307 6) (319 198) (320 58) (321 28) (322 6) (376 3) (466 2) |
| 139 | 2178,3 | 13,4624 | 129 | (53 6) (55 37) (56 10) (58 8) (59 24) (60 3) (61 11) (67 18) (68 12) (69 21) (70 10) (72 20) (73 999) (74 86) (75 172) (76 11) (77 8) (80 10) (81 44) (82 18) (83 15) (84 16) (87 4) (89 39) (90 3) (91 6) (95 21) (96 9) (97 13) (100 7) (101 16) (103 190) (104 19) (105 12) (109 6) (114 5) (116 7) (117 59) (118 6) (119 7) (122 4) (123 46) (124 14) (126 5) (129 45) (130 8) (131 21) (133 70) (134 8) (135 5) (142 5) (143 371) (144 48) (145 17) (146 3) (147 219) (148 33) (149 22) (150 3) (157 48) (158 8) (163 6) (169 6) (172 22) (173 12) (174 4) (175 4) (176 2) (177 4) (188 4) (189 27) (190 6) (191 16) (192 3) (201 6) (202 5) (203 5) (204 27) (205 151) (206 28) (207 17) (208 3) (213 3) (214 3) (215 2) (216 4) (217 91) (218 19) (219 9) (221 5) (229 19) (230 9) (231 5) (232 2) (235 3) (242 2) (243 3) (259 2) (260 2) (262 7) (263 4) (268 3) (277 6) (278 3) (288 2) (291 7) (292 2) (302 3) (303 2) (304 1) (305 8) (306 3) (307 6) (308 2) (318 2) (319 198) (320 57) (321 29) (322 6) (332 2) (333 2) (334 2) (335 2) (344 1) (347 1) (387 5) (388 2) (389 2) (402 2) |
| 140 | 2183,8 | 13,4957 | 18 | (57 999) (69 291) (70 169) (71 976) (84 130) (85 782) (86 55) (99 295) (113 176) (125 39) (126 58) (127 136) (141 87) (155 89) (290 29) (326 23) (341 23) (428 15) |
| 141 | 2209,6 | 13,6475 | 96 | (53 89) (54 38) (55 281) (59 34) (61 55) (62 6) (63 9) (65 101) (66 104) (67 476) (68 49) (69 105) (72 39) (73 925) (74 93) (75 886) (76 58) (77 300) (78 183) (79 999) (80 293) (81 184) (82 35) (83 37) (84 42) (89 18) (91 558) (92 128) (93 443) (94 140) (95 169) (96 23) (97 21) (101 16) (103 35) (104 21) (105 275) (106 98) (107 141) (108 210) (109 33) (115 29) (116 49) (117 172) (118 43) (119 184) (120 134) (121 73) (122 59) (123 14) (128 12) (129 165) (130 28) (131 90) (133 96) (134 54) (135 75) (136 12) (142 10) (143 32) (144 10) (145 75) (146 16) (147 90) (148 36) (149 23) (155 17) (156 8) (157 27) (159 30) (160 16) (161 59) (162 36) (163 10) (169 13) (171 41) (172 7) (173 21) (175 34) (176 14) (183 13) (185 16) (186 5) (187 13) (188 5) (189 36) (190 10) (197 8) (199 6) (201 11) (215 6) (216 5) (241 6) (252 9) (292 4) (333 7) |
| 142 | 2218 | 13,694 | 93 | (66 123) (78 122) (79 999) (80 523) (91 322) (94 301) (101 242) (102 28) (103 393) (104 50) (108 177) (122 155) (135 267) (136 184) (149 190) (150 246) (163 95) (164 107) (177 47) (178 132) (195 27) (198 8) (203 21) (211 144) (212 24) (220 92) (225 24) (226 8) (234 22) (238 10) (243 50) (244 11) (251 5) (253 25) (254 8) (261 5) (262 211) (263 43) (278 10) (285 68) (287 7) (294 347) (295 79) (296 33) (299 225) (300 51) (301 33) (302 5) (309 48) (310 15) (313 13) (315 106) (316 25) (317 18) (326 5) (327 7) (328 6) (329 25) (330 6) (335 10) (336 12) (337 411) (338 110) (343 6) (350 7) (351 8) (352 43) (357 298) (358 76) (359 36) (360 8) (370 17) (371 18) (372 7) (373 51) (374 12) (375 6) (387 25) (388 10) (389 18) (390 5) (415 4) (424 175) (425 70) (426 36) (427 9) (428 3) (439 2) (445 79) (446 25) (461 18) (462 7) (463 4) |
| 143 | 2218,7 | 13,6977 | 228 | (50 5) (51 14) (53 30) (54 117) (55 161) (58 18) (59 42) (60 7) (61 40) (62 2) (63 2) (64 2) (65 36) (66 32) (67 380) (68 118) (69 63) (71 23) (72 33) (73 999) (74 95) (75 554) (76 33) (77 97) (78 36) (79 257) (80 110) (81 277) (82 120) (83 20) (85 16) (86 6) (87 6) (88 7) (89 17) (91 81) (92 18) (93 97) (94 69) (95 166) (96 60) (97 9) (99 19) (100 5) (101 53) (102 9) (103 80) (104 11) (105 37) (106 14) (107 52) (108 40) (109 78) (110 44) (113 10) (114 5) (116 25) (119 19) (120 12) (121 58) (122 32) (123 27) (124 21) (125 12) (127 10) (128 3) (129 86) (130 10) (131 81) (133 49) (134 12) (135 57) (136 34) (137 17) (138 8) (139 8) (140 2) (141 7) (142 5) (143 6) (144 2) (147 89) (148 18) (149 44) (150 43) (151 17) (153 6) (156 5) (157 7) (158 5) (159 14) (161 7) (162 4) (163 19) (164 20) (165 4) (167 3) (170 2) (171 8) (172 4) (173 15) (174 6) (175 6) (176 3) (177 10) (178 23) (179 10) (180 6) (181 8) (182 3) (184 2) (187 12) (192 4) (193 5) (195 6) (196 2) (197 5) (198 3) (201 6) (203 7) (205 5) (206 2) (207 12) (208 4) (209 3) (211 29) (212 5) (213 5) (214 2) (215 5) (216 1) (218 11) (219 14) (220 22) (224 1) (225 6) (226 2) (227 15) (228 3) (229 5) (234 5) (237 1) (238 2) (239 3) (240 1) (241 4) (243 12) (244 3) (245 3) (251 1) (252 1) (253 6) (254 2) (256 2) (257 10) (258 2) (259 1) (261 1) (262 35) (263 8) (269 3) (277 1) (278 2) (279 1) (280 1) (283 4) (285 12) (286 3) (287 2) (292 2) (294 65) (295 17) (296 7) (297 3) (299 43) (300 10) (301 6) (302 1) (309 10) (310 3) (312 3) (313 3) (315 20) (316 5) (317 4) (329 4) (330 1) (335 2) (336 2) (337 67) (338 18) (341 5) (342 2) (343 2) (350 1) (351 1) (352 17) (353 2) (356 1) (357 54) (358 14) (359 7) (360 2) (366 1) (367 3) (370 3) (371 3) (372 1) (373 9) (374 2) (375 1) (387 5) (388 2) (389 4) (390 1) (415 1) (424 31) (425 12) (426 7) (427 2) (428 1) (445 14) (446 5) (447 3) (448 1) (461 3) (462 1) (463 1) |
| 144 | 2222 | 13,7163 | 203 | (53 36) (54 89) (55 436) (56 90) (57 114) (58 18) (59 26) (60 9) (61 56) (62 4) (63 3) (68 68) (69 185) (70 50) (71 21) (72 39) (73 999) (74 101) (75 747) (76 51) (82 79) (83 119) (84 143) (85 31) (86 10) (87 7) (88 5) (89 22) (90 3) (95 112) (96 168) (97 110) (98 125) (99 28) (100 4) (105 22) (106 6) (109 63) (110 66) (111 46) (112 25) (113 8) (114 2) (115 8) (116 60) (117 669) (118 67) (119 49) (120 7) (123 55) (124 30) (125 17) (126 6) (127 6) (128 5) (129 541) (130 71) (131 88) (132 68) (133 53) (134 20) (137 33) (138 24) (139 9) (141 7) (142 6) (143 45) (144 7) (145 163) (146 22) (147 107) (148 22) (151 22) (152 24) (153 5) (154 1) (155 26) (156 8) (157 20) (158 7) (159 16) (160 5) (161 8) (162 3) (165 11) (166 17) (167 8) (168 4) (169 18) (170 7) (171 26) (172 10) (173 12) (174 4) (175 5) (176 1) (179 5) (180 34) (181 8) (182 2) (183 24) (184 5) (185 44) (186 11) (187 10) (188 4) (189 7) (190 3) (191 27) (192 5) (193 8) (194 5) (196 1) (197 4) (199 50) (200 9) (201 13) (202 2) (203 2) (204 26) (205 12) (206 3) (207 7) (208 4) (209 2) (213 9) (214 2) (215 4) (216 1) (217 31) (218 31) (219 6) (221 18) (222 41) (223 9) (224 1) (225 2) (227 10) (228 2) (229 5) (230 2) (231 27) (232 6) (233 4) (235 7) (236 6) (237 1) (239 2) (241 10) (242 2) (244 1) (245 2) (246 4) (247 1) (249 2) (255 6) (256 1) (257 6) (258 3) (259 1) (264 32) (265 10) (266 1) (267 2) (269 3) (271 3) (272 4) (273 1) (277 1) (279 1) (281 2) (283 2) (286 2) (291 2) (293 1) (305 32) (306 10) (307 6) (308 1) (311 5) (312 1) (318 3) (319 112) (320 32) (321 17) (322 3) (323 2) (325 1) (331 2) (332 11) (333 5) (334 2) (339 216) (340 57) (341 15) (342 2) (354 13) (355 4) (367 1) (381 1) (382 1) (393 1) (407 1) |
| 145 | 2231,1 | 13,7669 | 223 | (50 1) (51 5) (52 3) (53 55) (54 121) (55 636) (56 130) (57 81) (58 24) (59 31) (60 13) (61 71) (62 5) (63 4) (65 23) (66 19) (67 275) (68 100) (69 289) (70 58) (71 26) (73 999) (75 976) (76 70) (77 63) (78 9) (79 81) (80 33) (81 221) (82 116) (83 144) (84 125) (85 38) (86 14) (87 9) (88 7) (89 25) (90 3) (91 30) (92 9) (93 56) (94 30) (95 175) (96 197) (97 128) (98 124) (99 34) (100 5) (101 12) (102 2) (103 5) (104 1) (105 27) (106 6) (107 34) (108 21) (109 99) (110 84) (111 61) (112 29) (113 8) (114 2) (117 606) (118 59) (119 50) (120 10) (121 41) (122 14) (123 60) (124 40) (125 25) (126 8) (127 6) (129 617) (130 78) (131 130) (132 84) (133 57) (134 26) (135 30) (136 8) (137 39) (138 33) (139 14) (140 4) (141 6) (143 44) (145 179) (146 24) (147 24) (148 15) (149 15) (150 6) (151 28) (152 37) (153 10) (155 22) (156 7) (157 21) (158 6) (159 19) (160 3) (161 10) (162 5) (163 7) (164 5) (165 16) (166 25) (167 11) (168 5) (169 19) (170 7) (171 38) (172 12) (173 14) (174 4) (175 9) (176 2) (177 3) (178 2) (179 10) (180 37) (181 11) (182 2) (183 26) (185 62) (186 14) (187 14) (188 8) (189 5) (190 2) (191 3) (192 1) (193 9) (194 7) (195 3) (196 1) (197 5) (198 1) (199 63) (200 12) (201 17) (202 3) (203 3) (204 1) (205 1) (207 9) (208 5) (209 3) (211 5) (212 1) (213 14) (214 3) (215 5) (216 1) (217 2) (220 8) (221 14) (222 41) (223 8) (225 3) (226 1) (227 12) (228 3) (229 4) (230 1) (235 9) (236 8) (237 1) (239 3) (241 13) (242 3) (243 4) (244 1) (245 1) (246 4) (247 1) (249 2) (253 2) (254 1) (255 12) (256 3) (257 8) (258 3) (259 1) (263 1) (264 43) (265 9) (266 1) (267 3) (268 1) (269 7) (270 2) (271 4) (272 5) (273 1) (281 3) (282 1) (283 4) (284 1) (286 3) (287 1) (295 8) (296 2) (297 3) (298 1) (299 3) (300 1) (309 2) (310 1) (311 8) (312 2) (313 1) (321 1) (323 3) (325 1) (339 292) (340 77) (341 20) (342 3) (354 16) (355 5) (356 1) |
| 146 | 2235,8 | 13,7927 | 170 | (52 14) (53 79) (54 199) (55 324) (59 43) (60 12) (61 66) (62 5) (63 8) (64 3) (65 58) (66 43) (67 648) (68 188) (69 147) (72 40) (73 881) (74 100) (75 999) (76 65) (77 131) (78 34) (79 351) (80 122) (81 438) (82 214) (83 73) (87 10) (88 6) (89 12) (90 5) (91 86) (92 19) (93 114) (94 78) (95 244) (96 153) (97 40) (100 6) (101 14) (104 6) (105 38) (106 14) (107 60) (108 47) (109 110) (110 67) (115 11) (116 51) (117 167) (118 26) (119 34) (120 13) (121 67) (122 33) (123 50) (124 33) (125 15) (128 5) (129 225) (130 28) (131 68) (132 13) (133 30) (134 9) (135 64) (136 40) (137 25) (138 16) (139 7) (143 18) (144 10) (145 29) (146 9) (147 27) (148 8) (149 47) (150 54) (151 18) (152 8) (153 7) (155 10) (156 8) (157 14) (158 4) (159 18) (160 4) (161 7) (162 2) (163 22) (164 22) (165 6) (166 7) (168 11) (169 7) (171 11) (173 18) (174 10) (175 7) (176 3) (177 11) (178 30) (179 10) (181 8) (183 9) (184 3) (185 9) (187 15) (188 6) (190 2) (191 9) (192 6) (195 4) (197 5) (198 4) (199 4) (200 5) (201 11) (202 4) (203 3) (204 4) (205 3) (206 5) (207 3) (208 4) (212 4) (213 6) (214 2) (215 7) (219 6) (220 20) (221 5) (225 4) (226 2) (227 6) (228 2) (229 7) (233 4) (234 7) (240 2) (246 2) (247 1) (251 1) (253 3) (254 2) (255 5) (256 3) (257 5) (261 4) (262 50) (263 10) (270 1) (271 3) (275 1) (281 2) (285 3) (296 1) (298 1) (300 3) (310 4) (312 2) (313 2) (325 4) (337 109) (338 28) (352 8) (357 5) (358 3) (359 2) (367 3) |
| 147 | 2236,2 | 13,7951 | 118 | (53 81) (54 258) (55 305) (58 21) (59 32) (60 11) (61 62) (62 4) (63 6) (64 3) (65 83) (66 59) (67 999) (68 251) (69 131) (72 46) (73 680) (74 80) (75 899) (76 60) (77 158) (78 39) (79 408) (80 175) (81 631) (82 281) (83 81) (89 15) (91 98) (92 23) (93 154) (94 109) (95 323) (96 151) (97 54) (100 13) (101 18) (105 40) (106 17) (107 87) (108 73) (109 145) (110 71) (111 42) (116 42) (118 18) (119 27) (120 15) (121 101) (122 51) (123 60) (124 37) (125 23) (128 8) (129 166) (130 21) (131 72) (133 23) (134 10) (135 98) (136 65) (137 22) (138 15) (139 10) (143 13) (144 8) (147 17) (149 67) (150 90) (151 20) (152 11) (153 11) (155 9) (156 7) (157 13) (158 5) (159 17) (161 5) (163 34) (164 36) (165 7) (168 17) (169 9) (171 13) (173 23) (174 13) (175 7) (176 4) (177 19) (178 51) (179 10) (181 9) (183 11) (184 5) (187 17) (191 7) (192 7) (197 6) (201 8) (206 6) (215 9) (220 29) (221 7) (227 5) (229 8) (233 5) (234 12) (255 5) (256 3) (261 5) (262 81) (263 14) (310 5) (325 6) (326 2) (337 170) (338 41) (352 10) |
| 148 | 2238,1 | 13,8055 | 20 | (56 166) (57 999) (58 43) (70 122) (71 678) (84 53) (85 518) (97 113) (99 128) (111 96) (113 88) (125 36) (127 38) (140 52) (141 37) (154 49) (168 35) (182 32) (210 17) (303 6) |
| 149 | 2247,7 | 13,859 | 199 | (51 1) (53 19) (54 21) (55 294) (56 64) (57 195) (58 27) (59 28) (60 10) (61 53) (62 3) (63 2) (65 4) (67 59) (68 17) (69 127) (70 29) (71 44) (73 927) (74 103) (75 711) (76 49) (77 36) (78 2) (79 18) (80 4) (81 50) (82 10) (83 65) (84 23) (85 29) (86 10) (87 8) (88 6) (89 21) (90 2) (91 7) (92 1) (93 19) (95 45) (96 7) (97 53) (98 39) (99 23) (101 11) (102 2) (103 4) (105 16) (106 1) (107 12) (108 1) (109 19) (110 3) (111 32) (112 13) (113 6) (114 1) (117 999) (118 94) (119 42) (120 2) (121 12) (122 1) (123 7) (124 1) (125 9) (126 4) (127 6) (129 515) (130 64) (132 279) (133 74) (134 16) (135 11) (136 1) (137 3) (138 1) (139 4) (140 3) (141 4) (143 47) (145 233) (146 30) (147 20) (148 2) (149 6) (150 1) (151 1) (152 1) (153 5) (154 8) (155 5) (157 12) (159 28) (160 4) (161 2) (163 2) (165 1) (167 4) (168 4) (169 2) (171 28) (172 4) (173 9) (174 5) (175 1) (177 1) (179 1) (181 5) (182 1) (183 1) (185 44) (186 6) (187 24) (188 8) (189 2) (190 1) (191 3) (193 1) (195 2) (196 1) (197 1) (199 11) (201 64) (203 4) (204 1) (205 1) (207 1) (209 2) (211 6) (213 9) (214 1) (215 12) (216 2) (217 2) (219 1) (223 6) (224 1) (225 1) (227 16) (228 3) (229 6) (230 3) (231 1) (237 2) (239 1) (241 14) (242 3) (243 16) (244 5) (245 1) (247 1) (255 10) (256 2) (257 25) (258 6) (259 2) (261 1) (265 1) (266 1) (267 1) (269 4) (270 2) (271 12) (272 3) (273 1) (283 5) (284 1) (285 5) (286 1) (297 30) (298 8) (299 16) (300 4) (301 1) (311 5) (313 27) (314 7) (315 5) (316 1) (317 1) (318 1) (325 1) (327 5) (328 1) (341 430) (342 114) (343 31) (344 5) (345 1) (356 36) (357 10) (358 3) (359 1) (369 1) (398 1) (407 1) (422 2) (423 1) |
| 150 | 2250,3 | 13,8734 | 35 | (100 37) (116 699) (128 218) (131 999) (142 25) (144 199) (156 6) (158 22) (166 2) (170 17) (180 2) (184 14) (194 3) (198 11) (200 25) (208 2) (212 6) (222 1) (226 9) (236 2) (240 6) (252 1) (254 3) (264 2) (268 4) (282 3) (284 6) (296 2) (310 6) (312 235) (326 6) (339 12) (340 5) (352 2) (361 1) |
| 151 | 2260,3 | 13,9291 | 23 | (73 999) (103 298) (117 117) (129 83) (133 65) (147 508) (157 67) (174 261) (175 55) (176 28) (189 67) (205 306) (206 52) (207 29) (217 203) (229 23) (259 21) (308 15) (319 297) (320 94) (321 45) (331 40) (421 20) |
| 152 | 2264 | 13,9496 | 51 | (54 18) (55 258) (56 195) (57 999) (58 45) (68 24) (69 168) (70 76) (71 605) (72 35) (82 20) (83 103) (84 41) (85 422) (86 20) (97 81) (98 21) (99 141) (110 8) (111 41) (112 15) (113 70) (125 12) (126 9) (127 47) (139 8) (140 6) (141 38) (155 29) (168 4) (169 25) (179 11) (182 4) (183 21) (191 12) (197 15) (204 12) (211 11) (218 17) (219 5) (225 12) (231 24) (232 4) (239 4) (280 14) (281 32) (282 7) (305 59) (306 18) (309 5) (324 2) |
| 153 | 2271,8 | 13,9928 | 218 | (53 3) (54 4) (58 14) (59 131) (60 11) (61 12) (62 1) (68 2) (70 7) (72 29) (73 999) (74 85) (75 89) (76 5) (84 3) (86 35) (87 12) (88 4) (89 9) (90 1) (98 2) (99 5) (100 143) (101 89) (102 16) (103 97) (104 8) (105 4) (111 5) (112 4) (113 17) (114 84) (115 51) (116 35) (117 20) (118 3) (119 7) (121 4) (123 1) (125 1) (126 1) (127 2) (129 75) (130 33) (131 54) (132 8) (133 75) (134 10) (135 19) (136 2) (137 6) (138 1) (139 5) (140 1) (141 1) (142 4) (143 2) (144 2) (145 3) (146 1) (147 131) (148 20) (149 17) (150 2) (151 5) (152 1) (153 2) (154 1) (156 2) (157 6) (158 4) (159 2) (160 1) (161 1) (163 8) (164 1) (165 2) (166 1) (167 2) (168 1) (169 2) (170 2) (172 163) (173 31) (174 173) (175 32) (176 14) (177 4) (179 3) (180 1) (181 10) (182 1) (183 4) (184 1) (185 31) (186 6) (187 22) (188 229) (189 49) (190 20) (191 16) (192 3) (193 14) (194 3) (195 16) (196 3) (197 4) (198 1) (203 9) (204 3) (205 7) (206 2) (207 25) (208 5) (209 4) (210 3) (211 85) (212 12) (213 10) (214 1) (217 7) (218 30) (219 13) (220 4) (221 2) (223 1) (225 17) (226 5) (227 16) (228 3) (229 1) (237 1) (238 1) (239 1) (240 2) (241 27) (242 5) (243 14) (244 2) (245 1) (253 6) (254 2) (255 4) (256 18) (257 16) (258 5) (259 11) (260 2) (267 1) (268 1) (269 6) (270 2) (271 1) (273 1) (281 1) (283 8) (284 3) (285 20) (286 4) (287 3) (299 243) (300 60) (301 34) (302 6) (303 2) (310 1) (311 1) (312 1) (313 6) (314 10) (315 76) (316 20) (317 10) (318 2) (326 2) (327 6) (328 16) (329 30) (330 9) (331 4) (332 1) (340 1) (341 18) (342 15) (343 6) (344 2) (345 1) (355 1) (357 166) (358 45) (359 23) (360 5) (361 1) (370 21) (371 8) (372 4) (373 13) (374 4) (375 2) (376 1) (387 22) (388 7) (389 10) (390 3) (391 2) (414 14) (415 7) (416 3) (417 1) (445 43) (446 15) (447 8) (448 2) (449 1) (461 29) (462 10) (463 6) (464 1) |
| 154 | 2291,7 | 14,1032 | 126 | (53 81) (54 83) (55 449) (56 172) (57 140) (58 233) (59 30) (60 15) (61 65) (62 4) (63 10) (65 102) (66 110) (67 563) (68 71) (69 149) (70 61) (71 99) (72 101) (73 942) (74 109) (75 999) (76 75) (77 312) (78 188) (79 821) (80 470) (81 297) (82 66) (83 64) (84 35) (85 26) (86 16) (89 14) (90 8) (91 581) (92 148) (93 393) (94 140) (95 93) (96 39) (97 46) (98 14) (99 12) (104 33) (105 237) (106 250) (107 145) (108 30) (109 45) (110 21) (111 18) (115 42) (117 309) (118 51) (119 151) (120 114) (121 91) (122 38) (123 29) (124 11) (128 24) (129 199) (130 39) (131 194) (132 62) (133 137) (134 47) (135 43) (136 115) (137 16) (141 18) (142 14) (143 25) (144 28) (145 76) (146 35) (147 57) (148 27) (149 32) (150 34) (151 7) (152 8) (154 8) (155 20) (157 34) (158 12) (159 31) (160 18) (162 24) (163 34) (164 7) (168 6) (169 26) (170 9) (171 38) (172 7) (173 26) (174 15) (175 50) (176 19) (181 6) (183 18) (184 5) (185 11) (187 23) (188 11) (189 28) (190 7) (197 12) (200 6) (201 13) (202 6) (203 19) (211 7) (213 7) (215 8) (216 7) (224 4) (230 8) (231 5) (235 4) (238 22) (278 4) (284 17) (347 5) |
| 155 | 2300,8 | 14,1541 | 60 | (53 14) (54 21) (55 272) (56 147) (57 999) (58 39) (59 6) (65 8) (66 3) (67 37) (68 26) (69 150) (70 109) (71 696) (72 39) (81 14) (82 26) (83 96) (84 55) (85 449) (86 27) (91 6) (96 13) (97 67) (98 30) (99 115) (100 7) (111 36) (112 21) (113 64) (114 5) (125 15) (126 20) (127 40) (128 3) (139 4) (141 32) (142 3) (154 11) (155 21) (156 2) (168 7) (169 17) (170 3) (182 6) (183 11) (185 2) (196 4) (197 8) (210 4) (211 8) (224 4) (225 6) (238 3) (239 4) (253 3) (267 3) (281 2) (324 5) (325 1) |
| 156 | 982,4 | 4,4188 | 87 | (53 6) (54 4) (55 19) (56 25) (58 330) (59 83) (60 76) (61 27) (62 14) (63 46) (64 8) (65 2) (69 245) (72 46) (73 630) (74 68) (75 134) (76 43) (77 999) (78 120) (79 45) (80 6) (81 9) (86 9) (87 5) (88 21) (89 7) (90 57) (91 38) (92 10) (93 4) (96 2) (97 18) (100 45) (101 5) (102 5) (103 154) (104 25) (105 8) (106 2) (107 36) (108 6) (109 3) (110 42) (111 4) (112 13) (114 1) (116 17) (118 37) (119 4) (120 79) (121 8) (122 3) (126 2) (130 34) (131 4) (132 4) (134 648) (135 63) (136 28) (137 4) (138 2) (140 100) (141 4) (143 87) (144 7) (145 2) (148 3) (160 14) (175 6) (183 1) (184 572) (185 61) (186 24) (187 1) (198 7) (199 2) (207 3) (209 1) (214 260) (215 30) (216 11) (228 2) (229 83) (230 11) (231 3) (250 1) |
| 157 | 2318,4 | 14,2519 | 74 | (51 41) (53 62) (55 300) (58 711) (59 66) (61 52) (65 56) (66 57) (67 236) (68 63) (69 96) (72 170) (73 999) (74 149) (75 672) (76 54) (77 177) (78 86) (79 449) (80 137) (81 147) (83 65) (84 39) (87 34) (91 319) (92 72) (93 194) (94 136) (95 95) (96 43) (104 24) (105 190) (106 119) (107 75) (108 27) (109 45) (111 32) (115 33) (117 554) (118 73) (119 130) (120 50) (121 37) (123 23) (125 19) (129 230) (130 41) (131 112) (132 114) (133 81) (134 42) (135 38) (143 27) (145 126) (146 22) (160 17) (161 35) (171 36) (173 32) (187 19) (201 20) (207 21) (221 22) (280 128) (281 34) (282 13) (292 14) (296 225) (297 59) (298 20) (351 23) (355 96) (356 27) (370 8) |
| 158 | 2325,8 | 14,2926 | 155 | (53 43) (54 134) (55 572) (56 127) (57 138) (58 16) (59 22) (60 10) (61 62) (62 5) (65 22) (66 18) (67 318) (68 114) (69 273) (70 63) (71 46) (72 43) (73 999) (74 104) (75 940) (76 64) (77 69) (78 8) (81 257) (82 133) (83 154) (84 122) (85 43) (86 10) (87 7) (88 6) (89 25) (91 35) (92 8) (93 57) (94 37) (95 172) (96 178) (97 120) (98 96) (99 29) (101 9) (105 23) (106 5) (107 30) (108 22) (109 84) (110 68) (111 43) (112 19) (113 8) (115 5) (116 53) (117 495) (118 48) (119 35) (120 6) (121 34) (122 14) (123 47) (124 30) (125 18) (126 5) (129 465) (130 56) (131 77) (132 64) (133 38) (134 14) (135 26) (137 27) (138 21) (139 9) (142 4) (143 30) (144 4) (145 135) (146 17) (148 6) (149 14) (151 18) (152 21) (153 5) (155 14) (156 4) (157 11) (158 4) (159 12) (161 6) (162 2) (165 9) (166 12) (167 4) (169 10) (170 5) (171 23) (172 8) (173 12) (175 5) (177 4) (179 6) (180 10) (181 5) (183 17) (184 3) (185 41) (186 8) (187 10) (188 4) (193 6) (194 19) (195 6) (197 7) (199 38) (200 6) (201 12) (208 5) (211 4) (213 9) (215 3) (221 4) (222 5) (227 10) (229 4) (234 8) (235 12) (236 27) (237 5) (239 2) (241 8) (243 3) (249 7) (250 6) (255 9) (256 2) (257 3) (260 3) (269 6) (271 5) (277 4) (278 26) (279 6) (283 2) (285 3) (286 3) (295 2) (309 5) (325 5) (353 183) (354 50) (355 13) (356 2) (368 11) (369 3) |
| 159 | 2325,9 | 14,2934 | 159 | (53 44) (54 136) (55 573) (56 127) (57 140) (58 16) (59 22) (60 10) (61 62) (62 5) (65 23) (66 19) (67 327) (68 116) (69 272) (70 63) (71 47) (72 43) (73 999) (74 104) (75 944) (76 64) (77 70) (78 9) (79 113) (80 53) (81 263) (82 135) (83 154) (84 121) (85 44) (86 10) (87 6) (88 6) (89 25) (91 36) (92 8) (93 59) (94 39) (95 175) (96 179) (97 120) (98 95) (99 30) (101 9) (105 24) (106 5) (107 31) (108 23) (109 85) (110 68) (111 43) (112 18) (113 8) (115 5) (116 52) (117 489) (118 48) (119 35) (120 7) (121 35) (122 15) (123 48) (124 30) (125 18) (126 5) (129 461) (130 55) (131 76) (132 64) (133 38) (134 14) (135 27) (136 14) (137 27) (138 21) (139 9) (142 4) (143 30) (144 4) (145 133) (146 17) (148 6) (149 14) (151 18) (152 21) (153 5) (155 13) (156 4) (157 11) (158 4) (159 12) (161 6) (162 2) (163 8) (164 5) (165 9) (166 12) (167 4) (169 10) (170 5) (171 23) (172 8) (173 12) (174 5) (175 5) (177 4) (178 4) (179 6) (180 10) (181 5) (183 17) (184 3) (185 41) (186 7) (187 10) (188 4) (193 6) (194 19) (195 6) (197 7) (199 37) (200 6) (201 12) (208 5) (211 4) (213 9) (215 3) (221 4) (222 5) (227 10) (229 4) (234 8) (235 11) (236 26) (237 5) (239 2) (241 7) (243 3) (249 7) (250 6) (255 9) (257 3) (260 2) (269 6) (271 5) (277 4) (278 25) (279 6) (285 3) (286 3) (295 2) (309 5) (325 5) (352 10) (354 48) (355 13) (368 10) (369 3) |
| 160 | 2326,5 | 14,2965 | 136 | (53 46) (54 147) (55 576) (56 127) (57 144) (58 15) (59 22) (60 11) (61 63) (62 5) (65 26) (66 21) (67 360) (68 126) (69 271) (70 61) (71 53) (72 44) (73 999) (74 103) (75 958) (76 63) (77 77) (78 10) (79 133) (80 63) (81 289) (82 143) (83 152) (84 116) (85 46) (86 11) (88 6) (89 25) (91 40) (92 8) (93 66) (94 44) (95 186) (96 180) (97 118) (98 90) (99 30) (105 25) (106 6) (107 34) (108 26) (109 88) (110 67) (111 42) (113 8) (115 5) (116 51) (117 468) (118 46) (119 34) (120 7) (121 38) (122 17) (123 50) (124 30) (125 17) (129 443) (130 53) (131 75) (132 61) (133 35) (134 14) (135 30) (136 17) (137 27) (138 20) (139 9) (142 4) (143 29) (144 4) (145 125) (146 16) (149 16) (150 18) (151 18) (152 20) (153 5) (155 13) (156 4) (157 10) (159 11) (161 6) (163 9) (164 6) (165 9) (166 11) (167 4) (169 10) (170 5) (171 22) (172 7) (173 12) (174 6) (175 5) (177 4) (178 5) (179 6) (180 9) (181 5) (183 16) (184 3) (185 38) (186 7) (187 9) (188 4) (192 9) (193 6) (194 17) (195 5) (197 7) (199 34) (200 6) (201 12) (211 4) (213 8) (227 9) (229 4) (234 9) (235 11) (239 2) (241 7) (243 3) (249 6) (276 17) (277 4) (279 5) (309 4) (351 38) (352 12) (366 2) |
| 161 | 2333,6 | 14,3363 | 50 | (53 49) (54 104) (55 519) (61 66) (67 219) (68 82) (69 297) (73 908) (74 114) (75 999) (76 65) (77 52) (81 209) (82 105) (83 114) (84 112) (95 148) (96 161) (98 97) (107 25) (109 83) (110 47) (116 50) (117 584) (118 61) (119 47) (121 26) (123 29) (124 22) (129 503) (130 62) (131 68) (132 97) (133 35) (137 35) (145 161) (146 39) (171 28) (180 17) (185 43) (194 20) (199 42) (201 18) (235 14) (236 24) (278 36) (353 227) (354 58) (355 21) (368 13) |
| 162 | 2336,7 | 14,3535 | 23 | (56 142) (57 999) (58 51) (70 78) (71 644) (83 76) (85 456) (86 35) (97 103) (99 141) (111 70) (113 70) (125 35) (127 58) (140 22) (141 29) (154 78) (155 35) (168 15) (174 15) (210 23) (211 15) (225 12) |
| 163 | 2346,5 | 14,408 | 73 | (56 27) (57 220) (58 14) (59 32) (70 25) (71 45) (72 17) (73 999) (74 87) (89 14) (98 48) (99 20) (101 59) (103 215) (104 21) (105 14) (116 16) (129 136) (130 17) (131 34) (133 61) (135 5) (143 17) (147 261) (148 44) (149 33) (150 4) (157 8) (161 8) (169 17) (175 8) (177 8) (189 39) (190 8) (191 59) (192 10) (193 5) (203 17) (204 813) (205 159) (206 74) (207 13) (217 172) (218 47) (219 48) (220 9) (221 8) (231 6) (233 3) (243 16) (244 4) (245 4) (249 4) (266 4) (267 4) (271 7) (291 9) (293 4) (303 2) (305 13) (306 5) (319 7) (331 4) (333 3) (337 76) (338 22) (339 10) (340 3) (361 17) (362 6) (363 3) (379 2) (451 2) |
| 164 | 2358,3 | 14,4734 | 27 | (55 285) (57 873) (67 54) (68 39) (69 269) (70 454) (71 999) (72 39) (82 39) (83 131) (84 92) (85 585) (86 35) (97 71) (98 35) (99 210) (110 31) (111 83) (112 42) (113 126) (127 88) (141 58) (155 42) (169 32) (183 31) (294 16) (295 32) |
| 165 | 2383,6 | 14,6137 | 246 | (51 18) (52 12) (53 80) (54 76) (55 365) (56 32) (57 56) (58 20) (59 27) (60 12) (61 61) (62 5) (63 9) (65 114) (66 122) (67 575) (68 65) (69 111) (70 16) (71 28) (72 63) (73 999) (74 112) (75 899) (76 70) (77 323) (78 218) (79 832) (80 486) (81 279) (82 54) (83 41) (84 19) (85 27) (86 16) (87 8) (88 8) (89 20) (91 670) (92 173) (93 423) (94 167) (95 156) (96 62) (97 29) (98 8) (99 11) (100 2) (101 14) (102 5) (103 41) (104 50) (105 318) (106 294) (107 174) (108 44) (109 55) (110 17) (111 11) (112 3) (113 5) (114 1) (115 48) (116 64) (117 491) (118 81) (119 230) (120 179) (121 146) (122 33) (123 26) (124 10) (125 6) (126 1) (127 7) (129 298) (130 64) (131 197) (132 79) (133 223) (134 74) (135 85) (136 33) (137 16) (138 4) (139 5) (140 1) (141 28) (142 19) (143 43) (145 127) (146 68) (147 128) (148 50) (149 53) (150 126) (151 27) (152 4) (153 6) (155 27) (156 10) (157 67) (158 15) (159 64) (160 37) (161 67) (162 31) (163 26) (164 38) (165 12) (166 4) (167 7) (168 4) (169 45) (170 14) (171 65) (172 12) (173 52) (174 23) (175 92) (176 34) (177 43) (178 10) (179 5) (180 1) (181 7) (182 3) (183 38) (184 8) (185 22) (186 5) (187 42) (188 18) (189 32) (190 22) (191 10) (192 2) (193 3) (194 3) (195 6) (196 2) (197 28) (198 6) (199 12) (201 33) (202 11) (203 44) (204 24) (205 7) (206 7) (207 4) (208 1) (209 4) (210 2) (211 13) (212 4) (213 7) (214 2) (215 18) (216 5) (217 29) (218 9) (219 4) (220 1) (221 4) (222 1) (223 4) (224 2) (225 5) (226 1) (227 3) (228 1) (229 6) (230 4) (231 8) (232 3) (233 1) (235 7) (236 2) (237 3) (238 17) (239 7) (240 2) (241 3) (242 3) (243 5) (244 13) (245 6) (246 1) (247 1) (249 6) (250 3) (251 5) (252 3) (253 1) (257 2) (258 4) (259 2) (261 1) (263 3) (264 2) (265 8) (266 2) (267 3) (268 2) (269 9) (270 2) (271 1) (275 1) (277 6) (278 8) (279 5) (280 1) (281 1) (285 1) (286 5) (287 1) (289 1) (291 5) (292 3) (293 2) (294 1) (295 1) (296 1) (303 1) (305 5) (306 3) (307 1) (308 3) (309 1) (319 3) (320 1) (321 1) (322 1) (333 2) (334 1) (335 1) (337 1) (343 1) (347 1) (348 1) (361 11) (362 3) (363 1) (376 5) (377 1) |
| 166 | 2384,3 | 14,6178 | 247 | (50 4) (51 19) (52 12) (53 81) (54 77) (55 372) (56 32) (57 57) (58 21) (59 28) (60 12) (61 62) (62 5) (63 9) (64 2) (65 116) (66 123) (67 581) (68 64) (69 112) (70 16) (71 28) (72 63) (73 999) (74 110) (75 898) (76 69) (77 322) (78 217) (79 830) (80 484) (81 276) (82 53) (83 40) (84 19) (85 26) (86 16) (87 8) (88 7) (89 20) (91 663) (92 170) (93 419) (94 164) (95 154) (96 61) (97 28) (98 8) (99 11) (100 2) (101 14) (102 5) (103 40) (104 49) (105 313) (106 288) (107 171) (108 43) (109 53) (110 16) (111 11) (112 3) (113 4) (114 1) (115 47) (116 63) (117 480) (118 77) (119 222) (120 173) (121 142) (122 32) (123 26) (124 10) (125 6) (126 1) (127 7) (129 289) (130 60) (131 189) (132 74) (133 212) (134 70) (135 80) (136 30) (137 15) (138 3) (139 5) (140 1) (141 27) (142 18) (143 41) (145 118) (146 63) (147 121) (148 46) (149 49) (150 115) (151 24) (152 4) (153 5) (155 26) (156 9) (157 62) (158 13) (159 60) (160 34) (161 62) (162 29) (163 24) (164 35) (165 11) (166 3) (167 6) (168 4) (169 41) (170 13) (171 60) (172 11) (173 47) (174 22) (175 83) (176 30) (177 38) (178 9) (179 4) (180 1) (181 7) (182 3) (183 34) (184 7) (185 20) (186 4) (187 38) (188 16) (189 29) (190 20) (191 9) (192 2) (193 3) (194 3) (195 6) (196 2) (197 25) (198 5) (199 11) (201 30) (202 10) (203 39) (204 20) (205 6) (206 6) (207 4) (208 1) (209 4) (210 1) (211 12) (212 4) (213 6) (214 2) (215 16) (216 5) (217 26) (218 8) (219 3) (220 1) (221 3) (222 1) (223 4) (224 2) (225 4) (226 1) (227 3) (228 1) (229 6) (230 4) (231 7) (232 3) (233 1) (235 6) (236 2) (237 3) (238 15) (239 6) (240 2) (241 2) (242 2) (243 4) (244 11) (245 5) (246 1) (247 1) (249 5) (250 2) (251 5) (252 2) (253 1) (257 2) (258 4) (259 2) (261 1) (263 3) (264 2) (265 7) (266 2) (267 3) (268 2) (269 7) (270 2) (271 1) (275 1) (277 5) (278 7) (279 4) (280 1) (281 1) (285 1) (286 5) (287 1) (289 1) (291 4) (292 2) (293 2) (294 1) (295 1) (303 1) (305 4) (306 2) (307 1) (308 2) (309 1) (319 3) (320 1) (321 1) (322 1) (333 1) (334 1) (335 1) (337 1) (343 1) (347 1) (348 1) (361 9) (362 3) (363 1) (376 4) (377 1) |
| 167 | 2394,3 | 14,6736 | 216 | (50 8) (51 33) (52 20) (53 112) (54 52) (55 338) (56 18) (58 21) (59 30) (60 14) (61 67) (62 7) (63 15) (65 160) (66 139) (67 630) (68 58) (69 80) (70 20) (73 978) (75 926) (76 77) (77 418) (78 236) (79 999) (80 285) (81 202) (82 42) (83 22) (84 16) (86 12) (87 7) (88 7) (89 21) (91 822) (92 183) (93 409) (94 140) (95 146) (96 20) (97 15) (98 5) (99 6) (100 1) (101 13) (102 6) (103 47) (104 57) (105 375) (106 253) (107 149) (108 142) (109 50) (110 7) (111 7) (112 1) (113 2) (115 63) (117 459) (118 87) (119 297) (120 153) (121 74) (122 29) (123 14) (124 3) (125 4) (127 8) (129 244) (130 59) (131 230) (132 74) (133 180) (134 67) (135 56) (136 13) (137 7) (138 1) (139 4) (141 29) (142 18) (143 53) (145 130) (146 59) (147 93) (148 50) (149 26) (150 6) (151 9) (152 3) (153 6) (155 29) (156 10) (157 51) (158 18) (159 76) (160 42) (161 54) (162 29) (163 11) (164 3) (165 6) (166 2) (167 9) (169 36) (170 11) (171 63) (172 15) (173 65) (174 27) (175 56) (176 17) (177 5) (178 1) (179 3) (180 1) (181 11) (182 4) (183 24) (184 8) (185 26) (186 9) (187 38) (188 17) (189 12) (190 2) (191 2) (192 1) (193 2) (194 2) (195 9) (196 3) (197 28) (198 6) (199 19) (200 7) (201 36) (202 10) (203 4) (204 3) (205 3) (206 1) (207 2) (208 1) (209 6) (210 2) (211 8) (212 2) (213 11) (215 28) (216 6) (217 1) (219 1) (221 2) (222 1) (223 7) (224 2) (225 4) (226 1) (227 6) (228 3) (229 4) (230 1) (231 1) (233 1) (235 4) (236 1) (237 6) (238 11) (239 4) (240 2) (241 5) (242 5) (243 2) (247 1) (249 3) (250 1) (251 3) (252 2) (253 1) (255 4) (256 2) (257 1) (261 1) (263 2) (264 1) (265 2) (266 1) (267 2) (275 1) (277 2) (278 4) (279 1) (289 2) (290 1) (291 2) (292 2) (293 1) (294 1) (303 1) (304 1) (305 5) (306 3) (307 1) (317 1) (318 1) (319 1) (320 1) (331 2) (332 1) (333 1) (341 1) (345 2) (346 1) (359 2) (374 1) |
| 168 | 2400,7 | 14,7085 | 97 | (51 2) (53 21) (54 32) (55 311) (56 156) (57 999) (58 40) (61 4) (63 1) (65 10) (66 9) (67 73) (68 30) (69 153) (70 99) (71 569) (72 31) (75 53) (76 4) (77 19) (78 11) (79 47) (80 32) (81 32) (82 31) (83 97) (84 51) (86 22) (91 35) (92 9) (93 26) (94 11) (95 15) (96 20) (104 2) (105 16) (106 18) (107 11) (108 3) (109 5) (110 10) (112 22) (117 28) (118 5) (119 12) (120 10) (121 8) (122 3) (123 2) (124 5) (125 17) (126 16) (128 5) (129 14) (130 2) (131 8) (132 3) (133 9) (134 3) (135 6) (136 3) (137 1) (138 3) (139 6) (140 12) (142 4) (143 2) (145 5) (149 2) (150 6) (151 1) (152 3) (153 3) (157 2) (159 3) (160 1) (161 3) (162 1) (164 2) (165 1) (166 1) (167 2) (170 2) (171 3) (173 2) (175 2) (176 1) (177 2) (181 1) (182 5) (183 12) (185 1) (189 1) (190 1) (197 8) (240 1) (342 1) |
| 169 | 2403,4 | 14,7224 | 38 | (59 93) (74 137) (88 33) (89 56) (101 225) (102 18) (103 240) (116 180) (147 615) (148 90) (149 64) (187 47) (188 25) (201 33) (203 180) (204 45) (205 154) (206 32) (207 23) (211 171) (212 20) (218 35) (219 14) (257 15) (285 59) (286 10) (314 9) (343 999) (344 265) (345 77) (346 9) (356 7) (365 10) (373 17) (375 7) (431 61) (432 20) (433 7) |
| 170 | 2410,2 | 14,7576 | 166 | (51 17) (52 19) (53 98) (54 47) (55 360) (56 22) (58 15) (59 28) (60 10) (61 66) (62 6) (63 11) (65 116) (66 115) (67 597) (68 62) (69 111) (72 38) (73 979) (74 100) (75 999) (76 76) (77 344) (78 183) (79 954) (80 315) (81 221) (82 45) (83 26) (84 14) (86 8) (87 9) (88 6) (89 21) (90 4) (91 607) (92 143) (93 431) (94 152) (95 166) (96 21) (97 18) (98 9) (101 8) (102 3) (103 21) (104 35) (105 274) (106 187) (107 155) (108 175) (109 52) (110 5) (115 33) (116 41) (117 320) (118 54) (119 225) (120 92) (121 82) (122 54) (123 16) (125 5) (128 13) (129 225) (130 39) (131 142) (132 42) (133 140) (134 56) (135 82) (136 19) (137 6) (141 14) (142 10) (143 39) (144 12) (145 90) (146 31) (147 67) (148 44) (149 33) (150 10) (151 6) (155 17) (156 7) (157 34) (158 10) (159 48) (160 23) (161 54) (162 29) (163 10) (167 5) (169 19) (170 5) (171 36) (172 7) (173 38) (174 15) (175 53) (176 15) (177 8) (178 3) (181 6) (182 2) (183 15) (184 4) (185 18) (186 6) (187 21) (188 13) (189 16) (190 11) (191 4) (195 5) (197 13) (198 3) (199 27) (200 5) (201 18) (202 4) (203 6) (204 3) (209 4) (211 4) (212 2) (213 7) (214 2) (215 16) (216 4) (217 22) (223 4) (225 5) (227 4) (228 2) (229 4) (237 3) (238 7) (239 5) (241 3) (242 2) (243 3) (249 2) (251 2) (255 2) (257 3) (263 2) (267 2) (275 1) (277 3) (278 3) (280 3) (285 2) (291 3) (292 2) (305 2) (317 2) (319 2) (320 2) (333 3) (347 2) (359 2) (361 9) (362 2) (376 3) |
| 171 | 2419,5 | 14,8051 | 222 | (51 8) (52 5) (53 73) (54 260) (55 522) (56 44) (57 57) (58 20) (59 27) (60 12) (61 65) (62 5) (63 4) (64 1) (65 61) (66 53) (67 774) (68 235) (69 213) (70 21) (71 20) (72 46) (73 999) (74 107) (75 996) (76 70) (77 137) (78 36) (79 349) (80 189) (81 553) (82 245) (83 110) (84 23) (85 26) (86 9) (87 7) (88 6) (89 26) (90 3) (91 103) (92 24) (93 153) (94 115) (95 378) (96 224) (97 98) (98 24) (99 23) (101 11) (102 3) (103 9) (104 6) (105 43) (106 16) (107 88) (108 74) (109 174) (110 107) (111 42) (112 8) (113 7) (114 1) (115 7) (116 40) (117 307) (118 35) (119 44) (120 15) (121 115) (122 55) (123 80) (124 50) (125 21) (126 3) (129 368) (130 45) (131 85) (132 37) (133 41) (134 15) (135 109) (136 71) (137 45) (138 32) (139 15) (140 4) (141 4) (143 30) (145 67) (146 19) (147 25) (148 9) (149 66) (150 101) (151 30) (152 18) (153 9) (154 2) (155 11) (156 4) (157 17) (158 3) (159 22) (160 6) (161 12) (162 4) (163 35) (164 31) (165 14) (166 9) (167 8) (168 2) (169 9) (170 3) (171 22) (172 6) (173 25) (174 7) (175 8) (176 3) (177 21) (178 17) (179 7) (180 7) (181 6) (182 2) (183 12) (185 20) (186 4) (187 21) (188 5) (189 4) (190 2) (191 11) (192 20) (193 7) (194 5) (195 5) (196 1) (197 4) (199 15) (200 2) (201 16) (202 4) (203 3) (204 2) (205 8) (206 38) (207 10) (208 3) (209 3) (211 4) (212 1) (213 7) (214 1) (215 12) (216 2) (219 7) (220 5) (221 2) (222 1) (223 2) (225 3) (227 9) (228 2) (229 9) (230 2) (233 6) (234 4) (235 2) (236 1) (239 3) (241 8) (242 2) (243 8) (244 2) (245 1) (246 2) (247 3) (248 23) (249 5) (251 1) (253 2) (255 7) (256 1) (257 5) (258 1) (261 3) (262 9) (263 2) (264 1) (265 1) (267 3) (269 3) (271 6) (272 3) (273 3) (281 3) (283 2) (285 3) (286 1) (289 2) (290 76) (291 17) (292 3) (295 3) (297 1) (299 1) (309 2) (313 1) (323 2) (337 2) (347 1) (349 1) (363 1) (365 154) (366 44) (367 16) (380 9) (381 2) (382 1) |
| 172 | 2426,3 | 14,8399 | 43 | (90 42) (100 89) (102 12) (114 6) (115 222) (116 674) (128 999) (131 617) (142 98) (144 874) (158 87) (170 25) (182 20) (184 138) (198 200) (200 59) (212 43) (214 14) (224 10) (226 87) (238 38) (240 65) (252 43) (254 81) (256 13) (266 20) (268 44) (280 14) (282 25) (294 8) (296 15) (308 11) (310 29) (317 4) (336 22) (338 406) (339 66) (340 18) (352 12) (353 99) (354 31) (355 9) (356 1) |
| 173 | 2429,6 | 14,8571 | 229 | (51 6) (52 4) (53 52) (54 120) (55 740) (56 140) (57 100) (58 22) (59 26) (60 11) (61 61) (62 4) (63 3) (65 24) (66 24) (67 327) (68 112) (69 348) (70 63) (71 33) (72 44) (73 999) (74 100) (75 927) (76 63) (77 75) (78 20) (79 167) (80 68) (81 258) (82 134) (83 186) (84 132) (85 37) (86 10) (87 9) (88 7) (89 32) (91 46) (92 12) (93 96) (94 54) (95 248) (96 223) (97 157) (98 129) (99 36) (101 12) (103 8) (104 1) (105 38) (106 8) (107 58) (108 59) (109 125) (110 95) (111 72) (112 32) (113 10) (117 741) (118 70) (119 58) (120 11) (121 70) (122 25) (123 75) (124 44) (125 29) (126 7) (127 5) (129 794) (130 90) (132 128) (133 83) (134 30) (135 58) (136 18) (137 46) (138 35) (139 16) (140 3) (141 6) (143 56) (145 257) (146 31) (147 32) (148 16) (149 32) (150 12) (151 30) (152 35) (153 12) (154 2) (155 17) (157 22) (159 25) (160 4) (161 14) (162 5) (163 14) (164 5) (165 19) (166 20) (167 9) (168 2) (169 13) (171 49) (172 10) (173 17) (174 5) (175 10) (176 4) (177 9) (178 4) (179 11) (180 12) (181 7) (183 23) (185 90) (186 11) (187 18) (188 9) (189 7) (190 3) (191 7) (192 4) (193 9) (194 17) (195 7) (197 9) (199 76) (201 26) (202 4) (205 3) (206 2) (207 11) (208 38) (209 10) (211 9) (213 17) (215 8) (218 2) (219 4) (221 15) (222 9) (223 3) (225 4) (227 17) (229 6) (230 2) (231 3) (232 1) (233 1) (235 17) (236 8) (237 4) (239 4) (241 17) (242 2) (243 6) (244 2) (245 2) (246 1) (247 1) (248 8) (249 22) (250 47) (251 11) (253 2) (255 14) (257 5) (258 2) (259 2) (260 1) (263 15) (264 10) (265 3) (267 4) (269 15) (271 5) (272 1) (273 1) (274 5) (275 2) (277 4) (281 4) (283 11) (284 2) (285 9) (286 4) (287 2) (288 1) (289 1) (291 2) (292 60) (293 15) (295 3) (297 7) (299 5) (300 6) (301 2) (305 1) (307 1) (309 3) (311 3) (313 2) (314 3) (315 1) (319 1) (321 2) (323 12) (325 3) (327 3) (335 1) (337 3) (341 1) (349 3) (350 1) (351 4) (365 1) (367 357) (368 103) (369 27) (370 4) (371 1) (378 1) (382 21) (383 6) (384 2) |
| 174 | 2433,8 | 14,8785 | 35 | (90 47) (100 82) (102 11) (115 243) (116 741) (128 999) (142 94) (144 890) (158 102) (170 33) (182 22) (184 151) (198 248) (200 55) (212 49) (214 27) (226 76) (238 39) (240 73) (252 57) (254 84) (256 12) (266 45) (268 65) (282 48) (296 12) (310 25) (336 23) (338 439) (339 74) (340 14) (352 13) (353 117) (354 35) (355 6) |
| 175 | 2434,1 | 14,8802 | 32 | (90 49) (91 94) (100 90) (102 15) (115 266) (116 810) (128 917) (131 999) (142 105) (144 846) (156 25) (158 117) (170 50) (172 22) (182 26) (186 54) (198 230) (200 78) (203 25) (212 56) (214 41) (252 54) (256 20) (266 59) (268 63) (270 16) (282 57) (284 9) (296 17) (308 11) (324 11) (353 108) |
| 176 | 2436,7 | 14,8934 | 25 | (57 999) (71 914) (85 675) (113 85) (125 42) (140 71) (141 45) (150 30) (154 54) (187 323) (188 39) (204 57) (206 22) (290 35) (330 17) (331 15) (332 6) (359 176) (360 54) (361 27) (365 77) (366 19) (413 25) (414 8) (429 9) |
| 177 | 2439,5 | 14,9078 | 110 | (51 10) (52 8) (53 49) (54 138) (55 522) (56 93) (60 8) (61 60) (62 5) (65 16) (66 6) (67 105) (68 35) (69 199) (70 45) (72 56) (74 87) (75 801) (76 49) (77 37) (81 24) (82 19) (83 16) (84 7) (86 16) (90 28) (91 38) (96 12) (100 62) (102 11) (110 6) (114 7) (115 157) (116 629) (122 16) (124 6) (126 26) (128 517) (130 35) (131 999) (136 9) (142 53) (144 475) (146 10) (152 6) (156 19) (158 59) (159 9) (160 4) (162 3) (164 4) (166 3) (170 34) (172 18) (173 11) (182 4) (184 53) (186 56) (198 69) (200 62) (203 17) (212 29) (213 4) (214 22) (216 4) (226 43) (228 17) (234 2) (238 5) (240 24) (242 28) (252 7) (253 2) (254 20) (256 14) (268 20) (270 9) (272 2) (278 3) (280 39) (281 8) (282 19) (283 3) (284 5) (292 3) (294 56) (295 12) (296 38) (298 6) (299 3) (308 27) (309 8) (310 38) (311 8) (312 4) (322 6) (323 2) (324 21) (334 1) (336 19) (337 7) (338 277) (339 69) (340 18) (351 2) (352 6) (353 91) (354 26) (355 7) (410 1) |
| 178 | 2442,4 | 14,9226 | 54 | (99 67) (101 59) (103 265) (104 22) (112 78) (133 186) (143 73) (147 354) (148 90) (149 63) (163 16) (167 14) (169 176) (183 134) (189 43) (191 69) (197 19) (199 63) (205 27) (215 51) (217 999) (218 223) (219 102) (225 14) (227 58) (229 20) (230 48) (231 45) (241 16) (243 93) (244 23) (245 58) (246 16) (247 7) (257 111) (258 36) (259 235) (260 52) (261 20) (271 13) (285 11) (313 50) (314 10) (327 8) (329 26) (348 8) (369 415) (370 119) (371 32) (384 55) (385 15) (386 4) (459 20) (460 6) |
| 179 | 2443,4 | 14,928 | 51 | (58 35) (59 35) (71 55) (73 999) (89 28) (99 49) (101 31) (103 133) (112 45) (117 941) (118 64) (119 47) (129 405) (132 193) (133 96) (134 24) (135 17) (143 43) (145 172) (147 156) (148 30) (149 33) (167 8) (169 67) (171 17) (185 20) (189 19) (191 29) (199 23) (201 39) (205 14) (215 17) (219 38) (229 10) (231 18) (241 11) (243 31) (244 7) (245 22) (257 42) (259 82) (261 7) (262 5) (285 8) (325 16) (369 282) (370 77) (371 19) (384 35) (385 9) (386 3) |
| 180 | 2450 | 14,9619 | 194 | (51 10) (52 5) (53 28) (54 35) (55 179) (56 41) (57 111) (58 17) (59 26) (60 8) (61 42) (62 3) (63 4) (65 38) (66 27) (67 153) (68 23) (69 58) (70 24) (71 34) (72 43) (73 689) (74 78) (75 559) (76 40) (77 109) (78 47) (79 186) (80 64) (81 52) (82 21) (83 25) (84 24) (86 9) (87 3) (89 9) (90 8) (91 202) (92 51) (93 94) (94 20) (95 28) (96 17) (97 21) (99 7) (100 26) (101 7) (102 5) (103 12) (104 23) (105 96) (106 53) (107 26) (108 7) (109 12) (110 10) (111 9) (112 3) (113 6) (114 4) (115 77) (116 398) (117 161) (118 36) (119 69) (120 27) (121 15) (122 9) (123 5) (124 6) (125 4) (126 4) (127 3) (128 120) (129 76) (130 23) (131 999) (132 126) (133 70) (134 14) (135 18) (136 7) (138 2) (139 1) (140 2) (141 8) (142 21) (143 14) (144 124) (145 51) (146 32) (147 25) (148 12) (149 6) (150 2) (151 2) (152 3) (153 3) (154 4) (155 8) (156 10) (157 11) (158 19) (159 22) (160 13) (161 11) (162 10) (163 2) (165 2) (166 2) (167 3) (168 2) (169 11) (170 14) (171 14) (172 11) (173 16) (174 5) (175 19) (176 5) (180 2) (181 3) (182 2) (183 8) (184 9) (185 7) (186 15) (187 11) (188 8) (189 3) (190 1) (191 2) (194 1) (195 2) (196 2) (197 4) (198 6) (199 6) (200 14) (201 12) (202 4) (203 9) (204 3) (205 2) (208 2) (209 2) (210 1) (212 6) (213 4) (214 5) (215 5) (216 3) (222 2) (223 3) (224 1) (225 1) (226 5) (227 3) (228 3) (229 1) (230 1) (233 1) (236 1) (237 1) (238 2) (239 1) (240 6) (241 3) (242 5) (243 2) (254 5) (256 3) (257 2) (264 1) (266 1) (277 1) (278 3) (279 1) (284 3) (294 2) (295 1) (296 2) (298 2) (310 2) (312 6) (326 3) (331 2) (340 110) (341 29) (342 7) (355 10) (356 3) (357 1) (389 1) |
| 181 | 2450,2 | 14,9626 | 171 | (51 10) (52 5) (53 29) (54 36) (55 184) (56 41) (57 113) (58 17) (59 26) (60 8) (61 42) (62 3) (63 4) (65 40) (66 29) (67 160) (68 24) (69 58) (70 24) (71 35) (72 43) (73 702) (74 80) (75 571) (76 40) (77 114) (78 50) (80 68) (81 54) (82 21) (83 26) (86 9) (87 3) (89 9) (90 8) (91 212) (92 55) (93 98) (94 21) (95 29) (96 17) (97 22) (99 7) (100 26) (101 7) (102 5) (103 12) (104 24) (105 105) (106 58) (107 28) (108 7) (109 12) (110 10) (111 9) (112 3) (113 6) (114 4) (115 78) (116 397) (117 167) (118 37) (119 75) (120 29) (121 16) (122 10) (123 5) (124 6) (125 4) (126 4) (128 119) (129 78) (130 24) (131 999) (132 127) (133 72) (134 14) (135 19) (136 7) (138 2) (140 2) (141 8) (142 21) (143 15) (144 124) (145 52) (146 35) (147 26) (148 12) (149 6) (150 2) (151 2) (152 3) (153 3) (154 4) (155 9) (156 10) (157 12) (158 19) (159 22) (160 13) (161 12) (162 11) (163 2) (165 2) (166 2) (167 3) (168 2) (169 11) (170 14) (171 15) (172 11) (173 16) (174 5) (175 21) (176 5) (180 2) (181 4) (182 3) (183 8) (184 9) (185 7) (186 15) (187 11) (188 9) (189 3) (190 1) (191 2) (194 1) (195 2) (196 2) (197 4) (198 6) (199 6) (200 14) (201 12) (202 4) (203 9) (204 3) (208 2) (209 2) (210 1) (212 6) (213 4) (214 5) (215 5) (216 3) (222 2) (223 3) (227 3) (228 3) (236 1) (237 1) (238 2) (239 1) (240 6) (241 3) (242 5) (243 2) (256 3) (257 2) (277 1) (278 3) (279 1) (294 2) (295 1) (296 2) (298 2) (331 2) (356 3) (357 1) |
| 182 | 2450,6 | 14,9649 | 138 | (51 17) (52 8) (53 44) (54 54) (55 275) (56 59) (57 163) (58 24) (59 37) (60 11) (61 60) (62 5) (63 6) (65 65) (66 46) (67 256) (68 36) (69 87) (71 53) (72 61) (73 999) (74 113) (75 818) (76 58) (77 183) (78 81) (79 321) (80 111) (81 85) (82 31) (83 37) (84 45) (86 13) (87 4) (89 14) (90 10) (91 342) (92 89) (93 158) (94 33) (95 45) (96 25) (97 32) (99 10) (101 10) (102 6) (103 20) (104 39) (105 170) (106 94) (107 44) (108 11) (109 19) (110 14) (111 12) (112 4) (113 8) (114 5) (115 105) (117 258) (118 54) (119 121) (120 46) (121 25) (122 14) (123 8) (124 9) (125 6) (126 5) (129 118) (130 34) (133 103) (134 22) (135 30) (136 11) (141 13) (142 28) (143 22) (145 75) (146 53) (147 40) (148 19) (149 9) (150 3) (151 3) (152 4) (153 5) (154 6) (155 14) (156 12) (157 18) (159 34) (160 21) (161 18) (162 17) (163 4) (167 4) (168 3) (169 16) (170 18) (171 23) (172 15) (173 24) (174 8) (175 33) (176 8) (181 5) (182 3) (183 12) (185 11) (187 16) (188 12) (189 5) (190 2) (191 3) (195 3) (196 3) (197 6) (199 8) (201 18) (202 5) (208 3) (209 3) (210 2) (213 6) (215 8) (216 4) (223 3) (227 5) (236 2) (237 2) (239 2) (241 4) (277 1) (278 4) (294 2) (295 2) (331 3) |
| 183 | 2450,8 | 14,9657 | 105 | (51 48) (52 23) (53 128) (54 150) (55 770) (56 161) (58 66) (59 100) (60 30) (62 13) (63 17) (65 191) (66 135) (67 747) (68 101) (69 242) (71 150) (72 167) (76 160) (77 532) (78 239) (79 947) (80 329) (81 243) (82 86) (83 104) (84 137) (85 147) (86 37) (89 38) (91 999) (92 260) (93 462) (94 97) (95 128) (96 68) (97 87) (101 27) (103 60) (104 115) (105 497) (106 277) (107 129) (108 30) (109 54) (110 39) (111 34) (117 737) (118 151) (119 354) (120 135) (121 71) (122 40) (123 23) (124 24) (125 16) (129 333) (130 93) (133 282) (134 62) (135 86) (136 32) (141 39) (143 63) (145 207) (146 151) (147 112) (148 53) (149 24) (150 9) (155 40) (157 51) (159 96) (160 59) (161 51) (162 50) (163 10) (167 12) (168 9) (169 44) (171 63) (173 69) (174 23) (175 95) (176 22) (181 14) (183 34) (185 30) (188 34) (189 14) (191 9) (195 9) (197 17) (199 23) (201 49) (202 15) (208 9) (209 9) (213 18) (215 21) (236 5) (239 6) (278 12) (295 7) (331 8) |
| 184 | 2463,8 | 15,0327 | 41 | (54 21) (55 268) (56 203) (57 999) (58 33) (67 24) (68 32) (69 188) (70 84) (71 607) (72 23) (82 28) (83 127) (84 51) (85 408) (86 20) (96 15) (97 90) (98 31) (99 115) (111 38) (112 16) (113 58) (114 4) (124 6) (125 19) (127 35) (141 30) (155 25) (169 13) (183 14) (197 15) (239 8) (253 8) (267 7) (308 11) (309 30) (310 7) (337 5) (357 11) (358 3) |
| 185 | 2466,7 | 15,0474 | 155 | (59 19) (61 6) (73 999) (74 84) (101 14) (103 36) (129 73) (130 7) (131 30) (133 61) (134 10) (135 12) (140 31) (142 5) (143 25) (145 8) (147 337) (148 51) (149 34) (150 5) (151 8) (153 5) (159 22) (160 6) (161 7) (173 15) (175 6) (181 5) (187 8) (189 15) (190 7) (191 80) (192 15) (193 12) (195 5) (201 8) (203 11) (204 28) (205 10) (206 4) (207 17) (208 6) (209 4) (211 19) (212 3) (213 4) (215 11) (216 4) (217 92) (218 32) (219 15) (220 4) (221 18) (222 3) (223 3) (225 9) (227 8) (228 3) (229 4) (230 14) (231 5) (241 3) (242 3) (243 27) (244 6) (245 6) (246 20) (247 4) (251 7) (255 13) (256 4) (257 7) (259 5) (265 5) (269 4) (270 3) (271 6) (279 3) (283 9) (284 4) (285 10) (286 3) (291 10) (292 3) (293 3) (299 231) (300 56) (301 33) (302 6) (303 3) (304 4) (305 19) (306 7) (307 3) (313 12) (314 5) (315 250) (316 60) (317 42) (318 325) (319 94) (320 44) (321 8) (322 2) (329 2) (331 4) (332 3) (333 3) (341 3) (342 8) (343 22) (344 7) (345 10) (346 2) (360 3) (369 4) (373 15) (374 4) (375 2) (386 5) (387 111) (388 36) (389 56) (390 16) (391 8) (392 2) (405 13) (406 5) (407 2) (417 4) (418 2) (419 8) (420 18) (421 7) (422 2) (429 10) (430 3) (431 3) (432 14) (433 12) (434 7) (435 3) (442 2) (443 3) (455 3) (457 5) (458 2) (459 1) (461 3) (462 1) (470 10) (471 4) (472 2) (495 2) (497 1) |
| 186 | 2477,1 | 15,1007 | 101 | (53 74) (54 94) (55 440) (59 29) (60 15) (61 51) (65 91) (66 109) (67 566) (68 78) (69 220) (72 40) (73 999) (74 115) (75 729) (76 57) (77 277) (78 168) (79 749) (80 435) (81 266) (82 55) (83 123) (84 35) (91 548) (92 144) (93 354) (94 120) (95 143) (96 42) (97 72) (101 42) (103 75) (104 41) (105 243) (106 215) (107 115) (108 37) (109 74) (110 34) (111 24) (115 38) (116 56) (117 430) (118 65) (119 146) (120 108) (121 81) (122 22) (123 26) (124 10) (125 19) (129 185) (130 51) (131 147) (132 58) (133 146) (134 45) (135 56) (136 16) (137 13) (145 85) (146 34) (147 187) (148 41) (149 42) (150 17) (151 13) (155 22) (157 27) (159 38) (160 17) (161 32) (162 14) (163 13) (164 63) (165 18) (171 27) (172 9) (173 23) (175 45) (176 11) (177 15) (178 18) (183 19) (187 26) (188 12) (189 21) (190 12) (197 10) (201 15) (204 24) (205 162) (206 31) (207 14) (231 9) (238 11) (249 7) (265 13) (342 8) (458 6) |
| 187 | 2493,4 | 15,1849 | 71 | (51 69) (53 92) (54 45) (55 313) (58 435) (61 61) (65 123) (66 125) (67 609) (68 83) (72 137) (73 999) (74 108) (75 966) (76 76) (77 369) (78 170) (79 969) (80 329) (81 238) (82 53) (84 56) (91 721) (92 175) (93 410) (94 153) (95 167) (96 31) (104 46) (105 313) (106 131) (107 140) (108 160) (109 54) (115 27) (117 181) (118 55) (119 259) (120 148) (121 79) (122 37) (128 24) (129 173) (131 130) (132 28) (133 72) (134 59) (135 44) (142 16) (143 49) (144 21) (145 133) (146 23) (155 26) (157 29) (159 47) (160 25) (161 44) (162 42) (169 30) (171 23) (173 42) (174 33) (175 46) (176 15) (185 21) (187 35) (201 32) (215 18) (229 13) (252 8) |
| 188 | 2500,8 | 15,2228 | 75 | (53 13) (54 25) (55 283) (56 152) (57 999) (58 36) (67 37) (68 28) (69 181) (70 116) (71 685) (72 36) (73 43) (81 16) (82 33) (83 118) (84 61) (85 442) (86 26) (95 8) (96 17) (97 88) (98 35) (99 111) (100 8) (101 9) (103 11) (110 8) (111 42) (112 21) (113 64) (114 4) (116 6) (124 4) (125 14) (126 17) (127 36) (128 4) (129 8) (138 2) (139 6) (140 11) (141 29) (145 4) (147 18) (153 3) (154 10) (155 22) (156 3) (168 7) (169 15) (182 5) (183 13) (187 3) (197 10) (201 3) (203 6) (204 2) (205 6) (210 3) (211 7) (224 2) (225 14) (239 5) (253 4) (266 2) (267 4) (281 3) (295 3) (309 2) (352 3) (357 30) (358 8) (359 2) (445 2) |
| 189 | 2511,3 | 15,2766 | 100 | (53 81) (54 287) (55 999) (56 298) (57 380) (58 44) (61 8) (65 32) (66 23) (67 334) (68 136) (69 666) (70 330) (71 162) (72 14) (77 26) (79 113) (80 61) (81 194) (82 238) (83 471) (84 172) (85 49) (86 5) (91 26) (93 62) (94 68) (95 96) (96 177) (97 341) (98 91) (105 12) (107 28) (108 61) (109 46) (110 121) (111 130) (112 97) (119 12) (120 14) (121 16) (122 309) (123 79) (124 82) (125 44) (126 19) (134 19) (135 12) (136 241) (137 66) (138 64) (139 21) (140 9) (148 13) (150 101) (151 35) (152 58) (153 16) (154 8) (162 8) (163 5) (164 70) (165 19) (166 43) (167 12) (168 6) (176 4) (178 61) (179 15) (180 32) (181 7) (192 54) (193 11) (194 23) (195 6) (206 47) (207 12) (208 14) (220 43) (221 11) (222 11) (223 3) (234 41) (235 12) (236 8) (248 39) (249 12) (250 6) (262 44) (263 13) (264 6) (276 52) (277 15) (278 10) (290 33) (291 8) (304 9) (318 7) (319 34) (320 8) |
| 190 | 2525,3 | 15,3483 | 43 | (53 41) (54 115) (55 649) (56 174) (61 59) (67 239) (68 102) (69 384) (72 50) (73 980) (74 105) (75 999) (76 69) (81 239) (82 116) (83 211) (84 129) (95 166) (96 153) (97 131) (98 88) (109 85) (116 58) (117 520) (118 38) (123 41) (129 508) (130 60) (131 66) (132 87) (134 21) (145 163) (171 22) (173 22) (185 54) (186 16) (199 43) (306 38) (307 11) (381 203) (382 64) (383 22) (396 17) |
| 191 | 2535,3 | 15,3999 | 32 | (55 257) (56 154) (57 999) (58 60) (68 28) (69 176) (70 97) (71 688) (72 34) (82 27) (83 122) (84 61) (85 533) (86 38) (97 117) (98 31) (99 153) (111 77) (113 79) (114 12) (125 40) (127 63) (140 18) (141 37) (154 56) (155 40) (183 21) (210 19) (211 15) (238 14) (239 12) (351 5) |
| 192 | 2537,3 | 15,4101 | 64 | (55 318) (56 170) (57 999) (58 89) (59 44) (65 24) (67 163) (68 51) (69 185) (70 85) (71 780) (72 55) (73 529) (74 44) (75 121) (77 67) (79 176) (80 63) (81 55) (83 115) (84 105) (85 599) (86 40) (89 26) (91 91) (92 38) (93 70) (97 122) (98 45) (99 134) (101 49) (103 464) (104 40) (105 87) (107 27) (111 78) (115 29) (122 36) (123 41) (125 32) (127 64) (129 196) (131 53) (133 49) (134 28) (135 34) (140 24) (141 59) (147 153) (148 42) (154 57) (155 55) (174 27) (175 21) (183 23) (191 27) (201 18) (203 36) (218 50) (219 24) (232 11) (238 21) (239 15) (257 14) |
| 193 | 2548,3 | 15,4663 | 104 | (53 24) (54 68) (55 347) (56 66) (59 68) (60 9) (61 25) (65 12) (66 12) (67 129) (68 43) (69 187) (70 28) (72 25) (73 999) (74 82) (75 285) (76 21) (77 26) (79 32) (80 17) (81 107) (82 29) (83 81) (84 15) (88 7) (89 47) (90 5) (93 24) (95 66) (96 28) (97 47) (101 69) (102 10) (103 881) (104 83) (105 47) (107 14) (109 30) (110 11) (111 17) (112 8) (115 26) (116 23) (117 52) (118 7) (119 13) (121 16) (123 11) (129 636) (130 87) (131 118) (132 12) (133 81) (135 21) (137 12) (145 20) (146 8) (147 368) (148 57) (149 56) (150 9) (151 8) (159 9) (163 23) (173 7) (175 19) (177 11) (185 6) (189 18) (190 4) (191 77) (192 16) (193 8) (201 55) (202 9) (203 78) (204 10) (215 13) (217 68) (218 160) (219 59) (220 21) (221 8) (237 19) (243 7) (244 5) (257 34) (258 16) (272 5) (285 5) (286 12) (311 52) (312 13) (325 6) (339 10) (340 3) (382 19) (383 17) (384 6) (385 7) (401 9) (457 11) (458 3) |
| 194 | 2556 | 15,5061 | 25 | (59 108) (67 96) (72 62) (73 999) (74 85) (75 186) (87 113) (88 37) (89 61) (101 145) (103 461) (104 36) (105 49) (115 32) (116 238) (117 590) (118 76) (119 50) (129 173) (131 45) (147 157) (161 126) (162 23) (205 31) (309 15) |
| 195 | 2561,9 | 15,5365 | 56 | (55 205) (56 79) (58 56) (59 75) (60 22) (61 23) (73 999) (74 98) (75 275) (83 82) (87 23) (89 62) (95 39) (97 63) (98 36) (103 419) (104 32) (113 68) (115 39) (129 939) (130 100) (131 105) (133 103) (134 18) (145 50) (147 563) (148 90) (149 84) (159 43) (163 60) (174 20) (175 37) (177 27) (191 229) (192 39) (193 16) (203 168) (204 29) (217 157) (218 571) (219 125) (220 60) (221 18) (239 43) (313 172) (314 44) (327 17) (329 9) (384 9) (387 19) (401 16) (403 24) (404 8) (459 26) (460 11) (461 5) |
| 196 | 2571 | 15,5833 | 18 | (56 43) (57 327) (70 174) (71 242) (83 59) (99 13) (104 48) (112 36) (113 66) (114 7) (149 999) (150 93) (151 8) (167 279) (168 20) (279 47) (280 8) (313 4) |
| 197 | 2573,8 | 15,5973 | 47 | (59 150) (84 180) (97 118) (98 157) (101 323) (102 37) (103 789) (110 70) (111 61) (122 155) (123 98) (129 727) (135 305) (136 64) (137 30) (147 999) (148 251) (152 34) (162 65) (163 39) (176 27) (189 53) (190 21) (201 98) (203 210) (204 38) (205 124) (206 25) (219 57) (231 17) (232 97) (233 26) (237 50) (257 20) (275 55) (276 10) (295 11) (308 11) (309 14) (337 9) (365 120) (366 31) (370 20) (378 27) (379 10) (453 49) (454 17) |
| 198 | 2576,9 | 15,6134 | 145 | (51 25) (52 14) (53 92) (54 33) (55 184) (58 9) (59 22) (60 9) (61 42) (62 5) (63 9) (64 3) (65 121) (66 125) (67 559) (68 44) (69 49) (72 45) (73 999) (74 95) (75 597) (76 38) (77 351) (78 199) (79 869) (80 238) (81 144) (82 27) (87 5) (88 9) (89 12) (90 4) (91 751) (92 162) (93 345) (94 101) (95 116) (96 13) (104 45) (105 292) (106 217) (107 108) (108 175) (109 42) (115 56) (116 79) (117 409) (118 87) (119 238) (120 79) (121 49) (122 16) (124 3) (125 3) (127 10) (128 31) (130 40) (131 182) (132 96) (133 109) (134 40) (139 3) (140 2) (142 14) (143 57) (144 24) (145 104) (146 49) (153 4) (154 4) (155 20) (156 9) (157 51) (158 16) (159 72) (160 27) (161 39) (162 12) (165 4) (166 3) (169 24) (170 9) (171 37) (172 15) (173 42) (174 18) (175 17) (176 4) (179 2) (180 8) (181 14) (182 5) (183 19) (184 7) (185 28) (186 12) (187 21) (188 9) (194 2) (195 7) (196 3) (197 15) (198 5) (199 17) (200 7) (202 4) (207 4) (209 6) (210 3) (211 9) (212 4) (213 14) (214 5) (215 14) (221 5) (223 14) (224 12) (225 7) (226 3) (227 8) (228 3) (235 3) (239 5) (240 2) (241 11) (242 2) (249 3) (251 2) (252 1) (253 3) (254 1) (255 3) (263 3) (264 3) (265 2) (267 6) (268 3) (281 2) (291 2) (292 1) (304 1) (331 2) (354 5) (355 1) (356 1) |
| 199 | 2582,3 | 15,6409 | 122 | (56 187) (70 93) (83 294) (84 72) (85 58) (89 68) (96 70) (97 169) (98 95) (99 40) (101 275) (102 27) (103 634) (110 26) (111 78) (112 24) (113 52) (121 102) (123 72) (124 19) (125 34) (129 999) (130 135) (135 116) (136 24) (137 77) (138 16) (139 16) (141 322) (142 29) (147 876) (148 144) (149 155) (150 69) (151 42) (152 13) (153 14) (163 37) (164 15) (165 15) (168 9) (175 53) (176 23) (177 26) (179 12) (188 18) (189 34) (190 12) (191 14) (192 18) (193 11) (194 6) (201 159) (202 27) (203 248) (204 59) (205 138) (206 22) (207 14) (215 32) (216 7) (217 38) (218 44) (219 83) (220 18) (221 14) (227 11) (229 13) (231 7) (234 6) (235 13) (236 22) (237 151) (238 30) (243 32) (244 20) (245 8) (250 8) (255 12) (257 99) (258 32) (271 12) (272 11) (276 9) (277 12) (279 100) (280 21) (285 16) (286 32) (287 7) (288 5) (297 8) (299 7) (300 9) (304 3) (311 49) (312 13) (313 14) (325 13) (326 5) (339 28) (340 10) (351 8) (353 7) (360 22) (361 12) (369 518) (370 142) (371 39) (372 6) (375 4) (382 47) (383 19) (384 5) (389 5) (399 6) (404 3) (457 72) (458 26) (459 11) (472 16) (473 6) |
| 200 | 2592,6 | 15,6942 | 176 | (51 23) (52 16) (53 97) (54 48) (55 292) (59 20) (60 10) (61 61) (62 6) (63 10) (64 3) (65 123) (66 124) (67 638) (68 57) (72 25) (73 891) (74 95) (75 964) (76 70) (77 346) (78 187) (79 999) (80 327) (81 246) (82 42) (87 5) (89 19) (90 5) (91 746) (92 177) (93 462) (94 192) (95 197) (96 24) (104 44) (105 365) (106 126) (107 181) (108 197) (109 64) (110 8) (115 51) (116 49) (117 326) (118 65) (119 347) (120 109) (121 107) (122 49) (123 19) (128 23) (129 250) (130 45) (131 217) (132 55) (133 177) (134 97) (135 83) (136 25) (137 10) (142 10) (143 56) (144 19) (145 153) (146 35) (148 62) (149 27) (150 9) (151 5) (153 3) (156 6) (157 41) (158 14) (159 86) (160 25) (161 70) (162 35) (163 18) (164 4) (165 4) (167 6) (169 21) (170 7) (171 36) (172 9) (173 53) (174 21) (175 55) (176 44) (177 9) (178 2) (179 2) (181 8) (184 5) (185 28) (186 7) (187 29) (188 12) (189 13) (190 7) (191 2) (193 1) (194 1) (195 5) (198 2) (199 15) (200 4) (201 32) (202 9) (209 4) (212 2) (213 9) (214 3) (215 22) (216 7) (223 4) (225 8) (226 2) (227 5) (228 2) (229 8) (230 2) (231 1) (232 1) (233 1) (235 2) (236 1) (237 4) (241 5) (242 2) (243 5) (244 1) (249 2) (250 1) (251 4) (255 3) (256 1) (261 1) (263 5) (264 1) (265 3) (266 5) (269 3) (270 1) (279 2) (283 3) (284 1) (289 1) (291 2) (292 1) (293 2) (303 1) (305 3) (306 3) (307 1) (317 2) (318 1) (319 2) (320 2) (321 1) (322 1) (331 2) (332 1) (333 5) (334 3) (335 2) (345 2) (346 1) (347 1) (348 1) (358 2) (359 2) (361 1) (387 5) (388 1) |
| 201 | 2595,6 | 15,7095 | 47 | (88 16) (101 173) (102 13) (103 150) (147 367) (203 179) (204 34) (205 162) (206 30) (207 10) (218 43) (219 13) (220 6) (221 11) (239 202) (240 35) (245 5) (257 13) (258 4) (259 5) (275 4) (285 7) (299 6) (311 6) (313 54) (314 13) (315 3) (327 7) (328 7) (329 3) (341 7) (342 6) (355 3) (371 999) (372 280) (373 71) (374 11) (383 2) (384 8) (385 2) (401 14) (402 3) (403 4) (459 66) (460 23) (461 8) (462 2) |
| 202 | 2607,8 | 15,7696 | 245 | (53 3) (54 2) (58 10) (59 27) (60 3) (61 6) (65 1) (66 1) (67 1) (68 2) (69 8) (70 4) (72 20) (73 999) (74 83) (75 84) (76 4) (77 3) (81 3) (84 8) (86 2) (87 4) (88 1) (89 11) (90 1) (92 1) (95 1) (97 3) (98 2) (99 7) (100 4) (101 20) (102 3) (103 101) (104 10) (105 6) (109 1) (110 6) (111 11) (112 2) (113 9) (114 2) (115 21) (116 13) (117 29) (118 4) (119 8) (120 2) (121 1) (122 1) (123 2) (124 3) (125 3) (126 3) (127 5) (128 2) (129 41) (130 5) (131 23) (132 4) (133 43) (134 8) (135 5) (136 1) (137 1) (138 8) (139 3) (140 3) (141 5) (142 5) (143 22) (144 4) (145 9) (146 3) (147 137) (148 21) (149 19) (150 4) (151 2) (152 2) (153 5) (154 1) (155 3) (156 3) (157 9) (158 3) (159 3) (160 1) (161 1) (162 3) (163 4) (164 2) (165 4) (166 13) (167 3) (168 2) (169 28) (170 5) (171 5) (172 2) (173 2) (175 1) (176 1) (177 6) (178 2) (179 3) (180 3) (181 3) (182 1) (183 1) (184 1) (185 1) (186 1) (187 1) (188 1) (189 16) (190 3) (191 10) (192 5) (193 103) (194 15) (195 5) (196 1) (199 1) (201 2) (203 2) (204 5) (205 4) (206 5) (207 27) (208 12) (209 47) (210 7) (211 3) (215 9) (216 2) (217 275) (218 56) (219 28) (220 4) (221 5) (222 2) (223 1) (224 1) (229 5) (230 211) (231 50) (232 21) (233 5) (234 2) (235 3) (237 84) (238 14) (239 5) (240 1) (241 1) (243 57) (244 13) (245 145) (246 30) (247 15) (248 3) (249 2) (250 1) (251 1) (252 1) (257 6) (258 14) (259 86) (260 20) (261 9) (262 1) (263 1) (264 1) (265 17) (266 5) (267 4) (268 1) (277 1) (278 1) (279 6) (280 26) (281 109) (282 27) (283 11) (284 2) (287 1) (291 2) (292 1) (293 2) (294 1) (295 1) (299 1) (305 1) (307 23) (308 6) (309 9) (310 3) (311 1) (319 1) (320 4) (321 2) (322 2) (323 19) (324 5) (325 2) (333 2) (334 1) (335 10) (336 2) (337 1) (338 3) (339 4) (340 1) (341 1) (347 2) (348 16) (349 5) (350 2) (351 1) (353 1) (361 1) (363 5) (364 1) (365 1) (375 1) (376 1) (377 3) (378 1) (379 1) (387 1) (393 1) (395 9) (396 3) (397 2) (409 1) (425 1) (451 2) (452 1) (453 6) (454 2) (455 1) (466 3) (467 1) (469 1) |
| 203 | 2620,5 | 15,8303 | 115 | (53 33) (54 120) (55 560) (56 115) (57 162) (59 19) (60 10) (61 49) (65 24) (66 22) (67 321) (68 120) (69 296) (70 79) (71 71) (72 38) (73 999) (74 101) (75 886) (76 59) (77 70) (79 107) (80 58) (81 263) (82 136) (83 178) (84 90) (85 44) (89 25) (91 36) (93 47) (94 32) (95 167) (96 151) (97 111) (98 73) (99 26) (105 17) (107 21) (108 13) (109 76) (110 54) (111 33) (112 14) (116 35) (117 409) (118 39) (119 22) (121 25) (122 10) (123 43) (124 23) (125 12) (129 421) (130 50) (131 68) (132 69) (133 41) (134 15) (135 31) (136 13) (137 26) (138 19) (139 9) (143 24) (145 119) (146 15) (149 13) (150 15) (151 14) (152 16) (155 16) (156 7) (157 12) (159 9) (166 7) (169 10) (171 24) (183 12) (185 35) (186 10) (187 10) (199 32) (200 6) (201 14) (227 9) (235 8) (236 14) (237 6) (241 8) (242 6) (249 8) (255 8) (263 6) (264 5) (276 5) (278 15) (283 6) (291 6) (292 5) (297 8) (311 5) (313 7) (320 30) (321 9) (332 8) (351 4) (367 5) (395 161) (396 48) (397 16) (398 3) (409 3) (410 11) (411 4) |
| 204 | 2629 | 15,871 | 97 | (54 97) (55 398) (56 49) (61 64) (67 159) (68 68) (69 244) (70 34) (73 999) (74 125) (75 905) (76 69) (77 43) (81 130) (82 77) (83 65) (84 39) (89 21) (90 13) (95 73) (96 61) (97 47) (98 30) (100 33) (101 24) (107 16) (108 18) (109 49) (110 27) (115 83) (116 298) (117 240) (118 26) (121 17) (122 21) (123 26) (128 244) (129 223) (130 54) (131 466) (132 88) (133 54) (135 16) (137 13) (143 15) (144 218) (145 91) (146 17) (158 32) (159 16) (170 17) (171 17) (172 13) (174 20) (184 19) (185 22) (186 29) (187 12) (198 37) (199 22) (200 26) (201 11) (203 15) (212 15) (214 8) (226 11) (227 10) (228 11) (240 15) (241 8) (242 7) (254 14) (266 17) (268 15) (278 6) (280 22) (281 11) (282 19) (284 8) (291 6) (296 15) (298 4) (308 6) (310 7) (320 12) (322 8) (338 13) (366 123) (367 38) (368 13) (381 49) (382 15) (384 7) (395 68) (396 23) (397 5) (410 5) |
| 205 | 2631,9 | 15,8846 | 11 | (58 999) (59 35) (72 101) (79 56) (80 17) (91 40) (93 19) (105 17) (224 6) (276 4) (375 4) |
| 206 | 2634,1 | 15,8953 | 30 | (56 128) (57 999) (70 91) (71 780) (83 76) (84 29) (85 514) (86 32) (97 94) (99 152) (111 78) (113 84) (114 8) (125 51) (127 41) (139 11) (141 44) (154 50) (155 32) (168 41) (169 20) (182 28) (183 22) (196 23) (197 13) (210 16) (224 13) (225 14) (238 14) (239 9) |
| 207 | 2641 | 15,9286 | 82 | (54 25) (55 245) (56 51) (57 364) (58 999) (59 136) (61 20) (63 10) (64 5) (65 22) (66 14) (67 127) (68 28) (69 98) (70 64) (71 659) (72 194) (73 838) (74 70) (75 269) (76 20) (77 88) (78 23) (79 120) (80 20) (81 50) (83 99) (84 54) (87 40) (89 94) (90 14) (91 141) (92 14) (95 57) (96 25) (97 44) (99 27) (101 78) (102 14) (103 175) (104 19) (105 55) (111 35) (112 113) (113 26) (116 170) (117 555) (118 61) (119 42) (124 17) (129 240) (130 20) (132 157) (133 78) (141 17) (145 131) (146 29) (147 75) (157 14) (161 26) (171 24) (185 14) (187 12) (190 8) (200 11) (201 19) (202 10) (205 15) (217 20) (242 6) (243 12) (244 6) (251 6) (255 5) (264 4) (298 3) (304 4) (353 10) (397 91) (398 26) (412 13) (413 5) |
| 208 | 2663,3 | 16,0354 | 51 | (53 9) (54 26) (55 287) (56 201) (57 999) (58 35) (67 45) (68 34) (69 207) (70 106) (71 622) (72 30) (82 38) (83 141) (84 50) (85 426) (86 29) (96 16) (97 108) (98 28) (99 129) (100 10) (111 44) (112 19) (113 61) (125 19) (126 8) (127 47) (139 9) (140 6) (141 34) (154 6) (155 26) (169 20) (182 4) (183 17) (196 5) (197 16) (210 3) (211 15) (225 13) (226 2) (239 11) (253 11) (267 7) (281 6) (295 3) (336 8) (337 27) (338 7) (365 6) |
| 209 | 2672,3 | 16,0782 | 114 | (59 26) (72 26) (73 999) (74 88) (75 80) (84 15) (86 6) (89 12) (99 12) (100 5) (103 78) (104 9) (105 10) (111 14) (113 8) (115 19) (116 9) (117 23) (123 4) (125 6) (129 15) (131 14) (133 27) (134 6) (135 5) (143 16) (144 4) (147 100) (148 17) (149 13) (150 4) (153 7) (157 8) (162 6) (165 28) (166 4) (169 24) (175 6) (176 10) (177 4) (179 4) (180 7) (188 5) (189 15) (190 10) (191 12) (192 116) (193 18) (194 5) (204 7) (206 28) (207 22) (208 60) (209 12) (210 4) (215 4) (216 3) (217 127) (218 28) (219 15) (220 6) (221 3) (229 3) (230 271) (231 58) (232 28) (233 4) (234 4) (236 287) (237 48) (238 14) (239 4) (243 56) (244 13) (245 133) (246 29) (247 13) (248 4) (257 5) (258 13) (259 32) (260 8) (261 4) (262 5) (264 18) (265 6) (266 4) (278 12) (279 13) (280 33) (281 9) (282 4) (292 3) (306 19) (307 6) (308 4) (309 2) (320 6) (321 2) (322 27) (323 7) (324 2) (334 11) (335 3) (337 7) (338 3) (348 9) (349 3) (375 2) (376 3) (394 3) (450 3) (452 3) (465 2) |
| 210 | 2700,4 | 16,2131 | 57 | (53 12) (54 26) (55 268) (56 142) (57 999) (58 37) (67 36) (68 29) (69 177) (70 117) (71 700) (72 31) (82 34) (83 125) (84 58) (85 451) (86 29) (96 18) (97 96) (98 35) (99 112) (100 8) (110 10) (111 45) (112 23) (113 60) (114 4) (124 5) (125 19) (126 20) (127 41) (138 3) (139 6) (140 14) (141 29) (154 9) (168 7) (180 2) (182 4) (183 12) (196 4) (197 11) (210 2) (211 7) (224 3) (225 7) (239 5) (252 2) (253 4) (266 2) (267 4) (281 4) (294 2) (295 4) (309 3) (323 2) (380 3) |
| 211 | 2726,9 | 16,3397 | 134 | (55 289) (56 137) (57 609) (58 50) (59 67) (60 6) (61 14) (68 17) (69 453) (70 66) (71 356) (72 48) (73 999) (74 91) (75 213) (76 10) (81 111) (82 23) (83 165) (84 32) (85 133) (86 12) (87 9) (88 13) (89 37) (95 125) (96 16) (97 149) (98 56) (99 41) (101 170) (102 17) (103 131) (107 9) (109 83) (110 12) (111 128) (112 38) (113 28) (115 18) (116 144) (117 158) (118 19) (121 10) (123 72) (124 9) (125 39) (129 178) (130 53) (131 86) (132 45) (133 114) (134 18) (135 17) (137 38) (138 6) (139 23) (140 19) (143 38) (145 126) (146 36) (147 413) (148 61) (149 60) (150 7) (151 37) (152 7) (153 11) (159 11) (161 9) (163 20) (164 4) (165 19) (170 12) (173 22) (175 49) (176 7) (179 8) (181 5) (185 6) (187 5) (188 21) (189 12) (193 5) (195 4) (201 61) (202 8) (203 126) (204 33) (205 121) (206 23) (207 17) (215 124) (216 22) (217 12) (218 31) (219 17) (220 6) (221 8) (228 25) (229 10) (237 26) (238 5) (275 4) (277 12) (278 6) (285 13) (289 11) (290 2) (293 5) (295 7) (305 22) (306 5) (307 3) (327 6) (337 11) (338 2) (355 4) (367 7) (369 23) (370 7) (383 5) (384 3) (397 3) (425 11) (427 616) (428 203) (429 55) (430 10) (440 3) (443 2) (457 8) (458 3) (459 3) |
| 212 | 2732,7 | 16,3676 | 84 | (53 29) (54 107) (55 437) (56 126) (59 34) (61 22) (67 229) (68 72) (70 104) (71 332) (72 30) (73 725) (74 62) (75 214) (76 18) (77 43) (79 108) (80 47) (81 122) (82 79) (83 130) (84 62) (85 283) (89 31) (91 43) (93 52) (94 19) (95 49) (96 40) (99 89) (103 999) (104 84) (105 59) (107 18) (110 17) (113 81) (121 14) (127 36) (129 662) (130 74) (131 89) (133 49) (135 17) (141 23) (147 232) (148 41) (149 35) (155 36) (163 18) (168 13) (177 16) (182 36) (183 24) (189 23) (191 99) (192 20) (193 11) (196 9) (201 34) (210 19) (211 14) (217 73) (218 171) (219 69) (220 23) (238 11) (239 13) (244 13) (246 10) (257 44) (258 14) (262 6) (265 15) (311 6) (314 9) (339 42) (340 9) (367 10) (410 22) (411 8) (413 5) (485 10) (486 4) (497 4) |
| 213 | 2741,1 | 16,4077 | 67 | (54 60) (55 275) (59 71) (61 19) (67 133) (68 26) (69 127) (73 999) (74 95) (75 311) (76 30) (77 39) (79 80) (80 31) (81 91) (82 32) (83 69) (89 38) (91 55) (93 66) (95 65) (101 70) (103 738) (104 74) (105 65) (109 32) (112 31) (116 41) (117 175) (119 26) (121 17) (123 17) (129 620) (130 89) (131 113) (132 44) (133 59) (135 23) (145 63) (147 358) (148 61) (149 68) (159 16) (163 21) (175 26) (177 15) (189 20) (191 65) (192 14) (201 46) (202 11) (203 74) (204 29) (205 21) (217 70) (218 150) (219 52) (220 18) (257 18) (265 16) (272 9) (339 41) (340 11) (361 21) (367 8) (410 29) (485 8) |
| 214 | 2749,9 | 16,4502 | 48 | (54 26) (59 37) (67 100) (73 999) (74 78) (75 80) (81 64) (84 48) (89 35) (93 34) (95 38) (101 38) (103 300) (104 24) (105 44) (107 18) (116 19) (117 87) (129 209) (130 31) (131 56) (133 67) (147 326) (148 41) (149 38) (157 22) (160 28) (161 12) (163 18) (169 37) (189 30) (204 233) (205 108) (206 33) (217 165) (219 33) (243 23) (271 24) (319 47) (320 14) (331 13) (337 7) (361 98) (362 27) (363 16) (408 8) (480 10) (481 5) |
| 215 | 2769,2 | 16,5426 | 115 | (53 26) (54 113) (55 490) (56 103) (57 86) (58 23) (59 57) (61 15) (65 37) (66 21) (67 309) (68 62) (69 237) (70 156) (71 37) (72 22) (73 999) (74 91) (75 256) (76 26) (77 45) (79 141) (80 59) (81 216) (82 57) (83 160) (84 57) (89 30) (91 48) (93 75) (94 19) (95 139) (96 38) (97 65) (98 44) (101 152) (102 15) (103 377) (104 68) (105 43) (107 28) (108 10) (109 56) (110 16) (113 15) (115 24) (116 87) (117 93) (118 16) (119 21) (121 55) (122 12) (123 23) (129 576) (130 82) (131 131) (132 48) (133 121) (134 18) (135 46) (137 11) (145 35) (146 13) (147 398) (148 71) (149 135) (150 23) (151 20) (163 14) (165 10) (166 8) (167 39) (175 24) (182 7) (187 20) (188 6) (201 69) (202 11) (203 113) (205 34) (215 10) (219 25) (220 11) (221 9) (237 18) (244 8) (246 6) (247 10) (257 37) (258 10) (262 15) (264 9) (265 41) (266 7) (285 5) (297 4) (307 33) (308 7) (314 10) (325 4) (339 12) (340 5) (353 4) (367 10) (368 4) (395 18) (396 6) (397 176) (398 56) (399 15) (410 16) (411 5) (485 26) (486 10) (487 3) |
| 216 | 2777,6 | 16,5825 | 84 | (53 25) (54 76) (55 424) (56 65) (59 52) (65 15) (67 180) (68 48) (69 257) (70 38) (72 19) (73 999) (74 82) (75 231) (77 24) (79 67) (81 148) (82 35) (83 146) (84 22) (88 11) (89 36) (93 39) (94 21) (95 130) (96 21) (97 70) (98 26) (101 128) (102 14) (103 328) (104 34) (105 34) (107 17) (109 55) (115 22) (116 82) (117 105) (121 22) (123 23) (129 491) (130 70) (131 99) (132 43) (133 114) (134 17) (135 25) (137 12) (145 28) (147 378) (148 65) (149 53) (151 11) (163 13) (165 10) (175 19) (177 12) (187 15) (201 61) (202 9) (203 95) (204 66) (205 71) (206 14) (219 25) (237 12) (243 15) (257 23) (265 37) (266 8) (271 11) (272 7) (287 8) (307 56) (308 12) (339 17) (340 5) (397 135) (398 46) (399 9) (410 32) (411 10) (485 24) (486 7) |
| 217 | 2792,3 | 16,6529 | 55 | (60 14) (61 29) (76 25) (77 19) (81 61) (88 28) (93 17) (95 90) (101 221) (102 28) (107 12) (109 51) (116 183) (121 14) (123 30) (132 89) (135 34) (137 15) (145 165) (151 13) (165 8) (175 60) (187 67) (188 32) (201 56) (203 221) (237 8) (249 5) (267 128) (268 21) (285 5) (299 6) (313 8) (327 4) (339 10) (340 3) (341 47) (342 13) (355 6) (356 5) (369 6) (370 6) (399 999) (400 305) (401 84) (402 14) (412 7) (415 4) (429 13) (430 4) (431 4) (487 69) (488 26) (489 8) (490 2) |
| 218 | 2804,2 | 16,7089 | 26 | (51 13) (84 69) (94 26) (98 191) (105 36) (106 6) (118 29) (119 27) (126 19) (150 20) (151 31) (180 5) (182 10) (185 10) (200 20) (203 999) (220 124) (232 20) (248 4) (261 4) (264 12) (290 139) (303 6) (305 214) (359 14) (366 2) |
| 219 | 2807,3 | 16,7225 | 22 | (68 19) (74 176) (75 162) (79 11) (92 1) (116 425) (149 40) (157 46) (172 9) (200 5) (204 31) (205 29) (226 1) (228 50) (245 17) (274 3) (290 63) (305 51) (319 20) (358 9) (361 999) (379 2) |
| 220 | 2810,2 | 16,7357 | 149 | (52 1) (53 5) (54 5) (55 12) (56 3) (57 3) (58 5) (59 26) (60 3) (61 5) (65 1) (66 3) (67 3) (68 2) (69 14) (73 999) (74 92) (75 112) (76 5) (77 5) (79 1) (80 1) (81 13) (83 6) (85 6) (86 1) (87 5) (88 4) (89 18) (90 2) (91 2) (95 1) (97 2) (99 6) (101 24) (103 156) (104 13) (105 7) (109 7) (111 4) (113 11) (114 3) (117 51) (118 6) (119 7) (120 1) (127 4) (129 131) (130 16) (131 21) (133 39) (134 5) (135 4) (139 3) (141 2) (143 14) (144 2) (145 4) (147 229) (148 36) (149 31) (150 3) (151 2) (153 3) (155 26) (157 15) (159 4) (161 3) (163 5) (169 116) (170 18) (173 5) (174 1) (175 5) (177 4) (178 1) (181 1) (183 5) (184 1) (185 2) (188 1) (189 29) (191 224) (192 36) (193 17) (194 2) (195 1) (199 3) (201 2) (204 75) (205 28) (206 8) (207 5) (215 3) (217 120) (218 28) (219 12) (221 8) (227 4) (229 9) (233 5) (241 4) (243 47) (244 11) (255 2) (257 5) (263 3) (271 58) (272 13) (273 7) (274 1) (275 1) (287 1) (289 1) (291 13) (292 4) (293 2) (303 1) (305 12) (306 5) (307 2) (317 6) (319 25) (320 7) (321 4) (331 32) (332 11) (333 6) (334 2) (335 1) (345 5) (346 2) (347 2) (361 397) (362 117) (363 57) (364 12) (365 3) (373 1) (377 2) (378 1) (379 1) (393 1) (435 3) (436 1) (437 1) (451 2) (452 1) (463 1) |
| 221 | 2812,2 | 16,7447 | 172 | (72 72) (84 15) (98 8) (100 24) (107 2) (112 6) (115 83) (116 44) (122 1) (123 2) (124 5) (125 6) (126 9) (132 35) (136 4) (137 3) (138 11) (140 5) (141 22) (144 10) (146 47) (152 7) (154 9) (158 38) (160 4) (162 4) (164 5) (165 21) (166 54) (167 11) (168 10) (171 54) (172 22) (174 5) (176 5) (179 5) (180 14) (182 10) (186 2) (187 9) (188 4) (190 49) (196 4) (197 5) (198 10) (202 4) (208 22) (209 6) (210 9) (211 4) (212 2) (216 6) (222 14) (223 11) (224 7) (225 4) (226 1) (230 290) (231 75) (232 34) (236 6) (237 9) (238 24) (239 8) (240 5) (245 594) (246 126) (247 54) (248 14) (249 4) (250 7) (251 18) (252 9) (253 5) (254 2) (258 10) (259 94) (260 24) (261 10) (262 8) (264 121) (265 28) (266 18) (267 6) (268 2) (276 4) (277 5) (278 41) (279 23) (280 628) (281 144) (282 61) (283 9) (284 1) (294 93) (295 129) (296 197) (297 50) (298 15) (299 3) (304 8) (308 45) (309 12) (310 5) (311 1) (322 11) (324 999) (325 249) (326 99) (327 16) (328 2) (335 5) (336 11) (337 4) (338 7) (339 2) (340 1) (348 24) (349 7) (350 10) (351 4) (352 93) (353 29) (354 18) (355 4) (356 1) (366 21) (367 27) (368 294) (369 95) (370 46) (371 9) (372 2) (374 2) (376 1) (380 6) (381 4) (382 5) (383 2) (384 1) (392 3) (394 107) (395 35) (396 24) (397 7) (398 2) (408 9) (409 12) (410 173) (411 60) (412 27) (413 5) (414 1) (420 1) (422 33) (423 11) (424 6) (425 37) (426 15) (427 5) (428 1) (434 2) (442 2) (448 7) (450 5) (464 7) (466 2) (468 1) (482 13) (483 5) (484 4) (485 1) |
| 222 | 2834,4 | 16,8438 | 178 | (53 32) (54 103) (55 490) (56 150) (57 418) (58 44) (59 32) (60 7) (61 39) (65 14) (66 9) (67 141) (68 60) (69 277) (70 108) (71 234) (72 56) (73 999) (74 111) (75 648) (76 46) (77 33) (79 25) (80 11) (81 74) (82 57) (83 120) (84 40) (85 146) (86 21) (89 17) (90 21) (91 32) (93 11) (94 9) (95 33) (96 39) (97 84) (98 20) (99 55) (100 45) (101 9) (102 9) (107 9) (108 8) (109 15) (110 23) (111 42) (113 37) (114 10) (115 105) (116 413) (118 13) (121 7) (122 26) (123 12) (124 16) (125 24) (128 330) (130 35) (131 759) (132 69) (133 46) (135 8) (136 18) (137 8) (138 10) (139 10) (141 20) (142 45) (143 12) (144 323) (145 40) (148 17) (152 10) (153 5) (154 26) (155 16) (156 15) (158 44) (159 10) (160 7) (161 12) (162 3) (164 6) (166 8) (167 8) (168 20) (169 31) (170 27) (171 7) (172 12) (178 6) (181 6) (183 10) (184 34) (185 5) (186 37) (187 12) (194 4) (197 8) (198 45) (199 9) (200 37) (201 12) (203 29) (212 21) (213 4) (214 16) (215 5) (216 8) (220 5) (222 3) (224 7) (225 5) (226 23) (227 6) (228 11) (231 5) (234 4) (240 22) (242 12) (252 6) (254 17) (255 5) (256 17) (262 4) (266 9) (267 7) (268 13) (269 4) (270 8) (276 5) (278 3) (280 14) (281 6) (282 18) (283 4) (284 5) (290 3) (294 27) (295 10) (296 22) (297 6) (308 31) (309 8) (310 24) (311 14) (312 9) (322 12) (323 4) (324 21) (325 5) (326 5) (336 7) (337 3) (338 9) (339 3) (340 2) (350 8) (351 3) (352 11) (353 4) (354 4) (362 9) (364 8) (366 12) (368 4) (378 3) (380 8) (392 12) (394 163) (395 52) (396 12) (408 7) (409 88) (410 29) (411 8) |
| 223 | 2847,9 | 16,9042 | 35 | (53 42) (65 12) (67 139) (68 129) (69 999) (70 48) (77 26) (79 61) (80 28) (81 516) (82 52) (91 36) (92 18) (93 104) (94 39) (95 145) (105 27) (106 9) (107 70) (108 18) (109 67) (119 20) (120 9) (121 94) (122 19) (123 62) (134 11) (135 33) (136 77) (137 68) (138 7) (149 29) (150 7) (161 11) (203 8) |
| 224 | 2863,2 | 16,9727 | 53 | (53 13) (54 25) (55 293) (56 201) (57 999) (58 39) (67 44) (68 39) (69 226) (70 99) (71 636) (72 36) (81 24) (82 39) (83 163) (84 52) (85 444) (86 28) (96 23) (97 122) (98 31) (99 128) (110 12) (111 58) (112 19) (113 70) (125 24) (126 12) (127 50) (138 4) (139 7) (140 7) (141 39) (153 6) (154 6) (155 21) (168 5) (169 19) (182 4) (183 16) (197 17) (211 16) (224 3) (225 12) (239 12) (253 10) (267 9) (295 7) (309 4) (364 8) (365 26) (366 8) (393 5) |
| 225 | 2888 | 17,0835 | 144 | (53 28) (54 19) (55 111) (58 9) (59 53) (60 7) (61 17) (62 2) (65 42) (66 46) (67 232) (68 19) (69 26) (70 7) (72 20) (73 999) (74 87) (75 189) (76 14) (77 125) (78 69) (79 350) (80 103) (81 71) (82 12) (84 14) (87 8) (89 24) (90 3) (91 254) (92 62) (93 129) (94 52) (95 57) (96 10) (101 50) (102 6) (103 766) (104 92) (105 151) (106 56) (107 45) (108 73) (109 17) (113 10) (115 27) (116 22) (117 114) (118 27) (119 88) (120 32) (121 26) (122 12) (123 6) (129 381) (130 64) (131 127) (132 31) (133 88) (134 27) (135 23) (136 6) (137 3) (141 6) (142 5) (143 18) (144 11) (145 58) (147 235) (148 50) (149 36) (150 5) (151 3) (155 6) (156 3) (157 18) (158 7) (159 25) (160 12) (161 19) (162 15) (163 11) (164 3) (165 2) (169 9) (170 4) (171 19) (172 6) (173 18) (174 11) (175 28) (176 10) (177 6) (183 4) (184 3) (185 9) (186 4) (187 9) (188 11) (189 21) (190 4) (191 25) (192 4) (195 2) (197 6) (198 2) (199 7) (200 10) (201 36) (202 9) (203 38) (204 7) (205 5) (213 5) (214 3) (215 7) (216 3) (217 28) (218 48) (219 28) (220 8) (221 4) (223 3) (225 2) (227 4) (228 3) (237 5) (240 3) (241 5) (242 3) (255 4) (256 3) (266 2) (283 2) (284 4) (285 1) (294 5) (295 3) (307 2) (321 2) (322 2) (430 2) (449 3) (450 1) |
| 226 | 2900,2 | 17,1379 | 53 | (53 10) (54 24) (55 253) (56 127) (57 999) (58 37) (68 23) (69 175) (70 114) (71 732) (72 37) (82 30) (83 139) (84 63) (85 472) (86 28) (96 13) (97 103) (98 34) (99 116) (100 5) (111 57) (112 29) (113 68) (125 21) (126 28) (127 49) (138 3) (139 9) (140 15) (141 36) (155 22) (168 7) (169 15) (183 14) (196 4) (197 12) (211 10) (224 3) (225 10) (239 6) (252 3) (253 6) (267 5) (280 2) (281 4) (295 4) (309 3) (323 3) (336 2) (379 10) (380 11) (381 3) |
| 227 | 2906,3 | 17,1653 | 72 | (53 44) (54 45) (55 376) (56 111) (58 66) (59 73) (66 29) (67 164) (68 156) (69 251) (70 116) (72 62) (73 999) (74 93) (75 116) (80 28) (81 169) (82 136) (83 99) (87 60) (88 31) (89 135) (90 12) (93 67) (95 108) (96 78) (97 61) (98 134) (99 37) (101 100) (102 22) (103 366) (104 25) (105 35) (109 61) (110 44) (115 25) (116 167) (117 273) (118 50) (119 52) (120 14) (121 18) (123 21) (124 25) (129 120) (130 32) (131 86) (132 17) (133 69) (137 24) (138 16) (144 14) (147 204) (148 31) (160 48) (161 73) (163 32) (169 31) (176 12) (189 30) (191 61) (203 28) (204 309) (205 67) (214 8) (217 110) (218 40) (271 17) (272 9) (320 10) (350 6) |
| 228 | 2917,3 | 17,2144 | 207 | (51 5) (53 27) (54 38) (55 172) (56 14) (57 28) (58 11) (59 45) (60 6) (61 14) (62 2) (63 2) (65 35) (66 48) (67 280) (68 36) (69 78) (70 6) (71 12) (72 22) (73 999) (74 83) (75 166) (76 11) (77 106) (78 70) (79 346) (80 183) (81 125) (82 27) (83 25) (84 20) (85 15) (87 7) (88 5) (89 23) (90 2) (91 249) (92 65) (93 153) (94 50) (95 79) (96 33) (97 25) (98 5) (99 8) (101 78) (102 9) (103 285) (104 54) (105 134) (106 62) (107 50) (108 16) (109 22) (110 8) (111 6) (113 6) (115 28) (116 36) (117 147) (118 31) (119 81) (120 32) (121 51) (122 13) (123 15) (124 6) (125 4) (127 4) (128 6) (129 315) (130 61) (131 146) (132 41) (133 126) (134 27) (135 36) (136 17) (137 10) (138 2) (141 6) (142 5) (143 16) (144 8) (145 60) (146 26) (147 318) (148 58) (149 58) (150 78) (151 15) (152 2) (155 6) (156 2) (157 21) (158 6) (159 23) (160 10) (161 26) (162 14) (163 18) (164 24) (165 6) (167 2) (169 10) (170 4) (171 13) (172 3) (173 21) (174 7) (175 48) (176 21) (177 20) (178 4) (179 3) (181 2) (182 1) (183 9) (184 2) (185 8) (186 2) (187 22) (188 20) (189 21) (190 12) (191 11) (195 2) (197 5) (198 2) (199 7) (200 2) (201 37) (202 18) (203 81) (204 18) (205 37) (206 9) (207 4) (209 3) (211 4) (212 1) (213 5) (214 2) (215 9) (216 4) (217 20) (218 15) (219 21) (220 4) (221 3) (223 1) (225 2) (227 5) (228 2) (229 5) (230 5) (231 3) (232 2) (235 1) (237 7) (238 2) (239 3) (240 2) (241 4) (242 2) (243 4) (244 6) (245 4) (255 1) (257 3) (258 4) (259 2) (265 1) (267 3) (268 4) (269 7) (275 1) (280 1) (281 2) (283 1) (285 4) (286 13) (287 5) (288 1) (293 2) (294 7) (295 3) (296 1) (303 2) (307 3) (308 1) (311 2) (312 1) (321 2) (329 10) (330 2) (347 2) (352 3) (353 1) (376 1) (419 16) (420 5) (421 1) (424 1) (432 3) (434 1) |
| 229 | 2931,6 | 17,278 | 12 | (56 159) (57 999) (71 708) (85 502) (86 29) (99 154) (111 108) (125 45) (138 12) (139 28) (154 51) (182 36) |
| 230 | 2937,2 | 17,3034 | 44 | (73 999) (74 91) (89 45) (100 24) (117 162) (126 132) (129 152) (132 37) (147 265) (160 154) (161 44) (169 59) (189 56) (190 18) (191 95) (204 551) (205 109) (206 49) (217 247) (218 51) (230 16) (231 33) (233 14) (243 60) (244 18) (245 21) (270 10) (271 66) (272 14) (274 10) (291 19) (305 25) (307 15) (319 45) (331 20) (361 558) (362 158) (363 76) (364 34) (390 13) (439 27) (440 10) (454 12) (480 18) |
| 231 | 2967,1 | 17,4371 | 40 | (53 76) (54 280) (55 999) (56 144) (65 64) (66 38) (67 828) (68 270) (69 540) (79 300) (80 310) (81 811) (82 515) (83 310) (93 168) (94 199) (95 573) (96 389) (97 226) (107 107) (108 117) (109 286) (110 97) (121 178) (122 76) (123 130) (124 54) (125 26) (135 176) (136 33) (137 39) (138 22) (150 22) (163 25) (174 34) (175 22) (210 17) (252 12) (291 12) (304 8) |
| 232 | 2975,8 | 17,4759 | 64 | (53 65) (54 129) (55 999) (56 357) (57 342) (63 11) (65 28) (66 22) (67 440) (68 569) (69 656) (70 142) (71 111) (77 29) (79 132) (80 87) (81 436) (82 416) (83 361) (84 48) (85 62) (91 51) (92 22) (93 74) (94 58) (95 346) (96 349) (97 273) (98 39) (106 13) (107 38) (108 76) (109 183) (110 109) (111 88) (119 26) (120 14) (121 46) (122 23) (123 85) (124 43) (125 26) (135 28) (136 15) (137 35) (138 28) (145 23) (151 12) (152 12) (159 19) (165 12) (166 13) (172 13) (174 11) (213 10) (214 6) (222 6) (250 15) (255 8) (353 7) (354 5) (368 18) (369 6) (424 5) |
| 233 | 2998,3 | 17,5763 | 126 | (53 13) (54 28) (55 310) (56 152) (57 999) (58 44) (65 3) (66 4) (67 54) (68 51) (69 196) (70 112) (71 641) (72 34) (79 5) (80 6) (81 34) (82 47) (83 150) (84 59) (85 446) (86 29) (87 1) (93 3) (94 4) (95 21) (96 35) (97 127) (98 39) (99 132) (100 10) (107 2) (109 9) (110 14) (111 63) (112 28) (113 80) (114 7) (121 3) (122 2) (123 5) (124 9) (125 29) (126 21) (127 54) (128 5) (136 1) (137 2) (138 6) (139 11) (140 15) (141 39) (142 4) (149 1) (151 1) (152 4) (153 7) (154 12) (155 31) (156 4) (166 3) (167 5) (168 10) (169 24) (170 3) (177 1) (180 2) (181 3) (182 8) (183 21) (184 3) (194 2) (195 2) (196 7) (197 17) (198 3) (208 1) (209 1) (210 6) (211 14) (212 2) (222 1) (223 1) (224 5) (225 12) (226 2) (236 1) (237 1) (238 4) (239 10) (240 2) (250 1) (251 1) (252 3) (253 8) (254 2) (266 3) (267 7) (268 1) (280 3) (281 6) (282 1) (294 2) (295 5) (296 1) (308 2) (309 4) (310 1) (322 2) (323 4) (324 1) (336 2) (337 3) (338 1) (350 1) (351 3) (352 1) (364 1) (365 3) (366 1) (378 1) (379 2) (380 1) (393 1) (422 3) (423 1) |
| 234 | 3007,6 | 17,6193 | 56 | (53 52) (54 128) (55 999) (56 293) (57 136) (63 11) (65 17) (66 20) (67 309) (68 440) (69 593) (70 110) (77 19) (79 68) (80 27) (81 333) (82 408) (83 323) (84 46) (93 30) (94 14) (95 264) (96 297) (97 280) (98 23) (109 148) (110 114) (111 97) (117 26) (119 16) (123 79) (124 57) (125 27) (137 36) (138 32) (139 8) (144 6) (145 11) (151 15) (152 20) (158 8) (165 7) (166 13) (172 11) (173 7) (180 7) (186 5) (194 13) (200 4) (208 8) (214 4) (228 8) (241 6) (250 18) (277 6) (424 6) |
| 235 | 3018,1 | 17,6679 | 36 | (54 39) (55 104) (57 170) (67 161) (70 33) (72 47) (73 999) (74 83) (75 356) (81 63) (84 73) (95 60) (96 212) (97 46) (98 42) (110 27) (116 147) (117 853) (118 74) (119 40) (129 88) (132 54) (133 36) (145 69) (157 16) (185 29) (186 14) (199 21) (292 15) (334 26) (376 30) (377 11) (451 82) (452 27) (466 7) (488 5) |
| 236 | 3027,7 | 17,7122 | 40 | (54 23) (55 193) (56 147) (57 999) (58 40) (69 166) (70 105) (71 686) (72 40) (73 259) (83 120) (84 45) (85 488) (86 25) (97 97) (98 34) (99 139) (111 92) (113 81) (124 11) (125 50) (127 47) (139 21) (141 36) (153 11) (155 30) (168 34) (169 26) (182 26) (183 21) (196 18) (197 14) (210 16) (211 12) (225 10) (238 9) (266 8) (309 6) (361 13) (488 3) |
| 237 | 3057,9 | 17,8519 | 44 | (54 12) (55 220) (56 190) (57 999) (58 37) (69 186) (70 111) (71 638) (72 34) (83 139) (84 54) (85 428) (86 28) (97 110) (98 34) (99 128) (111 52) (112 17) (113 71) (125 19) (126 12) (127 46) (139 10) (140 9) (141 36) (153 9) (154 7) (155 27) (168 7) (183 15) (197 13) (211 12) (225 12) (239 10) (253 7) (267 8) (281 6) (295 6) (309 7) (323 6) (392 7) (393 21) (394 6) (421 5) |
| 238 | 3094,1 | 18,0191 | 51 | (54 43) (55 372) (56 132) (57 999) (58 38) (67 112) (68 134) (69 259) (70 117) (71 731) (72 39) (81 87) (82 88) (83 184) (84 67) (85 483) (86 30) (94 20) (95 60) (96 77) (97 136) (98 30) (99 116) (109 31) (110 26) (111 62) (112 26) (113 66) (122 10) (123 12) (124 13) (125 25) (126 31) (127 45) (139 10) (140 14) (141 30) (154 9) (155 21) (169 15) (183 13) (196 5) (197 11) (211 11) (225 8) (239 6) (252 3) (253 6) (267 6) (295 4) (309 5) |
| 239 | 3103 | 18,0605 | 129 | (53 45) (54 33) (55 221) (59 46) (61 18) (65 52) (66 52) (67 311) (68 49) (69 135) (72 19) (73 999) (74 85) (75 231) (76 14) (77 143) (78 85) (79 444) (80 122) (81 164) (82 45) (83 68) (89 17) (91 358) (92 73) (93 217) (94 69) (95 146) (96 24) (97 42) (101 68) (102 10) (103 281) (104 48) (105 187) (106 71) (107 99) (108 102) (109 60) (110 12) (115 47) (116 36) (117 180) (118 39) (119 141) (120 50) (121 74) (122 18) (123 23) (128 15) (129 400) (130 78) (131 171) (132 59) (133 134) (134 41) (135 41) (137 11) (142 8) (143 50) (144 20) (145 95) (146 30) (147 241) (148 63) (149 47) (150 7) (155 19) (156 7) (157 34) (158 15) (159 50) (160 23) (161 34) (162 13) (163 20) (169 15) (170 7) (171 15) (173 31) (174 18) (175 29) (176 7) (177 11) (179 8) (181 7) (183 12) (185 18) (186 8) (187 16) (188 13) (189 18) (197 9) (198 5) (199 17) (200 8) (201 24) (202 12) (203 47) (205 26) (211 9) (213 18) (214 8) (215 18) (217 17) (219 25) (227 9) (228 5) (233 11) (237 7) (239 7) (241 10) (242 6) (255 15) (310 4) (312 3) (313 5) (314 11) (315 45) (316 11) (326 3) (339 22) (340 4) (354 37) (355 9) (429 5) (443 5) (444 17) (445 5) |
| 240 | 3126,4 | 18,1685 | 77 | (54 59) (55 461) (56 168) (57 999) (58 60) (65 52) (66 47) (67 356) (68 77) (69 279) (70 131) (71 621) (72 50) (73 839) (74 70) (75 131) (77 111) (78 62) (79 384) (80 126) (81 168) (82 73) (83 170) (84 59) (85 470) (91 272) (92 55) (93 165) (94 69) (95 125) (96 62) (97 184) (98 41) (99 145) (101 62) (103 237) (104 36) (105 139) (106 29) (107 46) (108 80) (109 45) (111 99) (113 78) (117 135) (118 38) (119 108) (120 34) (121 53) (122 17) (125 43) (129 289) (130 46) (131 124) (133 145) (134 28) (135 29) (145 48) (147 252) (148 57) (149 39) (154 85) (155 35) (161 22) (162 12) (168 24) (169 28) (171 21) (175 33) (176 21) (182 33) (183 29) (201 21) (203 41) (205 28) (323 13) (445 10) |
| 241 | 3180,1 | 18,4169 | 240 | (53 44) (54 12) (55 773) (56 50) (57 72) (59 34) (60 7) (61 32) (63 4) (65 30) (66 10) (67 268) (68 56) (69 999) (70 62) (71 18) (72 13) (73 833) (74 80) (75 486) (76 34) (77 107) (78 21) (79 271) (80 36) (81 359) (82 43) (83 44) (88 3) (89 15) (91 283) (92 48) (93 253) (94 54) (95 216) (96 18) (97 18) (99 11) (101 25) (102 4) (103 23) (104 13) (105 282) (106 49) (107 208) (108 39) (109 96) (110 15) (111 660) (112 53) (113 8) (114 3) (115 43) (116 19) (117 97) (118 31) (119 228) (120 97) (121 118) (122 20) (123 34) (124 6) (125 5) (127 10) (128 24) (129 679) (130 111) (131 160) (132 47) (133 183) (134 37) (135 62) (136 9) (137 16) (138 4) (139 4) (141 18) (142 21) (143 121) (144 35) (145 204) (146 53) (147 140) (148 23) (149 41) (150 8) (151 10) (155 45) (156 18) (157 67) (158 40) (159 186) (160 45) (161 92) (162 16) (163 28) (164 9) (165 12) (166 3) (167 8) (168 7) (169 28) (170 8) (171 47) (172 19) (173 66) (174 18) (175 27) (176 5) (177 15) (178 7) (179 9) (181 11) (182 9) (183 18) (184 7) (185 38) (186 14) (187 34) (188 11) (189 32) (190 6) (191 10) (193 10) (194 5) (195 10) (196 12) (197 24) (198 9) (199 45) (200 15) (201 32) (202 9) (203 13) (205 7) (208 9) (209 7) (211 22) (212 14) (213 72) (214 21) (215 90) (216 22) (217 20) (219 5) (220 4) (221 10) (223 8) (224 3) (225 14) (226 9) (227 21) (228 17) (229 22) (230 5) (231 8) (233 6) (234 3) (235 4) (236 53) (237 94) (238 21) (239 37) (240 14) (241 17) (242 7) (243 24) (244 7) (245 26) (246 6) (252 1) (253 49) (254 24) (255 198) (256 42) (257 15) (258 4) (259 3) (267 20) (268 5) (269 4) (271 19) (272 7) (273 8) (275 4) (277 8) (278 2) (281 7) (282 34) (283 9) (284 3) (285 3) (291 4) (295 10) (296 4) (297 4) (299 6) (309 6) (311 5) (312 2) (313 2) (315 4) (323 5) (324 11) (325 13) (326 42) (327 151) (328 38) (329 8) (330 4) (331 4) (337 4) (338 8) (340 2) (343 10) (344 8) (345 6) (346 2) (351 87) (352 27) (353 3) (357 3) (358 2) (365 4) (366 159) (367 48) (368 8) (372 21) (373 7) (374 3) (396 4) (414 2) (428 2) (441 25) (442 9) (443 2) (456 92) (457 33) (458 9) (459 2) |
| 242 | 3218,7 | 18,6176 | 267 | (53 36) (54 11) (55 374) (56 73) (57 371) (58 21) (59 43) (60 9) (61 42) (62 3) (63 5) (65 26) (66 8) (67 234) (68 34) (69 267) (70 39) (71 190) (72 25) (73 910) (74 82) (75 592) (76 42) (77 100) (78 20) (79 252) (80 32) (81 367) (82 38) (83 138) (84 13) (87 5) (88 3) (89 24) (90 3) (91 326) (92 60) (93 280) (94 54) (95 399) (96 35) (97 84) (98 6) (99 16) (101 46) (102 6) (103 47) (104 22) (105 365) (106 80) (107 311) (108 58) (109 195) (110 26) (111 55) (112 5) (113 13) (114 4) (115 61) (116 39) (117 138) (118 49) (119 352) (120 153) (121 270) (122 42) (123 79) (124 9) (125 23) (126 3) (127 17) (128 33) (129 999) (130 160) (131 210) (132 66) (133 214) (134 62) (135 127) (136 21) (137 36) (138 4) (139 9) (141 21) (142 25) (143 152) (144 51) (145 257) (146 70) (147 155) (148 44) (149 84) (150 12) (151 28) (152 5) (153 11) (154 6) (155 59) (156 19) (157 76) (158 40) (159 175) (160 104) (161 141) (162 32) (163 99) (164 13) (165 26) (166 5) (167 8) (168 9) (169 29) (170 9) (171 50) (172 20) (173 84) (174 32) (175 58) (176 11) (177 52) (178 9) (179 20) (180 5) (181 20) (182 13) (183 17) (184 7) (185 45) (186 14) (187 33) (188 10) (189 46) (191 25) (192 5) (193 26) (194 8) (195 14) (196 14) (197 22) (198 6) (199 46) (200 21) (201 42) (202 10) (203 67) (204 16) (205 30) (206 18) (209 6) (211 7) (212 4) (213 90) (214 27) (215 42) (216 13) (217 61) (218 12) (219 44) (220 13) (221 8) (222 2) (223 3) (225 3) (226 2) (227 22) (228 19) (229 25) (230 7) (231 17) (232 4) (233 42) (234 9) (235 6) (236 2) (239 6) (240 2) (241 18) (242 6) (243 7) (244 3) (245 25) (246 19) (247 103) (248 22) (249 4) (250 4) (251 2) (253 4) (254 4) (255 118) (256 27) (257 8) (258 2) (259 27) (260 17) (261 8) (262 2) (269 4) (271 5) (272 2) (273 19) (274 18) (275 38) (276 7) (283 10) (284 2) (285 4) (286 2) (287 6) (288 3) (289 3) (290 2) (291 6) (292 2) (297 5) (298 2) (299 6) (300 5) (301 25) (302 7) (303 4) (304 1) (311 9) (312 4) (313 10) (314 5) (315 4) (317 1) (325 7) (326 21) (327 28) (328 94) (329 365) (330 91) (331 11) (339 11) (340 17) (341 4) (342 5) (343 2) (345 6) (346 2) (353 170) (354 48) (355 7) (366 1) (367 8) (368 310) (369 93) (370 14) (371 2) (373 1) (387 2) (401 1) (416 3) (425 2) (429 2) (430 3) (443 47) (444 17) (445 5) (456 2) (457 2) (458 130) (459 48) (460 14) (461 2) |
| 243 | 3229 | 18,6773 | 116 | (53 74) (54 167) (55 999) (56 163) (61 29) (63 38) (65 31) (66 32) (67 636) (68 256) (69 611) (70 134) (73 376) (74 50) (75 710) (76 50) (77 110) (79 274) (80 121) (81 737) (82 412) (83 311) (88 16) (89 45) (91 194) (92 36) (93 248) (94 84) (95 582) (96 363) (97 223) (101 46) (104 34) (105 128) (106 112) (107 219) (108 79) (109 292) (110 160) (111 99) (116 28) (117 53) (119 72) (120 53) (121 163) (122 51) (123 148) (124 69) (129 176) (130 50) (131 186) (132 22) (135 114) (136 32) (137 68) (138 52) (140 76) (142 35) (144 30) (145 128) (146 35) (147 85) (148 49) (149 61) (151 27) (152 25) (154 71) (155 31) (157 20) (158 33) (159 73) (160 44) (161 67) (162 20) (163 31) (165 36) (172 31) (173 53) (174 39) (175 39) (176 12) (187 21) (188 15) (189 24) (199 16) (200 14) (201 47) (202 15) (214 10) (215 148) (216 79) (217 56) (228 15) (229 17) (230 29) (231 16) (241 12) (255 20) (256 11) (262 10) (305 31) (306 31) (329 48) (330 15) (353 23) (355 49) (356 13) (368 41) (369 18) (370 26) (403 19) (445 54) (446 20) (458 25) (460 36) (461 12) |
| 244 | 3255,3 | 18,8303 | 21 | (54 24) (56 195) (57 999) (71 775) (72 30) (85 591) (86 46) (97 130) (98 38) (99 165) (111 61) (112 32) (113 93) (127 45) (139 13) (140 15) (141 20) (315 9) (420 7) (421 25) (422 7) |
| 245 | 3260,9 | 18,8626 | 244 | (53 35) (54 6) (55 555) (57 118) (58 8) (59 24) (60 6) (61 27) (62 3) (63 3) (65 24) (66 8) (67 258) (68 27) (69 999) (70 73) (72 7) (73 607) (74 56) (75 403) (76 29) (77 86) (78 15) (79 211) (80 26) (81 301) (82 49) (83 259) (84 16) (88 3) (89 14) (91 232) (92 36) (93 209) (94 42) (95 167) (96 21) (97 19) (101 22) (102 4) (103 20) (104 12) (105 231) (106 42) (107 180) (108 34) (109 109) (110 12) (111 9) (114 2) (115 38) (116 16) (117 83) (118 23) (119 191) (120 79) (121 101) (122 16) (123 33) (124 10) (125 230) (126 23) (127 5) (128 22) (129 585) (130 93) (131 134) (132 37) (133 172) (134 33) (135 58) (136 8) (137 13) (141 14) (142 16) (143 100) (144 29) (145 172) (146 42) (147 126) (148 19) (149 32) (150 5) (151 9) (152 3) (153 5) (154 3) (155 36) (156 14) (157 64) (158 35) (159 164) (160 40) (161 76) (162 14) (163 22) (164 6) (165 8) (166 2) (168 4) (169 25) (170 8) (171 43) (172 16) (173 74) (174 16) (175 24) (176 5) (177 9) (178 6) (179 6) (180 2) (181 10) (182 8) (183 15) (184 5) (185 35) (186 12) (187 29) (188 9) (189 26) (190 5) (193 4) (194 3) (195 6) (196 10) (197 21) (198 9) (199 43) (200 12) (201 28) (202 6) (203 12) (205 3) (209 3) (210 2) (211 26) (212 13) (213 72) (214 21) (215 64) (216 17) (217 20) (218 5) (219 4) (221 2) (225 12) (226 9) (227 23) (228 21) (229 19) (230 4) (231 5) (233 3) (235 2) (237 2) (239 23) (240 14) (241 19) (242 7) (243 19) (244 3) (245 3) (247 2) (252 2) (253 56) (254 26) (255 183) (256 41) (257 35) (258 7) (259 12) (260 3) (267 17) (268 4) (269 4) (270 2) (271 29) (272 7) (273 2) (281 16) (282 29) (283 14) (284 3) (285 4) (286 3) (287 3) (288 1) (291 3) (295 13) (296 7) (297 3) (298 1) (302 1) (303 3) (304 1) (309 4) (310 2) (311 3) (312 2) (313 6) (315 2) (318 2) (325 2) (329 6) (330 5) (331 4) (337 20) (338 13) (339 11) (340 35) (341 70) (342 22) (343 20) (344 11) (345 6) (351 3) (352 6) (353 2) (354 2) (357 3) (358 2) (365 62) (366 18) (367 3) (371 3) (372 17) (373 6) (374 1) (380 137) (381 42) (382 6) (386 2) (428 1) (442 1) (455 20) (456 7) (457 2) (468 2) (469 1) (470 87) (471 34) (472 9) (473 2) |
| 246 | 3290,5 | 19,0344 | 114 | (53 61) (54 207) (55 999) (56 220) (57 527) (63 19) (65 29) (66 29) (67 475) (68 282) (69 635) (70 135) (71 343) (72 19) (77 26) (79 90) (80 42) (81 500) (82 470) (83 373) (84 56) (85 206) (86 14) (89 11) (90 6) (91 34) (92 6) (93 31) (94 16) (95 334) (96 327) (97 241) (98 28) (99 54) (104 15) (105 13) (106 7) (107 11) (108 6) (109 176) (110 95) (111 76) (112 16) (113 29) (116 7) (117 20) (118 8) (119 11) (123 90) (124 49) (125 23) (126 15) (130 19) (131 16) (132 10) (133 10) (137 43) (138 27) (139 8) (140 7) (141 14) (143 11) (144 48) (145 17) (146 18) (151 15) (152 20) (153 4) (155 9) (157 11) (158 40) (159 14) (160 16) (161 4) (165 7) (166 8) (171 10) (172 39) (173 11) (174 14) (185 7) (186 13) (187 8) (188 7) (197 5) (199 7) (200 19) (201 5) (202 9) (211 5) (213 6) (214 8) (215 4) (216 5) (225 4) (227 5) (228 20) (229 6) (230 8) (239 3) (241 6) (242 6) (243 4) (256 2) (269 11) (270 5) (271 4) (284 12) (285 3) (286 4) (379 2) (456 4) (458 8) (460 4) |
| 247 | 3318,5 | 19,1972 | 191 | (53 74) (54 46) (55 949) (56 128) (59 33) (60 11) (61 45) (65 40) (66 21) (67 460) (68 135) (69 999) (70 72) (73 957) (74 91) (75 689) (76 50) (77 127) (78 27) (79 364) (80 52) (81 659) (82 136) (83 376) (84 140) (89 32) (90 7) (91 410) (92 66) (93 369) (94 64) (95 476) (96 91) (97 276) (98 16) (101 43) (102 8) (103 55) (104 19) (105 400) (106 81) (107 346) (108 59) (109 242) (110 32) (111 60) (115 60) (116 23) (117 123) (118 42) (119 409) (120 140) (121 224) (122 38) (123 131) (124 32) (125 54) (126 11) (128 35) (129 962) (130 146) (131 220) (132 62) (133 220) (134 50) (135 117) (136 20) (137 69) (141 23) (142 24) (143 162) (144 61) (145 211) (146 64) (147 144) (148 21) (149 83) (150 19) (151 40) (152 14) (153 21) (155 54) (156 24) (157 96) (158 82) (159 212) (160 65) (161 133) (162 25) (163 68) (164 7) (165 18) (166 8) (169 46) (170 17) (171 66) (172 44) (173 93) (174 32) (175 65) (176 14) (177 25) (180 8) (181 20) (183 17) (184 11) (185 53) (186 23) (187 57) (188 18) (189 56) (190 12) (193 15) (195 15) (196 10) (197 45) (198 17) (199 56) (200 18) (201 37) (202 12) (203 36) (211 76) (212 27) (213 90) (214 33) (215 49) (216 8) (217 26) (225 23) (226 19) (227 40) (228 30) (229 34) (231 20) (239 33) (240 13) (241 22) (242 8) (243 42) (244 11) (245 18) (253 148) (254 46) (255 36) (256 23) (257 95) (258 23) (259 29) (267 23) (268 9) (269 7) (270 7) (271 18) (272 5) (281 133) (282 49) (283 26) (284 5) (285 8) (286 6) (296 160) (297 47) (298 8) (317 5) (318 5) (326 4) (337 8) (338 9) (339 12) (340 32) (341 98) (342 26) (345 9) (351 7) (352 8) (365 81) (366 29) (371 53) (372 32) (373 9) (380 88) (381 28) (386 150) (387 48) (388 11) (455 42) (456 17) (468 3) (470 45) (471 18) |
| 248 | 3323,9 | 19,2281 | 205 | (53 41) (54 29) (55 398) (56 30) (57 422) (58 22) (59 31) (61 38) (65 20) (67 265) (68 39) (69 184) (70 32) (71 417) (72 32) (73 834) (74 75) (75 630) (76 50) (77 99) (78 32) (79 290) (80 36) (81 462) (82 61) (83 141) (85 137) (86 16) (89 20) (91 327) (92 60) (93 283) (94 52) (95 423) (96 49) (97 121) (99 33) (101 28) (103 47) (104 32) (105 361) (106 79) (107 305) (108 58) (109 203) (110 24) (111 59) (115 48) (116 33) (117 121) (118 43) (119 302) (120 144) (121 273) (122 39) (123 92) (124 18) (125 20) (128 29) (129 999) (130 176) (131 233) (132 66) (133 215) (134 59) (135 136) (136 30) (137 48) (141 25) (142 31) (143 175) (144 59) (145 257) (146 74) (147 175) (148 43) (149 94) (150 10) (151 23) (153 18) (154 30) (155 78) (156 26) (157 83) (158 36) (159 175) (160 96) (161 142) (162 32) (163 97) (164 17) (165 27) (167 8) (168 16) (169 30) (170 11) (171 54) (172 19) (173 99) (174 34) (175 48) (176 9) (177 48) (178 10) (179 20) (181 21) (182 17) (183 21) (184 9) (185 48) (186 14) (187 36) (188 9) (189 46) (190 10) (191 25) (193 9) (195 10) (196 15) (197 21) (198 6) (199 51) (200 20) (201 44) (202 10) (203 60) (204 17) (205 18) (207 18) (209 8) (213 86) (214 27) (215 42) (216 7) (217 65) (218 14) (219 20) (220 15) (221 26) (222 5) (223 5) (227 25) (228 17) (229 23) (231 11) (233 27) (234 9) (239 8) (241 14) (245 18) (247 28) (248 7) (250 4) (255 106) (256 25) (259 31) (260 17) (261 64) (262 15) (273 19) (274 14) (275 7) (283 12) (287 11) (288 12) (289 24) (290 8) (291 6) (299 4) (301 5) (303 8) (315 20) (316 5) (325 6) (326 3) (327 7) (328 5) (329 4) (340 19) (342 77) (343 278) (344 75) (345 15) (353 10) (354 13) (356 4) (363 8) (364 3) (367 134) (368 41) (369 6) (379 13) (382 266) (383 86) (384 15) (430 4) (444 3) (457 40) (458 14) (459 4) (469 13) (472 111) (473 43) (474 12) (484 5) |
| 249 | 3354,4 | 19,4052 | 164 | (53 33) (54 13) (55 766) (56 55) (57 307) (59 21) (61 29) (65 25) (67 222) (68 31) (71 215) (72 22) (73 647) (74 65) (75 448) (76 30) (77 88) (79 217) (80 31) (81 449) (82 50) (83 999) (84 92) (85 94) (86 10) (91 239) (92 40) (93 237) (94 47) (95 198) (96 43) (97 170) (99 29) (101 23) (103 20) (105 246) (106 41) (107 183) (108 32) (109 101) (110 22) (111 26) (113 17) (115 31) (116 14) (117 81) (118 20) (119 192) (120 82) (121 101) (122 21) (123 45) (124 11) (125 12) (127 13) (128 18) (129 612) (130 95) (131 139) (132 37) (133 189) (134 33) (135 55) (136 8) (137 29) (142 16) (143 98) (144 29) (145 170) (146 40) (147 108) (148 20) (149 28) (151 11) (155 39) (156 15) (157 68) (158 36) (159 163) (160 39) (161 75) (162 12) (163 25) (164 5) (169 31) (170 7) (171 42) (172 15) (173 78) (174 15) (175 20) (181 13) (182 10) (183 20) (184 7) (185 36) (186 12) (187 31) (188 9) (189 23) (196 8) (197 22) (198 10) (199 43) (200 11) (201 29) (202 6) (203 11) (211 28) (212 16) (213 76) (214 22) (215 52) (216 15) (225 14) (226 8) (227 24) (228 25) (229 19) (239 23) (240 12) (241 20) (243 16) (253 56) (254 27) (255 180) (256 38) (257 30) (267 15) (271 30) (272 8) (273 9) (281 16) (282 23) (283 17) (284 4) (295 14) (296 9) (309 13) (310 4) (330 7) (342 7) (344 8) (351 50) (352 19) (353 12) (354 33) (355 54) (356 14) (365 7) (366 6) (371 5) (372 11) (379 56) (380 18) (386 5) (394 136) (395 41) (396 6) (469 20) (470 8) (484 103) (485 38) (486 11) |
| 250 | 3415,9 | 19,792 | 302 | (53 34) (54 10) (55 458) (56 41) (57 379) (58 19) (59 32) (60 7) (61 36) (62 3) (63 4) (65 23) (66 8) (67 225) (68 33) (69 329) (70 32) (71 176) (72 22) (73 781) (74 73) (75 519) (76 38) (77 83) (78 16) (79 226) (80 28) (81 348) (82 34) (83 138) (84 16) (85 138) (86 10) (87 6) (88 3) (89 20) (90 3) (91 279) (92 52) (93 263) (94 53) (95 361) (96 32) (97 115) (98 13) (99 32) (100 4) (101 35) (102 5) (103 30) (104 17) (105 335) (106 74) (107 304) (108 58) (109 186) (110 27) (111 71) (112 7) (113 15) (114 3) (115 52) (116 27) (117 124) (118 42) (119 322) (120 157) (121 262) (122 43) (123 85) (124 11) (125 28) (126 2) (127 14) (128 28) (129 999) (130 158) (131 204) (132 62) (133 198) (134 49) (135 119) (136 22) (137 41) (138 7) (139 14) (140 2) (141 22) (142 24) (143 151) (144 48) (145 250) (146 67) (147 149) (148 43) (149 81) (150 12) (151 24) (152 4) (153 9) (154 6) (155 59) (156 20) (157 79) (158 47) (159 183) (160 96) (161 136) (162 31) (163 91) (164 14) (165 20) (166 4) (167 7) (168 9) (169 32) (170 10) (171 50) (172 24) (173 102) (174 33) (175 58) (176 11) (177 41) (178 8) (179 11) (180 4) (181 18) (182 12) (183 18) (184 7) (185 42) (186 16) (187 37) (188 12) (189 42) (190 8) (191 21) (192 4) (193 10) (194 5) (195 12) (196 14) (197 26) (198 10) (199 47) (200 19) (201 40) (202 10) (203 50) (204 9) (205 12) (206 2) (207 9) (208 3) (209 5) (210 2) (212 12) (213 88) (214 31) (215 51) (216 12) (217 55) (218 10) (219 12) (220 3) (221 12) (222 3) (223 2) (225 9) (227 31) (228 24) (229 28) (230 6) (231 16) (232 3) (233 12) (234 10) (235 12) (236 2) (237 1) (239 13) (240 6) (241 21) (242 8) (243 15) (244 5) (246 6) (247 21) (248 6) (249 4) (250 4) (251 3) (252 1) (254 14) (255 116) (256 33) (258 8) (259 23) (260 5) (261 21) (262 4) (263 2) (267 6) (268 4) (269 7) (270 3) (271 6) (272 2) (273 18) (274 13) (275 54) (276 12) (277 2) (283 15) (284 5) (285 4) (286 2) (287 14) (288 11) (289 6) (290 3) (291 5) (292 1) (295 2) (298 4) (299 3) (300 2) (301 10) (302 12) (303 20) (304 6) (305 1) (311 2) (312 1) (313 2) (314 2) (315 4) (316 2) (317 2) (318 2) (325 3) (326 1) (327 5) (328 4) (329 20) (330 6) (331 2) (337 1) (339 5) (340 3) (341 5) (342 7) (343 12) (344 6) (345 7) (346 2) (351 1) (352 2) (353 8) (354 21) (355 35) (356 70) (357 223) (358 62) (359 8) (365 1) (367 10) (368 13) (369 3) (373 3) (374 1) (381 111) (382 33) (383 5) (388 9) (389 2) (393 2) (396 238) (397 76) (398 12) (399 1) (429 1) (444 2) (445 1) (453 1) (457 2) (458 2) (459 1) (469 10) (470 4) (471 33) (472 12) (473 3) (474 1) (483 1) (484 11) (485 6) (486 97) (487 38) (488 10) (489 2) |
| 251 | 3416,2 | 19,7945 | 305 | (53 35) (54 10) (55 474) (56 42) (57 370) (58 18) (59 32) (60 8) (61 36) (62 3) (63 4) (65 23) (66 8) (67 229) (68 34) (69 339) (70 33) (72 21) (73 790) (74 74) (75 525) (76 38) (77 84) (78 16) (79 229) (80 28) (81 351) (82 35) (83 142) (84 16) (85 135) (86 10) (87 6) (88 3) (89 21) (90 3) (91 281) (92 53) (93 265) (94 53) (95 362) (96 32) (97 118) (98 13) (99 32) (100 4) (101 35) (102 5) (103 30) (104 17) (105 336) (106 74) (107 305) (108 58) (109 186) (110 27) (111 70) (112 7) (113 15) (114 3) (115 52) (116 27) (117 124) (118 43) (119 324) (120 157) (121 261) (122 43) (123 86) (124 11) (125 28) (126 2) (127 14) (128 28) (129 999) (130 158) (131 204) (132 62) (133 198) (134 49) (135 119) (136 22) (137 42) (138 7) (139 14) (140 2) (141 22) (142 24) (143 151) (144 48) (145 249) (146 67) (147 149) (148 42) (149 80) (150 12) (151 24) (152 4) (153 9) (154 6) (155 59) (156 20) (157 79) (158 48) (159 184) (160 95) (161 136) (162 30) (163 90) (164 14) (165 20) (166 4) (167 7) (168 9) (169 32) (170 10) (171 51) (172 25) (173 102) (174 33) (175 58) (176 11) (177 41) (178 8) (179 11) (180 4) (181 18) (182 12) (183 18) (184 7) (185 42) (186 16) (187 37) (188 13) (189 42) (190 8) (191 21) (192 4) (193 10) (194 5) (195 12) (196 14) (197 26) (198 10) (199 47) (200 19) (201 40) (202 10) (203 50) (204 9) (205 11) (206 2) (207 9) (208 3) (209 5) (210 2) (212 13) (213 89) (214 31) (215 51) (216 12) (217 54) (218 10) (219 11) (220 3) (221 12) (222 3) (223 2) (225 10) (226 8) (227 31) (228 24) (229 29) (230 6) (231 16) (232 4) (233 12) (234 10) (235 11) (236 2) (237 1) (239 13) (240 6) (241 21) (242 8) (243 16) (244 5) (247 21) (248 7) (249 4) (250 4) (251 2) (252 1) (253 34) (254 15) (255 114) (256 33) (258 9) (259 23) (260 5) (261 20) (262 4) (263 2) (267 7) (268 4) (269 7) (270 4) (271 6) (272 2) (273 18) (274 13) (275 51) (276 11) (277 2) (283 15) (284 4) (285 4) (286 2) (287 14) (288 10) (289 6) (290 3) (291 5) (292 1) (295 2) (297 28) (298 5) (299 3) (300 2) (301 10) (302 12) (303 19) (304 6) (305 1) (311 2) (312 1) (313 2) (314 2) (315 4) (316 2) (317 3) (318 2) (325 3) (326 1) (327 5) (328 4) (329 19) (330 6) (331 2) (337 1) (339 5) (340 3) (341 5) (342 7) (343 13) (344 6) (345 7) (346 2) (351 1) (352 2) (353 8) (354 21) (355 36) (356 68) (357 214) (358 59) (359 8) (365 1) (367 9) (368 12) (369 3) (371 24) (372 10) (373 3) (374 1) (379 15) (380 4) (381 107) (382 32) (383 5) (384 1) (387 36) (388 9) (389 2) (393 2) (394 11) (398 12) (399 1) (429 1) (444 2) (445 1) (453 1) (457 2) (458 2) (469 10) (470 4) (471 31) (472 12) (473 3) (474 1) (483 1) (484 12) (485 6) (488 10) (489 2) |
| 252 | 3416,8 | 19,799 | 267 | (53 38) (54 11) (55 543) (56 45) (59 33) (60 8) (61 38) (62 3) (63 4) (65 25) (66 8) (67 245) (68 38) (69 385) (70 35) (72 20) (73 832) (74 78) (75 546) (76 39) (77 87) (78 18) (79 241) (80 30) (81 363) (82 37) (83 159) (84 17) (87 6) (88 3) (89 21) (90 3) (91 290) (92 54) (93 275) (94 55) (95 369) (96 32) (97 133) (98 15) (99 29) (100 3) (101 36) (102 5) (103 30) (104 17) (105 342) (106 74) (107 310) (108 58) (109 189) (110 27) (111 70) (112 6) (113 15) (114 3) (115 53) (116 27) (117 125) (118 43) (119 335) (120 157) (121 260) (122 42) (123 87) (124 11) (125 27) (127 14) (128 29) (129 999) (130 157) (131 205) (132 62) (133 196) (134 49) (135 118) (136 21) (137 43) (138 7) (139 13) (141 23) (142 24) (143 150) (144 48) (145 246) (146 66) (147 150) (148 41) (149 78) (150 11) (151 24) (152 4) (153 8) (154 6) (155 58) (156 21) (157 81) (158 51) (159 187) (160 92) (161 136) (162 29) (163 84) (164 13) (165 19) (166 4) (168 9) (169 34) (170 11) (171 52) (172 27) (173 102) (174 34) (175 58) (176 11) (177 39) (178 8) (179 11) (180 4) (181 18) (182 12) (183 19) (184 7) (185 43) (186 17) (187 39) (188 13) (189 42) (190 8) (191 19) (192 4) (193 9) (194 4) (195 12) (196 14) (197 27) (198 10) (199 48) (200 18) (201 41) (202 10) (203 48) (204 9) (205 10) (207 9) (208 3) (209 5) (210 2) (211 45) (212 17) (213 88) (214 31) (215 50) (216 12) (218 9) (219 11) (220 3) (221 11) (222 3) (223 2) (225 12) (226 10) (227 34) (228 26) (229 30) (230 6) (231 15) (232 4) (233 11) (234 9) (235 10) (236 2) (237 2) (239 15) (240 8) (241 21) (242 8) (243 19) (244 5) (248 6) (249 4) (250 4) (251 2) (252 2) (253 44) (254 18) (255 106) (256 33) (257 50) (258 11) (259 21) (260 4) (262 4) (263 1) (267 8) (268 5) (269 6) (270 4) (271 7) (272 3) (273 18) (281 83) (282 23) (283 15) (284 4) (285 4) (286 2) (289 5) (290 3) (291 4) (292 1) (295 3) (296 125) (297 35) (298 6) (299 3) (300 2) (301 9) (302 11) (304 6) (305 1) (309 1) (312 1) (313 2) (315 4) (316 2) (317 3) (318 3) (325 2) (327 5) (328 3) (330 5) (331 2) (337 1) (339 5) (340 3) (342 8) (343 15) (344 7) (345 6) (346 2) (351 2) (352 3) (353 8) (354 20) (355 38) (365 1) (366 1) (371 30) (372 13) (373 4) (374 1) (379 19) (380 6) (386 128) (387 43) (388 11) (389 2) (393 2) (394 14) (399 1) (453 1) (469 12) (470 5) (483 1) (484 14) (485 7) |
| 253 | 3432 | 19,9159 | 305 | (50 3) (51 9) (53 59) (54 19) (55 992) (56 81) (57 112) (58 9) (59 38) (60 8) (61 42) (62 4) (63 6) (64 1) (65 33) (66 12) (67 318) (68 64) (69 653) (70 67) (71 52) (72 17) (73 999) (74 94) (75 598) (76 43) (77 106) (78 20) (79 288) (80 36) (81 409) (82 56) (83 270) (84 23) (85 35) (86 3) (88 4) (89 22) (90 3) (91 343) (92 60) (93 327) (94 62) (95 393) (96 39) (97 172) (98 26) (99 20) (100 2) (101 42) (102 6) (103 35) (104 19) (105 399) (106 81) (107 337) (108 61) (109 206) (110 32) (111 61) (112 8) (113 15) (114 3) (115 61) (116 27) (117 144) (118 52) (119 429) (120 163) (121 244) (122 42) (123 96) (124 13) (125 25) (126 6) (127 16) (128 27) (129 905) (130 145) (131 231) (132 75) (133 220) (134 53) (135 117) (136 20) (137 47) (138 10) (139 17) (141 31) (142 29) (143 168) (144 60) (145 258) (146 65) (147 169) (148 37) (149 69) (150 10) (151 18) (152 5) (153 9) (154 6) (155 58) (156 36) (157 108) (158 102) (159 229) (160 75) (161 134) (162 25) (163 57) (164 10) (165 15) (166 4) (167 10) (168 12) (169 63) (170 19) (171 80) (172 52) (173 117) (174 43) (175 68) (176 18) (177 22) (178 5) (179 8) (180 4) (181 21) (182 17) (183 36) (184 18) (185 69) (186 32) (187 59) (188 26) (189 43) (190 9) (191 10) (192 2) (193 6) (194 6) (195 14) (196 22) (197 53) (198 24) (199 79) (200 29) (201 50) (202 19) (203 31) (204 6) (205 5) (206 1) (207 6) (208 3) (209 6) (210 4) (211 193) (212 59) (213 126) (214 55) (215 50) (216 11) (217 26) (218 5) (219 6) (220 2) (221 4) (222 2) (223 3) (224 2) (225 41) (226 38) (227 84) (228 56) (229 49) (230 10) (231 12) (232 3) (233 6) (234 2) (235 4) (236 2) (237 3) (238 2) (239 37) (240 25) (241 30) (242 14) (243 32) (244 9) (245 7) (246 2) (247 5) (248 6) (249 3) (250 2) (251 1) (253 114) (254 47) (255 103) (256 58) (257 160) (258 32) (259 10) (260 2) (261 2) (262 2) (263 1) (266 1) (267 21) (268 19) (269 8) (270 11) (271 8) (272 2) (273 13) (274 3) (275 2) (279 1) (280 1) (281 339) (282 83) (283 17) (284 3) (285 4) (286 1) (287 3) (288 2) (289 4) (290 2) (291 1) (295 8) (296 432) (297 107) (298 13) (299 3) (300 2) (301 16) (302 9) (303 10) (304 9) (305 2) (309 2) (310 1) (311 1) (313 1) (315 2) (316 2) (317 9) (318 8) (319 2) (325 1) (327 3) (329 3) (330 6) (331 3) (332 1) (337 2) (339 2) (341 1) (342 10) (343 28) (344 15) (345 5) (346 1) (351 3) (352 4) (353 6) (354 8) (355 29) (356 8) (357 5) (358 3) (359 1) (365 2) (366 2) (371 99) (372 38) (373 10) (374 2) (379 31) (380 9) (381 1) (385 1) (386 416) (387 133) (388 36) (389 6) (393 5) (394 16) (395 5) (396 1) (451 1) (455 1) (469 28) (470 11) (471 3) (472 1) (482 1) (483 3) (484 26) (485 10) (486 3) |
| 254 | 3656,4 | 21,638 | 213 | (53 11) (55 82) (56 10) (57 999) (58 43) (65 9) (66 3) (67 10) (69 19) (71 5) (73 8) (74 4) (77 18) (78 7) (79 22) (81 5) (83 6) (84 4) (89 3) (90 2) (91 147) (92 12) (93 11) (95 9) (99 3) (102 3) (103 15) (104 10) (105 75) (106 9) (107 32) (108 3) (109 3) (115 56) (116 23) (117 77) (118 14) (119 65) (120 8) (121 16) (122 2) (127 10) (128 39) (129 48) (130 15) (131 77) (132 28) (133 28) (134 7) (135 19) (141 14) (142 14) (143 31) (144 11) (145 20) (146 6) (147 124) (148 18) (149 23) (150 2) (153 3) (155 4) (156 3) (157 15) (158 7) (159 50) (160 7) (161 12) (162 2) (163 22) (164 2) (165 2) (169 4) (171 6) (172 4) (173 17) (174 5) (175 77) (176 10) (178 19) (179 17) (180 2) (181 2) (183 2) (186 1) (187 23) (188 7) (189 13) (190 9) (191 173) (192 26) (193 6) (194 6) (195 4) (197 21) (198 2) (203 3) (204 8) (205 8) (206 17) (208 8) (209 5) (210 3) (211 4) (213 4) (214 1) (215 11) (216 2) (217 6) (219 11) (220 3) (221 2) (222 17) (223 4) (225 10) (229 2) (233 2) (235 15) (236 2) (237 28) (238 4) (239 2) (249 2) (251 16) (252 3) (253 43) (254 6) (255 2) (263 14) (264 2) (265 3) (267 3) (271 5) (273 6) (274 2) (275 1) (277 2) (287 2) (288 1) (289 14) (290 2) (291 54) (292 9) (293 2) (301 1) (302 7) (303 6) (304 1) (305 15) (306 2) (307 3) (308 7) (309 3) (313 1) (316 381) (321 4) (327 2) (329 9) (330 2) (331 11) (332 2) (339 3) (340 1) (343 1) (345 6) (347 6) (361 8) (362 2) (363 2) (367 48) (368 11) (369 2) (376 1) (379 5) (381 8) (382 2) (383 1) (385 3) (387 5) (388 1) (401 4) (402 1) (403 9) (404 2) (407 9) (408 3) (417 1) (421 3) (423 35) (424 9) (425 2) (437 9) (438 3) (439 1) (441 5) (443 12) (444 3) (457 2) (458 1) (459 13) (460 4) (461 1) (463 5) (464 2) (474 2) (475 1) (477 2) (479 28) (480 9) (481 2) (493 11) (494 4) (495 1) |
| 255 | 978,8 | 4,1473 | 28 | (53 49) (55 131) (56 340) (57 78) (60 53) (61 80) (66 59) (73 991) (74 152) (75 999) (76 87) (81 27) (85 96) (86 355) (88 138) (92 16) (103 80) (104 31) (110 43) (114 194) (115 28) (131 57) (132 249) (133 65) (138 24) (147 133) (151 255) (152 47) |
| 256 | 985,5 | 4,2091 | 27 | (56 116) (57 36) (58 219) (60 74) (69 872) (78 383) (90 33) (96 32) (97 69) (98 22) (106 85) (108 29) (110 62) (112 20) (114 32) (126 999) (127 428) (139 183) (140 337) (141 15) (155 200) (156 11) (183 9) (189 11) (222 18) (252 10) (263 15) |
| 257 | 998,3 | 4,3278 | 15 | (53 22) (55 181) (56 215) (57 999) (67 13) (70 145) (71 353) (83 15) (84 85) (85 211) (98 56) (99 45) (113 22) (142 43) (146 16) |
| 258 | 1053 | 4,9932 | 51 | (52 14) (55 25) (58 31) (59 77) (60 7) (61 18) (66 95) (70 6) (72 24) (73 999) (74 92) (75 115) (76 13) (77 4) (82 1) (87 26) (88 56) (94 5) (101 24) (102 24) (103 17) (104 4) (105 4) (115 13) (116 7) (117 753) (118 85) (119 21) (129 16) (131 43) (132 6) (133 88) (134 9) (135 7) (147 967) (148 151) (149 78) (150 6) (175 6) (176 2) (190 121) (191 146) (192 32) (193 12) (194 2) (203 6) (204 1) (205 2) (219 41) (220 7) (221 6) |
| 259 | 1063,6 | 5,0796 | 57 | (53 4) (54 1) (55 10) (56 4) (57 9) (58 44) (59 49) (60 18) (61 150) (62 7) (63 8) (70 4) (71 5) (72 32) (73 999) (74 96) (75 415) (76 31) (77 20) (81 8) (87 5) (88 10) (89 6) (90 3) (91 14) (92 2) (101 12) (102 1) (103 3) (104 5) (105 35) (106 4) (107 124) (108 9) (109 10) (110 1) (116 6) (117 41) (118 4) (119 181) (120 18) (121 15) (122 1) (132 3) (134 39) (135 18) (136 5) (137 1) (163 133) (164 15) (165 12) (166 1) (178 116) (179 14) (180 10) (181 1) (184 1) |
| 260 | 1036,2 | 4,8567 | 36 | (56 96) (57 488) (70 115) (71 415) (73 800) (74 53) (84 74) (85 277) (112 20) (113 46) (127 41) (163 15) (178 8) (189 20) (190 6) (191 99) (192 18) (193 27) (194 5) (207 999) (208 198) (209 119) (210 17) (211 4) (247 18) (248 4) (263 10) (264 3) (265 8) (279 24) (280 6) (281 5) (295 164) (296 52) (297 32) (298 5) |
| 261 | 1140,8 | 5,7081 | 25 | (53 30) (59 98) (67 15) (68 23) (70 64) (73 158) (86 32) (95 34) (100 302) (101 31) (110 11) (116 54) (123 29) (124 7) (125 90) (127 37) (133 24) (137 13) (147 67) (153 999) (154 132) (155 41) (168 174) (169 50) (170 10) |
| 262 | 1215 | 6,3121 | 89 | (52 18) (53 12) (54 9) (55 59) (56 7) (57 15) (59 74) (60 11) (61 22) (65 2) (66 174) (67 22) (68 5) (69 7) (70 8) (71 9) (72 36) (73 695) (74 62) (75 139) (76 9) (77 7) (81 5) (82 6) (83 74) (84 10) (86 3) (87 7) (89 6) (94 27) (95 3) (97 5) (98 7) (99 22) (100 5) (101 4) (102 3) (103 16) (104 3) (105 7) (108 15) (109 3) (112 6) (113 130) (114 17) (115 26) (116 4) (117 13) (118 2) (119 10) (127 5) (128 18) (129 26) (130 6) (131 36) (132 6) (133 107) (134 14) (135 10) (140 4) (141 3) (143 6) (147 999) (148 153) (149 118) (150 12) (151 5) (155 2) (156 76) (157 24) (158 4) (159 3) (163 1) (177 4) (187 2) (191 6) (202 1) (215 4) (229 4) (230 7) (231 286) (232 58) (233 26) (234 3) (245 1) (246 2) (272 1) (371 3) (372 1) |
| 263 | 1251,2 | 6,6064 | 15 | (55 229) (56 111) (57 993) (69 142) (70 252) (71 999) (83 30) (84 119) (85 407) (110 98) (112 60) (113 104) (128 26) (155 121) (228 28) |
| 264 | 1274,1 | 6,7933 | 48 | (58 27) (59 68) (60 19) (61 53) (62 3) (72 30) (73 999) (74 128) (75 441) (76 36) (77 22) (80 61) (87 20) (88 37) (89 21) (90 5) (95 5) (101 20) (102 30) (103 109) (104 11) (105 10) (110 3) (117 182) (118 43) (119 11) (125 1) (130 58) (131 55) (135 4) (145 20) (146 59) (149 15) (150 2) (159 45) (161 2) (188 14) (189 5) (202 13) (203 5) (204 4) (206 6) (216 5) (217 3) (218 3) (220 7) (221 3) (235 4) |
| 265 | 1290,2 | 6,9239 | 79 | (52 2) (53 2) (54 1) (55 14) (56 6) (57 16) (58 22) (59 94) (60 9) (61 15) (62 1) (63 1) (66 7) (67 2) (70 7) (71 15) (73 999) (74 86) (75 96) (76 5) (77 3) (83 1) (85 11) (86 1) (87 9) (88 7) (89 42) (90 4) (91 2) (97 1) (99 5) (101 53) (103 186) (104 18) (105 14) (106 1) (107 1) (111 1) (113 7) (115 20) (117 287) (118 29) (119 30) (120 3) (121 2) (129 51) (131 52) (133 222) (134 30) (135 20) (136 2) (143 1) (145 2) (147 675) (148 103) (149 73) (150 8) (151 3) (159 5) (160 1) (163 12) (164 2) (165 1) (173 1) (175 40) (176 7) (177 20) (178 3) (179 2) (189 9) (191 39) (192 7) (203 32) (204 44) (217 16) (221 4) (222 1) (263 1) (293 6) |
| 266 | 1305,9 | 7,052 | 20 | (55 222) (56 157) (57 999) (68 26) (69 101) (70 199) (71 969) (72 53) (82 30) (83 57) (84 84) (85 430) (86 31) (99 77) (112 43) (113 86) (126 30) (127 59) (153 21) (155 40) |
| 267 | 1315,3 | 7,1285 | 67 | (54 5) (55 35) (56 89) (57 305) (58 21) (59 45) (60 11) (61 31) (62 2) (66 21) (69 19) (70 31) (71 116) (73 999) (74 121) (75 258) (80 35) (83 9) (84 18) (85 72) (86 15) (87 52) (98 9) (99 17) (100 22) (101 29) (102 20) (103 14) (104 5) (112 7) (113 12) (114 30) (115 28) (116 23) (117 437) (118 46) (119 20) (126 4) (127 11) (128 7) (129 6) (130 301) (131 101) (132 92) (133 34) (134 6) (140 2) (144 3) (146 124) (147 175) (148 36) (149 15) (155 6) (176 4) (177 2) (202 3) (203 5) (204 13) (205 5) (206 1) (219 134) (220 33) (221 13) (230 2) (248 13) (249 3) (250 1) |
| 268 | 1330,4 | 7,2517 | 99 | (53 6) (54 3) (55 104) (56 50) (57 38) (58 24) (59 52) (60 10) (61 30) (62 2) (63 1) (65 1) (66 3) (67 5) (68 3) (69 35) (70 18) (71 21) (72 28) (73 654) (74 66) (76 18) (77 12) (81 2) (82 4) (83 17) (84 15) (85 25) (86 53) (87 9) (88 4) (89 5) (95 1) (97 5) (98 1) (99 5) (100 25) (101 10) (102 4) (103 6) (104 1) (105 3) (110 1) (111 13) (112 3) (113 9) (114 2) (115 12) (116 20) (117 17) (118 2) (119 5) (120 1) (125 3) (126 2) (128 3) (129 70) (130 18) (131 28) (132 5) (133 59) (134 8) (135 5) (136 1) (143 5) (144 3) (145 6) (146 2) (147 999) (148 161) (149 88) (150 9) (151 2) (154 1) (156 2) (157 3) (158 4) (159 1) (161 1) (162 1) (163 2) (172 37) (173 22) (177 3) (190 1) (203 3) (204 1) (205 1) (217 2) (218 7) (219 2) (220 1) (247 75) (248 45) (249 14) (250 5) (251 1) (262 3) (263 1) |
| 269 | 1340,3 | 7,3318 | 50 | (53 56) (54 20) (55 392) (56 199) (57 999) (58 39) (65 13) (66 5) (67 56) (68 41) (69 379) (70 285) (71 902) (72 49) (77 9) (81 17) (82 32) (83 160) (84 240) (85 594) (86 46) (95 12) (97 48) (98 29) (99 87) (100 7) (109 6) (110 15) (111 112) (112 54) (113 76) (114 6) (125 23) (126 41) (127 62) (128 6) (140 4) (141 28) (153 7) (154 18) (155 38) (156 4) (166 4) (169 17) (170 3) (197 3) (212 2) (240 32) (241 7) (242 3) |
| 270 | 1350,7 | 7,4168 | 27 | (55 282) (56 163) (57 999) (65 21) (68 23) (69 134) (70 232) (71 942) (83 35) (84 158) (85 532) (86 32) (98 21) (99 102) (111 21) (112 38) (113 85) (126 37) (127 73) (155 23) (193 30) (325 25) (326 7) (341 60) (342 21) (429 23) (430 9) |
| 271 | 1358,8 | 7,4827 | 27 | (53 50) (55 265) (56 140) (57 883) (69 98) (70 255) (71 999) (72 77) (84 136) (85 481) (99 101) (100 59) (112 30) (113 92) (140 14) (141 167) (147 56) (169 19) (215 82) (243 19) (244 12) (325 10) (341 26) (342 7) (343 8) (429 9) (431 3) |
| 272 | 1382,3 | 7,6743 | 56 | (60 12) (61 22) (66 20) (72 32) (86 39) (87 62) (102 48) (103 35) (118 27) (119 67) (120 10) (131 205) (132 95) (133 137) (134 22) (135 13) (146 91) (147 999) (148 165) (149 79) (150 8) (160 242) (161 41) (162 19) (175 11) (176 82) (177 17) (178 7) (188 179) (189 41) (190 20) (191 14) (193 11) (207 70) (208 15) (209 7) (221 336) (222 76) (223 42) (224 7) (241 19) (242 6) (249 21) (250 6) (254 5) (256 3) (262 5) (263 28) (264 17) (265 6) (278 213) (279 54) (280 25) (293 102) (294 24) (295 12) |
| 273 | 1418,4 | 7,9678 | 56 | (53 24) (54 21) (55 200) (56 165) (57 999) (58 42) (65 4) (66 2) (67 20) (68 15) (69 87) (70 117) (71 597) (72 31) (73 10) (77 3) (79 4) (81 3) (82 15) (83 45) (84 59) (85 360) (86 22) (87 1) (95 1) (96 6) (97 30) (98 43) (99 70) (100 5) (110 2) (111 11) (112 32) (113 31) (114 2) (125 3) (126 22) (127 20) (128 2) (140 14) (141 15) (142 1) (147 7) (148 1) (154 6) (155 9) (156 1) (168 2) (169 4) (198 18) (199 3) (221 7) (222 2) (223 1) (267 2) (282 1) |
| 274 | 1484,7 | 8,5075 | 35 | (55 295) (56 153) (57 999) (67 36) (68 34) (69 176) (70 196) (71 898) (72 55) (73 793) (74 71) (83 57) (84 80) (85 557) (99 177) (111 63) (112 47) (113 105) (125 22) (127 49) (133 59) (141 34) (143 76) (147 294) (148 44) (149 38) (155 76) (203 21) (215 131) (216 32) (217 459) (218 99) (219 41) (230 17) (291 12) |
| 275 | 1516,9 | 8,7697 | 66 | (55 796) (58 196) (59 365) (60 42) (61 97) (66 24) (72 256) (74 590) (75 999) (76 69) (77 38) (101 425) (102 47) (105 33) (115 68) (116 52) (117 147) (129 65) (131 234) (132 40) (133 749) (134 105) (135 80) (136 38) (143 98) (144 19) (148 441) (149 309) (150 29) (151 34) (171 97) (175 264) (176 40) (177 82) (179 103) (180 16) (189 357) (190 262) (191 274) (192 58) (193 29) (203 34) (204 15) (205 18) (217 146) (218 27) (219 17) (221 78) (231 11) (234 131) (235 58) (246 75) (247 50) (260 11) (263 76) (264 21) (265 106) (266 23) (267 13) (305 25) (306 42) (308 36) (309 15) (319 43) (320 10) (336 27) |
| 276 | 1533,8 | 8,9073 | 31 | (70 40) (71 153) (72 38) (73 999) (74 84) (99 35) (100 136) (103 68) (115 129) (128 64) (131 40) (133 64) (143 26) (146 43) (147 384) (148 58) (149 37) (154 39) (155 79) (191 737) (192 126) (193 60) (194 9) (215 26) (216 17) (221 40) (231 32) (243 74) (244 99) (245 28) (246 9) |
| 277 | 1552,4 | 9,0583 | 80 | (53 45) (54 16) (55 132) (56 82) (57 584) (59 68) (60 14) (66 32) (67 37) (68 52) (69 100) (70 137) (71 509) (72 84) (73 999) (74 76) (75 232) (77 28) (82 25) (83 54) (84 117) (85 396) (86 28) (87 22) (89 56) (92 11) (94 25) (95 14) (96 23) (97 38) (98 39) (99 117) (101 19) (103 169) (104 15) (110 13) (113 65) (114 16) (116 80) (117 102) (118 20) (122 11) (125 21) (126 28) (127 60) (133 38) (134 12) (139 9) (141 18) (142 19) (143 16) (144 26) (147 275) (148 48) (149 32) (154 9) (155 31) (156 20) (158 32) (159 11) (160 105) (161 30) (168 7) (173 38) (174 28) (183 22) (185 7) (186 8) (187 5) (189 33) (201 32) (215 29) (216 34) (228 28) (231 12) (244 190) (245 44) (246 16) (259 17) (341 7) |
| 278 | 1559,9 | 9,1199 | 29 | (78 38) (88 999) (90 44) (120 16) (136 37) (164 44) (193 23) (194 6) (195 12) (208 61) (209 8) (210 7) (211 12) (222 127) (223 28) (224 14) (236 49) (237 260) (238 78) (240 17) (252 35) (253 8) (254 25) (255 10) (268 39) (269 8) (283 122) (284 27) (285 10) |
| 279 | 1571,9 | 9,2175 | 22 | (56 166) (57 999) (70 72) (71 979) (72 36) (84 113) (85 777) (86 40) (99 214) (100 48) (113 112) (114 16) (126 25) (127 79) (141 23) (155 46) (168 16) (170 21) (174 74) (197 14) (240 10) (254 14) |
| 280 | 1581,4 | 9,2945 | 16 | (55 297) (56 134) (57 988) (70 160) (71 999) (72 46) (82 85) (83 89) (84 131) (85 643) (97 33) (99 200) (113 116) (141 34) (155 35) (183 27) |
| 281 | 1592,2 | 9,3825 | 94 | (54 2) (55 16) (58 7) (59 33) (60 2) (61 5) (66 3) (72 20) (73 999) (74 83) (75 71) (76 4) (83 8) (87 5) (88 2) (89 18) (101 12) (102 40) (103 95) (104 10) (105 6) (113 4) (115 8) (116 3) (117 119) (118 11) (119 11) (129 25) (130 37) (131 33) (132 5) (133 66) (134 10) (135 7) (143 16) (144 2) (145 2) (147 412) (148 63) (149 41) (150 4) (151 2) (155 5) (156 6) (157 5) (159 2) (161 2) (163 5) (173 1) (175 4) (177 11) (178 2) (179 1) (189 21) (190 4) (191 8) (192 1) (203 5) (204 13) (205 89) (206 17) (207 11) (208 2) (217 73) (218 14) (219 10) (220 106) (221 37) (222 13) (223 3) (231 1) (245 13) (246 3) (247 1) (277 6) (278 2) (279 1) (291 14) (292 175) (293 50) (294 24) (295 5) (296 1) (305 2) (307 1) (319 14) (320 3) (321 3) (322 1) (379 3) (380 1) (409 4) (410 2) (411 1) |
| 282 | 1617,4 | 9,5877 | 60 | (53 21) (54 22) (55 224) (56 157) (57 999) (58 42) (65 5) (66 2) (67 25) (68 18) (69 106) (70 116) (71 634) (72 32) (77 2) (79 4) (80 1) (81 6) (82 20) (83 60) (84 59) (85 399) (86 25) (91 1) (95 1) (96 9) (97 42) (98 39) (99 98) (100 7) (110 4) (111 18) (112 29) (113 48) (114 4) (124 1) (125 7) (126 21) (127 25) (128 2) (139 1) (140 15) (141 17) (142 1) (153 1) (154 10) (155 12) (156 1) (168 7) (169 10) (170 1) (182 3) (183 5) (184 1) (193 1) (196 1) (197 3) (226 13) (227 2) (235 1) |
| 283 | 1623,5 | 9,6369 | 69 | (53 3) (54 5) (59 30) (72 12) (73 781) (74 58) (75 36) (81 6) (85 4) (87 3) (89 7) (99 4) (101 24) (102 2) (103 31) (104 3) (105 3) (111 9) (112 2) (113 4) (115 5) (117 23) (119 4) (125 2) (127 6) (129 140) (130 12) (131 27) (133 59) (134 7) (135 5) (142 2) (143 37) (144 4) (145 3) (147 168) (148 26) (149 20) (150 2) (175 7) (176 1) (177 2) (182 1) (189 3) (191 4) (201 2) (203 12) (204 3) (205 6) (206 1) (207 2) (215 2) (216 1) (217 999) (218 195) (219 85) (220 11) (221 6) (227 1) (230 6) (231 1) (242 1) (243 6) (289 2) (291 4) (292 1) (317 5) (318 2) (319 2) |
| 284 | 1647,4 | 9,8315 | 10 | (172 43) (255 14) (260 24) (261 9) (262 999) (263 227) (264 91) (350 44) (351 12) (352 6) |
| 285 | 1670,9 | 10,0229 | 36 | (54 40) (55 233) (56 47) (57 139) (61 62) (67 44) (68 31) (70 111) (71 156) (72 45) (73 280) (75 999) (76 86) (77 52) (82 44) (83 86) (85 78) (94 29) (96 25) (97 68) (99 34) (113 105) (117 245) (118 29) (126 73) (129 44) (140 25) (152 15) (154 41) (168 15) (182 68) (196 15) (208 32) (226 340) (227 49) (228 19) |
| 286 | 1675,9 | 10,0639 | 54 | (54 26) (55 218) (56 173) (57 823) (59 56) (68 44) (69 144) (70 84) (71 460) (72 38) (73 833) (74 63) (82 18) (83 75) (84 32) (85 303) (86 39) (97 62) (98 21) (99 120) (100 23) (103 88) (111 31) (112 27) (113 77) (114 15) (125 15) (127 60) (129 68) (131 44) (133 101) (139 12) (141 45) (147 289) (148 48) (151 22) (155 18) (167 53) (168 9) (172 22) (174 40) (191 40) (196 30) (197 40) (201 18) (203 37) (204 35) (217 999) (218 196) (219 89) (220 14) (230 34) (256 43) (332 10) |
| 287 | 1687,4 | 10,1577 | 44 | (50 66) (51 55) (55 150) (59 48) (60 24) (61 36) (68 25) (72 27) (73 999) (74 80) (75 462) (83 67) (86 82) (89 57) (93 22) (96 20) (97 28) (100 38) (103 141) (107 20) (109 20) (110 18) (111 30) (116 28) (117 77) (124 50) (129 91) (130 19) (131 26) (133 23) (147 85) (152 49) (157 18) (185 16) (189 25) (199 42) (201 77) (217 71) (218 20) (226 43) (227 11) (242 19) (244 11) (245 23) |
| 288 | 1732,9 | 10,528 | 35 | (53 21) (56 38) (57 268) (69 52) (71 241) (73 999) (85 152) (99 63) (103 91) (113 59) (116 31) (127 31) (129 101) (131 38) (133 59) (143 28) (147 152) (148 22) (149 21) (155 26) (161 16) (189 77) (190 13) (191 59) (192 12) (204 294) (205 63) (206 32) (217 272) (218 54) (219 25) (319 7) (333 42) (334 14) (335 7) |
| 289 | 1772,2 | 10,8477 | 47 | (55 280) (56 78) (57 190) (59 99) (61 25) (66 22) (67 53) (69 398) (70 72) (71 97) (72 31) (73 999) (74 55) (75 108) (82 98) (83 251) (84 75) (85 62) (87 41) (88 57) (89 41) (97 100) (101 140) (103 200) (104 15) (109 21) (110 17) (111 165) (112 64) (116 326) (117 623) (118 68) (119 25) (125 60) (126 17) (139 12) (153 29) (161 73) (196 9) (224 7) (225 7) (246 28) (247 8) (274 23) (275 6) (285 5) (363 6) |
| 290 | 1784,2 | 10,9453 | 54 | (53 19) (57 96) (59 19) (71 117) (72 20) (73 999) (74 83) (75 29) (77 16) (85 98) (99 32) (106 14) (109 9) (113 34) (127 23) (131 14) (133 50) (134 8) (141 12) (143 31) (147 241) (148 47) (149 26) (150 8) (160 12) (169 31) (178 5) (191 51) (192 7) (203 9) (217 678) (218 165) (219 70) (220 12) (232 24) (233 7) (234 3) (239 3) (257 24) (258 5) (268 7) (294 6) (305 24) (306 7) (317 5) (318 4) (319 3) (321 5) (345 4) (450 24) (451 11) (452 5) (453 2) (454 1) |
| 291 | 1787,4 | 10,9718 | 61 | (51 35) (53 75) (54 51) (55 925) (56 746) (57 999) (58 31) (65 16) (67 158) (68 98) (69 774) (70 695) (71 677) (72 46) (79 40) (81 109) (82 83) (83 389) (84 225) (85 315) (86 13) (94 11) (95 85) (96 69) (97 274) (98 102) (99 75) (101 23) (103 151) (109 36) (110 37) (111 291) (112 94) (113 43) (117 66) (123 15) (124 23) (125 202) (126 124) (127 38) (140 48) (141 23) (147 111) (184 9) (196 8) (204 19) (205 59) (206 8) (214 6) (273 9) (274 6) (275 4) (277 11) (278 4) (292 105) (293 30) (294 15) (307 14) (333 15) (376 2) (421 2) |
| 292 | 1802,9 | 11,0976 | 11 | (52 54) (59 62) (66 99) (73 144) (87 22) (131 73) (132 14) (147 999) (148 158) (149 83) (294 21) |
| 293 | 1828,1 | 11,3027 | 45 | (70 194) (84 74) (125 11) (139 14) (141 176) (155 40) (157 999) (158 112) (159 34) (167 12) (171 44) (183 43) (184 12) (211 40) (240 22) (245 16) (254 5) (256 506) (257 153) (268 10) (269 6) (273 259) (274 56) (275 27) (284 4) (285 9) (305 10) (347 55) (348 33) (349 14) (350 3) (358 13) (359 4) (363 44) (364 12) (365 5) (373 24) (374 10) (375 54) (376 14) (377 6) (450 5) (465 17) (466 6) (467 2) |
| 294 | 1832,6 | 11,3396 | 68 | (51 28) (53 163) (54 44) (55 756) (56 231) (57 833) (58 28) (63 4) (65 76) (66 35) (67 665) (68 999) (69 670) (70 162) (71 463) (72 23) (77 67) (78 11) (79 220) (80 50) (81 548) (82 746) (83 460) (84 61) (85 197) (86 17) (91 30) (92 4) (93 54) (94 98) (95 827) (96 264) (97 323) (98 41) (99 23) (107 21) (108 14) (109 249) (110 102) (111 147) (112 22) (113 14) (121 6) (122 14) (123 420) (124 169) (125 52) (126 31) (127 10) (135 5) (137 68) (138 27) (139 7) (140 9) (151 15) (152 12) (165 7) (168 8) (179 15) (180 3) (193 11) (194 3) (204 5) (208 7) (236 3) (249 6) (263 6) (278 6) |
| 295 | 1853,4 | 11,5089 | 60 | (53 81) (55 167) (60 15) (65 22) (66 19) (67 209) (68 274) (69 158) (70 39) (77 48) (79 210) (80 45) (81 378) (82 368) (83 123) (84 153) (86 119) (87 13) (88 34) (91 23) (93 28) (94 45) (95 297) (96 105) (97 79) (102 30) (108 8) (109 108) (110 52) (111 42) (112 19) (114 19) (123 158) (124 74) (125 15) (128 55) (130 51) (137 27) (138 12) (146 33) (151 9) (156 131) (157 21) (158 13) (168 25) (172 21) (174 999) (175 178) (176 81) (177 8) (200 170) (201 29) (202 11) (216 7) (230 20) (258 33) (259 8) (278 17) (362 26) (363 8) |
| 296 | 1868,8 | 11,6343 | 49 | (53 211) (54 40) (55 522) (56 121) (57 622) (65 81) (66 58) (67 589) (68 698) (69 453) (70 103) (71 304) (72 21) (77 113) (79 566) (80 104) (81 980) (82 999) (83 383) (84 40) (85 95) (91 51) (93 84) (94 107) (95 850) (96 305) (97 242) (107 38) (108 10) (109 251) (110 115) (111 124) (112 15) (121 18) (122 19) (123 441) (124 160) (125 38) (137 81) (138 31) (139 11) (151 21) (152 18) (165 12) (179 22) (193 14) (263 9) (278 58) (279 16) |
| 297 | 1874 | 11,6753 | 57 | (54 50) (55 277) (56 130) (58 77) (68 47) (69 218) (70 126) (71 461) (72 68) (73 552) (74 123) (84 213) (85 373) (86 57) (97 57) (98 114) (99 512) (100 58) (110 32) (111 71) (112 169) (113 190) (122 79) (123 36) (124 37) (126 66) (127 78) (136 51) (137 38) (141 54) (150 23) (154 29) (155 30) (165 63) (169 34) (171 67) (174 74) (176 24) (181 25) (192 167) (193 27) (206 42) (237 27) (238 16) (239 16) (241 29) (248 21) (264 999) (265 223) (266 90) (278 18) (279 192) (280 37) (327 66) (328 23) (329 11) (342 29) |
| 298 | 1887,7 | 11,7879 | 98 | (59 37) (60 5) (72 29) (73 999) (74 87) (87 8) (88 5) (89 57) (90 4) (91 11) (100 10) (101 20) (102 6) (103 347) (104 32) (105 23) (113 17) (114 13) (115 8) (120 2) (133 58) (134 8) (135 8) (141 5) (142 5) (144 3) (147 205) (148 32) (149 32) (150 5) (151 2) (154 7) (155 5) (156 4) (158 3) (163 5) (164 2) (168 5) (169 4) (172 9) (173 12) (175 6) (188 2) (189 29) (190 6) (191 17) (192 2) (197 1) (198 2) (199 2) (201 10) (202 7) (203 5) (204 22) (214 2) (215 6) (216 4) (219 21) (220 10) (221 7) (222 7) (223 2) (228 2) (229 7) (231 5) (232 2) (237 1) (241 7) (243 5) (244 4) (246 2) (256 2) (262 12) (263 7) (264 2) (276 1) (277 25) (278 7) (279 3) (288 1) (291 4) (293 3) (302 1) (305 3) (308 25) (309 12) (315 6) (316 2) (331 10) (333 5) (334 3) (335 4) (336 2) (361 1) (364 12) (365 4) (366 2) (377 1) |
| 299 | 1888,4 | 11,7932 | 106 | (53 93) (54 243) (55 860) (56 307) (57 999) (59 91) (60 15) (65 21) (66 17) (67 167) (68 102) (69 503) (70 438) (71 400) (72 88) (74 219) (77 42) (79 117) (80 43) (81 136) (82 486) (83 478) (84 242) (85 232) (86 31) (87 26) (88 13) (90 11) (91 35) (93 101) (94 50) (95 87) (96 648) (97 810) (98 185) (99 98) (100 28) (101 50) (102 16) (107 46) (108 27) (109 33) (110 603) (111 319) (112 92) (113 57) (115 23) (119 40) (121 28) (122 32) (123 14) (124 371) (125 107) (126 70) (127 27) (130 46) (131 92) (134 23) (135 26) (136 20) (137 7) (138 179) (139 43) (140 41) (141 15) (142 16) (143 32) (144 8) (150 14) (151 7) (152 113) (153 22) (154 23) (155 15) (156 9) (158 8) (159 13) (164 5) (166 89) (167 19) (168 18) (169 14) (180 101) (181 18) (182 16) (188 5) (194 130) (195 24) (196 19) (199 7) (201 29) (202 17) (208 97) (209 18) (215 21) (220 31) (222 23) (223 5) (228 4) (229 22) (236 7) (241 17) (243 17) (302 4) (315 16) (361 3) |
| 300 | 1888,7 | 11,7959 | 76 | (53 95) (54 261) (55 859) (56 307) (57 999) (65 22) (66 17) (67 166) (68 103) (69 502) (70 453) (71 414) (72 60) (77 35) (79 120) (80 45) (81 130) (82 550) (83 508) (84 222) (85 221) (86 27) (87 18) (91 28) (93 100) (94 52) (95 81) (96 754) (97 911) (98 182) (99 82) (107 45) (108 28) (109 33) (110 703) (111 346) (112 96) (113 48) (121 26) (122 34) (123 13) (124 435) (125 115) (126 68) (127 25) (134 13) (135 20) (136 21) (137 7) (138 206) (139 46) (140 41) (141 12) (152 126) (153 21) (154 21) (166 99) (167 18) (168 15) (169 11) (180 114) (181 21) (182 15) (194 147) (195 26) (196 19) (199 5) (208 104) (209 19) (215 15) (220 24) (222 20) (229 15) (236 7) (243 13) (275 4) |
| 301 | 1902,1 | 11,9055 | 189 | (53 3) (54 5) (58 6) (59 34) (60 3) (61 6) (65 1) (66 2) (68 3) (70 5) (72 19) (73 999) (74 75) (75 93) (76 5) (77 5) (82 5) (83 6) (84 8) (86 6) (88 4) (89 46) (90 4) (96 5) (97 4) (98 5) (99 7) (100 12) (101 15) (102 7) (103 110) (104 10) (105 29) (106 3) (107 3) (109 2) (110 3) (111 3) (112 3) (113 6) (114 12) (115 7) (116 8) (117 110) (118 11) (119 11) (120 2) (121 2) (122 1) (123 2) (124 1) (126 2) (127 5) (128 5) (129 85) (130 16) (131 29) (132 6) (133 66) (134 9) (135 6) (136 1) (137 1) (138 2) (139 1) (140 1) (141 3) (142 4) (143 12) (144 2) (145 11) (146 2) (147 331) (148 50) (149 31) (150 4) (151 2) (152 1) (155 3) (156 2) (157 56) (158 11) (159 5) (160 165) (161 27) (162 8) (163 10) (164 1) (165 1) (168 1) (169 3) (170 1) (171 1) (172 3) (173 5) (174 2) (175 5) (176 1) (177 4) (178 1) (179 1) (180 2) (181 1) (183 1) (185 2) (186 3) (187 1) (188 1) (189 34) (190 8) (191 15) (192 3) (193 1) (199 1) (200 1) (201 8) (202 2) (203 4) (204 29) (205 204) (206 39) (207 20) (208 2) (209 1) (210 3) (211 1) (214 1) (215 3) (216 9) (217 95) (218 20) (219 9) (220 1) (221 7) (222 2) (223 1) (228 1) (229 25) (230 7) (231 10) (232 4) (233 5) (234 3) (235 1) (240 1) (242 1) (243 2) (244 3) (245 1) (246 3) (247 1) (256 1) (260 1) (262 4) (263 1) (265 1) (268 1) (269 3) (270 1) (274 6) (275 2) (276 1) (277 8) (278 3) (279 1) (291 12) (292 3) (293 2) (297 5) (298 1) (300 2) (301 1) (302 1) (304 1) (305 7) (306 3) (307 6) (308 2) (321 28) (322 5) (323 1) (330 1) (332 1) (343 2) (344 1) (358 1) (364 4) (374 2) (376 1) |
| 302 | 1913,3 | 11,9965 | 13 | (55 216) (56 141) (57 999) (69 181) (71 905) (83 76) (85 554) (97 106) (99 106) (111 47) (125 22) (182 30) (183 31) |
| 303 | 1927,2 | 12,1094 | 149 | (53 2) (55 24) (56 9) (57 53) (58 11) (59 25) (60 4) (61 7) (63 2) (65 3) (66 2) (67 3) (68 2) (69 13) (70 26) (71 54) (72 22) (73 999) (74 88) (75 103) (76 7) (77 9) (78 4) (81 2) (82 3) (83 9) (84 8) (85 33) (86 12) (87 3) (88 2) (89 12) (90 7) (91 18) (92 1) (95 2) (96 1) (97 6) (98 3) (99 11) (100 126) (101 19) (102 9) (103 25) (104 6) (105 10) (106 1) (108 1) (109 2) (111 4) (112 2) (113 7) (114 1) (115 10) (116 4) (117 10) (118 7) (119 7) (120 2) (121 3) (125 2) (127 4) (128 2) (130 14) (131 16) (132 28) (133 33) (134 7) (135 11) (136 2) (137 1) (141 3) (144 3) (145 4) (146 6) (147 119) (148 22) (149 27) (150 4) (151 5) (157 2) (158 3) (159 3) (160 7) (161 4) (162 3) (163 22) (164 8) (165 11) (166 2) (167 1) (172 2) (174 7) (175 5) (176 6) (177 7) (178 2) (179 75) (180 17) (181 5) (183 1) (188 2) (189 2) (190 6) (191 4) (192 15) (193 5) (194 1) (202 2) (203 7) (204 4) (205 11) (206 4) (207 9) (208 2) (209 2) (210 1) (216 1) (218 797) (219 152) (220 68) (221 10) (223 2) (234 1) (237 1) (238 1) (239 2) (248 2) (264 2) (265 8) (266 3) (267 2) (279 5) (280 75) (281 19) (282 7) (283 1) (291 1) (299 21) (300 6) (301 1) (307 2) (308 1) (319 12) (320 4) (321 2) (331 1) (355 8) (356 3) |
| 304 | 1947,6 | 12,2753 | 131 | (53 17) (58 12) (59 27) (60 3) (61 7) (62 5) (63 11) (67 12) (72 18) (73 999) (74 89) (75 79) (76 47) (77 22) (78 316) (79 29) (80 6) (83 54) (87 5) (88 9) (89 31) (90 8) (91 9) (92 4) (93 12) (94 7) (102 12) (103 103) (104 22) (105 7) (106 134) (107 13) (108 11) (115 21) (116 20) (118 9) (119 10) (120 9) (121 5) (122 13) (123 32) (128 17) (129 16) (130 91) (131 63) (132 46) (133 26) (134 8) (135 7) (136 9) (138 3) (142 7) (143 15) (144 20) (145 24) (146 31) (147 77) (148 16) (150 7) (153 7) (156 10) (157 14) (158 14) (159 16) (160 48) (161 68) (162 27) (163 6) (164 2) (170 7) (172 16) (173 8) (174 33) (175 6) (176 7) (178 3) (184 2) (186 12) (187 6) (188 77) (189 34) (190 32) (191 5) (200 3) (201 3) (202 35) (203 23) (204 97) (205 58) (206 13) (207 3) (215 2) (216 20) (217 10) (218 36) (219 14) (220 5) (230 8) (231 6) (232 184) (233 59) (234 17) (246 35) (247 18) (248 53) (249 18) (250 7) (251 2) (260 9) (261 6) (262 581) (263 147) (264 55) (265 8) (274 2) (275 1) (276 20) (277 9) (278 4) (290 2) (292 3) (306 200) (307 52) (308 21) (309 4) (319 3) (321 995) (322 267) (323 100) (324 19) (325 3) |
| 305 | 1954,8 | 12,3341 | 36 | (59 110) (72 31) (73 999) (74 126) (75 107) (87 28) (101 49) (103 442) (104 35) (112 43) (117 116) (129 361) (130 42) (131 76) (132 36) (133 155) (147 535) (148 94) (149 68) (160 64) (175 25) (177 78) (188 49) (189 27) (203 33) (204 44) (205 34) (207 19) (221 20) (276 209) (277 60) (278 33) (313 12) (350 24) (351 7) (496 3) |
| 306 | 1960 | 12,3766 | 72 | (53 18) (54 25) (55 262) (56 151) (57 999) (58 43) (65 4) (66 2) (67 31) (68 22) (69 136) (70 111) (71 650) (72 33) (77 2) (79 3) (80 1) (81 10) (82 27) (83 85) (84 58) (85 436) (86 27) (91 1) (95 3) (96 14) (97 68) (98 37) (99 122) (100 9) (109 1) (110 7) (111 33) (112 27) (113 71) (114 6) (124 3) (125 14) (126 20) (127 45) (128 4) (138 2) (139 5) (140 16) (141 30) (142 3) (152 1) (153 2) (154 12) (155 19) (156 2) (167 1) (168 8) (169 13) (170 1) (182 7) (183 9) (184 1) (196 5) (197 7) (198 1) (210 4) (211 5) (212 1) (224 3) (225 5) (226 1) (238 1) (239 3) (253 1) (282 7) (283 1) |
| 307 | 1972,7 | 12,4802 | 35 | (191 100) (204 51) (205 23) (206 7) (217 999) (218 206) (219 94) (220 12) (231 94) (233 25) (244 12) (245 30) (246 18) (247 26) (257 28) (258 7) (259 10) (260 33) (269 8) (271 9) (285 36) (286 12) (305 103) (306 36) (317 8) (318 130) (319 42) (320 22) (331 32) (332 20) (359 34) (360 10) (361 5) (449 6) (450 3) |
| 308 | 1982,7 | 12,5611 | 238 | (51 4) (52 3) (53 40) (54 89) (55 418) (56 78) (57 49) (58 17) (59 25) (60 10) (61 60) (62 4) (63 3) (65 17) (66 12) (67 191) (68 69) (69 172) (70 36) (71 19) (72 40) (73 999) (74 102) (75 825) (76 58) (77 53) (78 7) (79 65) (80 30) (81 162) (82 81) (83 104) (84 132) (85 32) (86 10) (87 6) (88 5) (89 21) (90 3) (91 31) (92 9) (93 41) (94 23) (95 117) (96 157) (97 95) (98 122) (99 28) (100 4) (101 11) (102 2) (103 9) (104 2) (105 25) (106 6) (107 24) (108 15) (109 63) (110 63) (111 41) (112 23) (113 8) (114 2) (115 8) (116 53) (117 627) (118 62) (119 49) (120 8) (121 23) (122 9) (123 50) (124 28) (125 12) (126 5) (127 7) (128 5) (129 565) (130 68) (131 95) (132 66) (133 54) (134 19) (135 16) (136 6) (137 34) (138 25) (139 10) (140 2) (141 11) (142 8) (143 37) (144 6) (145 150) (146 21) (147 79) (148 18) (149 14) (150 3) (151 20) (152 49) (153 12) (154 3) (155 28) (156 6) (157 22) (158 6) (159 14) (160 2) (161 6) (162 2) (163 4) (164 1) (165 13) (166 9) (167 4) (168 3) (169 13) (170 6) (171 23) (172 9) (173 13) (175 5) (176 2) (177 2) (178 1) (179 8) (180 4) (181 4) (182 1) (183 19) (184 4) (185 42) (186 10) (187 10) (188 4) (189 8) (190 5) (191 15) (192 12) (193 15) (194 49) (195 10) (196 1) (197 4) (198 1) (199 44) (200 8) (201 12) (202 2) (203 5) (204 11) (205 5) (206 1) (207 8) (208 7) (209 2) (211 4) (212 1) (213 12) (214 3) (215 5) (216 1) (217 170) (218 38) (219 16) (220 2) (221 5) (222 1) (223 1) (225 3) (226 1) (227 11) (228 2) (229 7) (230 6) (231 5) (232 2) (233 2) (235 1) (236 30) (237 6) (238 1) (239 3) (240 1) (241 5) (242 2) (243 9) (244 6) (245 2) (246 7) (247 2) (248 1) (253 2) (255 5) (256 1) (257 2) (258 3) (259 1) (265 1) (267 5) (268 1) (269 3) (270 1) (271 4) (272 1) (281 1) (283 5) (284 1) (285 1) (291 2) (292 2) (293 2) (294 1) (295 1) (297 1) (305 15) (306 8) (307 3) (308 1) (311 254) (312 61) (313 17) (314 2) (317 3) (318 12) (319 4) (320 2) (325 1) (326 14) (327 3) (328 1) (331 1) (332 3) (333 1) (345 3) (346 1) (367 1) |
| 309 | 1996,8 | 12,6761 | 10 | (50 8) (80 53) (94 43) (115 166) (116 999) (128 89) (200 29) (242 26) (284 38) (330 7) |
| 310 | 1997,6 | 12,6828 | 125 | (72 91) (74 188) (80 4) (94 5) (104 1) (118 51) (119 24) (120 2) (121 6) (122 1) (124 2) (125 4) (127 3) (129 392) (130 56) (132 295) (133 74) (134 20) (135 8) (136 1) (138 2) (139 3) (141 4) (143 44) (145 257) (146 35) (147 19) (148 3) (149 3) (150 1) (151 2) (152 1) (153 5) (154 4) (155 7) (156 1) (157 17) (158 3) (159 26) (160 6) (161 2) (163 3) (165 1) (166 1) (167 5) (168 3) (169 2) (170 1) (171 40) (172 7) (173 12) (174 8) (175 2) (177 2) (181 6) (182 6) (183 2) (185 65) (186 13) (187 40) (188 11) (189 3) (190 1) (191 2) (194 1) (195 10) (196 4) (197 1) (199 21) (200 2) (201 96) (202 18) (203 6) (204 1) (205 1) (209 4) (210 1) (211 1) (213 21) (214 4) (215 18) (216 4) (217 3) (219 2) (220 1) (227 24) (228 5) (229 26) (230 7) (231 2) (237 1) (238 2) (239 2) (241 15) (243 29) (244 7) (245 2) (255 9) (256 2) (257 22) (258 6) (259 1) (269 61) (270 15) (271 17) (272 4) (273 1) (283 11) (285 50) (286 13) (287 3) (297 2) (299 13) (300 3) (301 1) (311 3) (313 999) (314 259) (315 68) (316 10) (317 1) (328 58) (329 17) (330 4) (353 1) |
| 311 | 1998,8 | 12,6925 | 153 | (51 1) (52 1) (53 19) (54 22) (55 277) (56 57) (57 137) (58 26) (59 31) (60 11) (61 62) (62 4) (63 3) (65 5) (66 3) (67 58) (68 16) (69 118) (70 27) (71 34) (73 999) (75 803) (76 59) (77 41) (78 2) (79 20) (81 48) (82 10) (83 60) (84 25) (85 27) (86 12) (87 8) (88 6) (89 21) (90 3) (91 7) (92 1) (93 17) (95 46) (96 8) (97 45) (98 35) (99 23) (101 11) (102 2) (103 3) (105 17) (106 2) (107 9) (109 16) (110 3) (111 23) (112 11) (113 4) (114 1) (117 869) (118 84) (119 37) (120 2) (121 8) (122 1) (123 5) (124 1) (125 5) (126 4) (127 4) (129 411) (130 52) (131 134) (132 207) (133 48) (134 10) (135 6) (136 1) (137 2) (138 1) (139 3) (140 3) (141 2) (143 33) (145 151) (146 18) (147 7) (149 2) (151 1) (153 3) (154 4) (155 3) (157 9) (159 18) (160 3) (161 1) (163 1) (167 2) (168 2) (169 1) (171 18) (172 3) (173 7) (174 3) (175 1) (177 1) (181 2) (182 2) (183 1) (185 26) (186 4) (187 16) (188 5) (189 1) (195 5) (196 1) (199 6) (201 35) (202 6) (203 2) (209 1) (213 6) (214 1) (215 5) (216 1) (227 7) (228 1) (229 9) (230 2) (238 1) (239 1) (241 4) (243 10) (244 2) (255 2) (257 7) (258 2) (269 19) (270 4) (271 5) (272 1) (283 3) (285 16) (286 3) (287 1) (297 1) (299 4) (300 1) (311 1) (313 299) (314 69) (315 17) (316 2) (328 17) (329 4) (330 1) |
| 312 | 2037,6 | 13,0084 | 24 | (61 73) (73 745) (75 999) (76 64) (88 26) (89 54) (103 244) (104 26) (116 37) (117 140) (129 141) (147 76) (157 20) (204 47) (269 80) (270 14) (311 52) (343 89) (344 28) (345 12) (359 125) (360 34) (361 12) (389 12) |
| 313 | 2043,5 | 13,0564 | 123 | (50 5) (51 9) (53 37) (54 38) (59 38) (60 13) (61 72) (62 5) (63 5) (65 28) (66 26) (67 230) (68 38) (72 56) (75 999) (76 69) (77 107) (78 35) (79 210) (80 115) (81 156) (82 27) (84 34) (86 12) (88 6) (90 3) (91 114) (92 28) (93 108) (94 56) (95 84) (96 22) (103 13) (104 6) (105 58) (106 25) (107 56) (108 23) (109 30) (110 11) (115 13) (119 62) (120 39) (121 48) (123 20) (124 7) (125 9) (128 5) (133 86) (134 23) (135 34) (136 25) (137 9) (138 3) (142 6) (143 36) (144 6) (147 28) (148 6) (149 26) (150 13) (151 4) (153 3) (154 4) (155 9) (156 3) (157 15) (158 2) (159 22) (160 3) (161 11) (162 10) (163 13) (164 6) (165 3) (167 3) (169 3) (170 2) (171 23) (172 4) (173 9) (174 5) (175 9) (176 3) (177 4) (181 4) (182 2) (183 4) (185 27) (186 4) (189 5) (190 3) (191 3) (195 2) (199 6) (203 4) (204 7) (205 2) (211 2) (212 1) (213 4) (217 6) (218 2) (219 3) (223 2) (225 1) (227 7) (229 6) (230 2) (231 8) (232 1) (245 1) (246 3) (247 2) (248 4) (249 3) (252 3) (305 2) (319 1) (321 4) (322 1) (323 2) (336 2) |
| 314 | 2055,3 | 13,1522 | 251 | (51 4) (52 2) (53 41) (54 103) (55 352) (56 62) (57 51) (58 19) (59 25) (60 9) (61 50) (62 4) (63 3) (65 25) (66 19) (67 274) (68 90) (69 137) (70 28) (71 17) (72 37) (73 999) (74 99) (75 718) (76 50) (77 65) (78 14) (79 121) (80 58) (81 222) (82 98) (83 90) (84 85) (85 27) (86 9) (87 5) (88 5) (89 19) (90 2) (91 45) (92 11) (93 64) (94 44) (95 140) (96 139) (97 79) (98 77) (99 23) (100 3) (101 11) (102 3) (103 19) (104 3) (105 26) (106 9) (107 38) (108 29) (109 73) (110 59) (111 35) (112 16) (113 7) (114 1) (115 7) (116 48) (117 445) (118 45) (119 41) (120 10) (121 44) (122 18) (123 47) (124 27) (125 12) (126 5) (127 6) (128 4) (129 423) (130 52) (131 84) (132 48) (133 59) (134 17) (135 40) (136 22) (137 29) (138 20) (139 7) (140 2) (141 6) (142 5) (143 35) (144 6) (145 108) (146 15) (147 146) (148 26) (149 33) (150 33) (151 20) (152 20) (153 7) (154 2) (155 20) (156 6) (157 17) (158 5) (159 15) (160 3) (161 8) (162 2) (163 11) (164 21) (165 12) (166 24) (167 7) (168 3) (169 13) (170 5) (171 19) (172 7) (173 14) (174 3) (175 5) (176 1) (177 8) (178 3) (179 6) (180 3) (181 4) (182 2) (183 15) (184 12) (185 30) (186 7) (187 11) (188 4) (189 8) (190 4) (191 66) (192 14) (193 9) (194 3) (195 3) (197 4) (198 1) (199 31) (200 6) (201 10) (202 2) (203 3) (204 28) (205 8) (206 19) (207 12) (208 26) (209 6) (210 1) (211 3) (212 1) (213 7) (214 1) (215 5) (216 1) (217 110) (218 22) (219 11) (220 4) (221 16) (222 6) (223 2) (225 3) (226 1) (227 9) (228 2) (229 4) (230 4) (231 3) (232 2) (233 1) (235 1) (239 2) (240 1) (241 6) (242 1) (243 7) (244 2) (245 1) (247 1) (248 23) (249 5) (250 19) (251 4) (253 2) (255 3) (256 1) (257 3) (258 3) (265 19) (266 5) (267 4) (268 1) (269 1) (271 1) (272 1) (279 1) (281 3) (282 1) (283 1) (285 1) (291 9) (292 2) (293 3) (294 1) (295 1) (297 3) (298 1) (305 83) (306 24) (307 13) (308 4) (309 2) (311 1) (317 1) (318 45) (319 19) (320 8) (321 2) (323 48) (324 11) (325 132) (326 34) (327 9) (328 1) (331 1) (338 2) (339 1) (340 7) (341 2) (342 1) (343 2) (367 4) (368 1) (369 1) (393 3) (394 1) (419 1) (432 7) (433 5) (434 2) (435 1) |
| 315 | 2109,3 | 13,5918 | 104 | (50 1) (51 3) (53 19) (54 27) (55 282) (56 151) (57 999) (58 43) (59 1) (65 6) (66 5) (67 49) (68 26) (69 152) (70 107) (71 641) (72 36) (81 19) (82 29) (83 102) (84 59) (85 440) (86 27) (87 1) (91 16) (92 4) (96 17) (97 82) (98 38) (99 130) (100 9) (104 1) (105 7) (106 6) (110 10) (111 42) (112 29) (113 78) (114 7) (115 1) (119 6) (122 4) (123 2) (124 5) (125 19) (126 21) (127 52) (128 5) (131 4) (134 2) (136 1) (138 3) (139 7) (140 16) (141 36) (142 4) (146 1) (148 1) (149 2) (151 1) (152 2) (153 4) (154 12) (155 27) (156 3) (157 1) (162 1) (166 1) (167 2) (168 10) (169 19) (170 2) (171 1) (173 1) (175 2) (176 1) (180 1) (181 1) (182 7) (183 13) (184 2) (187 1) (189 1) (195 1) (196 6) (197 10) (198 1) (210 5) (211 7) (212 1) (224 3) (225 6) (226 1) (238 3) (239 5) (240 1) (252 2) (253 4) (254 1) (266 1) (267 2) (281 1) (310 6) (311 1) |
| 316 | 2109,5 | 13,5935 | 129 | (51 3) (53 21) (54 29) (55 298) (56 155) (57 999) (58 43) (59 2) (60 1) (65 8) (66 7) (67 63) (68 28) (69 162) (70 109) (71 644) (72 37) (76 4) (77 15) (78 7) (79 40) (80 21) (81 29) (82 31) (83 103) (84 59) (85 435) (86 27) (87 1) (91 24) (92 6) (93 19) (94 8) (95 15) (96 18) (97 82) (98 38) (99 128) (100 9) (101 1) (103 1) (104 1) (105 11) (106 7) (107 9) (108 4) (109 5) (110 10) (111 41) (112 28) (113 76) (114 6) (115 2) (116 5) (119 9) (120 8) (121 7) (122 5) (123 2) (124 5) (125 18) (126 20) (127 49) (128 5) (129 14) (130 3) (131 6) (133 10) (134 3) (135 4) (136 2) (137 2) (138 3) (139 7) (140 15) (141 34) (142 4) (145 7) (146 1) (147 4) (148 1) (149 3) (150 3) (151 1) (152 2) (154 11) (155 25) (156 3) (157 2) (161 3) (162 2) (163 2) (164 1) (166 1) (167 2) (168 9) (169 17) (170 2) (171 2) (173 1) (175 3) (176 1) (177 1) (181 1) (182 7) (183 12) (184 1) (185 2) (187 1) (189 1) (196 5) (197 9) (201 1) (203 1) (210 4) (211 6) (212 1) (224 3) (225 5) (226 1) (238 3) (239 4) (252 2) (253 4) (254 1) (266 1) (369 9) (370 3) (371 1) |
| 317 | 2110,3 | 13,6001 | 91 | (59 24) (60 10) (61 54) (62 3) (66 62) (73 999) (74 104) (75 799) (76 54) (77 161) (78 76) (79 420) (80 236) (81 282) (88 5) (89 19) (91 241) (92 58) (93 197) (94 102) (95 167) (101 12) (103 18) (104 8) (105 102) (107 95) (108 40) (109 48) (115 21) (116 62) (117 712) (118 77) (119 91) (120 87) (121 85) (123 24) (129 168) (130 28) (131 58) (132 163) (133 120) (134 35) (135 47) (136 25) (137 19) (143 293) (144 40) (145 91) (146 12) (147 41) (149 34) (150 41) (151 8) (157 23) (159 385) (160 52) (161 38) (162 21) (163 22) (164 11) (171 14) (173 13) (176 6) (177 12) (178 5) (185 18) (187 8) (189 7) (190 3) (191 9) (199 5) (201 20) (203 7) (204 3) (213 3) (218 7) (229 25) (230 3) (243 5) (249 5) (260 3) (263 4) (271 8) (291 5) (335 5) (350 2) (369 119) (370 33) (371 8) (384 4) (422 3) |
| 318 | 2122,6 | 13,7004 | 103 | (50 3) (51 13) (52 10) (53 89) (54 258) (59 25) (60 11) (61 59) (62 5) (63 6) (64 2) (65 101) (66 86) (67 999) (68 292) (76 65) (77 227) (78 80) (79 649) (80 341) (81 781) (82 301) (88 6) (91 207) (92 47) (93 280) (94 235) (95 462) (102 3) (104 15) (105 73) (106 37) (107 180) (108 161) (109 206) (110 94) (120 35) (121 240) (122 125) (123 73) (124 50) (135 246) (136 182) (139 15) (149 155) (150 262) (151 33) (159 31) (160 11) (161 10) (163 82) (164 115) (173 50) (174 11) (177 42) (178 160) (179 27) (182 6) (187 34) (192 10) (195 5) (196 2) (198 3) (202 3) (206 9) (212 4) (215 15) (216 4) (220 72) (225 6) (226 3) (229 5) (233 6) (234 29) (238 1) (239 8) (240 3) (243 9) (244 5) (245 3) (251 1) (253 4) (254 3) (262 226) (263 45) (268 2) (294 5) (309 3) (310 1) (315 6) (316 2) (337 471) (338 130) (350 3) (351 1) (352 24) (353 5) (409 11) (410 4) (411 2) (424 12) (425 5) (426 3) |
| 319 | 2126,1 | 13,7287 | 204 | (50 1) (51 3) (52 2) (53 45) (54 94) (55 526) (56 123) (57 156) (58 24) (59 27) (60 12) (61 66) (62 5) (63 3) (65 15) (66 12) (67 197) (68 67) (69 235) (70 67) (71 28) (72 60) (73 999) (74 116) (75 913) (76 63) (77 46) (81 171) (82 87) (83 150) (84 162) (85 42) (86 15) (87 8) (88 7) (89 25) (90 3) (91 24) (92 8) (93 42) (94 17) (95 134) (96 209) (97 140) (98 160) (99 36) (100 5) (101 11) (102 2) (103 4) (105 29) (106 5) (107 26) (108 12) (109 85) (110 90) (111 68) (112 35) (113 9) (114 2) (116 59) (117 768) (118 73) (119 62) (120 10) (121 31) (123 69) (124 43) (125 27) (126 9) (127 6) (129 713) (130 86) (131 130) (132 84) (133 57) (134 31) (135 18) (137 47) (138 38) (139 14) (140 5) (141 8) (142 7) (143 55) (145 219) (146 28) (148 19) (151 32) (152 43) (153 11) (154 3) (155 35) (156 10) (157 27) (158 8) (159 20) (160 2) (161 11) (162 4) (165 19) (166 31) (167 13) (168 7) (169 29) (171 38) (172 14) (173 15) (174 3) (175 7) (176 2) (179 9) (180 52) (181 14) (183 37) (184 7) (185 77) (186 18) (187 14) (188 7) (189 5) (190 2) (193 10) (194 8) (195 3) (196 1) (197 7) (198 2) (199 85) (200 15) (201 19) (202 3) (203 3) (207 10) (208 6) (209 4) (210 1) (211 6) (212 1) (213 16) (214 3) (215 4) (216 1) (220 8) (221 18) (222 60) (223 12) (224 1) (225 3) (227 18) (228 4) (229 4) (230 1) (235 10) (236 10) (237 2) (239 3) (241 17) (242 4) (243 4) (244 1) (245 2) (246 5) (247 1) (249 2) (253 2) (255 10) (256 2) (257 9) (258 4) (264 53) (265 11) (266 1) (267 2) (269 4) (270 1) (271 5) (272 7) (273 1) (281 2) (283 3) (285 1) (286 3) (287 1) (295 6) (296 2) (297 3) (298 1) (299 2) (300 1) (309 1) (311 8) (312 2) (313 1) (323 2) (325 2) (339 321) (340 81) (341 21) (342 3) (353 1) (354 18) (355 5) (356 1) |
| 320 | 2129,6 | 13,7571 | 214 | (51 7) (52 4) (59 46) (60 17) (62 7) (63 7) (64 1) (65 35) (66 28) (71 42) (74 172) (76 104) (77 105) (78 17) (79 144) (80 57) (85 59) (86 21) (87 13) (88 10) (89 40) (90 5) (91 55) (92 16) (93 103) (94 54) (95 277) (97 194) (99 55) (100 8) (101 20) (102 4) (103 7) (104 1) (105 49) (106 12) (107 61) (108 42) (109 146) (110 126) (111 90) (112 44) (113 15) (114 3) (116 106) (118 97) (119 81) (120 18) (121 68) (122 26) (123 93) (124 60) (125 38) (126 13) (127 11) (129 999) (130 129) (131 205) (132 142) (133 97) (134 41) (135 50) (136 16) (137 61) (138 48) (139 22) (140 6) (141 11) (143 72) (145 304) (146 42) (147 44) (148 25) (149 28) (150 11) (151 44) (152 53) (153 16) (155 35) (156 11) (157 35) (158 11) (159 32) (160 6) (161 19) (162 8) (163 13) (164 9) (165 26) (166 38) (167 16) (168 8) (169 31) (170 13) (171 64) (172 20) (173 27) (174 6) (175 15) (176 5) (177 7) (178 4) (179 16) (180 63) (181 18) (183 42) (184 9) (185 106) (186 25) (187 25) (188 12) (189 9) (190 3) (191 5) (192 3) (193 14) (194 11) (195 6) (196 2) (197 8) (198 3) (199 106) (200 20) (201 29) (202 5) (203 6) (204 2) (205 1) (206 2) (207 15) (208 9) (209 5) (210 2) (211 8) (212 2) (213 24) (214 5) (215 8) (216 2) (220 15) (221 24) (222 73) (223 16) (225 5) (226 1) (227 22) (228 5) (229 7) (230 2) (235 15) (236 14) (237 3) (239 5) (240 1) (241 23) (242 5) (243 8) (244 2) (245 3) (246 12) (247 3) (248 1) (249 4) (250 1) (253 4) (254 1) (255 20) (256 5) (257 13) (258 6) (259 1) (263 2) (264 73) (265 16) (266 2) (267 5) (268 1) (269 12) (270 3) (271 8) (272 9) (273 2) (274 1) (281 5) (282 1) (283 7) (284 1) (285 2) (286 4) (287 1) (295 13) (296 3) (297 5) (298 1) (299 4) (300 1) (309 4) (310 2) (311 15) (312 4) (313 3) (321 2) (323 4) (324 1) (325 3) (339 535) (340 144) (341 39) (342 6) (343 1) (353 1) (354 30) (355 9) (356 2) |
| 321 | 2134,3 | 13,7948 | 80 | (52 26) (53 68) (54 278) (55 341) (58 42) (59 22) (60 23) (61 53) (65 61) (67 999) (68 267) (69 174) (72 54) (73 623) (74 54) (75 791) (76 39) (77 72) (80 122) (81 710) (82 341) (83 102) (94 88) (95 322) (96 191) (97 89) (107 39) (109 145) (110 93) (111 57) (116 41) (121 90) (122 45) (123 74) (124 39) (125 42) (128 14) (131 36) (135 88) (136 73) (137 19) (138 12) (139 22) (149 57) (150 93) (151 15) (153 9) (156 11) (163 29) (164 35) (165 14) (168 30) (169 16) (173 19) (178 48) (182 18) (183 12) (184 7) (187 18) (191 17) (192 11) (196 12) (201 17) (215 9) (218 8) (220 30) (229 7) (234 10) (241 9) (243 7) (262 75) (263 19) (267 5) (269 5) (281 4) (295 7) (308 7) (309 7) (337 168) (338 45) |
| 322 | 2143,5 | 13,8705 | 21 | (100 20) (110 42) (115 42) (116 999) (128 193) (142 14) (144 248) (158 14) (170 22) (180 6) (184 12) (194 3) (198 8) (212 8) (226 8) (240 5) (254 2) (282 3) (310 5) (312 232) (326 8) |
| 323 | 2153,3 | 13,9502 | 39 | (50 6) (54 11) (55 247) (56 196) (57 999) (58 24) (68 23) (69 149) (70 71) (71 600) (82 22) (83 98) (84 37) (85 402) (86 21) (96 10) (97 55) (98 15) (99 118) (100 5) (111 27) (112 15) (113 53) (125 13) (126 8) (127 33) (140 6) (141 29) (155 21) (183 17) (184 3) (197 16) (211 13) (225 10) (252 2) (253 2) (280 10) (281 28) (282 7) |
| 324 | 2778,6 | 19,0395 | 32 | (54 31) (55 307) (56 162) (57 999) (58 38) (67 47) (68 35) (69 222) (70 119) (71 752) (72 41) (81 24) (82 48) (83 165) (84 67) (85 490) (86 31) (96 31) (97 151) (98 37) (99 123) (111 75) (112 32) (113 70) (125 32) (126 37) (127 52) (141 31) (155 29) (169 16) (183 11) (197 14) |
| 325 | 953,9 | 4,1867 | 39 | (55 34) (56 138) (57 37) (58 258) (59 75) (60 79) (61 71) (62 6) (64 11) (69 933) (70 23) (74 153) (76 62) (78 385) (90 39) (96 34) (97 73) (98 26) (100 63) (106 85) (107 12) (108 27) (110 72) (112 21) (114 34) (126 999) (127 440) (128 317) (129 38) (130 31) (139 182) (140 320) (141 13) (146 97) (155 204) (156 9) (182 4) (183 11) (252 9) |
| 326 | 2309,1 | 14,1998 | 90 | (53 93) (54 237) (55 999) (56 293) (57 873) (58 70) (61 34) (65 84) (66 69) (67 644) (68 177) (69 588) (70 274) (71 305) (72 31) (73 479) (74 50) (75 535) (76 34) (77 182) (78 84) (79 551) (80 229) (81 375) (82 225) (83 324) (84 170) (85 233) (86 29) (91 278) (92 59) (93 237) (94 123) (95 256) (96 185) (97 252) (98 201) (99 85) (105 122) (106 67) (107 108) (108 107) (109 104) (110 90) (111 77) (112 43) (113 40) (116 43) (117 173) (119 66) (120 45) (121 78) (122 222) (123 84) (124 67) (125 40) (129 171) (131 70) (133 41) (135 51) (136 181) (137 55) (138 34) (145 47) (149 28) (150 83) (152 38) (157 20) (161 38) (163 20) (164 37) (166 23) (173 19) (178 55) (183 19) (192 23) (197 24) (206 19) (220 34) (234 29) (248 30) (249 14) (262 30) (263 11) (266 10) (291 22) (294 17) (329 12) (333 14) (337 23) |
| 327 | 2449,5 | 14,959 | 60 | (87 3) (90 7) (100 25) (102 4) (110 8) (114 4) (116 386) (126 3) (128 111) (131 999) (132 111) (138 2) (140 2) (142 17) (144 117) (154 3) (156 9) (158 19) (165 2) (166 2) (170 12) (172 10) (180 2) (184 8) (186 15) (194 1) (198 6) (200 15) (203 9) (209 1) (212 7) (214 5) (216 3) (222 2) (226 5) (228 3) (236 1) (240 6) (242 5) (254 5) (256 4) (264 1) (268 2) (270 4) (271 1) (284 3) (296 2) (298 2) (310 2) (312 9) (313 2) (326 4) (327 1) (340 154) (341 42) (342 10) (343 2) (354 4) (355 14) (356 4) |
| 328 | 2571,3 | 15,5845 | 19 | (56 47) (57 368) (70 182) (71 264) (83 68) (85 54) (99 17) (112 39) (113 68) (114 6) (149 999) (150 94) (151 9) (167 270) (168 18) (225 12) (279 45) (280 8) (313 4) |
| 329 | 2928,7 | 17,2653 | 225 | (51 8) (52 6) (53 36) (54 25) (55 156) (56 10) (58 11) (59 50) (60 6) (61 13) (62 1) (63 4) (65 46) (66 56) (67 303) (68 30) (69 55) (70 12) (72 19) (73 999) (74 85) (75 165) (76 10) (77 135) (78 79) (79 419) (80 124) (81 103) (82 23) (83 20) (84 18) (86 2) (87 6) (88 5) (89 23) (90 2) (91 314) (92 76) (93 184) (94 64) (95 93) (96 18) (97 22) (98 4) (101 76) (102 10) (103 285) (104 56) (105 175) (106 74) (107 65) (108 96) (109 32) (110 6) (113 8) (114 1) (115 36) (116 39) (117 180) (118 39) (119 123) (120 45) (121 43) (122 18) (123 12) (124 3) (127 5) (128 9) (129 330) (130 67) (131 179) (132 46) (133 137) (134 36) (135 39) (136 10) (137 5) (138 1) (141 10) (142 6) (143 26) (144 14) (145 76) (146 32) (147 334) (148 74) (149 49) (150 7) (151 5) (152 1) (153 3) (155 10) (156 4) (157 28) (158 9) (159 34) (160 15) (161 32) (162 23) (163 13) (164 4) (165 3) (166 1) (167 4) (168 2) (169 13) (170 5) (171 28) (172 8) (173 28) (174 12) (175 45) (176 16) (177 6) (178 1) (179 2) (180 1) (181 5) (183 10) (184 4) (185 17) (186 7) (187 22) (188 21) (189 18) (190 4) (191 5) (192 1) (193 2) (195 4) (196 2) (197 11) (198 3) (199 16) (200 13) (201 48) (202 13) (203 67) (204 18) (205 40) (206 8) (207 5) (208 1) (209 4) (210 3) (211 5) (212 2) (213 10) (214 5) (215 15) (216 6) (217 16) (218 9) (219 18) (220 4) (221 4) (222 1) (223 4) (224 1) (225 4) (226 1) (227 8) (228 7) (229 4) (230 3) (231 1) (233 1) (235 1) (237 10) (238 4) (239 4) (240 3) (241 7) (242 4) (243 4) (244 1) (245 1) (247 1) (251 1) (252 1) (253 1) (255 5) (256 5) (257 3) (258 1) (259 1) (263 1) (265 2) (266 3) (267 3) (268 1) (269 2) (275 1) (279 1) (280 1) (281 2) (282 1) (283 3) (284 6) (285 3) (291 1) (293 2) (294 6) (295 2) (301 1) (303 1) (307 2) (308 1) (309 2) (321 1) (322 1) (327 4) (328 1) (335 1) (347 1) (374 1) (375 1) (384 1) (387 1) (417 14) (418 4) (419 1) (424 1) (430 1) (452 1) |
| 330 | 2976,3 | 17,4782 | 55 | (53 64) (54 133) (55 999) (56 384) (57 333) (63 11) (65 27) (66 22) (67 428) (68 511) (69 662) (70 145) (71 99) (77 27) (79 134) (80 79) (81 435) (82 424) (83 368) (84 48) (85 52) (91 54) (93 72) (95 331) (96 337) (97 278) (98 33) (107 34) (109 176) (110 105) (111 88) (119 24) (120 15) (121 43) (123 87) (124 45) (125 22) (136 13) (137 34) (138 28) (145 24) (151 12) (152 13) (159 16) (165 12) (166 13) (172 13) (174 11) (213 11) (214 6) (222 7) (250 17) (353 7) (368 19) (369 5) |
| 331 | 2984 | 17,5125 | 43 | (53 49) (54 116) (55 999) (56 286) (57 179) (63 9) (65 17) (66 19) (67 309) (68 413) (69 546) (70 120) (71 68) (79 66) (80 37) (81 347) (82 403) (83 326) (84 44) (85 36) (93 24) (94 28) (95 253) (96 288) (97 269) (98 24) (109 135) (110 108) (111 89) (123 69) (124 55) (125 25) (137 43) (138 31) (151 16) (152 17) (166 12) (172 10) (228 8) (235 6) (250 18) (277 6) (424 10) |
| 332 | 2064,3 | 12,7723 | 33 | (53 17) (54 25) (55 295) (56 203) (57 999) (67 38) (68 25) (69 152) (70 82) (71 596) (81 14) (82 26) (83 96) (85 402) (86 16) (96 16) (97 81) (98 25) (112 15) (113 45) (126 11) (127 42) (141 34) (155 34) (174 28) (183 22) (197 13) (211 9) (225 4) (252 17) (253 35) (254 7) (281 5) |
| 333 | 1087,3 | 5,2728 | 43 | (53 12) (56 59) (57 201) (58 39) (59 67) (60 22) (61 39) (62 4) (70 48) (71 183) (73 999) (74 100) (75 470) (76 25) (77 20) (84 36) (85 95) (86 17) (90 4) (99 11) (105 97) (106 9) (107 48) (108 4) (109 4) (113 10) (115 5) (120 10) (127 15) (129 36) (130 36) (131 20) (133 176) (134 17) (135 15) (146 116) (149 20) (177 97) (178 13) (179 9) (192 79) (193 9) (194 7) |
| 334 | 2161,6 | 14,0177 | 50 | (51 63) (53 141) (54 72) (55 238) (65 186) (66 150) (67 581) (68 56) (77 426) (78 197) (79 999) (80 318) (81 181) (82 68) (83 68) (91 711) (92 133) (93 413) (94 103) (95 130) (96 28) (104 42) (105 241) (106 97) (107 95) (108 86) (109 32) (115 33) (117 104) (118 31) (119 212) (120 108) (121 50) (122 72) (132 47) (133 81) (134 42) (135 31) (136 24) (143 16) (144 21) (145 26) (146 64) (155 29) (159 17) (160 19) (162 12) (173 14) (175 31) (214 13) |
| 335 | 2168,4 | 14,073 | 62 | (53 150) (54 32) (55 349) (59 90) (63 17) (65 166) (66 151) (67 565) (68 67) (69 245) (70 61) (71 67) (74 98) (77 390) (78 189) (79 999) (80 241) (81 173) (83 96) (84 30) (85 116) (87 33) (88 16) (91 786) (92 171) (93 353) (94 114) (95 81) (97 40) (104 58) (105 304) (106 176) (107 113) (108 157) (109 34) (111 55) (115 60) (118 33) (119 214) (120 73) (121 48) (122 18) (131 100) (133 102) (134 33) (135 29) (141 20) (145 50) (146 21) (148 31) (153 17) (154 14) (159 21) (160 25) (161 28) (162 14) (173 23) (175 22) (180 22) (201 28) (215 13) (241 31) |
| 336 | 2191,2 | 14,2587 | 86 | (53 107) (54 70) (55 460) (56 82) (58 54) (61 65) (65 94) (66 105) (67 403) (68 79) (69 131) (72 52) (73 999) (74 107) (75 958) (76 62) (77 289) (78 125) (79 746) (80 210) (81 264) (82 65) (83 80) (84 82) (89 28) (91 514) (92 126) (93 304) (94 224) (95 120) (96 84) (97 48) (98 71) (99 30) (104 39) (105 269) (106 180) (107 105) (108 38) (109 65) (110 23) (111 34) (112 19) (115 40) (116 61) (117 555) (118 75) (119 188) (120 83) (121 62) (122 17) (123 36) (129 312) (130 48) (131 134) (132 57) (133 101) (134 52) (135 34) (141 22) (143 24) (145 136) (146 42) (155 31) (157 36) (158 14) (159 41) (160 20) (161 43) (171 38) (173 30) (175 13) (185 27) (187 26) (197 18) (199 22) (201 18) (213 9) (227 9) (236 16) (240 8) (278 14) (352 11) (353 90) (354 19) (368 7) |
| 337 | 2195,2 | 14,2911 | 161 | (56 207) (58 34) (59 45) (60 17) (61 98) (62 7) (63 5) (70 115) (74 181) (76 110) (83 269) (84 225) (86 21) (87 14) (88 11) (89 44) (97 224) (98 194) (99 55) (100 8) (101 22) (102 5) (103 25) (104 4) (110 123) (111 83) (112 42) (115 11) (116 102) (117 999) (118 101) (119 68) (123 85) (124 55) (125 32) (126 9) (129 922) (130 117) (131 149) (132 137) (133 84) (134 28) (137 49) (138 39) (139 16) (140 6) (143 59) (144 7) (145 282) (146 37) (147 50) (148 15) (151 29) (152 39) (153 12) (154 4) (155 23) (157 24) (158 8) (159 25) (160 4) (161 12) (162 5) (165 18) (166 21) (167 7) (168 6) (169 18) (170 9) (171 48) (172 16) (173 18) (175 9) (176 2) (179 10) (180 20) (181 8) (182 4) (183 30) (184 6) (185 78) (186 18) (187 21) (188 10) (189 9) (193 10) (194 43) (195 11) (197 11) (198 3) (199 77) (200 14) (201 23) (202 4) (203 4) (207 12) (208 11) (209 4) (213 19) (214 4) (215 9) (219 4) (221 14) (222 11) (223 4) (225 4) (227 18) (228 3) (229 6) (234 14) (235 22) (236 63) (237 13) (239 5) (241 16) (242 3) (243 5) (249 15) (250 14) (251 4) (253 4) (255 20) (256 5) (257 7) (260 6) (261 2) (263 3) (264 4) (267 3) (268 4) (269 14) (271 11) (272 5) (278 64) (279 14) (281 3) (283 5) (284 2) (285 6) (286 8) (287 2) (297 4) (300 4) (309 11) (310 3) (311 5) (313 3) (323 3) (325 11) (326 3) (335 2) (338 3) (339 4) (343 2) (353 450) (354 126) (355 34) (356 5) (368 27) (369 8) (370 2) |
| 338 | 2196,1 | 14,2986 | 164 | (51 5) (53 50) (54 146) (55 577) (56 115) (57 125) (58 18) (59 25) (60 11) (61 60) (62 4) (63 3) (65 28) (66 22) (67 353) (68 124) (69 266) (70 57) (71 44) (72 44) (73 999) (74 103) (75 941) (76 66) (77 76) (78 13) (79 128) (80 61) (81 287) (82 139) (83 147) (84 109) (85 42) (86 12) (87 8) (88 6) (89 27) (90 2) (91 42) (92 10) (93 64) (94 42) (95 188) (96 182) (97 118) (99 32) (100 4) (101 11) (102 2) (104 2) (105 24) (106 7) (107 35) (108 24) (109 89) (110 71) (111 45) (112 20) (113 9) (114 2) (115 6) (116 51) (118 48) (119 36) (120 8) (121 42) (122 17) (123 52) (124 31) (125 18) (126 5) (127 4) (131 80) (132 65) (133 40) (134 14) (135 34) (136 18) (137 28) (138 21) (139 9) (140 3) (142 3) (143 28) (144 4) (148 7) (149 17) (150 20) (151 17) (152 19) (153 7) (154 2) (155 13) (156 7) (157 14) (158 4) (159 12) (160 2) (161 6) (162 3) (163 9) (164 7) (165 9) (166 10) (167 5) (168 3) (169 10) (170 5) (171 24) (172 8) (173 12) (174 4) (175 5) (176 1) (177 5) (178 5) (179 5) (180 9) (181 5) (183 14) (184 3) (185 36) (186 8) (187 13) (188 5) (192 10) (193 5) (195 5) (196 2) (197 5) (198 2) (200 7) (201 11) (202 2) (209 2) (211 4) (213 8) (214 2) (215 5) (225 2) (227 9) (228 1) (229 4) (234 10) (239 2) (241 6) (243 3) (248 2) (253 2) (257 3) (267 2) (268 2) (269 6) (272 2) (276 18) (277 5) (281 2) (285 2) (295 2) (323 2) (343 1) (351 40) (352 12) (366 2) |
| 339 | 2246,3 | 14,7072 | 74 | (54 23) (55 260) (56 152) (57 999) (58 39) (68 21) (69 144) (70 101) (71 605) (72 27) (82 28) (83 105) (84 53) (85 416) (86 25) (96 17) (97 85) (98 35) (99 121) (100 9) (110 8) (111 42) (112 25) (113 72) (114 6) (124 5) (125 18) (126 18) (127 46) (128 3) (138 3) (139 7) (140 13) (141 31) (142 2) (152 2) (153 3) (154 10) (155 22) (156 2) (166 1) (167 1) (168 7) (169 15) (170 2) (182 6) (183 11) (184 1) (196 5) (197 9) (198 1) (210 4) (212 1) (224 3) (225 5) (226 1) (238 2) (239 4) (240 1) (252 2) (253 4) (254 1) (266 2) (267 3) (268 1) (280 2) (281 3) (294 1) (295 2) (309 1) (338 4) (339 1) (342 1) (363 1) |
| 340 | 2256,1 | 14,7865 | 77 | (51 32) (52 22) (63 12) (65 134) (66 124) (77 399) (78 218) (79 999) (91 838) (92 169) (93 451) (102 6) (103 58) (104 54) (105 350) (106 279) (108 207) (115 54) (118 72) (119 272) (120 138) (131 189) (132 48) (133 175) (134 75) (141 21) (142 16) (143 38) (144 10) (145 87) (146 37) (147 102) (148 40) (154 6) (155 19) (156 8) (157 51) (158 17) (159 66) (160 36) (161 53) (162 24) (169 30) (170 11) (171 52) (172 10) (173 54) (174 22) (175 66) (176 22) (181 8) (183 14) (186 5) (187 62) (188 16) (189 12) (197 23) (198 5) (200 5) (201 27) (202 11) (203 6) (213 8) (215 34) (216 6) (223 7) (224 2) (237 6) (238 10) (239 4) (242 4) (266 3) (305 6) (306 4) (307 4) (328 4) (331 6) |
| 341 | 2261,3 | 14,8287 | 51 | (51 37) (53 65) (63 10) (64 7) (65 128) (66 106) (77 379) (78 199) (79 999) (80 180) (91 902) (92 155) (93 507) (103 42) (104 35) (105 360) (106 136) (107 126) (108 139) (118 49) (119 293) (120 112) (133 162) (134 70) (135 54) (147 86) (148 33) (157 43) (159 49) (160 26) (161 45) (162 20) (169 51) (171 18) (173 42) (174 21) (175 92) (176 43) (187 27) (188 8) (197 23) (201 17) (215 14) (261 11) (265 4) (267 6) (303 4) (305 7) (306 7) (318 4) (370 3) |
| 342 | 2261,8 | 14,833 | 108 | (51 19) (53 81) (55 180) (59 39) (60 13) (61 62) (64 5) (65 65) (72 86) (73 816) (74 138) (75 809) (76 71) (87 12) (90 46) (100 91) (101 20) (102 10) (104 15) (114 15) (115 222) (116 721) (118 24) (122 43) (126 10) (128 682) (130 29) (131 999) (132 61) (136 43) (142 103) (144 643) (146 28) (147 40) (150 12) (154 22) (155 28) (156 36) (158 90) (159 35) (160 14) (164 9) (168 23) (169 24) (170 52) (172 33) (173 20) (174 11) (178 9) (182 25) (184 109) (186 56) (187 20) (196 13) (198 138) (200 76) (201 12) (202 8) (203 22) (210 13) (212 37) (214 23) (216 12) (220 10) (226 59) (227 10) (228 13) (229 7) (230 5) (234 7) (238 36) (240 46) (241 9) (242 11) (252 31) (253 5) (254 54) (255 8) (256 20) (261 10) (262 5) (266 21) (268 36) (270 15) (272 4) (280 12) (282 19) (284 6) (294 14) (296 15) (298 6) (308 11) (310 20) (311 5) (312 5) (317 9) (318 4) (320 3) (322 7) (324 10) (336 22) (338 278) (339 81) (340 22) (352 13) (353 75) (354 22) (355 5) |
| 343 | 2266,8 | 14,8736 | 78 | (53 46) (54 152) (55 271) (56 62) (59 36) (61 72) (65 24) (67 162) (68 43) (69 76) (70 45) (72 71) (73 999) (74 129) (75 966) (76 72) (77 59) (90 33) (91 72) (100 76) (102 14) (106 21) (115 203) (116 644) (120 19) (122 40) (128 657) (130 46) (131 844) (132 72) (136 33) (142 83) (144 644) (145 54) (146 29) (147 31) (156 27) (158 91) (159 19) (164 13) (170 37) (172 24) (173 14) (182 19) (184 90) (186 54) (198 145) (199 19) (200 58) (201 16) (203 26) (212 38) (214 21) (216 9) (226 41) (228 17) (238 17) (240 44) (252 35) (254 51) (255 9) (256 15) (266 39) (267 9) (268 42) (270 14) (282 34) (283 7) (296 13) (310 19) (336 17) (338 248) (339 57) (340 22) (352 8) (353 83) (354 19) (355 6) |
| 344 | 1436,5 | 8,1155 | 53 | (56 31) (59 48) (60 12) (61 18) (72 26) (73 999) (74 158) (75 211) (88 16) (89 23) (90 9) (91 26) (100 29) (102 46) (103 157) (104 20) (105 34) (108 29) (114 16) (116 11) (117 17) (119 16) (128 102) (129 136) (130 27) (131 32) (132 16) (133 71) (134 13) (142 12) (144 181) (145 32) (147 190) (148 34) (149 39) (161 14) (188 16) (189 98) (190 19) (191 11) (203 56) (204 34) (205 71) (206 11) (207 8) (217 25) (218 14) (221 15) (233 7) (234 651) (235 135) (236 59) (237 8) |
| 345 | 2269 | 14,8915 | 35 | (57 999) (71 747) (83 103) (85 552) (86 37) (97 137) (99 138) (110 25) (111 73) (113 70) (125 49) (127 41) (139 20) (140 51) (141 42) (154 46) (155 26) (181 9) (187 162) (188 26) (196 21) (197 11) (204 21) (211 10) (224 10) (281 13) (295 15) (330 9) (357 9) (359 94) (360 26) (361 12) (413 15) (414 5) (429 4) |
| 346 | 2273 | 14,9245 | 114 | (55 195) (58 48) (59 25) (60 9) (61 34) (67 42) (69 40) (72 36) (73 999) (74 99) (75 538) (76 31) (77 35) (79 77) (81 50) (83 74) (84 36) (86 7) (87 5) (89 26) (93 78) (94 9) (95 70) (96 10) (97 28) (101 10) (102 12) (103 42) (105 29) (106 17) (107 19) (109 10) (112 41) (117 601) (118 73) (119 57) (120 15) (121 17) (123 6) (124 5) (125 17) (129 310) (130 43) (132 206) (133 85) (134 27) (135 26) (143 19) (145 163) (146 30) (147 72) (148 18) (149 8) (159 25) (160 5) (164 4) (165 5) (166 3) (167 8) (171 15) (174 16) (176 3) (185 24) (187 17) (188 7) (189 11) (191 15) (199 11) (201 28) (202 7) (204 10) (205 13) (207 4) (213 4) (215 8) (217 29) (218 15) (220 6) (221 4) (227 12) (228 3) (229 6) (241 8) (243 10) (244 3) (255 3) (257 7) (262 5) (269 3) (271 4) (277 2) (285 6) (298 2) (299 6) (305 3) (307 5) (312 1) (313 4) (319 5) (320 2) (325 11) (326 2) (327 3) (331 5) (332 2) (341 11) (342 3) (357 2) (369 147) (370 41) (371 10) (384 19) (385 5) (421 2) |
| 347 | 2316,5 | 15,2786 | 91 | (53 81) (54 279) (55 999) (56 313) (57 357) (58 37) (60 7) (65 28) (66 19) (67 308) (68 129) (69 659) (70 316) (71 157) (72 12) (77 17) (79 99) (80 53) (81 185) (82 247) (83 474) (84 175) (86 8) (93 39) (94 67) (95 94) (96 191) (97 376) (98 98) (107 22) (108 59) (109 39) (110 126) (111 134) (112 102) (120 11) (121 11) (122 316) (123 86) (124 88) (125 46) (126 23) (134 18) (135 9) (136 258) (137 71) (138 70) (139 20) (140 8) (148 11) (150 108) (151 32) (152 54) (153 15) (154 7) (164 72) (165 19) (166 45) (167 12) (178 65) (179 12) (180 31) (181 7) (192 52) (193 11) (194 22) (195 6) (206 49) (207 13) (208 15) (220 43) (221 12) (222 10) (234 43) (235 11) (236 8) (248 40) (249 11) (250 5) (262 45) (263 12) (264 5) (276 54) (277 13) (278 9) (290 31) (291 8) (304 8) (318 7) (319 30) (320 6) |
| 348 | 2332,6 | 15,4094 | 39 | (53 26) (55 315) (56 167) (57 999) (58 61) (67 106) (68 32) (69 196) (70 96) (71 690) (72 39) (77 41) (80 34) (81 41) (82 37) (83 123) (84 63) (85 505) (86 30) (91 66) (96 23) (97 120) (98 40) (99 149) (104 26) (105 37) (111 80) (113 76) (125 50) (127 54) (140 29) (141 31) (148 25) (154 60) (155 31) (169 18) (211 14) (238 13) (239 12) |
| 349 | 2338,4 | 15,457 | 35 | (55 288) (56 117) (57 999) (58 64) (67 88) (68 46) (69 477) (70 135) (71 264) (79 89) (80 30) (81 154) (83 101) (85 170) (93 80) (95 95) (97 44) (98 270) (99 70) (105 65) (107 103) (109 84) (111 35) (113 30) (119 38) (121 61) (123 24) (124 15) (135 22) (136 20) (161 22) (191 20) (203 24) (294 16) (295 17) |
| 350 | 2354,3 | 15,5864 | 17 | (56 71) (57 421) (70 191) (71 292) (83 100) (85 73) (104 71) (112 44) (113 73) (114 7) (149 999) (150 105) (151 15) (167 259) (168 23) (279 43) (280 8) |
| 351 | 2356 | 15,5998 | 136 | (54 47) (55 144) (58 10) (59 68) (60 5) (67 165) (68 20) (69 58) (72 8) (73 999) (74 85) (80 70) (81 90) (82 11) (83 40) (84 64) (85 40) (87 7) (89 35) (90 2) (93 84) (95 15) (96 19) (97 42) (98 36) (99 21) (101 102) (102 13) (103 271) (107 33) (109 50) (110 20) (111 28) (115 8) (116 9) (121 33) (122 65) (123 32) (124 7) (125 7) (129 297) (130 53) (131 64) (133 136) (134 54) (135 102) (136 24) (137 12) (138 3) (139 2) (147 345) (148 83) (151 6) (152 6) (153 3) (161 9) (162 24) (163 15) (164 4) (165 4) (175 29) (176 10) (177 7) (178 3) (179 3) (188 5) (189 19) (190 8) (191 9) (193 2) (194 2) (201 27) (202 5) (203 66) (204 20) (205 43) (206 8) (207 4) (213 5) (214 8) (215 14) (216 3) (217 18) (218 9) (219 16) (220 5) (221 4) (229 4) (230 2) (231 7) (232 31) (233 8) (234 2) (237 13) (238 3) (239 5) (244 1) (245 2) (247 1) (249 4) (252 1) (256 2) (257 5) (258 1) (270 1) (275 17) (276 4) (281 2) (282 2) (283 1) (295 2) (296 2) (305 1) (307 1) (308 3) (309 3) (321 3) (322 1) (335 1) (337 1) (365 40) (366 11) (367 4) (369 11) (370 3) (377 2) (378 10) (379 3) (380 1) (397 1) (398 1) (453 15) (454 6) (455 2) (457 1) (468 1) |
| 352 | 2357,7 | 15,6141 | 103 | (51 27) (52 19) (53 95) (58 12) (60 10) (61 49) (62 4) (63 11) (65 131) (66 136) (67 619) (68 53) (72 56) (74 110) (75 700) (77 396) (78 228) (79 999) (80 275) (81 169) (82 28) (88 11) (90 4) (91 860) (92 191) (93 397) (94 114) (95 132) (104 37) (105 336) (106 238) (107 123) (108 199) (115 64) (116 86) (117 464) (118 94) (119 273) (120 86) (121 53) (127 8) (128 31) (131 202) (132 99) (142 13) (143 62) (144 26) (145 113) (146 49) (155 23) (156 11) (157 54) (158 19) (159 79) (160 27) (161 48) (169 26) (170 9) (171 44) (172 16) (173 48) (174 19) (180 8) (181 17) (182 5) (183 21) (184 7) (185 31) (186 13) (187 22) (188 9) (193 3) (195 7) (196 3) (197 15) (198 6) (199 27) (200 9) (209 6) (210 3) (211 9) (212 4) (213 15) (223 19) (224 13) (225 10) (226 3) (227 8) (228 3) (235 3) (239 6) (240 2) (241 13) (242 2) (251 2) (253 3) (263 2) (264 4) (265 2) (267 9) (268 4) (291 2) (331 2) |
| 353 | 2367,2 | 15,691 | 30 | (53 62) (65 121) (66 114) (67 515) (77 318) (78 197) (79 999) (80 323) (91 713) (92 167) (93 386) (94 172) (105 237) (106 106) (107 117) (108 167) (119 197) (120 80) (121 45) (122 36) (136 17) (144 25) (158 9) (160 11) (162 17) (171 13) (225 9) (266 6) (306 5) (358 15) |
| 354 | 2372,3 | 15,7328 | 87 | (50 1) (51 2) (53 14) (54 24) (55 271) (56 150) (57 999) (58 39) (65 3) (66 2) (67 32) (68 27) (69 164) (70 106) (71 618) (72 31) (82 33) (83 114) (84 56) (85 433) (86 28) (96 20) (97 96) (98 35) (99 130) (100 10) (110 10) (111 50) (112 28) (113 78) (114 7) (124 6) (125 22) (126 20) (127 52) (128 4) (138 4) (139 8) (140 15) (141 37) (142 4) (152 2) (153 4) (154 12) (155 27) (156 3) (166 2) (167 3) (168 9) (169 20) (170 3) (180 1) (181 2) (182 7) (183 15) (184 2) (194 1) (195 1) (196 5) (197 12) (198 2) (210 4) (211 9) (212 1) (224 3) (225 7) (226 1) (238 2) (252 2) (253 4) (254 1) (266 2) (267 3) (268 1) (280 2) (281 3) (294 1) (295 3) (296 1) (308 1) (309 3) (310 1) (322 1) (323 2) (337 1) (366 3) (367 1) |
| 355 | 2408,2 | 16,0246 | 78 | (59 60) (61 17) (65 35) (66 36) (67 283) (73 999) (74 87) (75 178) (76 15) (77 92) (78 53) (79 294) (80 145) (81 149) (89 32) (91 168) (92 44) (93 167) (94 46) (95 75) (101 98) (103 274) (104 33) (105 98) (106 41) (107 68) (108 17) (109 30) (110 24) (115 25) (116 47) (117 99) (118 20) (119 39) (120 18) (121 49) (122 19) (123 33) (129 283) (130 47) (131 110) (132 28) (133 118) (134 24) (135 44) (136 54) (137 16) (146 14) (147 336) (148 71) (149 95) (150 24) (151 10) (161 23) (162 41) (163 24) (175 34) (176 9) (187 10) (189 13) (201 21) (202 9) (203 58) (205 45) (219 16) (229 7) (237 9) (246 24) (247 8) (289 16) (308 7) (379 33) (380 9) (392 10) (452 15) (453 7) (467 14) (468 5) |
| 356 | 2409,2 | 16,0329 | 61 | (53 43) (54 60) (55 579) (56 317) (58 49) (65 24) (66 25) (67 223) (68 71) (69 395) (70 204) (71 999) (72 58) (81 103) (82 67) (83 255) (84 102) (85 675) (86 44) (95 45) (96 37) (97 194) (98 60) (99 206) (100 15) (110 24) (111 107) (112 31) (113 123) (124 13) (125 41) (126 17) (127 70) (138 6) (139 10) (141 55) (152 6) (153 14) (154 11) (155 40) (168 8) (183 27) (197 26) (211 23) (212 5) (225 21) (226 5) (239 19) (253 17) (267 13) (281 9) (295 7) (308 5) (309 5) (336 18) (337 46) (338 12) (365 9) (452 10) (453 5) (454 2) |
| 357 | 2443,8 | 16,3149 | 77 | (51 51) (53 31) (55 87) (59 41) (65 28) (66 37) (67 169) (68 21) (70 44) (73 999) (74 69) (75 151) (77 79) (78 49) (79 272) (80 76) (81 37) (87 16) (89 41) (91 171) (92 26) (93 105) (94 40) (95 40) (101 30) (103 572) (104 52) (105 120) (106 22) (107 22) (108 75) (113 33) (115 22) (119 61) (120 15) (121 16) (122 26) (128 15) (129 283) (130 37) (131 85) (133 63) (134 19) (135 23) (147 201) (148 44) (149 34) (156 15) (159 13) (160 12) (161 37) (162 17) (163 13) (169 36) (174 13) (189 32) (191 107) (192 20) (202 7) (217 130) (218 66) (219 26) (221 14) (230 11) (243 27) (259 119) (260 22) (261 9) (271 17) (275 5) (291 8) (331 19) (349 14) (361 123) (362 44) (363 14) (404 4) |
| 358 | 2446,5 | 16,3364 | 100 | (56 182) (58 58) (60 8) (61 15) (69 592) (71 488) (72 57) (76 17) (83 222) (84 43) (95 151) (96 22) (97 207) (98 74) (101 226) (102 21) (109 108) (110 18) (111 173) (112 54) (116 201) (117 211) (118 25) (123 97) (124 11) (125 58) (132 64) (133 146) (137 46) (138 8) (139 30) (140 24) (143 47) (144 6) (145 163) (146 50) (147 491) (148 70) (149 72) (151 47) (153 11) (159 10) (163 21) (165 23) (166 4) (167 9) (173 29) (174 8) (175 60) (176 8) (179 11) (188 33) (201 74) (202 11) (203 172) (204 27) (205 171) (206 31) (207 20) (215 200) (216 35) (221 12) (228 6) (237 37) (238 7) (275 5) (277 16) (278 8) (285 20) (286 5) (289 16) (290 3) (292 3) (293 6) (295 8) (305 31) (306 7) (327 7) (337 14) (338 3) (355 5) (367 10) (369 37) (370 11) (371 3) (383 6) (384 3) (385 3) (397 4) (425 18) (426 3) (427 999) (428 333) (429 88) (430 16) (439 5) (440 3) (443 4) (457 14) (459 6) |
| 359 | 2460,3 | 16,4488 | 70 | (59 45) (65 26) (66 28) (67 196) (73 999) (74 101) (75 103) (77 52) (79 230) (80 114) (81 93) (84 47) (89 37) (91 139) (92 51) (93 124) (94 39) (95 81) (101 74) (103 240) (104 25) (105 73) (106 32) (107 51) (116 41) (117 141) (118 28) (119 37) (120 16) (121 44) (129 143) (131 60) (133 95) (134 18) (135 26) (147 308) (148 53) (149 50) (150 37) (160 29) (161 23) (162 32) (163 29) (164 13) (175 23) (176 12) (179 10) (187 14) (189 24) (201 18) (204 209) (205 103) (206 23) (217 82) (229 12) (230 15) (231 16) (260 18) (264 33) (271 20) (303 12) (319 51) (320 14) (361 108) (362 33) (393 16) (480 8) (481 14) (482 6) (483 4) |
| 360 | 2466,3 | 16,4976 | 215 | (51 7) (52 7) (53 37) (54 28) (55 159) (56 8) (57 18) (58 10) (59 54) (60 5) (61 13) (62 2) (63 3) (65 42) (66 51) (67 303) (68 32) (69 69) (70 33) (71 28) (72 21) (73 999) (74 85) (75 180) (76 12) (77 121) (78 71) (79 426) (80 126) (81 112) (82 23) (83 20) (84 30) (85 20) (86 2) (87 7) (88 6) (89 27) (90 3) (91 274) (92 61) (93 189) (94 58) (95 105) (96 15) (97 21) (98 13) (99 10) (101 86) (102 11) (103 263) (104 45) (105 167) (106 50) (107 64) (108 128) (109 34) (110 7) (111 12) (112 3) (115 29) (116 41) (117 145) (118 34) (119 108) (120 35) (121 43) (122 45) (123 17) (124 3) (125 4) (127 2) (128 5) (129 306) (130 51) (131 135) (132 36) (133 134) (134 36) (135 62) (136 12) (137 7) (138 2) (139 2) (141 6) (142 3) (143 15) (144 8) (145 44) (146 14) (147 326) (148 63) (149 50) (150 9) (151 5) (153 2) (155 8) (156 2) (157 12) (158 6) (159 29) (160 13) (161 51) (162 31) (163 16) (164 3) (165 3) (169 7) (170 2) (171 22) (172 6) (173 22) (174 19) (175 30) (176 11) (177 5) (178 4) (179 3) (180 1) (181 3) (182 1) (183 7) (184 2) (185 9) (186 2) (187 14) (188 6) (189 23) (190 9) (193 2) (195 2) (197 6) (198 2) (199 7) (200 1) (201 26) (202 11) (203 63) (205 39) (206 8) (207 4) (209 2) (211 4) (212 1) (213 5) (214 3) (215 7) (216 4) (219 14) (220 3) (221 3) (222 1) (223 2) (225 1) (227 3) (228 1) (229 5) (230 3) (231 2) (235 1) (237 9) (238 2) (239 4) (240 3) (241 6) (242 2) (253 1) (254 1) (255 3) (256 1) (257 4) (258 10) (259 4) (260 1) (265 2) (267 1) (268 1) (269 2) (275 3) (276 1) (277 1) (281 1) (283 2) (293 1) (295 1) (296 1) (301 9) (302 2) (308 3) (309 2) (321 3) (322 1) (324 1) (335 3) (336 1) (347 1) (348 1) (349 1) (375 1) (391 27) (392 8) (393 2) (398 1) (404 3) (405 1) (423 1) (425 2) (426 1) (438 1) (479 17) (480 6) (481 2) (482 1) (494 4) (495 1) |
| 361 | 2477,6 | 16,5899 | 86 | (53 29) (54 55) (55 367) (59 49) (60 9) (61 20) (65 24) (67 279) (68 46) (69 157) (70 21) (72 20) (73 999) (74 84) (75 244) (76 21) (77 75) (78 44) (79 282) (81 150) (82 32) (83 78) (89 28) (93 141) (95 154) (96 25) (97 41) (98 15) (101 100) (102 11) (103 264) (104 26) (105 79) (107 51) (108 70) (109 39) (116 66) (117 108) (118 21) (121 50) (122 14) (123 18) (124 7) (129 416) (130 58) (131 116) (132 37) (133 119) (134 25) (135 48) (136 12) (137 11) (143 13) (145 27) (146 14) (147 360) (148 58) (149 60) (159 18) (161 18) (163 15) (171 9) (173 11) (175 15) (187 16) (189 15) (201 32) (202 10) (203 67) (205 50) (206 10) (218 13) (219 15) (237 10) (257 10) (260 5) (261 5) (265 13) (303 14) (304 4) (391 4) (393 19) (394 6) (406 6) (481 7) (482 3) |
| 362 | 2490,6 | 16,6958 | 180 | (51 1) (55 85) (56 40) (63 1) (67 14) (70 29) (82 11) (83 35) (85 105) (86 7) (93 1) (95 2) (96 6) (97 30) (98 9) (99 48) (106 1) (110 4) (111 23) (113 46) (114 8) (120 1) (121 1) (123 1) (124 3) (125 10) (126 6) (127 30) (135 12) (136 1) (137 1) (138 2) (139 13) (140 6) (141 23) (143 53) (144 7) (145 12) (148 104) (149 84) (150 10) (151 7) (152 2) (153 16) (155 79) (157 52) (158 8) (159 11) (161 7) (163 14) (164 2) (165 1) (166 1) (167 5) (169 332) (170 56) (171 30) (173 19) (174 3) (175 13) (176 2) (177 14) (178 2) (179 2) (180 1) (181 6) (183 20) (184 3) (185 5) (187 4) (189 96) (191 613) (192 111) (193 54) (194 6) (195 2) (197 5) (199 9) (201 6) (204 211) (205 78) (206 26) (207 16) (208 3) (209 2) (213 1) (215 11) (217 388) (218 96) (219 44) (220 7) (221 27) (222 6) (223 4) (227 11) (229 31) (230 18) (231 28) (232 7) (233 17) (234 5) (235 2) (239 3) (241 13) (243 154) (244 37) (245 34) (246 7) (247 8) (248 1) (249 1) (255 6) (257 14) (258 4) (259 11) (260 2) (261 1) (263 9) (264 2) (265 6) (266 2) (271 157) (272 38) (273 20) (274 3) (275 2) (277 2) (279 7) (280 2) (287 3) (288 1) (289 4) (291 32) (292 10) (293 6) (294 2) (303 2) (305 31) (306 13) (307 7) (308 2) (317 14) (319 62) (320 20) (321 10) (322 2) (331 84) (332 30) (333 17) (334 4) (335 2) (345 12) (346 4) (347 4) (348 1) (349 2) (361 999) (362 323) (363 159) (364 35) (365 9) (366 1) (373 2) (374 1) (377 5) (378 2) (379 3) (380 1) (393 2) (405 1) (435 9) (436 4) (437 2) (450 1) (451 5) (452 2) (453 1) (463 5) (464 2) (465 1) |
| 363 | 2491,2 | 16,7004 | 122 | (53 5) (54 7) (55 28) (56 12) (59 23) (60 2) (61 5) (65 1) (66 2) (67 5) (69 25) (73 999) (74 84) (75 91) (76 6) (77 4) (79 1) (81 14) (82 4) (87 5) (89 15) (90 1) (91 1) (101 18) (103 143) (104 14) (105 7) (109 7) (111 7) (113 15) (114 3) (115 6) (117 53) (118 6) (119 7) (120 1) (127 9) (129 142) (130 18) (131 27) (133 45) (134 6) (135 5) (139 5) (143 19) (144 2) (145 4) (147 251) (148 41) (149 33) (150 4) (151 3) (152 1) (153 6) (155 29) (157 18) (158 3) (159 4) (161 2) (163 5) (164 1) (167 1) (169 123) (170 20) (171 11) (173 6) (175 4) (177 4) (178 1) (181 2) (183 6) (184 1) (185 1) (187 1) (189 30) (191 203) (192 37) (193 18) (194 2) (195 1) (199 3) (201 2) (204 63) (205 23) (206 8) (207 5) (208 1) (215 3) (217 115) (218 28) (219 13) (221 8) (222 2) (223 1) (227 3) (229 9) (230 5) (231 8) (232 2) (234 1) (235 1) (241 4) (243 47) (244 11) (245 10) (246 2) (247 2) (255 2) (257 4) (259 3) (271 46) (272 11) (273 6) (274 1) (279 2) (287 1) (305 9) (332 8) (333 5) (334 1) (345 4) (373 1) |
| 364 | 2491,6 | 16,704 | 59 | (52 1) (53 4) (54 5) (59 24) (60 2) (61 5) (65 1) (66 2) (67 3) (69 16) (73 999) (74 86) (75 92) (76 5) (77 4) (79 1) (81 12) (82 2) (87 5) (89 14) (90 1) (91 1) (101 18) (103 121) (104 12) (105 6) (109 6) (113 8) (114 2) (115 5) (117 41) (118 5) (119 5) (129 109) (130 14) (131 20) (133 35) (134 5) (135 4) (139 3) (143 14) (144 2) (145 3) (147 183) (148 30) (149 24) (150 3) (151 2) (153 4) (155 20) (157 12) (158 2) (159 3) (161 1) (163 3) (169 87) (170 14) (171 7) (173 4) |
| 365 | 2494,2 | 16,7245 | 39 | (54 44) (61 79) (73 999) (74 302) (79 32) (82 57) (84 72) (87 41) (110 14) (114 22) (117 669) (119 132) (141 255) (152 32) (159 57) (170 56) (171 64) (174 80) (182 10) (197 8) (200 44) (205 69) (208 11) (228 197) (233 44) (246 15) (264 10) (269 14) (279 16) (291 35) (319 90) (321 19) (346 14) (357 6) (358 35) (377 11) (451 9) (452 5) (464 5) |
| 366 | 2495,9 | 16,7386 | 32 | (59 295) (61 36) (83 19) (87 24) (117 131) (131 73) (133 247) (138 7) (141 135) (148 197) (149 192) (161 20) (174 62) (192 108) (193 36) (208 6) (214 10) (216 999) (230 20) (245 33) (269 13) (289 32) (290 93) (304 175) (307 11) (308 3) (318 239) (332 23) (333 17) (361 525) (363 63) (364 43) |
| 367 | 2496,2 | 16,7409 | 54 | (58 864) (64 16) (68 160) (69 64) (77 43) (78 26) (86 132) (90 40) (98 20) (100 58) (107 8) (108 23) (112 54) (114 46) (116 999) (118 62) (119 53) (135 58) (137 6) (140 79) (145 88) (151 41) (157 193) (159 30) (160 50) (170 48) (171 78) (175 71) (176 70) (179 9) (189 102) (191 983) (198 5) (200 45) (202 44) (203 840) (217 895) (218 55) (220 25) (231 70) (247 16) (257 19) (259 14) (261 4) (265 26) (291 209) (303 9) (305 295) (319 392) (333 25) (334 14) (361 169) (379 11) (437 9) |
| 368 | 2497,2 | 16,7491 | 15 | (70 732) (79 62) (126 24) (141 47) (172 70) (174 46) (194 25) (204 574) (205 155) (222 23) (256 193) (269 19) (290 335) (330 999) (363 222) |
| 369 | 2498,4 | 16,759 | 173 | (52 1) (53 5) (54 7) (55 15) (56 4) (57 2) (58 6) (59 30) (60 3) (61 6) (65 1) (66 2) (67 3) (68 2) (69 14) (70 5) (73 999) (74 87) (75 95) (76 6) (77 4) (80 1) (81 12) (82 1) (83 3) (85 5) (86 1) (87 3) (89 16) (90 2) (91 1) (95 1) (97 2) (99 5) (101 25) (103 132) (104 10) (105 5) (109 6) (110 1) (111 6) (113 12) (114 7) (115 9) (117 56) (118 6) (119 7) (120 1) (125 1) (127 4) (129 129) (130 17) (131 25) (133 48) (134 7) (135 5) (139 4) (140 1) (141 4) (143 16) (144 2) (145 7) (147 261) (148 42) (149 33) (150 3) (151 2) (153 5) (155 27) (157 15) (158 3) (159 4) (160 1) (161 2) (163 5) (164 1) (167 1) (168 1) (169 118) (170 19) (171 10) (173 6) (175 5) (176 1) (177 4) (181 2) (183 5) (184 1) (187 1) (199 3) (201 2) (208 1) (209 1) (215 3) (220 3) (221 9) (222 2) (223 1) (227 4) (228 1) (229 10) (230 7) (231 9) (232 3) (233 4) (234 2) (235 1) (239 1) (241 4) (243 57) (244 13) (245 13) (246 3) (247 3) (255 2) (256 1) (257 6) (258 1) (259 5) (260 1) (263 3) (264 1) (265 3) (271 60) (272 13) (273 7) (274 1) (275 1) (278 1) (279 3) (280 2) (281 1) (287 1) (289 2) (291 13) (292 4) (293 2) (294 1) (303 1) (304 2) (305 14) (306 5) (307 3) (308 1) (317 6) (319 24) (320 7) (321 3) (322 1) (324 2) (331 30) (332 10) (333 6) (334 1) (335 1) (345 5) (346 2) (347 1) (349 1) (361 335) (362 101) (363 48) (364 10) (365 2) (373 1) (377 2) (378 1) (379 1) (393 1) (435 2) (436 1) (451 1) (463 1) |
| 370 | 2498,5 | 16,7599 | 219 | (53 12) (54 15) (55 34) (56 9) (58 19) (59 75) (60 8) (61 15) (63 1) (65 3) (66 6) (67 6) (68 6) (69 35) (75 237) (76 17) (77 11) (80 3) (81 29) (82 3) (83 6) (85 12) (86 3) (87 10) (89 40) (90 4) (91 4) (94 1) (95 2) (96 1) (97 5) (99 17) (101 66) (103 367) (104 30) (105 14) (106 1) (107 1) (109 16) (111 15) (113 34) (114 18) (115 22) (117 166) (118 18) (119 22) (120 2) (121 1) (123 1) (125 3) (127 12) (129 376) (130 47) (131 74) (133 138) (134 19) (135 15) (136 1) (137 1) (139 9) (140 3) (141 11) (143 50) (144 4) (145 21) (147 736) (148 112) (149 92) (150 9) (151 7) (152 1) (153 13) (155 71) (157 45) (158 8) (159 12) (160 2) (161 7) (162 1) (163 15) (164 2) (165 2) (166 1) (167 3) (168 3) (169 326) (170 55) (171 29) (173 19) (175 16) (176 3) (177 14) (178 1) (179 1) (181 5) (183 14) (184 2) (185 4) (189 93) (191 670) (192 111) (193 53) (194 5) (195 2) (197 1) (199 9) (201 7) (204 220) (205 81) (206 26) (207 18) (208 3) (209 2) (211 1) (213 1) (215 11) (217 424) (218 101) (219 45) (220 7) (221 30) (222 6) (223 4) (227 12) (228 4) (229 33) (230 21) (231 30) (232 9) (233 14) (234 5) (235 2) (239 2) (241 13) (243 169) (244 38) (245 40) (246 8) (247 8) (248 1) (249 1) (255 7) (257 20) (258 4) (259 15) (260 3) (261 1) (263 9) (264 3) (265 8) (266 1) (267 1) (270 1) (271 184) (272 42) (273 22) (274 3) (275 2) (277 1) (278 3) (279 9) (280 6) (281 2) (287 4) (288 1) (289 5) (291 42) (292 13) (293 8) (294 2) (295 1) (296 1) (303 2) (304 5) (305 42) (306 17) (307 8) (308 2) (317 17) (319 73) (320 22) (321 11) (322 2) (324 6) (325 1) (331 94) (332 31) (333 19) (334 5) (335 2) (343 1) (345 15) (346 5) (347 4) (348 1) (349 2) (359 1) (361 999) (362 314) (363 152) (364 30) (365 8) (366 1) (368 1) (373 2) (377 5) (378 2) (379 3) (380 1) (393 2) (394 1) (405 1) (435 7) (436 2) (437 1) (451 4) (452 1) (463 3) (464 1) |
| 371 | 2499,5 | 16,7681 | 23 | (71 221) (72 908) (79 134) (88 151) (98 70) (100 63) (102 258) (116 867) (128 101) (132 363) (142 376) (146 73) (154 104) (156 306) (188 33) (190 610) (196 10) (203 999) (216 124) (242 140) (290 171) (318 118) (360 24) |
| 372 | 2509,2 | 16,8471 | 69 | (53 19) (54 36) (55 337) (56 170) (57 999) (58 56) (68 35) (69 205) (70 129) (71 734) (72 55) (82 68) (83 204) (84 73) (85 585) (86 39) (96 44) (97 171) (98 35) (99 158) (100 26) (110 25) (111 101) (113 81) (114 15) (124 23) (125 56) (127 51) (128 51) (131 89) (138 13) (139 24) (141 36) (142 11) (144 43) (153 13) (154 43) (160 15) (161 26) (168 37) (169 33) (182 23) (183 24) (184 7) (196 19) (197 16) (198 8) (204 169) (205 69) (206 23) (211 13) (223 23) (224 16) (225 15) (238 9) (240 5) (252 10) (266 11) (267 13) (268 5) (280 9) (296 5) (311 18) (312 3) (324 4) (394 24) (395 9) (409 11) (410 4) |
| 373 | 2538,4 | 17,0846 | 47 | (65 35) (66 47) (67 193) (73 999) (74 85) (75 198) (77 115) (78 52) (79 352) (80 99) (81 70) (91 243) (92 60) (93 157) (94 59) (95 70) (101 85) (103 785) (104 96) (105 180) (106 63) (107 55) (108 80) (115 57) (117 119) (118 34) (119 102) (120 27) (121 35) (122 16) (123 18) (129 389) (130 66) (131 137) (133 81) (134 24) (145 53) (146 19) (147 216) (148 56) (159 27) (171 22) (201 39) (202 11) (203 38) (218 47) (219 32) |
| 374 | 2576,2 | 17,3922 | 71 | (53 32) (54 40) (55 268) (56 49) (59 49) (65 43) (66 53) (67 318) (68 38) (69 172) (73 999) (74 81) (75 177) (77 125) (78 63) (79 436) (80 136) (81 140) (82 35) (83 57) (89 33) (91 269) (92 67) (93 206) (94 77) (95 118) (97 50) (101 95) (103 288) (104 45) (105 173) (106 56) (107 90) (108 113) (109 43) (116 49) (117 150) (118 27) (119 119) (120 29) (121 40) (122 43) (128 17) (129 413) (130 56) (131 170) (132 38) (133 139) (134 32) (135 71) (136 14) (143 26) (145 35) (147 328) (148 71) (149 49) (157 18) (159 25) (161 37) (162 14) (173 19) (175 35) (199 14) (200 9) (201 28) (203 64) (204 86) (205 50) (217 36) (286 9) (419 19) |
| 375 | 2580,2 | 17,4246 | 59 | (54 119) (55 461) (56 108) (67 408) (68 89) (69 292) (73 999) (74 101) (75 204) (77 72) (79 164) (80 117) (81 247) (82 95) (83 130) (91 83) (93 99) (95 222) (96 65) (97 92) (101 148) (103 362) (104 36) (105 59) (106 38) (107 51) (109 83) (110 27) (116 72) (117 164) (118 28) (121 36) (123 34) (129 581) (130 82) (131 166) (132 40) (133 85) (135 33) (147 283) (148 59) (149 57) (150 22) (161 22) (188 11) (201 27) (203 88) (219 33) (237 16) (290 22) (291 18) (333 67) (334 16) (423 43) (425 33) (426 9) (436 38) (437 14) (438 9) |
| 376 | 2726,4 | 18,6148 | 166 | (53 29) (55 251) (56 35) (57 185) (59 40) (61 36) (65 25) (67 160) (68 22) (69 191) (70 25) (71 86) (72 20) (73 999) (74 91) (75 483) (76 40) (77 102) (78 21) (79 216) (80 26) (81 304) (82 27) (83 94) (89 32) (91 281) (92 46) (93 213) (94 46) (95 287) (96 21) (97 47) (101 43) (102 12) (103 112) (104 28) (105 321) (106 68) (107 231) (108 45) (109 130) (110 17) (111 41) (114 7) (115 51) (116 34) (117 133) (118 37) (119 421) (120 130) (121 189) (122 29) (123 54) (125 15) (128 23) (129 744) (130 122) (131 163) (132 51) (135 103) (136 15) (137 25) (142 28) (143 117) (144 37) (145 189) (146 54) (147 150) (148 37) (149 63) (150 10) (151 14) (153 9) (155 41) (156 18) (157 58) (158 32) (159 118) (160 69) (161 98) (162 24) (163 63) (165 12) (168 6) (169 23) (171 40) (172 16) (173 56) (174 19) (175 40) (176 8) (178 11) (179 13) (181 11) (183 13) (184 6) (185 32) (186 11) (187 22) (188 8) (189 34) (191 20) (193 17) (195 8) (197 9) (198 5) (199 30) (200 14) (201 27) (202 9) (203 42) (204 24) (213 50) (214 17) (215 28) (216 6) (217 48) (218 10) (219 30) (221 9) (227 11) (228 10) (229 14) (230 7) (231 12) (233 25) (234 5) (235 5) (241 9) (245 16) (246 12) (247 68) (248 13) (255 74) (256 14) (257 7) (259 17) (260 10) (261 7) (273 14) (274 11) (275 22) (276 6) (291 5) (301 17) (302 4) (314 3) (326 13) (327 16) (328 61) (329 239) (330 58) (331 8) (339 7) (340 11) (353 114) (354 32) (367 7) (368 199) (369 63) (370 8) (443 31) (444 12) (458 89) (459 34) (460 10) |
| 377 | 2712,5 | 18,5015 | 101 | (53 10) (54 25) (55 269) (56 151) (57 999) (58 41) (65 2) (66 3) (67 40) (68 34) (69 199) (70 120) (71 675) (72 35) (81 20) (82 42) (83 156) (84 62) (85 437) (86 28) (95 9) (96 22) (97 125) (98 36) (99 118) (100 8) (109 3) (110 11) (111 59) (112 26) (113 70) (114 5) (123 1) (124 7) (125 28) (126 19) (127 47) (128 3) (138 4) (139 11) (140 14) (141 33) (142 3) (152 3) (153 6) (154 11) (155 24) (156 3) (166 2) (167 4) (168 8) (169 21) (170 3) (180 1) (181 2) (182 6) (183 15) (184 2) (194 1) (195 2) (196 5) (197 13) (198 2) (210 4) (211 10) (212 1) (224 3) (225 9) (226 1) (236 1) (238 3) (239 8) (240 1) (252 2) (253 6) (254 1) (266 2) (267 6) (268 1) (281 5) (294 2) (295 5) (296 1) (308 1) (309 3) (310 1) (322 1) (323 3) (336 1) (337 2) (338 1) (350 1) (351 2) (365 3) (378 1) (379 2) (392 1) (393 2) (394 1) (407 1) (450 1) |
| 378 | 3041,3 | 21,178 | 50 | (54 24) (55 260) (56 139) (57 999) (58 46) (67 45) (68 41) (69 211) (70 119) (71 677) (73 68) (81 33) (82 45) (83 186) (84 68) (85 461) (86 32) (96 29) (97 160) (98 41) (99 133) (100 8) (103 18) (110 14) (111 72) (112 28) (113 76) (114 8) (125 29) (126 23) (127 47) (129 27) (138 6) (139 10) (140 13) (141 36) (153 8) (154 9) (155 30) (168 6) (169 19) (182 8) (183 16) (197 12) (204 51) (205 11) (211 9) (217 25) (225 10) (239 8) |
| 379 | 2853,8 | 19,6517 | 80 | (53 9) (54 25) (55 276) (56 152) (57 999) (58 43) (66 3) (67 47) (68 36) (69 211) (70 122) (71 689) (72 36) (73 12) (81 31) (82 46) (83 170) (84 68) (85 468) (86 29) (91 6) (93 8) (95 23) (96 29) (97 145) (98 41) (99 130) (100 10) (109 8) (110 13) (111 67) (112 28) (113 73) (114 5) (121 3) (124 8) (125 27) (126 19) (127 48) (128 5) (137 5) (138 7) (139 12) (140 14) (141 32) (142 4) (152 4) (153 7) (154 10) (155 26) (156 4) (167 3) (169 17) (170 3) (182 5) (183 16) (184 2) (196 4) (197 12) (210 4) (211 10) (224 3) (225 9) (226 2) (238 2) (239 7) (253 8) (267 7) (282 3) (295 5) (308 1) (309 3) (323 3) (337 2) (343 3) (351 2) (365 2) (393 2) (407 2) (421 2) |
| 380 | 959,5 | 4,2326 | 23 | (59 77) (66 104) (67 22) (73 720) (77 57) (88 17) (92 12) (103 80) (115 28) (131 54) (133 70) (135 16) (147 999) (148 145) (149 76) (184 12) (191 100) (192 21) (193 14) (204 116) (206 17) (208 13) (282 7) |
| 381 | 963,5 | 4,2652 | 28 | (58 52) (59 101) (61 32) (66 112) (73 624) (74 80) (75 122) (88 24) (89 27) (103 136) (104 13) (108 11) (115 24) (122 11) (128 31) (131 49) (133 46) (147 999) (148 160) (149 78) (150 9) (152 19) (188 16) (191 107) (192 19) (193 8) (200 52) (201 5) |
| 382 | 972,8 | 4,3404 | 73 | (52 29) (53 36) (54 27) (55 175) (56 191) (57 999) (59 34) (60 47) (61 9) (63 43) (64 12) (69 236) (70 160) (71 342) (72 55) (73 329) (74 38) (76 26) (77 707) (78 74) (80 12) (81 26) (82 8) (83 17) (84 87) (85 238) (86 21) (90 32) (91 23) (92 8) (96 6) (97 17) (98 55) (99 42) (100 42) (102 6) (103 86) (104 23) (105 11) (106 7) (107 21) (108 5) (110 30) (112 23) (113 28) (116 6) (118 18) (119 5) (120 58) (121 5) (122 5) (127 9) (128 5) (130 26) (134 467) (135 35) (136 13) (137 4) (138 5) (140 59) (142 34) (143 69) (144 6) (184 405) (185 44) (186 13) (198 5) (214 199) (215 24) (216 8) (229 62) (230 6) (231 3) |
| alanine | 1116,5 | 5,424 | 33 | (55 13) (56 6) (57 15) (60 8) (61 14) (70 6) (71 20) (73 482) (74 46) (75 47) (85 11) (94 14) (98 3) (100 25) (101 14) (102 13) (103 37) (104 4) (114 5) (116 1000) (117 112) (118 39) (128 9) (129 5) (147 121) (148 15) (149 11) (159 2) (190 40) (191 9) (192 4) (217 15) (218 14) |
| arginine/citrulline/ornithine | 1834 | 11,277 | 136 | (57 3) (58 7) (59 97) (60 12) (61 6) (68 7) (71 4) (72 18) (73 1000) (74 102) (75 54) (85 5) (86 99) (87 12) (88 6) (90 4) (98 7) (100 93) (101 15) (102 60) (103 18) (104 4) (110 6) (112 17) (113 9) (114 35) (115 22) (116 16) (119 3) (126 20) (127 12) (128 63) (130 65) (131 32) (132 30) (133 33) (134 6) (135 3) (140 8) (142 997) (143 131) (144 58) (145 6) (146 48) (147 120) (148 22) (149 13) (150 2) (152 4) (153 3) (154 4) (160 16) (161 4) (162 4) (168 2) (169 7) (170 4) (172 50) (173 10) (174 466) (175 84) (176 46) (177 6) (178 1) (186 13) (187 9) (188 10) (189 21) (190 6) (191 11) (192 2) (193 2) (200 78) (201 18) (202 13) (203 9) (204 10) (205 3) (212 1) (213 2) (214 50) (215 14) (216 42) (217 15) (218 23) (219 5) (220 6) (221 2) (225 1) (226 3) (227 1) (228 1) (229 2) (230 4) (231 2) (232 4) (233 1) (234 1) (241 6) (242 5) (243 4) (244 4) (246 2) (248 1) (258 32) (259 30) (260 9) (261 3) (262 1) (287 4) (288 3) (289 1) (290 1) (291 1) (292 1) (301 1) (303 5) (304 1) (305 1) (313 1) (315 8) (316 5) (317 2) (318 1) (330 13) (331 6) (332 2) (403 2) (404 1) (405 4) (406 1) (420 43) (421 16) (422 8) (423 2) (424 1) |
| asparagine | 1686,7 | 10,221 | 102 | (58 15) (59 45) (60 11) (61 18) (62 1) (66 3) (70 13) (72 40) (73 1000) (74 127) (75 203) (76 16) (77 10) (84 9) (86 11) (87 5) (88 3) (90 8) (98 10) (100 121) (101 15) (102 13) (114 28) (115 36) (116 501) (117 59) (118 28) (119 6) (125 9) (126 2) (128 17) (130 28) (131 76) (132 223) (133 63) (134 15) (135 4) (140 2) (141 104) (142 21) (143 11) (144 16) (146 7) (147 148) (148 27) (149 23) (150 4) (156 4) (157 2) (158 5) (159 35) (160 5) (161 2) (163 2) (169 6) (170 2) (172 15) (173 4) (174 12) (175 3) (176 3) (188 139) (189 25) (190 20) (191 2) (199 4) (200 2) (202 43) (203 11) (204 15) (205 3) (206 3) (213 7) (214 5) (215 17) (216 13) (218 48) (219 9) (220 5) (228 1) (229 3) (230 2) (231 191) (232 43) (233 18) (234 3) (243 8) (244 5) (245 4) (246 1) (258 31) (259 7) (260 3) (262 2) (316 7) (317 2) (333 10) (334 3) (335 1) (348 8) (349 2) (350 1) |
| aspartate | 1532 | 9,034 | 112 | (54 2) (58 12) (59 48) (60 8) (61 12) (62 1) (66 4) (70 23) (72 28) (73 1000) (74 98) (75 135) (76 7) (77 3) (84 5) (86 9) (87 6) (98 7) (99 6) (100 232) (101 29) (102 13) (103 16) (104 2) (105 3) (113 1) (114 4) (115 17) (116 5) (117 51) (118 7) (119 12) (128 6) (129 4) (130 26) (131 26) (132 19) (133 58) (134 10) (135 6) (136 1) (140 1) (142 18) (143 6) (144 5) (145 2) (146 3) (147 189) (148 31) (149 29) (150 4) (151 1) (157 1) (158 3) (159 2) (163 20) (164 4) (165 2) (171 2) (172 11) (173 2) (174 14) (175 3) (177 4) (184 2) (188 64) (189 13) (190 8) (191 4) (192 1) (193 1) (200 1) (201 1) (202 66) (203 12) (204 16) (205 5) (206 2) (207 1) (214 1) (215 1) (216 25) (217 13) (218 131) (219 25) (220 11) (221 6) (222 1) (230 2) (231 1) (232 632) (233 124) (234 56) (235 9) (236 1) (243 2) (244 5) (245 5) (246 3) (247 1) (262 2) (292 9) (293 2) (294 1) (306 17) (307 5) (308 3) (334 7) (335 2) (336 1) (349 4) (350 1) |
| cellobiose | 2763,6 | 16,516 | 109 | (57 236) (58 15) (59 20) (69 61) (71 133) (72 15) (73 1000) (74 83) (75 51) (83 56) (85 113) (86 7) (89 39) (90 4) (97 29) (99 34) (100 12) (103 64) (111 18) (113 19) (114 9) (117 81) (125 7) (127 13) (133 29) (141 20) (142 6) (143 29) (147 278) (148 42) (153 6) (157 20) (158 6) (160 60) (163 7) (169 61) (170 10) (177 5) (183 10) (189 47) (190 9) (191 76) (192 12) (193 6) (204 507) (205 172) (206 59) (207 17) (210 8) (211 5) (215 7) (216 9) (217 247) (218 60) (219 23) (221 12) (229 18) (230 12) (231 23) (232 9) (233 13) (234 3) (239 3) (240 2) (241 5) (242 4) (243 40) (244 13) (245 13) (246 5) (247 6) (256 5) (259 7) (270 3) (271 39) (272 9) (273 9) (274 3) (275 3) (276 4) (291 8) (300 9) (301 4) (305 6) (319 45) (320 13) (321 7) (330 1) (331 24) (332 10) (333 7) (345 5) (351 5) (358 3) (359 5) (361 254) (362 79) (363 43) (364 9) (365 3) (373 3) (374 3) (390 3) (451 5) (452 2) (464 2) (480 20) (481 7) (482 5) |
| cellobiose(2) | 2790,1 | 16,642 | 243 | (53 3) (54 4) (59 24) (60 3) (61 4) (65 1) (66 2) (72 13) (73 1000) (74 83) (75 84) (76 4) (77 3) (82 8) (86 2) (87 4) (88 3) (89 38) (90 3) (91 3) (99 4) (100 6) (101 20) (102 4) (103 159) (104 15) (105 31) (106 3) (107 1) (110 1) (111 4) (112 2) (113 9) (114 6) (115 7) (117 87) (118 9) (119 8) (120 1) (121 1) (125 2) (126 1) (127 5) (128 5) (129 103) (130 17) (131 28) (133 60) (134 8) (135 5) (138 1) (139 2) (140 2) (142 5) (143 24) (144 4) (145 14) (147 310) (148 48) (149 35) (150 4) (151 2) (152 2) (153 3) (154 2) (155 12) (156 4) (157 16) (158 5) (159 5) (160 89) (161 19) (162 5) (163 9) (164 1) (165 1) (166 1) (167 1) (168 3) (169 52) (170 10) (171 6) (172 3) (173 6) (174 5) (175 8) (176 2) (177 5) (178 1) (179 1) (180 1) (181 2) (182 1) (183 2) (184 1) (185 2) (186 9) (189 40) (190 9) (191 61) (192 11) (193 6) (194 1) (196 1) (197 1) (198 1) (199 2) (200 1) (201 8) (202 3) (204 451) (205 159) (206 52) (207 15) (208 3) (209 1) (210 2) (212 1) (214 2) (215 6) (216 3) (217 209) (218 53) (219 24) (220 5) (221 11) (222 3) (223 2) (227 3) (228 3) (229 11) (230 8) (231 10) (232 4) (233 6) (234 3) (235 1) (237 1) (240 2) (241 4) (242 3) (243 41) (244 37) (245 16) (246 6) (247 4) (248 1) (249 1) (254 1) (255 2) (256 3) (257 6) (258 2) (259 5) (260 2) (261 1) (262 1) (263 1) (265 2) (266 1) (268 2) (269 5) (270 3) (271 41) (272 10) (273 5) (274 4) (275 2) (276 1) (277 2) (278 2) (279 1) (287 1) (288 1) (289 1) (291 9) (292 3) (293 2) (299 1) (300 9) (301 2) (302 1) (303 1) (304 1) (305 11) (306 4) (307 8) (308 2) (309 1) (313 1) (317 3) (318 3) (319 38) (320 12) (321 6) (322 1) (330 1) (331 14) (332 6) (333 4) (334 1) (335 1) (343 1) (344 1) (345 3) (346 1) (347 1) (358 1) (359 3) (361 313) (362 98) (363 47) (364 10) (365 4) (366 1) (369 1) (374 1) (376 2) (377 2) (378 1) (379 1) (383 1) (389 1) (390 6) (391 2) (392 1) (393 1) (412 1) (435 1) (436 1) (448 1) (449 1) (450 2) (451 5) (452 2) (453 1) (464 1) (480 6) (481 2) (482 1) (489 1) (495 3) (496 1) |
| D-ribose | 1699,3 | 10,313 | 131 | (54 3) (58 5) (59 45) (60 4) (61 6) (68 3) (72 19) (73 1000) (74 82) (75 69) (76 3) (82 3) (86 5) (87 5) (88 2) (89 52) (90 4) (100 18) (101 18) (102 8) (103 498) (104 45) (105 35) (106 3) (107 1) (112 2) (114 10) (115 6) (116 5) (117 48) (118 4) (119 7) (128 4) (129 36) (130 9) (131 25) (132 3) (133 65) (134 9) (135 6) (142 4) (143 10) (144 1) (145 6) (147 197) (148 31) (149 20) (150 2) (151 1) (157 2) (158 6) (159 2) (160 51) (161 11) (162 3) (163 8) (164 1) (168 3) (170 1) (172 3) (173 6) (174 7) (175 6) (176 1) (177 2) (186 1) (188 1) (189 80) (190 15) (191 21) (192 3) (193 1) (198 2) (200 2) (201 3) (202 1) (203 3) (204 17) (205 25) (206 5) (207 4) (208 1) (214 1) (215 1) (216 6) (217 255) (218 51) (219 22) (220 3) (221 5) (222 1) (228 5) (229 1) (230 1) (231 4) (232 3) (233 15) (234 5) (235 2) (242 2) (243 1) (246 1) (248 2) (256 2) (257 2) (262 7) (263 2) (264 1) (272 1) (274 3) (275 1) (276 1) (277 28) (278 8) (288 2) (290 1) (291 3) (292 1) (305 1) (306 1) (307 100) (308 28) (309 13) (310 3) (311 1) (316 1) (318 1) (319 1) (330 1) (362 2) (364 1) |
| fructose | 1900,1 | 11,718 | 147 | (59 29) (60 3) (61 4) (66 1) (73 1000) (74 84) (75 64) (76 3) (77 2) (87 4) (88 5) (89 48) (90 4) (91 3) (100 8) (101 13) (102 4) (103 460) (104 44) (105 20) (106 1) (114 8) (115 5) (116 6) (117 62) (118 7) (119 7) (120 1) (128 3) (129 33) (130 6) (131 24) (132 4) (133 76) (134 10) (135 6) (138 1) (142 5) (143 7) (144 1) (145 5) (146 1) (147 209) (148 33) (149 21) (150 2) (151 1) (156 2) (157 5) (158 3) (159 3) (161 1) (163 9) (164 1) (172 19) (173 16) (174 2) (175 6) (176 1) (177 4) (186 2) (187 1) (188 3) (189 39) (190 8) (191 20) (192 3) (193 2) (198 1) (200 2) (201 10) (202 8) (203 5) (204 20) (205 29) (206 6) (207 6) (208 1) (214 4) (215 2) (216 5) (217 300) (218 60) (219 27) (220 4) (221 8) (222 1) (223 1) (229 1) (230 3) (231 5) (232 2) (233 1) (235 2) (240 1) (242 2) (244 3) (245 1) (246 1) (247 1) (256 4) (257 1) (260 3) (261 1) (262 4) (263 6) (264 1) (270 1) (275 1) (276 2) (277 35) (278 9) (279 4) (280 1) (288 3) (289 1) (291 7) (292 2) (293 1) (300 1) (302 2) (303 1) (304 1) (305 2) (306 1) (307 117) (308 33) (309 16) (310 3) (318 2) (319 1) (330 2) (331 2) (332 2) (333 2) (334 4) (335 5) (336 2) (337 1) (344 1) (350 1) (364 17) (365 5) (366 2) (376 1) (464 1) (466 1) |
| fructose(2) | 1910,2 | 11,785 | 105 | (59 75) (74 167) (88 8) (89 148) (90 11) (100 17) (101 36) (103 1000) (104 89) (105 55) (114 29) (128 7) (133 125) (142 11) (147 566) (148 91) (149 71) (158 7) (163 14) (164 4) (170 4) (172 29) (173 39) (175 14) (189 83) (190 18) (191 48) (192 8) (193 4) (198 7) (200 4) (201 18) (202 20) (203 13) (204 59) (205 64) (206 15) (207 10) (214 5) (216 11) (217 791) (218 161) (219 64) (221 23) (223 3) (228 4) (230 9) (231 15) (232 4) (240 3) (241 8) (242 4) (244 14) (245 8) (246 3) (254 2) (256 4) (260 3) (262 49) (263 24) (264 7) (268 3) (270 3) (276 3) (277 86) (278 23) (279 12) (288 4) (291 13) (293 6) (302 3) (304 2) (305 8) (306 3) (307 302) (308 89) (309 42) (310 5) (315 8) (318 5) (330 5) (331 17) (332 7) (334 8) (335 10) (336 7) (337 2) (344 2) (350 5) (358 3) (364 44) (365 14) (366 7) (367 2) (376 5) (377 2) (390 3) (432 4) (433 3) (434 1) (448 1) (464 3) (466 4) (467 1) (468 1) |
| galactose | 1915,1 | 11,818 | 90 | (59 31) (72 16) (73 1000) (74 73) (88 3) (89 53) (90 5) (100 10) (101 14) (102 5) (103 117) (104 11) (105 29) (106 3) (114 11) (115 4) (130 8) (131 13) (133 56) (134 6) (135 5) (142 5) (143 7) (147 336) (148 54) (149 31) (157 54) (158 11) (159 3) (160 160) (161 28) (162 9) (163 10) (164 2) (172 3) (173 4) (175 4) (177 3) (178 1) (189 30) (190 8) (191 14) (192 3) (200 2) (201 5) (203 4) (204 20) (205 215) (206 42) (207 23) (210 3) (216 9) (217 88) (218 19) (219 5) (221 7) (229 23) (230 7) (231 7) (232 4) (233 5) (234 4) (244 2) (246 3) (262 4) (269 2) (274 6) (275 2) (276 1) (277 8) (278 3) (291 12) (292 4) (293 2) (305 8) (306 4) (307 6) (319 216) (320 64) (321 29) (322 6) (323 2) (343 2) (344 2) (364 2) (365 2) (366 1) (376 2) (434 1) (466 1) |
| glucose | 1928,6 | 11,908 | 215 | (53 2) (54 2) (55 8) (56 1) (58 6) (59 39) (60 3) (61 5) (66 1) (67 3) (68 1) (69 2) (70 4) (73 1000) (74 82) (75 70) (76 3) (77 2) (80 1) (81 1) (82 4) (83 5) (84 2) (85 3) (86 6) (87 3) (88 3) (89 51) (90 4) (91 3) (95 1) (96 1) (97 2) (98 2) (99 4) (100 13) (101 17) (102 9) (103 99) (104 10) (105 34) (106 3) (107 2) (109 1) (110 1) (111 2) (112 2) (113 4) (114 14) (115 9) (117 105) (118 11) (119 10) (120 1) (121 1) (125 1) (126 2) (127 4) (128 5) (129 131) (130 25) (131 34) (132 6) (133 77) (134 10) (135 6) (136 1) (138 1) (139 1) (140 1) (141 2) (142 6) (143 17) (144 3) (145 9) (147 420) (148 65) (149 40) (150 4) (151 2) (152 1) (153 1) (154 1) (155 2) (156 2) (157 99) (158 18) (160 180) (161 30) (162 9) (163 13) (164 2) (165 1) (168 2) (169 3) (170 2) (171 1) (172 3) (173 5) (174 2) (175 6) (176 1) (177 4) (178 1) (180 1) (182 1) (185 1) (186 4) (187 1) (189 43) (190 11) (191 19) (192 3) (193 2) (196 1) (198 1) (199 1) (200 2) (201 7) (202 2) (203 5) (204 34) (205 212) (206 41) (207 22) (208 3) (209 1) (210 4) (211 1) (212 1) (214 1) (215 3) (216 12) (217 115) (218 24) (219 11) (220 2) (221 9) (222 2) (223 1) (229 31) (230 9) (231 13) (232 4) (233 5) (234 4) (235 1) (240 1) (241 1) (242 1) (243 2) (244 4) (245 2) (246 4) (247 2) (248 1) (254 1) (256 2) (257 1) (259 1) (260 1) (262 5) (263 1) (264 1) (265 1) (268 1) (269 4) (270 2) (271 1) (274 8) (275 2) (276 2) (277 10) (278 4) (279 2) (290 1) (291 18) (292 5) (293 3) (297 1) (300 3) (301 1) (302 1) (303 1) (304 1) (305 10) (306 5) (307 8) (308 2) (309 1) (317 1) (319 265) (320 77) (321 38) (322 7) (323 2) (330 1) (331 1) (332 1) (333 1) (343 3) (344 1) (345 1) (358 1) (364 6) (365 3) (366 1) (374 3) (375 1) (376 2) (377 1) (390 1) (448 1) (464 1) (466 1) |
| glucose 6P | 2366,8 | 14,521 | 48 | (59 36) (73 1000) (74 89) (89 37) (101 47) (115 23) (116 25) (130 20) (131 42) (133 74) (147 122) (157 53) (160 90) (161 21) (170 12) (189 19) (204 20) (211 22) (217 55) (225 14) (227 10) (230 10) (231 12) (247 32) (248 8) (274 8) (299 186) (300 50) (301 24) (314 15) (315 67) (316 19) (317 9) (331 17) (332 8) (341 9) (357 66) (358 21) (359 8) (361 13) (362 6) (387 210) (388 67) (389 43) (390 10) (471 16) (472 5) (473 4) |
| glucose(2) | 1947,6 | 12,034 | 179 | (53 7) (57 44) (58 11) (59 39) (60 5) (65 3) (66 2) (68 3) (70 8) (71 8) (72 23) (73 1000) (74 87) (77 11) (79 3) (80 1) (85 8) (86 5) (87 5) (88 4) (89 57) (90 5) (91 8) (93 3) (94 1) (96 3) (98 9) (99 8) (100 13) (101 16) (102 7) (103 142) (104 14) (105 20) (106 2) (107 3) (109 4) (110 1) (111 6) (113 5) (114 4) (115 9) (119 18) (120 2) (121 3) (123 2) (124 1) (125 2) (126 3) (127 5) (128 4) (130 22) (131 45) (133 79) (134 12) (135 8) (136 1) (138 1) (139 1) (140 1) (141 2) (142 2) (143 16) (147 276) (148 42) (149 28) (150 4) (151 2) (152 1) (157 46) (158 8) (159 10) (160 112) (161 23) (162 6) (163 11) (164 2) (165 1) (167 1) (168 1) (169 4) (170 1) (172 4) (173 5) (175 11) (176 2) (177 11) (178 3) (180 1) (182 1) (186 4) (187 5) (188 3) (189 34) (190 8) (191 14) (192 4) (193 2) (196 1) (199 2) (200 2) (201 13) (202 3) (203 6) (204 26) (205 177) (206 34) (207 18) (208 3) (209 1) (210 1) (214 4) (215 4) (216 3) (217 80) (218 17) (219 9) (221 7) (222 2) (223 1) (228 1) (229 21) (230 6) (231 5) (232 3) (233 11) (234 3) (235 6) (236 1) (237 1) (240 1) (241 1) (242 2) (243 4) (244 2) (245 2) (246 2) (247 1) (256 1) (258 1) (259 1) (260 1) (262 1) (265 1) (268 3) (269 2) (270 1) (272 1) (273 2) (274 2) (275 1) (277 7) (278 3) (279 1) (285 1) (291 9) (292 3) (293 2) (302 1) (304 1) (305 6) (306 2) (307 6) (308 1) (309 1) (315 1) (319 167) (320 49) (321 24) (322 5) (323 1) (331 1) (332 1) (337 1) (342 1) (347 1) (365 1) (374 1) (376 1) |
| glutamate | 1629,2 | 9,8 | 131 | (54 6) (55 44) (56 63) (57 33) (58 17) (59 61) (60 48) (61 34) (62 2) (65 2) (66 6) (67 5) (68 6) (69 13) (70 12) (71 16) (72 35) (73 1000) (74 103) (75 238) (76 17) (77 10) (82 8) (83 15) (84 117) (85 22) (86 14) (87 12) (88 4) (89 3) (95 6) (96 4) (97 9) (98 17) (99 11) (100 91) (101 21) (102 30) (103 16) (104 2) (105 4) (109 2) (111 4) (112 14) (113 14) (114 25) (115 26) (116 11) (117 18) (118 3) (119 4) (124 1) (125 1) (126 4) (128 297) (129 69) (130 26) (131 30) (132 22) (133 67) (134 11) (135 7) (140 28) (141 3) (143 6) (147 217) (148 38) (149 49) (150 8) (151 3) (154 2) (156 150) (157 31) (158 29) (159 5) (160 4) (163 4) (172 4) (174 7) (176 2) (183 12) (184 2) (186 3) (188 2) (189 2) (200 12) (201 11) (202 9) (203 5) (204 25) (205 4) (206 2) (214 9) (215 2) (216 5) (218 32) (219 8) (220 3) (221 6) (222 2) (228 1) (229 1) (230 91) (231 19) (232 9) (244 2) (245 8) (246 730) (247 152) (248 64) (249 9) (250 2) (258 17) (259 4) (260 1) (272 1) (273 1) (274 8) (275 2) (320 8) (321 2) (322 1) (330 1) (332 1) (348 37) (349 11) (350 5) (351 1) (363 18) (364 6) (365 3) |
| glutamine | 1787,8 | 10,962 | 56 | (58 46) (59 52) (61 25) (72 36) (74 80) (75 324) (87 24) (100 93) (103 105) (104 27) (105 16) (114 62) (115 56) (116 61) (117 56) (126 21) (128 114) (129 185) (130 53) (131 79) (132 29) (134 16) (139 73) (140 18) (142 28) (145 64) (146 13) (155 371) (156 1000) (157 160) (158 48) (173 15) (175 31) (188 40) (189 103) (190 25) (200 9) (202 10) (203 57) (216 16) (221 36) (227 11) (228 11) (229 37) (230 21) (244 57) (245 170) (246 37) (247 13) (263 8) (272 12) (273 22) (301 16) (302 6) (348 11) (362 7) |
| glycerate | 1340,3 | 7,436 | 39 | (59 59) (72 17) (73 1000) (74 85) (75 89) (89 25) (101 44) (102 77) (103 153) (104 18) (105 13) (115 16) (116 15) (117 87) (130 23) (131 29) (133 187) (134 23) (135 15) (147 468) (148 70) (149 45) (175 15) (177 8) (189 258) (190 59) (191 27) (204 7) (205 69) (206 8) (207 7) (217 21) (221 8) (227 8) (292 148) (293 35) (294 18) (307 24) (308 6) |
| glycine | 1319,4 | 7,252 | 110 | (53 10) (54 4) (55 77) (56 32) (57 72) (58 23) (59 137) (60 12) (61 13) (66 3) (67 11) (68 6) (69 91) (70 43) (71 49) (72 25) (73 903) (74 78) (75 103) (76 6) (77 5) (81 4) (82 5) (83 45) (84 41) (85 52) (86 216) (87 25) (88 9) (95 4) (96 1) (97 13) (98 4) (99 7) (100 118) (101 26) (102 13) (103 11) (104 2) (105 5) (109 2) (110 5) (111 31) (112 8) (113 7) (115 10) (116 16) (117 40) (118 6) (119 12) (121 1) (125 8) (126 4) (130 38) (131 43) (132 11) (133 121) (134 17) (135 10) (144 10) (145 4) (146 3) (147 442) (148 68) (149 37) (150 4) (153 2) (154 3) (158 19) (159 5) (160 5) (161 2) (162 1) (172 17) (173 7) (174 1000) (175 183) (176 81) (177 14) (178 2) (187 1) (188 9) (189 3) (190 2) (202 5) (203 1) (204 4) (205 1) (206 1) (218 2) (246 11) (247 16) (248 149) (249 38) (250 18) (251 3) (252 1) (262 1) (276 40) (277 10) (278 5) (279 1) (325 2) (326 1) (341 6) (342 2) (343 1) (429 2) (430 1) (431 1) |
| glycolate/glyoxylate | 1090,9 | 5,187 | 40 | (59 75) (60 12) (61 15) (66 181) (67 20) (72 26) (73 1000) (74 88) (75 105) (81 31) (87 8) (88 25) (89 18) (95 5) (103 37) (104 6) (105 8) (115 15) (117 25) (131 43) (132 5) (133 104) (134 14) (135 9) (140 3) (147 996) (148 152) (149 93) (150 9) (151 4) (161 58) (162 11) (163 5) (177 126) (178 20) (179 8) (190 4) (205 78) (206 14) (207 6) |
| histidine | 1937,2 | 11,964 | 179 | (56 8) (58 12) (59 110) (60 13) (61 8) (66 3) (68 7) (70 8) (71 6) (72 21) (73 1000) (74 99) (75 82) (76 5) (80 2) (82 14) (83 5) (84 20) (85 12) (86 102) (87 13) (88 56) (89 3) (90 3) (91 4) (94 3) (97 4) (98 14) (99 9) (100 129) (101 18) (102 36) (103 15) (104 3) (105 1) (108 1) (110 5) (111 2) (112 30) (113 11) (114 40) (115 63) (116 21) (118 6) (119 5) (120 1) (124 4) (125 2) (126 14) (127 4) (128 164) (129 35) (130 60) (131 39) (132 27) (133 35) (134 6) (135 2) (136 1) (138 4) (139 2) (140 27) (141 6) (142 25) (143 6) (144 7) (146 33) (147 118) (148 22) (149 11) (150 2) (151 9) (152 3) (153 1) (154 40) (155 20) (156 416) (157 60) (158 29) (159 5) (160 14) (161 2) (162 3) (164 1) (166 5) (167 3) (168 5) (169 2) (170 6) (171 2) (172 21) (173 5) (174 575) (175 101) (176 48) (177 5) (178 1) (181 1) (182 2) (183 1) (184 3) (185 2) (186 18) (187 5) (188 7) (189 2) (190 2) (191 10) (192 2) (196 1) (198 2) (199 2) (200 41) (201 9) (202 17) (203 3) (204 2) (212 4) (213 5) (214 17) (215 8) (216 9) (217 4) (218 22) (219 7) (220 5) (221 1) (227 2) (228 31) (229 9) (230 109) (231 23) (232 11) (233 1) (239 1) (240 3) (241 1) (242 1) (243 1) (244 2) (245 1) (246 1) (254 1) (255 2) (256 2) (257 1) (258 3) (260 2) (262 1) (271 1) (272 5) (273 5) (274 3) (275 1) (277 1) (288 1) (301 1) (302 1) (303 1) (304 1) (317 138) (318 42) (319 17) (320 4) (321 1) (327 1) (329 13) (330 5) (331 2) (332 1) (391 2) (392 1) (419 4) (420 2) (421 1) (434 21) (435 8) (436 4) (437 1) |
| lactate | 1069,8 | 4,992 | 44 | (52 13) (53 5) (55 13) (58 34) (59 79) (60 10) (61 18) (66 108) (67 20) (72 33) (73 1000) (74 102) (75 106) (87 20) (88 44) (89 16) (94 3) (98 1) (101 27) (102 15) (103 11) (115 22) (117 690) (118 71) (119 14) (129 9) (131 38) (133 74) (134 10) (135 5) (142 1) (147 870) (148 131) (149 72) (150 8) (174 9) (175 8) (190 101) (191 127) (192 26) (193 8) (203 6) (219 33) (220 6) |
| malate | 1499,3 | 8,769 | 91 | (50 11) (51 18) (55 26) (58 26) (59 59) (60 9) (61 16) (66 4) (72 26) (73 1000) (74 92) (75 173) (76 14) (77 10) (87 4) (101 59) (102 10) (103 11) (105 4) (115 7) (116 14) (117 23) (118 4) (119 9) (129 14) (131 36) (132 6) (133 102) (134 16) (135 11) (136 3) (143 13) (144 3) (145 6) (146 3) (147 426) (148 67) (149 45) (150 6) (151 6) (157 5) (160 2) (163 2) (171 18) (173 4) (174 8) (175 39) (176 7) (177 11) (179 7) (180 2) (185 1) (189 51) (190 39) (191 38) (192 8) (193 4) (203 4) (204 3) (205 3) (207 3) (217 20) (218 5) (219 3) (221 11) (222 2) (223 2) (233 91) (234 18) (235 8) (245 50) (246 12) (247 7) (260 1) (263 13) (264 3) (265 17) (266 4) (267 2) (305 4) (306 5) (307 18) (308 5) (309 2) (319 6) (320 2) (321 1) (335 13) (336 4) (337 2) (350 1) |
| maltose | 2824 | 16,797 | 85 | (69 38) (73 1000) (74 85) (75 101) (81 14) (85 19) (89 41) (101 12) (103 151) (104 12) (105 17) (107 7) (117 65) (129 77) (130 12) (131 18) (133 50) (134 8) (143 20) (147 271) (148 44) (149 33) (157 11) (159 10) (160 56) (161 16) (163 8) (169 64) (170 10) (175 7) (177 11) (178 4) (186 8) (189 43) (190 10) (191 63) (192 12) (201 10) (202 4) (203 9) (204 518) (205 166) (206 60) (207 15) (214 8) (217 232) (218 57) (219 19) (221 11) (227 6) (228 4) (229 11) (230 7) (231 13) (233 7) (241 5) (243 47) (244 41) (245 13) (246 7) (259 9) (270 5) (271 49) (272 12) (273 10) (274 5) (291 9) (300 9) (305 10) (307 10) (318 7) (319 21) (320 8) (328 4) (330 3) (331 15) (332 9) (349 3) (361 355) (362 115) (363 51) (364 14) (365 6) (451 7) (480 4) |
| myoinositol | 2126,3 | 13,148 | 242 | (53 13) (54 27) (55 98) (56 18) (57 17) (58 12) (59 20) (60 4) (61 15) (62 1) (65 7) (66 6) (67 67) (68 23) (69 38) (70 13) (72 21) (73 1000) (74 85) (75 240) (76 16) (77 18) (78 4) (79 27) (80 12) (81 57) (82 24) (83 25) (84 25) (85 8) (86 3) (87 3) (88 2) (89 7) (90 1) (91 10) (92 2) (93 14) (94 9) (95 33) (96 36) (97 21) (98 22) (99 8) (101 9) (102 2) (103 36) (104 4) (105 8) (106 2) (107 9) (108 6) (109 18) (110 14) (111 11) (112 4) (113 5) (114 1) (115 5) (116 17) (117 123) (118 13) (119 15) (120 3) (121 10) (123 12) (124 6) (125 4) (126 2) (127 5) (128 2) (129 171) (130 21) (131 51) (132 17) (133 79) (134 14) (135 15) (136 5) (137 8) (138 5) (139 2) (141 3) (142 3) (143 26) (144 4) (145 31) (147 330) (148 50) (149 34) (150 8) (151 6) (152 5) (153 3) (155 8) (156 4) (157 9) (158 2) (159 5) (160 1) (161 6) (163 4) (164 5) (165 3) (166 7) (167 2) (168 1) (169 5) (170 2) (171 5) (172 2) (173 5) (175 5) (176 1) (177 7) (178 1) (179 2) (180 1) (181 1) (183 5) (184 14) (185 10) (186 2) (187 3) (188 1) (189 16) (190 9) (191 160) (192 29) (193 15) (194 2) (195 1) (197 1) (199 8) (200 2) (201 4) (202 1) (203 5) (204 71) (205 19) (206 10) (207 11) (208 9) (209 2) (211 1) (213 2) (215 5) (216 1) (217 289) (218 60) (219 26) (220 4) (221 32) (222 8) (223 4) (224 1) (225 1) (227 3) (228 1) (229 2) (230 6) (231 4) (232 2) (233 1) (235 1) (239 1) (241 2) (242 1) (243 7) (244 2) (245 2) (246 1) (248 4) (249 2) (250 6) (251 1) (255 2) (256 1) (257 2) (258 1) (265 51) (266 13) (267 7) (268 1) (269 1) (271 2) (272 1) (277 1) (278 1) (279 1) (281 1) (289 1) (291 26) (292 7) (293 9) (294 2) (295 1) (297 1) (303 1) (305 229) (306 66) (307 33) (308 8) (309 2) (316 1) (317 2) (318 131) (319 51) (320 22) (321 6) (322 1) (323 10) (324 3) (325 41) (326 10) (327 3) (328 1) (329 1) (331 1) (332 1) (340 2) (341 1) (343 5) (344 2) (345 2) (367 13) (368 4) (369 2) (379 1) (392 1) (393 8) (394 3) (395 2) (417 1) (418 1) (419 4) (420 1) (432 22) (433 17) (434 8) (435 3) (436 1) |
| ornithine/citrulline | 1768,9 | 10,823 | 122 | (53 16) (54 15) (55 19) (56 27) (59 130) (60 25) (61 14) (65 8) (67 28) (68 53) (69 26) (70 161) (72 21) (73 970) (74 183) (75 168) (76 19) (77 13) (80 20) (81 19) (82 14) (86 185) (87 27) (90 25) (91 15) (92 5) (93 11) (94 20) (95 20) (96 12) (97 18) (98 16) (100 118) (102 38) (106 7) (107 8) (108 14) (109 7) (110 9) (112 22) (113 9) (114 24) (115 25) (120 7) (121 6) (122 5) (123 9) (124 10) (126 13) (128 60) (129 26) (130 91) (131 57) (132 36) (133 24) (134 8) (135 5) (136 4) (137 6) (138 6) (140 9) (141 6) (142 243) (143 48) (144 31) (146 79) (147 122) (148 38) (149 18) (150 5) (152 6) (153 7) (154 21) (156 5) (158 19) (159 22) (160 17) (162 8) (169 16) (170 17) (171 5) (172 50) (173 14) (174 1000) (175 183) (176 80) (177 10) (178 2) (186 245) (187 47) (188 17) (189 5) (200 16) (201 5) (202 4) (203 3) (204 10) (215 7) (216 39) (217 18) (218 7) (220 5) (227 2) (230 3) (232 7) (243 13) (244 71) (245 18) (246 7) (257 3) (258 27) (259 13) (260 4) (318 3) (333 2) (347 2) (348 65) (349 21) (350 9) (351 6) (352 1) (366 1) |
