# Supplementary material for: Metabolic Adaptations of Benthic Forams: Foraminiferal Species Adaptations to Intertidal Mudflat Assessed by a Metabolic Approach
Source: J Eukaryot Microbiol. 2025 Nov 11;72(6):e70051. doi: 10.1111/jeu.70051 (PMC12605782; doi:10.1111/jeu.70051)
Supplement: Supplementary file 2 — Table S2: Normalized features intensities for three foraminiferal species: Elphidium oceanense, Haynesina germanica, and Ammonia confertitesta. Each sample (a, b, c) corresponds to a pool of specimens, whose sizes are detailed in Table 2. The values represent the area under the curve for each of the 107 features detected in at least three replicates of a given species. The areas under the curve were divided by the number of individuals in each pool and normalized to the internal standard (ribitol). [file JEU-72-e70051-s002.docx]

Table S2: Normalized features intensities for three foraminiferal species: *Elphidium oceanense, Haynesina germanica, and Ammonia confertitesta*. Each sample (a, b, c) corresponds to a pool of specimens, whose sizes are detailed in Table 2. The values represent the area under the curve for each of the 107 features detected in at least three replicates of a given species. The areas under the curve were divided by the number of individuals in each pool and normalized to the internal standard (ribitol).

| Species | *Elphidium oceanense* | | | *Haynesina germanica* | | | *Ammonia confertitesta* | | |
| --- | --- | --- | --- | --- | --- | --- | --- | --- | --- |
| Sample | Eo_a | Eo_b | Eo_c | Hg_a | Hg_b | Hg_c | Am_a | Am_b | Am_c |
| 2 | 60148937 | 72398323 | 79549477 | 106550464 | 80279324 | 140252766 | 80805867 | 96275599 | 99431243 |
| 6 | 370367875 | 421782079 | 444744389 | 361525402 | 373888083 | 241997993 | 338445537 | 148809949 | 273593955 |
| 8 | 699407948 | 1034129431 | 1010940080 | 926310736 | 810927389 | 933082305 | 1052862722 | 969642189 | 1135636508 |
| 11 | 0 | 0 | 0 | 61236471 | 49560054 | 81238930 | 57153008 | 153032839 | 118019353 |
| 13 | 0 | 0 | 0 | 249321016 | 116671791 | 224753745 | 118725761 | 159220188 | 156066650 |
| 17 | 13422021 | 18629996 | 16026008 | 47226583 | 36026712 | 119388807 | 83631889 | 74081921 | 88556304 |
| 18 | 2650903311 | 2932057621 | 2908305336 | 2906771900 | 2989158364 | 2789869221 | 2827393061 | 2873791503 | 3201215133 |
| 24 | 62776978 | 53630302 | 71923654 | 53869276 | 23364425 | 42834795 | 170194490 | 278959384 | 252199900 |
| 25 | 540933860 | 678751243 | 605540155 | 5089550443 | 3430870711 | 6954768989 | 3784304768 | 9131665042 | 7252317048 |
| 28 | 138224188 | 175007835 | 142930438 | 0 | 0 | 0 | 149207781 | 360113428 | 285983859 |
| 29 | 126927690 | 134690780 | 119164599 | 144757246 | 131782609 | 128201558 | 130156896 | 144051873 | 153968224 |
| 38 | 1039747399 | 2344712099 | 2804327865 | 2761525700 | 3379016483 | 2020986996 | 1072029931 | 476365341 | 763358869 |
| 42 | 0 | 0 | 0 | 218567199 | 105428178 | 208787402 | 197615606 | 815139917 | 499288473 |
| 52 | 72578573 | 112293717 | 50166688 | 362559748 | 147301054 | 333023900 | 306761418 | 577187590 | 435786861 |
| 53 | 25709152 | 66874436 | 73795006 | 71102164 | 75493110 | 73297637 | 49632903 | 55537654 | 59053267 |
| 59 | 822727736 | 1054421304 | 882581818 | 0 | 0 | 0 | 1047389913 | 780290181 | 901046286 |
| 63 | 2878663973 | 3449467716 | 2710543086 | 3061982240 | 2431764925 | 3919079479 | 3239866166 | 2557916831 | 2858307018 |
| 66 | 10102064 | 20033116 | 16896992 | 46682267 | 22898370 | 51033343 | 45826028 | 77424504 | 60762512 |
| 70 | 68678624 | 98942765 | 115350750 | 0 | 0 | 0 | 0 | 0 | 0 |
| 77 | 0 | 0 | 0 | 34282927 | 50915759 | 67548592 | 0 | 0 | 0 |
| 89 | 96578579 | 121117409 | 119393919 | 480813169 | 233916975 | 570580262 | 963942152 | 2800995487 | 1833524630 |
| 101 | 0 | 0 | 0 | 0 | 0 | 0 | 856762102 | 1676279890 | 1233591450 |
| 103 | 90983659 | 172143312 | 174876914 | 265524452 | 95873853 | 338670080 | 0 | 0 | 0 |
| 104 | 0 | 0 | 0 | 0 | 0 | 0 | 329977393 | 846914744 | 573146471 |
| 107 | 0 | 0 | 0 | 586329326 | 276247663 | 1205721773 | 0 | 0 | 0 |
| 113 | 0 | 0 | 0 | 0 | 0 | 0 | 46859264 | 481816811 | 257465249 |
| 114 | 0 | 0 | 0 | 105463057 | 113357135 | 121251213 | 83430696 | 829816590 | 695437808 |
| 115 | 25593553.5 | 27772386 | 23414721 | 0 | 0 | 0 | 0 | 0 | 0 |
| 116 | 0 | 0 | 0 | 152559586 | 18570839 | 168165042 | 1508639785 | 5106031866 | 5037072462 |
| 118 | 0 | 0 | 0 | 67690309 | 38142137 | 158047830 | 278954729 | 1133712457 | 1075746062 |
| 123 | 4712232322 | 4996493323 | 4369877677 | 7078473545 | 4588061558 | 7622343617 | 7929525947 | 1.6157E+10 | 1.8342E+10 |
| 133 | 0 | 0 | 0 | 71539227 | 18249808 | 76545410 | 0 | 0 | 0 |
| 134 | 0 | 0 | 0 | 111237707 | 35461455 | 147460037 | 35158374 | 191051319 | 172258681 |
| 135 | 66180074 | 78065977 | 89524889 | 0 | 0 | 0 | 178030623 | 715093283 | 680113854 |
| 136 | 57557293 | 96256192 | 214996757 | 0 | 0 | 0 | 64902636 | 433825074 | 379781151 |
| 139 | 0 | 0 | 0 | 118636880 | 11975935 | 65306407 | 12320735 | 84809935 | 73965005.2 |
| 141 | 0 | 0 | 0 | 373106547 | 94008495 | 582138375 | 0 | 0 | 0 |
| 144 | 46664230 | 45982229 | 38609537 | 2296478056 | 971744099 | 2134081132 | 373847676 | 886809000 | 959990059 |
| 145 | 318304411 | 339978359 | 293412933 | 2259464986 | 961123134 | 2538701997 | 2413745865 | 7053601325 | 7209384885 |
| 147 | 0 | 0 | 0 | 1184903528 | 327669275 | 2070085188 | 60598350 | 352869842 | 314856028 |
| 149 | 4785728893 | 4769160809 | 4710432677 | 5608994804 | 3248979647 | 5240394269 | 6004858719 | 1.2344E+10 | 1.3973E+10 |
| 153 | 0 | 0 | 0 | 0 | 0 | 0 | 73969769 | 932857719 | 766699132 |
| 154 | 18065618 | 19641761 | 16489475 | 0 | 0 | 0 | 0 | 0 | 0 |
| 158 | 0 | 0 | 0 | 0 | 0 | 0 | 96650548 | 458051251 | 422405420 |
| 161 | 0 | 0 | 0 | 23163509 | 27985022 | 32806536 | 0 | 0 | 0 |
| 163 | 0 | 0 | 0 | 1050848746 | 543375425 | 1524551064 | 0 | 0 | 0 |
| 165 | 170944267 | 149531320 | 135338364 | 177555611 | 191348772 | 205141933 | 950790309 | 4109445841 | 3853369828 |
| 166 | 194650787 | 122351285 | 134923739 | 0 | 0 | 0 | 0 | 0 | 0 |
| 167 | 34888309.5 | 31407611 | 38369008 | 3426956677 | 1136623511 | 4455212643 | 3285054883 | 1.3115E+10 | 1.2489E+10 |
| 170 | 0 | 0 | 0 | 0 | 0 | 0 | 96768008 | 230008820 | 248840555 |
| 171 | 0 | 0 | 0 | 160176561 | 11060905 | 131032405 | 231694110 | 1103935647 | 1017082060 |
| 173 | 0 | 0 | 0 | 0 | 0 | 0 | 451654948 | 1974742175 | 1847701409 |
| 200 | 0 | 0 | 0 | 0 | 0 | 0 | 126093594 | 1260857168 | 1056163005 |
| 201 | 0 | 0 | 0 | 34908421 | 0 | 91765757 | 19243650 | 35135839 | 41409980 |
| 209 | 0 | 0 | 0 | 1609665536 | 1035615895 | 2762413257 | 0 | 0 | 0 |
| 210 | 0 | 0 | 0 | 2148520679 | 1966191038 | 1579444671 | 2835422490 | 2738061020 | 4244207693 |
| 211 | 0 | 0 | 0 | 26433608 | 51788773 | 77143938 | 55339874 | 481910660 | 409116282 |
| 215 | 0 | 0 | 0 | 14338522 | 41303578 | 68268635 | 0 | 0 | 0 |
| 220 | 0 | 0 | 0 | 3.6215E+10 | 3.7088E+10 | 3.7962E+10 | 0 | 0 | 0 |
| 225 | 0 | 0 | 0 | 0 | 0 | 0 | 79195883 | 102298404 | 138207900 |
| 233 | 1459397049 | 1290012002 | 1259526080 | 2564900654 | 2280135280 | 2062779160 | 3006877657 | 3235852340 | 4753838893 |
| 241 | 0 | 0 | 0 | 694488596 | 267618233 | 946207449 | 86465793 | 440218624 | 401070184 |
| 242 | 0 | 0 | 0 | 40722075 | 27001233 | 45122710 | 487972433 | 1231846954 | 1309642463 |
| 245 | 604643883 | 223036765 | 513843560 | 592183474 | 185282447 | 746365529 | 98731127 | 643439524 | 565162951 |
| 250 | 0 | 0 | 0 | 0 | 0 | 0 | 652256386 | 2739803564 | 2583053652 |
| 253 | 0 | 0 | 0 | 0 | 0 | 0 | 1109456634 | 3472370388 | 3489061277 |
| 254 | 56280004 | 59515697 | 62751390 | 120213413 | 127869126 | 118094974 | 154979098 | 126609163 | 214429461 |
| 257 | 716170035 | 649527988 | 697619996 | 904387805 | 956384707 | 709118974 | 1060445114 | 1095883475 | 1210778503 |
| 258 | 719774768 | 752194418 | 744201350 | 0 | 0 | 0 | 826514882 | 960877276 | 1003620697 |
| 259 | 150405817 | 195026309 | 219587313 | 92887321 | 29034720 | 95320858 | 0 | 0 | 0 |
| 260 | 27562210 | 34076474 | 29719618 | 62494392 | 54154469 | 99132662 | 49988583 | 55407116 | 59179685 |
| 262 | 0 | 0 | 0 | 28766461 | 35306020 | 41845579 | 0 | 0 | 0 |
| 266 | 0 | 0 | 0 | 87050375 | 44032290 | 67235051 | 0 | 0 | 0 |
| 268 | 0 | 0 | 0 | 221846216 | 86379967 | 184961982 | 0 | 0 | 0 |
| 273 | 901499292 | 901289507 | 955094892 | 1332760263 | 1409728252 | 1086742979 | 1452592441 | 1431044127 | 1421632828 |
| 281 | 0 | 0 | 0 | 41768545 | 65343208 | 88917871 | 0 | 0 | 0 |
| 282 | 1035063634 | 1065319577 | 1091751844 | 1533459537 | 1638703296 | 1282870448 | 1727394513 | 1672560666 | 1676177903 |
| 283 | 509056455 | 763262019 | 743556535 | 546209291 | 383859815 | 732480807 | 677910386 | 577675782 | 619003981 |
| 306 | 1252399312 | 1280656062 | 1266945128 | 2048467277 | 2114603482 | 1765595088 | 2350077070 | 2197199928 | 2214523898 |
| 314 | 205277483 | 199300783 | 211254182 | 477940793 | 251385447 | 493933606 | 0 | 0 | 0 |
| 315 | 1413350053 | 1446814272 | 1386900287 | 2365622925 | 2402362638 | 2018612793 | 2621883251 | 2564368804 | 3949330940 |
| 318 | 0 | 0 | 0 | 514209839 | 692841142 | 871472445 | 0 | 0 | 0 |
| 339 | 1621186938 | 1623835494 | 1503334707 | 2745872376 | 2568346183 | 2348752250 | 3208417988 | 3283668946 | 4943724200 |
| 350 | 0 | 0 | 0 | 49056242 | 47805642 | 46555043 | 49572345 | 52587172 | 77794472 |
| 354 | 1752321904 | 1698250898 | 1606653643 | 2912385138 | 2800006608 | 2390096531 | 3373207699 | 3172962810 | 4984908843 |
| 362 | 0 | 0 | 0 | 1.003E+10 | 1.1182E+10 | 1.2335E+10 | 0 | 0 | 0 |
| 377 | 1792447157 | 1612270145 | 942993121 | 2270674542 | 2049241724 | 1750528036 | 2620040364 | 2788158355 | 4118343325 |
| alanine | 62063755 | 65249728 | 58877782 | 333335196 | 34287436 | 313979099 | 205817536 | 27851161 | 131204973 |
| arginine/citrulline/ornithine | 0 | 0 | 0 | 156104392 | 78554612 | 160788162 | 39867850 | 142215450 | 88674567.1 |
| asparagine | 0 | 0 | 0 | 0 | 0 | 0 | 73540831 | 396138840 | 231552078 |
| aspartate | 0 | 0 | 0 | 0 | 0 | 0 | 78239877 | 298999916 | 185979218 |
| cellobiose(2) | 133382309 | 142297021 | 124467596 | 0 | 0 | 0 | 326758916 | 730663663 | 805227294 |
| D-ribose | 48045232 | 19941221 | 33993226 | 0 | 0 | 0 | 592058272 | 667440268 | 620932780 |
| fructose | 11774126 | 34669886 | 23132780 | 35502106 | 32667098 | 70095998 | 39132181 | 61955361 | 49229633 |
| glucose | 297501076 | 486535591 | 400270765 | 287710663 | 200152775 | 218697531 | 4143898803 | 7858495619 | 5845166084 |
| glutamate | 0 | 0 | 0 | 72500590 | 106365874 | 140231157 | 150328569 | 699447751 | 418939726 |
| glycerate | 0 | 0 | 0 | 73304352 | 33261832 | 68462625 | 0 | 0 | 0 |
| glycine | 29044934 | 41873405 | 25016762 | 82368058 | 43760629 | 49217444 | 181843535 | 162024753 | 193082044 |
| glycolate/glyoxylate | 0 | 0 | 0 | 50500582 | 29091941 | 49341803 | 0 | 0 | 0 |
| histidine | 0 | 0 | 0 | 566577670 | 137542025 | 527125054 | 244146854 | 534110753 | 379011455 |
| malate | 0 | 0 | 0 | 54061271 | 44387159 | 34713048 | 0 | 0 | 0 |
| myoinositol | 0 | 0 | 0 | 0 | 0 | 0 | 658493908 | 1744718382 | 1830046159 |
| putrescine | 28009569 | 33656771 | 22362367 | 165900428 | 68890837 | 97398717 | 17455701 | 32259926 | 24509804 |
| saccharose | 30622525 | 172497225 | 64999849 | 35113923 | 39146468 | 207576384 | 53186302 | 128603475 | 138432915 |
| trehalose | 573326334 | 438235432 | 407111034 | 1.9582E+10 | 2.5805E+10 | 1.4933E+10 | 2.0793E+10 | 3.3587E+10 | 4.141E+10 |
| tyrosine | 0 | 0 | 0 | 96213464 | 108401023 | 120588581 | 128784478 | 440462295 | 277223178 |
| valine | 0 | 0 | 0 | 34081764 | 0 | 45216041 | 36800574 | 50353856 | 48937212 |
